# Knowledge Discovery in Databases of Proteomics by Systems Modeling in Translational Research on Pancreatic Cancer

**DOI:** 10.3390/proteomes13020020

**Published:** 2025-05-29

**Authors:** Mathilde Resell, Elisabeth Pimpisa Graarud, Hanne-Line Rabben, Animesh Sharma, Lars Hagen, Linh Hoang, Nan T. Skogaker, Anne Aarvik, Magnus K. Svensson, Manoj Amrutkar, Caroline S. Verbeke, Surinder K. Batra, Gunnar Qvigstad, Timothy C. Wang, Anil Rustgi, Duan Chen, Chun-Mei Zhao

**Affiliations:** 1 Department of Clinical and Molecular Medicine, Norwegian University of Science and Technology, 7030 Trondheim, Norway; 2 PROMEC—Proteomics and Modomics Experimental Core Facility at NTNU and the Central Norway Regional Health Authority, 7030 Trondheim, Norway; 3 Department of Pathology, Oslo University Hospital, 0372 Oslo, Norwayc.s.verbeke@medisin.uio.no (C.S.V.); 4 Institute of Clinical Medicine, University of Oslo, 0318 Oslo, Norway; 5 Department Biochemistry and Molecular Biology, University of Nebraska College of Medicine, Omaha, NE 68198, USA; 6 Department of Gastroenterology, St. Olav’s Hospital, 7030 Trondheim, Norway; 7 Division of Digestive and Liver Diseases, Herbert Irving Comprehensive Cancer Center, Columbia University Irving Medical Center, New York, NY 10032, USA

**Keywords:** proteomics, bioinformatics, signaling pathways, networks, research models

## Abstract

Background: Knowledge discovery in databases (KDD) can contribute to translational research, also known as translational medicine, by bridging the gap between *in vitro* and *in vivo* studies, and clinical applications. Here, we propose a ‘systems modeling’ workflow for KDD. Methods: This framework includes the data collection of a composition model (various research models), processing model (proteomics) and analytical model (bioinformatics, artificial intelligence/machine leaning and pattern evaluation), knowledge presentation, and feedback loops for hypothesis generation and validation. We applied this workflow to study pancreatic ductal adenocarcinoma (PDAC). Results: We identified the common proteins between human PDAC and various research models *in vitro* (cells, spheroids and organoids) and *in vivo* (mouse mice). Accordingly, we hypothesized potential translational targets on hub proteins and the related signaling pathways, PDAC-specific proteins and signature pathways, and high topological proteins. Conclusions: This systems modeling workflow can be a valuable method for KDD, facilitating knowledge discovery in translational targets in general, and in particular to PADA in this case.

## 1. Introduction

Translational medicine from basic to clinical research is a challenge, as the average rate of successful translation from animal models to clinical trials is less than 8% and about 90% of clinical drug development fails [1,2]. One of the obstacles in translational research is ‘mismatches’ between research models and human patients. Current efforts to overcome the obstacle include the development of more accurate and reliable animal models (e.g., humanized mouse models), improved cell line authentication, and patient-derived organoids with advantages as well as limitations in each model. In fact, it is not uncommon that there are similarities and differences between the research models, and it is common that selections of certain research models are dependent on the research purposes. In the field of human cancer modeling, *in vitro* models (e.g., cell lines and organoids) and *in vivo* models (e.g., mice) are widely used either alone or in combination. However, it should be noted that the cell lines used in 2D *in vitro* and spheroids in 3D *in vitro* experiments often diverge from their parental origins due to ongoing genetic modifications over the passages and adaptations to culture environments, and that the tumor organoid models in 3D *in vitro* experiments can change in the composition of cell types over time. The mouse models, as *in vivo* experiments, have species-specific differences which are often questioned about the accurate recapitulation of *de novo* human tumor development. Recently, we presented data collection on pancreatic cancer by gathering and analyzing accurate data from *in vitro* and *in vivo* models for translation research [3]. In this work, we further present a workflow of ‘systems modeling’ as a process of developing abstract models of a system, with each model presenting a different view or perspective of that system, for knowledge discovery in databases (KDD). The workflow consists of a data processing model (i.e., proteomics), composition model (different experimental models), data mining models (data selection and integration, and functional enrichment analysis and artificial intelligence/AI/ML, AI/ML), and knowledge presentation model. We validated this workflow focusing on ‘matched proteins’ between research models and human patients of pancreatic cancer.

Pancreatic cancer here refers to pancreatic ductal adenocarcinoma (PDAC), which is the third deadliest cancer in developed countries. It is characterized by an insidious clinical syndrome with aggressive malignant and dismal outcomes, with a 5-year survival rate of 13% [4,5]. Despite substantial research efforts, investment in PDAC has yet to yield significant improvements in both survival rates and the quality of life (QoL) over the past decades. In clinical practice, PDAC is seldom diagnosed at a stage when curative surgery is viable, primarily due to the absence of early detection biomarkers, even among high-risk populations. A common treatment in patients with inoperable PDAC is chemotherapy which may offer modest benefits [6,7]. Current ‘experimental’ treatments include immunotherapies and various targeted therapies, whereas palliative care focuses on symptom management to improve the QoL [8,9,10,11].

The development of molecular targeted treatments for PDAC relies on a profound understanding of its molecular biology. Currently, identified genetic mutations that serve as potential genomic biomarkers for PDAC include the activation of the oncogene KRAS, the inactivation of tumor suppressor genes, the inactivation of genome maintenance genes, and alterations in genes specifically involved in the homologous recombination repair pathway [12]. These mutations present both opportunities and challenges for targeted therapy. For example, the FDA approval of sotorasib for targeting the KRASG12C mutation has been applied to only 1–2% of PDAC patients who exhibited this specific mutation [13]. Several molecules that demonstrated promising preclinical efficacy failed in clinical trials, likely due to off-target effects such as intolerability, adverse reactions and failure to achieve objective responses. Together, these represent the challenge of the ‘translational gap’ in research on PDAC [14].

The present work is a continuation of our previous study [3], aiming to develop a workflow to identify the matched proteins across different research models in bridging the translational gap between laboratory research and clinical research. The matched proteins, together with their functional networks, provide a strong foundation for identifying therapeutic targets, biomarkers, and pathways that are more likely to hold clinical significance. Of note, the proteomics datasets used in the present study were designed to detect which proteins were presented rather than abundance/expression levels in an attempt to show the match targets between research models and patients.

## 2. Materials and Methods

### 2.1. Data Processing

The information about the different proteomics datasets used in this study can be found in our recently published paper entitled “Proteomics profiling of research models for studying pancreatic ductal adenocarcinoma” (DOI: 10.1038/s41597-025-04522-x). This publication details the preparation, validation and proteomic profiling of the research models, ensuring transparency and reproducibility. Aspects such as sample collection, proteomics analysis using LC-MS/MS and data processing, including quality control, were performed following the protocols outlined in that work. The corresponding proteomic datasets generated in the study are publicly available through the PRIDE database under accession numbers PXD057793, PXD057795, PXD057798, PXD057804, PXD057888, PXD057829, PXD057928, and PXD057607.

To ensure high-confidence protein identification, MS data were processed using MaxQuant (version 2.3.1.0) with standard filtering steps, including the removal of contaminants, reverse proteins, and proteins identified only by site modifications. Within each model, proteins were considered reliably detected if they appeared in at least 50% of biological replicates. This was indicated in our previous publication (DOI: 10.1038/s41597-025-04522-x). This filtering step reduced variability and ensured robust protein detection within each model.

### 2.2. Bioinformatic Analysis

RStudio (ggVennDiagram-package in Rstudio 1.0.136) and Cytoscape 3.10 were used. ClueGO analysis of the 1975 common proteins was performed in Cytoscape v3.9.1 (ClueGO v2.5.10) using the Homo sapiens (NCBI Taxonomy ID: 9606) and Reactome pathways (version 20.06.2024) as the reference set. In ClueGO, the Benjamini–Hochberg procedure with a one-sided *p*-value correction and a significance threshold of adjusted *p* < 0.05 were used on enriched (not depleted) pathways, while GO levels were restricted to 5–11 to balance specificity. Pathways were clustered using a Kappa score threshold of 0.4, which optimizes functional grouping by balancing sensitivity and specificity. It should be noticed that the ClueGO network is created with kappa statistics and reflects the relationships between the terms based on the similarity of their associated genes. False discovery rates were not applied, as the current study was not designed for null hypothesis testing of multiple comparisons. The significance of the terms and groups is automatically calculated. The initial group size of 1 and 50% sharing ratio were chosen based on ClueGO’s iterative algorithm, which first identified 180 groups and then merged redundant clusters in two iterations, yielding 121 final pathway groups. This ensured biologically meaningful pathway associations while minimizing weak overlaps. After applying significance selection criteria, 566 representative terms remained, covering 1597 genes (80.86%), while 340 genes (17.22%) lacked functional annotations.

### 2.3. AI/ML

AI was applied as a method to generate python scripts using ChatGPT (GPT 4o) (Large Language Model, retrieved from https://openai.com/chatgpt/, accessed 14 Febuary 2025). It facilitated rapid debugging and iterative refinement, allowing for the efficient implementation of complex ML pipelines (see below). To ensure robust evaluation, each model was trained and validated to allow for better generalization by reducing bias and variance. The AI-assisted scripting was used to automatically split the dataset, train models, and compute evaluation metrics, significantly reducing the need for time consuming manual labor. These metrics were calculated automatically for each model, with AI-assisted programming generating performance summaries. This ensured that the identification of the best predictive model and python v3.13.0 64-bit was used to generate scatter plots with ML-fitted curves for each model. The visualization provided a comparative analysis of model performance, making it possible to assess how well each model captured the relationship between degree and topological coefficient.

ML preprocessing: The dataset used in the regression analysis was obtained from a Cytoscape network analysis [15] made on the common 1975 proteins, where each node represents a protein, and edges indicate interactions between them. Each node in the network can be characterized by degree, which represents the number of connections it has, as well as topological coefficient, which is a measure of the similarity between a node’s connectivity pattern and that of its neighbors. The aim of the analysis was to investigate the topological coefficient based on the degree of each node, the reason being that degree as a fundamental centrality measure in network science and often correlates with a node’s functional importance. Given the complex and nonlinear nature of biological networks, traditional linear models may not properly capture the relationships between these variables, necessitating the use of AI/ML approaches. Data preprocessing involved extracting relevant information from the network-based dataset, specifically the node degree as the predictor variable (x) and topological coefficient as the target variable (y). No additional scaling of the input data was performed, as many tree-based models inherently handle input data scaling and normalization. The dataset was used in its entirety without filtering or transformation to allow models to learn from the full distribution of degree values. Furthermore, 5-fold cross-validation was applied to evaluate each model’s predictive performance. This method splits the dataset into five equal parts, using four for training and one for testing. Subsequently, it iterates the process across all five subsets to obtain an average performance score.

Evaluation: A diverse set of AI/ML regression models was applied to predict the topological coefficient from node degree. The models included tree-based ensemble methods, which are highly effective for capturing nonlinearity in structured datasets. They also included non-tree-based methods, which serve as baseline comparisons for evaluating alternative approaches. Among the tree-based ensemble methods, Random Forest [16,17,18] and Extra Trees [19] were implemented. These models are known for their ability to capture nonlinear relationships through recursive partitioning. They use multiple decision trees to improve prediction accuracy, with Random Forest and Extra Trees averaging multiple trees to reduce variance, while Gradient Boosting and CatBoost refine predictions to minimize errors. Gaussian Process Regression (GPR) [20] was included as a probabilistic approach that models the data using a kernel function to capture uncertainty and complex relationships between degree and topological coefficient. While highly flexible, GPR can sometimes be sensitive to noise and computationally expensive for large datasets. In addition, K-Nearest Neighbors (KNN) regression was included [21]. It relies on the proximity of data points in feature space to make predictions. KNN is effective in capturing local structures but may struggle with global generalization. Several linear and regularization-based models were also evaluated, including Bayesian Ridge Regression and ElasticNet Regression [22,23]. Bayesian Ridge is a probabilistic linear model that introduces regularization through a Bayesian prior, making it more robust to collinearity in the dataset. ElasticNet combines L1 (Lasso) and L2 (Ridge) penalties, balancing input variable selection with coefficient shrinkage to prevent overfitting. Decision Tree Regression [24] was included as a baseline tree-based model, providing a simple interpretable alternative to ensemble methods. Although useful for understanding decision boundaries, single decision trees often overfit the data and lack the predictive power of ensemble methods.

Training: Each AI/ML model was trained on the dataset using 5-fold cross-validation. The models were first trained on four subsets of the data and then validated on the remaining subset, with the process repeating five times to obtain an average performance score [25]. R^2^ Score (Coefficient of Determination) was calculated to measure the proportion of variance explained by the model [26]. An R^2^ score close to or like 1 indicates a better fit. Mean Absolute Error (MAE) measures the average absolute difference between predicted and actual values [27]. Lower values indicate better accuracy. The Mean Squared Error (MSE), not shown in graph, was visible in console when running script. MSE is similar to MAE but penalizes larger errors more heavily [27]. Hyperparameters for tree-based models were optimized to balance bias and variance. For example, Random Forest and Extra Trees were trained with 300 estimators (trees) and a depth limit of 6 to prevent overfitting. Gradient Boosting and CatBoost were fine-tuned with a learning rate of 0.05–0.1 to capture smooth nonlinear patterns. Gaussian Process Regression was configured with a Radial Basis Function (RBF) kernel for smooth nonlinear patterns.

Analysis: The analysis was conducted in the Visual Studio Code (v 1.100.0) software was used with Python-based computational tools for data processing, machine learning modeling, and visualization. Data handling and preprocessing were performed using Pandas for tabular data manipulation and NumPy for numerical computations [28,29]. For predictive modeling, various machine learning algorithms were applied using Scikit-Learn (v 1.5.1) [30] and CatBoost (v 1,2,7) [18]. Matplotlib (v 3.10.1) was used to visualize the fitted models against the original dataset [31]. The predictions generated by each model were visualized against the actual data points to assess their alignment with the observed trend.

Validation: To assess the generalizability of the ML models trained on the dataset of 1975-node protein interaction network, an independent validation dataset which is nerve-related proteins was used. Performance metrics, including coefficient of determination or R-squared (R^2^), mean absolute error (MAE), mean squared error (MSE), and their respective 95% confidence intervals, were calculated to assess prediction stability across subsets of the validation data. R^2^, MAE, and MSE were selected as complementary performance metrics to provide a comprehensive evaluation of model accuracy and stability. R^2^ measures the proportion of variance in the topological coefficient explained by the model, offering insight into how well the learned relationship captures overall trends in the data. MAE provides an interpretable average of absolute prediction errors, highlighting how close predictions are to observed values regardless of error direction. MSE, by squaring the errors, penalizes larger deviations more heavily and is particularly for identifying models that occasionally produce large outliers. Together, these metrics ensure both precision and reliability.

## 3. Results

### 3.1. Workflow of Systems Modeling for KDD

**Figure 1:**
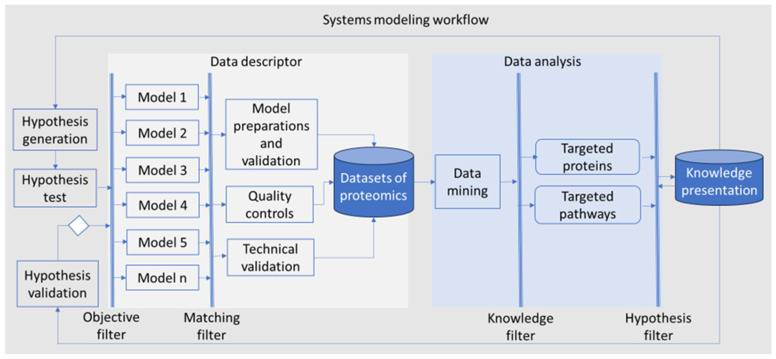
Schematic diagram of systems modeling. Hypotheses in translational medicine are formulated based on clinical observations (‘bedside’) and research findings (‘bench’). These hypotheses are subjected to ‘objective filter’ and then tested using one or more research models, clinical data and trials. In this study, five distinct models were employed, including murine PDAC cells, spheroids, organoids, orthotopic model, and human PDAC organoids. To address the translational gap, patient-derived materials were utilized as a ‘matching filter,’ ensuring that the models closely align with the human disease. The datasets included in this work comprised proteomic profiles from both research models and patient samples (showed in grey scare). The data analysis in this work (showed in light-blue squares) included data mining with functional enrichment analysis and machine leaning. A ‘knowledge filter’, as a ‘human-in-the-loop’ element, was employed for interpreting data, and a ‘hypothesis filter’ was used for the knowledge discovery, e.g., targeted proteins and signaling pathways. The knowledge presentation in this work can lead to the validation of a hypothesis and the generation of new hypotheses.

We hypothesized that the translational gap in PDAC research arises from mismatches between research models and actual patient conditions. To address this, we aimed to develop and validate the systems modeling workflow designed to identify ’translatable targets’ for PDAC. Our workflow incorporated a comprehensive range of PDAC models, including murine PDAC cells, murine normal pancreas tissue, murine PDAC spheroids, murine PDAC organoids, murine pancreatic exocrine organoids, human PDAC organoids and human PDAC tumor tissue for data collection, particularly on proteomics. Analytic methods included bioinformatics and AI/ML. With filters in each step, the knowledge on potential translational targets was discovered, which led to new hypothesis generation, test and validation (Figure 1).

### 3.2. Knowledge Presentation of Common Proteins Across Various Research Models and Human PDAC

**Figure 2:**
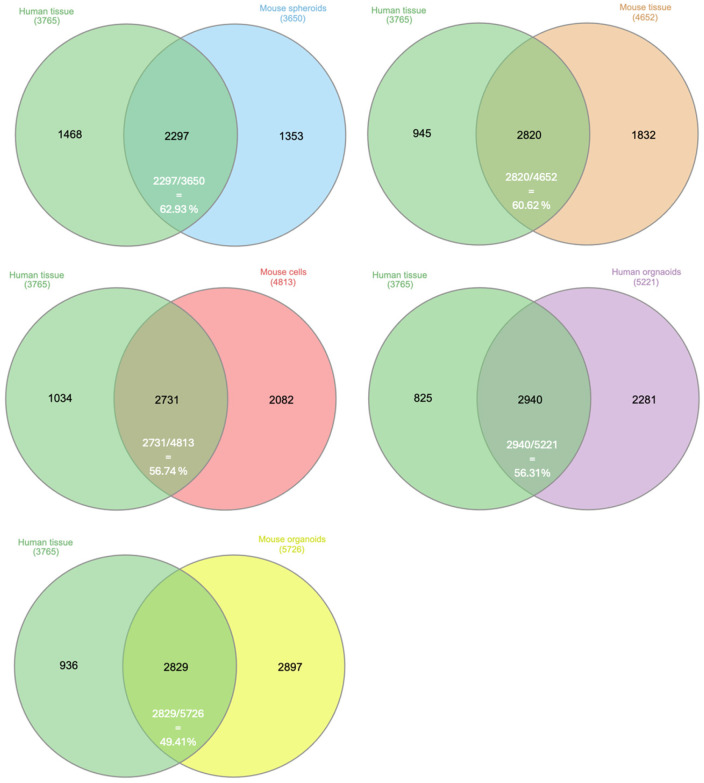
Venn diagrams comparing human PDAC with various PDAC research models. Note: Venn diagrams display the overlap of proteins identified in human PDAC tissue (*n* = 3765) with proteins detected in five different PDAC research models: mouse spheroids (*n* = 3650), mouse tissue (*n* = 4652), mouse cells (*n* = 4813), human organoids (*n* = 5221), and mouse organoids (*n* = 5726). The overlap percentages indicate the proportion of common proteins between human tissue and each research model, ranging from 49.41% (mouse organoids) to 62.93% (mouse spheroids).

**Figure 3:**
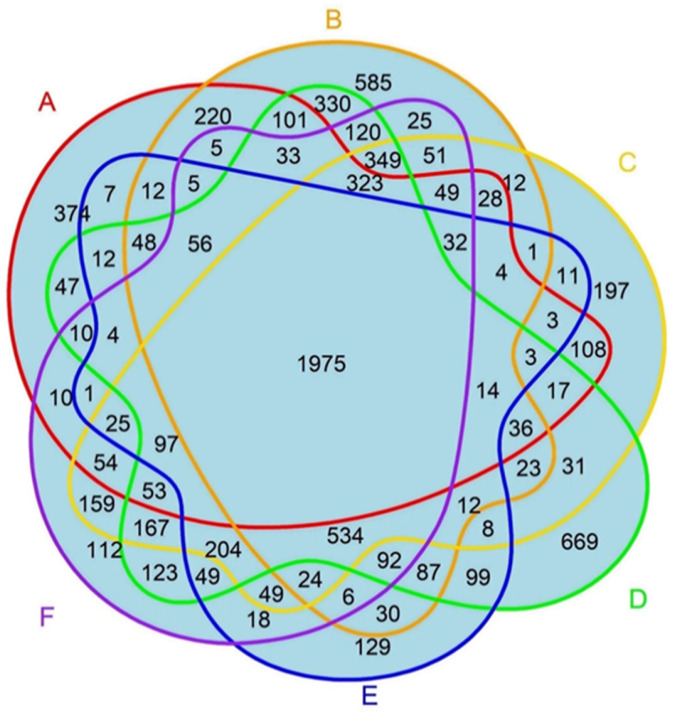
Venn diagram comparing six different PDAC models. Note: overlap of proteins identified among six PDAC groups: (A) Human PDAC tissue, (B) Human PDAC organoids, (C) Murine PDAC tissue, (D) Murine PDAC organoids, (E) Murine PDAC spheroids, and (F) Murine PDAC cells. For detailed information on each of 1975 proteins (IDs, genes, descriptions, locations, families and repurposing drugs), see Data S1 in Supplementary Materials.

Common proteins were defined as the proteins presented in the intersection of all models, as visualized in the Venn diagram. Only proteins that passed the initial filtering within each model and were subsequently found in all six models were considered the common proteins in the study. The research models shared proteins with human PDAC in a range between ~50 and 60% (Figure 2), highlighting both the existence of a translational gap and the potential for successful translation. A total of 1975 proteins that were commonly presented among all six PDAC research models are shown in Figure 3. The proportion of these shared proteins varied among the different models, ranging from 35% to 54%, is shown in Table 1.

### 3.3. Knowledge Presentation of Hub Proteins Across Various Research Models and Human PDAC

**Figure 4:**
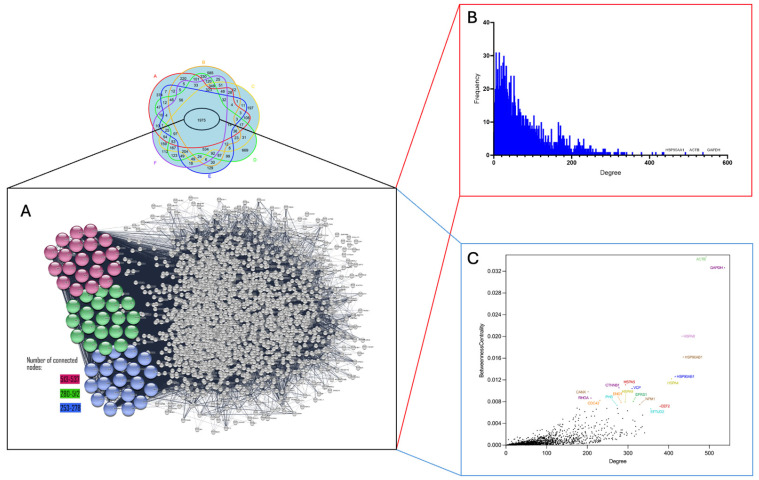
PPI of overlapped proteins (**A**), degree distribution (**B**) and betweenness centrality (**C**) across six research models. Note: the top 60 hub proteins are highlighted based on their degree of connectivity within the network. The top 20 proteins in purple, having 513–537 connective nodes, the next 21–40 proteins in green with 280–512 connections, and the proteins ranked 41–60 in blue with 253–278 connections (**A**). The degree distribution, where the *x*-axis represents the degree of connectivity (number of direct interactions) and the *y*-axis shows the frequency of each degree (**B**). Betweenness centrality is plotted against degree; the *x*-axis again represents the degree, while the *y*-axis measures betweenness centrality, indicating how often a protein acts as a bridge along the shortest path between two other proteins in the network (**C**). Each dot represents a protein, with ACTB exhibiting the highest betweenness centrality and degree, followed by GAPDH, HSPA8, and HSP90AB1. For full list of hub proteins, see Data S2 in Supplementary Materials.

PPI analysis of the 1975 common PDAC proteins identified several hub proteins, including GAPDH, ACTB, HSP90AA1, HSPA8, HSP90AB1, HSPA4, EEF2, EFTUD2, RPS3, and HNRNPA1, which emerged as the top 10 hub proteins based on Cytoscape network analysis. Each of these proteins had 300 or more node connections. The 1975 common proteins identified across all six PDAC research models were further analyzed for degree and betweenness centrality. Betweenness centrality was employed to assess a node’s significance by determining how frequently it acts as a bridge on the shortest path between two other nodes. ACTB showed the highest betweenness centrality and degree, followed by GAPDH, HSPA8, and HSP90AB1 (Figure 4, and Table 2).

### 3.4. Knowledge Presentation of Hub Canonical Pathways

**Figure 5:**
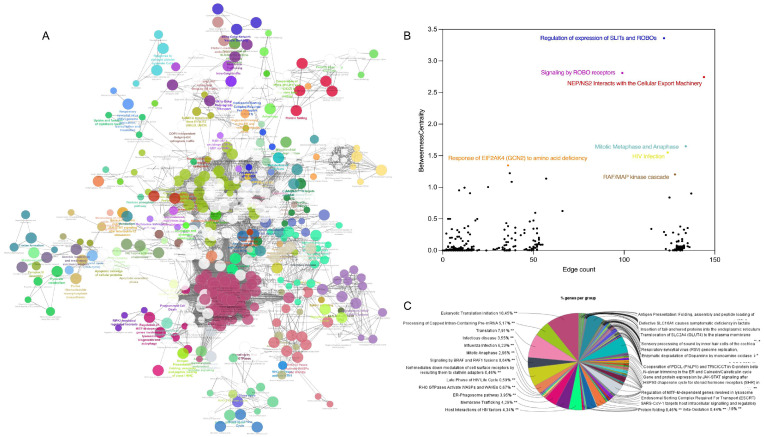
Network of hub canonical pathways (**A**), scatter plot of pathways in terms of betweenness centrality vs. edge count (**B**), and pie chart of distribution of genes per pathway (**C**). In (**A**), different colors represent different pathways when a node (representing a gene or a pathway) is divided into multiple colors, because the genes associated with that node are involved in multiple pathways or functional groups. In (**B**), each dot represents a pathway with *x*-axis in terms of edge count (degree centrality), and *y*-axis in terms of betweenness centrality. Pathways with higher edge counts are those that interact with more other pathways, while higher betweenness centrality values indicate pathways that serve as critical connectors or bridges within the network. The highlighted pathways represent those with the highest edge counts and significant betweenness centrality values, indicating their roles as critical hubs within the network. In (**C**), each sector represents a specific pathway or biological process, with the size of the sector corresponding to the proportion of genes from the protein dataset involved in that pathway. **: *p* ≤ 0.05 after Benjamini–Hochberg correction. For detailed information in C (names of pathways, term p values corrected with Benjamini-Hockberg and UNIQUE-IDs) see Data S3 in Supplementary Materials.

To gain deeper insights into PDAC progression and uncover potential early diagnostic biomarkers and therapeutic targets, hub canonical pathways were identified alongside hub proteins. Notably, several pathways with high betweenness centrality and a significant number of connections were highlighted, including the regulation of SLITs and ROBOs, signaling of ROBO receptors, and NEP/NS2 interactions with cellular machinery. Additionally, key canonical pathways such as eukaryotic translation initiation, processing of capped intron-containing pre-mRNA, and SRP-dependent co-translational protein targeting to the membrane were identified (Figure 5, and Table 3 and Table 4).

### 3.5. Knowledge Presentation of High Topological Proteins Across Various Research Models and Human PDAC

**Figure 6:**
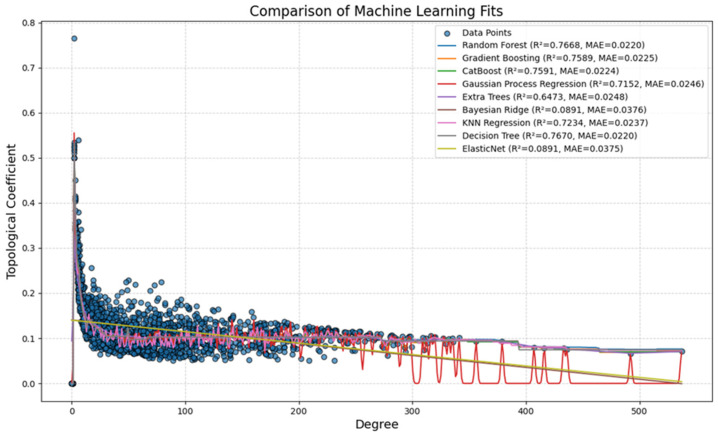
Scatter plot of topological coefficient against degree. Note: overlaid curves of various regression models including tree-based ensemble methods (Random Forest, Gradient Boosting, CatBoost, Extra Trees, and Decision Tree), probabilistic approaches (Gaussian Process Regression), distance-based methods (K-Nearest Neighbors), and linear regularization models (Bayesian Ridge and ElasticNet). Performance metrics, including the R^2^ (explained variance) and Mean Absolute Error (MAE), for each model are displayed in different colors.

The topological coefficient is a relative measure for the extent to which a node shares neighbors with other nodes. Nine AI/ML models were used and compared to determine which model best captured the relationship between node degree and topological coefficient in the network dataset consisting of the 1975 common PDAC proteins (Figure 6). Each model was trained using 5-fold cross-validation, and its predictive performance was assessed using three key evaluation metrics. R^2^ Score (Coefficient of Determination) shows how well the model explains variance in the data. The Mean Absolute Error (MAE) represents the average magnitude of prediction errors. Mean Squared Error (MSE) is similar to MAE but penalizes larger errors more heavily. Among the models tested, tree-based ensemble methods consistently outperformed other regression approaches. The Random Forest Regressor achieved the highest R^2^ score (0.7668) and the lowest MAE (0.0220), indicating that it was the most reliable model for predicting the topological coefficient. The Gradient Boosting Regressor (R^2^ = 0.7589, MAE = 0.0225) and CatBoost Regressor (R^2^ = 0.7591, MAE = 0.0224) performed nearly as well. This observation suggests that boosting-based AI/ML approaches are highly effective in modeling trends for topological coefficient. The performance differences between these models were marginal. This implies that any of these three models could serve as viable options for regression analyses on similar network datasets. The Gaussian Process Regressor (R^2^ = 0.7152, MAE = 0.0246) also provided a reasonable fit to the data but exhibited some instability, particularly at higher degree values, due to observed oscillations in predictions. This model captured nonlinear variations well but was more sensitive to noise in the dataset.

ML with Random Forest, Gradient Boosting, and CatBoost showed the best predictive accuracy, capturing the nonlinear relationship between degree and topological coefficient with minimal error. Comparably, linear models such as Bayesian Ridge and ElasticNet failed to capture the underlying trend, while Gaussian Process Regression exhibited instability at higher degrees (Figure 6). Of note, the distribution curves showed most nodes with coefficient more than zero and an inverse relationship between the coefficient and degree (Figure 6 and Table 5).

Several other models were tested, showing moderate predictive ability. The Extra Trees Regressor (R^2^ = 0.6473, MAE = 0.0248) introduces additional randomness to tree splitting, but performed worse than Random Forest. This suggests that the increased variance introduced by Extra Trees did not lead to improved generalization in this case. The K-Nearest Neighbors (KNN) Regressor (R^2^ = 0.7234, MAE = 0.0237) showed surprising effectiveness, likely due to its ability to capture local patterns in the dataset. However, it struggled to generalize globally across the entire degree range, which limits its applicability for large-scale network predictions.

Among the models that underperformed, the Decision Tree Regressor (R^2^ = 0.7670, MAE = 0.0220) was competitive in terms of R^2^ and MAE, but its predictions exhibited overfitting. This resulted in abrupt changes in predicted values across different degree values. In contrast, Bayesian Ridge Regression (R^2^ = 0.0891, MAE = 0.0376) and ElasticNet Regression (R^2^ = 0.0891, MAE = 0.0375) failed to capture meaningful relationships, as they assume a linear dependency between degree and topological coefficient, which does not align with the true nonlinear nature of the dataset.

Using the independent dataset with ensemble methods, particularly Random Forest, Gradient Boosting, and CatBoost, validation of the generalizability of the ML models achieved high predictive performance, with mean R^2^ values around 0.82, MSE values near 0.0014, and low MAE values (~0.024). The confidence intervals for R^2^, MAE and MSE were tight across all bootstrapped iterations, indicating model robustness despite the increased biological heterogeneity of the test set (Supplementary Table S1).

### 3.6. Knowledge Presentation of PDAC Signaling Network ‘Signature’

**Figure 7:**
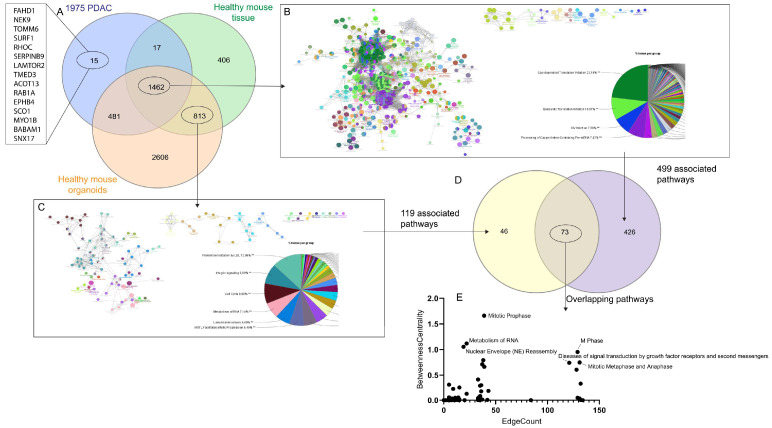
Comparative analysis of PDAC and non-cancerous models with pathway associations. Venn diagram depicting the 1975 common PDAC proteins and their overlapping with proteins from healthy mouse pancreatic tissue and healthy mouse pancreatic organoids. The unique proteins for PDAC are listed in the left column (**A**). Visualization of the pathways associated with the proteins common to all groups in the Venn diagram (i.e., overlapping proteins between PDAC models and healthy models) (**A**). The pathways are color-coded, and the pie chart provides a breakdown of the pathway categories (**B**). Pathways associated exclusively with proteins found in healthy models (non-cancerous) (**C**). Venn diagram showing the overlap between pathways associated with healthy models and cancerous PDAC models (**D**). This highlights the shared pathways. Scatter plot illustrates the relationship between edge count and betweenness centrality for the identified pathways (**E**). **: *p* < 0.01 corrected with Benjamini-Hockberg. For detailed information in (**D**), also see Data S3 in Supplementary Materials.

It is a common idea to identify specific biomarkers that are presented only by PDAC cancerous cells. By the systems modeling further filed with normal controls (i.e., normal mouse pancreas and normal mouse exocrine organoids), only 15 proteins were shown up (Figure 7A). Based on the TCGA dataset at Human Protein Atlas, the following were of low cancer specificity, including FAHD1, NEK9, SURF1, RHOC, LAMTOR2, TMED3, ACOT13, RAB1A, EPHB4, SCO1, MYO18, and BABAM1, whereas TOMM6 was not detected in cancer and SERPINB9 was highly abundant in testis cancer, respectively. Furthermore, 74% (i.e., 1462 out of 1975) PDAC proteins were also present in normal tissue, probably indicating that the ‘building blocks’ were similar but the ‘building appearance and function as “signature in terms of the signaling networks” differ between PDAC and the normal tissue (Figure 7B,C). Significant pathways of the PDAC proteins (‘1462’) in pie chart included Cap-dependent translation initiation, eukaryotic translation initiation, and processing of capped intron-containing pre-mRNA (Figure 7B), whereas significant pathways of the non-neoplastic proteins (‘813’) in pie chart included platelet sensitization by LDL, integrin signaling, cell cycle, metabolism of RNA, laminin interactions, and MASTL facilitates mitotic progression (Figure 7C). It should be noticed in terms of betweenness centrality vs. edges that 73 of these pathways were shared by the PDAC and the non-neoplastic proteins, such as metabolism of RNA, mitotic prophase, M phase, signal transduction by growth factor receptor, mitotic metaphase and anaphase and nuclear envelope reassembly (Figure 7D,E).

## 4. Discussion

**Figure 8:**
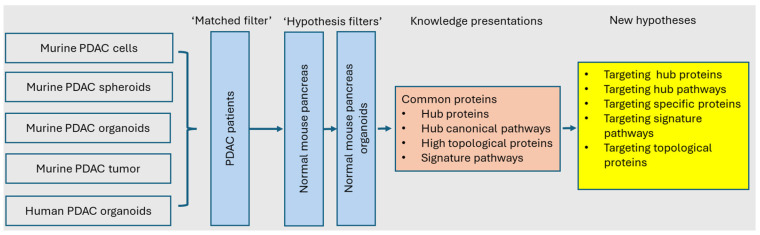
Summary of the main results. Nore: study models, filters, knowledge presentations and new hypotheses for the translational research of PDAC.

In the field of translational research, ‘systems biology’ is the state-of-the-art approach using multi-omics, such as transcriptomics and proteomics to acquire data for constructing models of complex biological systems and diseases [32,33]. However, extracting high-quality RNA from pancreatic tissue, including pancreatic tumors, poses significant technical challenges for many laboratories due to the high ribonuclease content in the pancreas [34,35,36]. It should also be noticed that discrepancies between mRNA expression and protein abundance are common, especially in the pancreas [37,38]. Thus, the datasets used in the present study were proteomics that were standardized and annotated from various PDAC research models and patient samples [3]. The results of the systems modeling showed the knowledge presentations, which led to the generation of hypothesis that the common proteins among the PDAC research models and patient samples, particularly hub proteins, hub canonical signaling pathways, high topological proteins and signature pathways, are the targets to overcome the ‘translational gap’ (Figure 8).

### 4.1. Targeting Common Proteins, Particularly Hub Proteins

The results of the present study showed about 35–50% of proteins being common across PDAC models and human PDAC tissues. Presumably, the tumor microenvironment contributes mostly to the mismatched proteins. The hub proteins, such as CCT4, EZR, and GAPDH, are known to be involved in critical pathways like neutrophil degranulation and sirtuin signaling, which are linked to the altered metabolic profiles and increased cancer stemness observed in neoadjuvant-treated PDAC. PPI analysis revealed that the distribution of protein interactions followed an inverse power law, indicative of a scale-free network topology, where correlations between degree and betweenness (“bottleneck-ness”) centrality confirmed the flow of communication within the network. Nodes with both high degree and high betweenness centrality are particularly significant as they lie on key communication paths and can regulate the flow of information within the network [39]. A comparative study in yeast identified a core set of 2708 proteins with an average of 5.26 PPIs per protein. In the present study involving 1975 proteins, the average number of PPIs per protein was 75.6 with the top 60 proteins exhibiting 253–537 PPIs each, reflecting the complexity of the PDAC network. As expected, housekeeping gene-encoded proteins, such as GAPDH and β-actin, emerged as top hub proteins that are crucial for cellular metabolism, homeostasis, motility, structure, and integrity. However, it would be impossible to target ACTB unless its specific isoforms for PDAC are identified. The next set of top hub proteins included heat shock proteins (HSPs), vital for maintaining cellular proteostasis through the integration of protein folding and degradation processes [40]. Notably, β-catenin, a well-known protein under investigation as a potential target in cancer treatment, particularly in PDAC, also ranked among the top hub proteins [41,42]. Ribosomal proteins, essential for the translation process and indicative of the high metabolic and proliferative demands of PDAC cells, also featured prominently among the hub proteins. When considering potential therapeutic targets, it is important to recognize that both the hub protein itself and its interaction partners must be specific in order to avoid off-target effects.

### 4.2. Targeting Hub Pathways

The present study showed the hub canonical pathways defined by hub proteins with high betweenness centrality and high edge count. For instance, the regulation of the expression of slit glycoprotein (SLIT) and roundabout receptor (ROBO) and signaling of ROBO receptors might represent new targets [43]. SLIT and ROBO are axon guidance molecules which has been suggested to play important roles in tumorigenesis, cancer progression and metastasis in general and in PDAC specifically [44,45]. NEP/NS2 interacts with cellular machinery is known to be involved with the influenza virus NEP (NS2 protein) [46], and has not been reported in connection with cancer, particularly PDAC (until this study). The present study also identified the hub canonical pathways defined by the percentages of its hub proteins included eukaryotic translation Initiation [47], processing of capped intron-containing pre-mRNA [48], and SRP-dependent co-translational protein targeting to membrane [49,50]. Of note, high betweenness of PTM (0.9517) is aligned with the strong impact on the connectivity of the network.

Several additional highly connected pathways were identified, including the RAF/MAP kinase cascade, mitotic metaphase and anaphase, and response of EIF2AK4 (GCN2) to amino acid deficiency, all of which are relevant to cancer progression and therapy resistance. The RAF/MAP kinase cascade is a well-established driver of PDAC proliferation and survival, frequently altered in pancreatic tumors. Similarly, mitotic metaphase and anaphase regulation is crucial for tumor cell division and chromosomal stability, making it a potential therapeutic target. The EIF2AK4 pathway, involved in the cellular response to amino acid deprivation and metabolic stress, has been linked to tumor adaptation under nutrient-limited conditions, which is particularly relevant for PDAC’s hypoxic and nutrient-deprived microenvironment. The identification of these pathways supports their potential roles in tumor progression and resistance mechanisms in PDAC and highlights key regulatory networks that may be further explored for therapeutic targeting.

### 4.3. Targeting Specific Proteins

Despite of hard attempts during the past several decades, no specific proteins or protein signatures have been conclusively identified as reliable clinical indicators for risk assessment, early detection, progression, treatment response, or prognosis for PDAC. While the 15 proteins identified in the present study are unlikely to serve as definitive PDAC-specific biomarkers, the broader set of 1.462 proteins may hold potential as PDAC-unique proteins, particularly when considering personalized medicine approaches, such as filtering with individual patient proteomic data. Additionally, the 813 ‘normal’ proteins, identified by filtering against normal mouse pancreas and pancreatic organoids, could be further refined using normal human pancreatic protein data. The systems modeling approach is adaptable, allowing for various types of ‘filtering’—for example, isolating cancerous cells from the tumor to exclude contributions from the tumor microenvironment, including immune cells. It is believed that non-neoplastic cells within the tumor, such as those associated with chronic pancreatitis, may increase the risk of PDAC. The findings of the present study may inspire further research into identifying PDAC initiators or promoters beyond inflammation, such as cytokine interleukin 33 [51].

### 4.4. Targeting Specific Pathways

The ‘PDAC signaling network signature’ proposed in the present study highlights the pivotal roles of signaling pathways associated with translation initiation, including cap-dependent translation initiation, eukaryotic translation initiation, and the processing of capped intron-containing pre-mRNA [52,53,54,55,56]. These pathways are central to the functionality of PDAC cells, reflecting their high translational activity and protein synthesis demands. Additionally, the significant pathways identified in non-neoplastic proteins may be viewed as key contributors to the ‘PDAC tumor microenvironment’ excluding immune cells. These pathways include metabolism of RNA, platelet sensitization by LDL, integrin signaling, cell cycle regulation, laminin interactions, and MASTL-facilitated mitotic progression [57,58,59,60,61,62,63]. Moreover, potential communication pathways between cancerous cells and tumor microenvironment cells appear to be active during mitotic phases, including prophase, M phase, metaphase, and anaphase, as well as during nuclear envelope reassembly. Diseases of signal transduction involving growth factor receptors and second messengers further underscore the complexity of the interactions within the PDAC microenvironment [64].

### 4.5. Targeting Topological Proteins

It is generally believed that proteins with high topological coefficients in a network are key nodes that are similar to the hubs in a network structure. By effectively identifying and protecting these critical nodes, the robustness of the network can be improved, making it more resistant to external interference and attacks [65]. However, the present study showed the negative correlation between these two variables, probably suggesting a potential biological significance. For instance, Glyceraldehyde 3-phosphate dehydrogenase (GAPDH) and beta (β)-actin (ACTB) that are housekeeping gene-coded proteins exhibited the highest degrees but low topological coefficients, whereas anoctamin 10 (ANO10) that manifests chloride channel activity exhibited the highest coefficient but degree of 2. There is little literature on the top key nodes in connection with PDAC yet, which is worthful for further investigation. There is little literature on the top key nodes in connection with PDAC yet, which is worthful for further investigation.

### 4.6. Notes on AI/ML

The results of the present study showed that the tree-based models (Random Forest, Gradient Boosting, and CatBoost) produced smooth well-aligned curves that closely followed the distribution of actual data points. Their ability to learn from the underlying structure of the dataset resulted in highly accurate predictions with minimal deviation from observed values [16,17,18]. In comparison, the Gaussian Process Regressor displayed high variability, specifically in regions where data density was low [20]. The Decision Tree Regressor generated highly fragmented predictions, further highlighting its tendency to overfit individual training points rather than generalizing globally across the dataset [24].

Linear models, including Bayesian Ridge and ElasticNet, produced nearly flat trend lines with little predictive power [22,23]. This implies that simple linear dependency is inadequate for modeling topological coefficients in biological networks. Given the forementioned findings, Random Forest emerges as the most effective model, with Gradient Boosting and CatBoost as strong alternatives. These results suggested that ensemble tree-based methods are optimal for modeling the topological coefficient in network analysis, as they effectively handle nonlinearity and complex interactions between degree and connectivity patterns.

The validation of the generalizability of the ML models tested on the independent dataset (neural related proteins) suggested that the topological relationships between topological coefficient and node degree learned from a general proteomic network are preserved across disease-specific and neurol contexts. The ability of models to maintain high predictive accuracy on the independent and biologically diverse dataset supports that the generalizability of ML models in studying protein network behavior.

### 4.7. Limitations

The actual complexity of proteomes and their constituent proteoforms were not addressed in the present study, as the methods such as bottom-up/shotgun/proteogenomic approaches are not available yet for it. Canonical protein identifications are inferred, intact full-length species are assumed, and the current methods cannot address the full range of potential proteoforms. Weaknesses of the present study included small sample size in some research models and human tissue samples (three patients) and limited research models, although the present study was not designed for comparations in terms of abundant levels. Of note, the limitation of small size can be mitigated and even overcome by including more available PDAC proteomic datasets, yielding a more robust set of common/matched proteins and potential targets. This workflow can be enriched by incorporating other models and filters, such as human cell lines, human spheroids, genetically engineered mouse models, tumor microenvironment components like fibroblasts and cancer-associated fibroblast organoids, and liquid proteomics.

## 5. Conclusions

The systems modeling workflow developed in the present study may provide a robust framework for bridging the ‘translational gap’ by identifying potential ‘matched targets.’ The findings, along with the predictions and hypotheses generated, warrant further validation through additional models. Moreover, this approach supports the refinement of existing hypotheses and the data presented in connection with the present study may open avenues for generating new hypotheses related to PDAC initiation, early detection, and prevention strategies.

## Figures and Tables

**Table 1:** Proportion of common proteins across research models.

Research Model	% of Common Proteins in Research Models
Murine PDAC cells	1975/4813 = 41.0%
Murine PDAC tumor tissue	1975/4652 = 42.5%
Murine PDAC spheroids	1975/3650 = 54.1%
Murine PDAC organoids	1975/5726 = 34.5%
Human PDAC organoids	1975/5221 = 37.8%
Human PDAC tumor tissue	1975/3765 = 52.5%

**Table 2:** Top 10 hub proteins. The hub proteins are ranked by their degree of connectivity within the network. The proteins are highlighted with colors corresponding to the clusters depicted in Figure 4A.

Gene Name	Degree	Betweenness Centrality	Description
HSP90AA1	436	0.01622	Heat shock protein 90 alpha family class A member 1
HSPA8	433	0.02005	Heat shock protein family A (Hsp70) member 8
HSP90AB1	416	0.01260	Heat shock protein 90 alpha family class B member 1
EEF2	379	0.00714	Eukaryotic translation elongation factor 2
VCP	310	0.01041	Valosin containing protein
RPL3	297	0.00179	Ribosomal protein L3
HSPA5	294	0.01109	Heat shock protein family A (Hsp70) member 5
HSPA9	293	0.00795	Heat shock protein family A (Hsp70) member 9
CTNNB1	278	0.01054	Catenin beta 1
PHB	273	0.00736	Prohibitin 1

**Table 3:** Connections of pathways sorted by betweenness centrality of edge counts. The edge count is the sum of indegree and outdegree. Here, the pathways are sorted on the betweenness centrality.

Term	Betweenness Centrality	Edge Count	Indegree	Outdegree
Regulation of expression of SLITs and ROBOs	3.3599	122	15	107
Signaling by ROBO receptors	2.8086	99	65	34
Disorders of transmembrane transporters	2.7432	144	64	80
Mitotic metaphase and anaphase	1.6494	134	96	38
Response of EIF2AK4 (GCN2) to amino acid deficiency	1.3463	36	9	27
Nuclear envelope (NE) reassembly	1.2219	37	16	21
RAF/MAP kinase cascade	1.2031	128	58	70
Nonsense-mediated decay (NMD)	1.0862	38	10	28
SARS-CoV-1-host interactions	1.0032	25	8	17
MAP2K and MAPK activation	0.9931	12	8	4
Post-translational protein modification (PTM)	0.9517	9	3	6
Metabolism of RNA	0.9059	15	2	13
Deubiquitination	0.8379	125	52	73
Signal transduction by growth factor receptors	0.6253	66	31	35
Defective TPR towards thyroid papillary carcinoma	0.5965	51	14	37
Energy-dependent regulation of mTOR by LKB1-AMPK	0.5000	4	1	3
Protein localization	0.5000	3	1	2
SARS-CoV-2-host interactions	0.4988	19	4	15
SARS-CoV-1 Infection	0.4758	18	8	10
Mitotic prophase	0.4741	50	11	39
tRNA processing	0.4574	48	6	42
Metabolism of amino acids and derivatives	0.4316	35	16	19
Infectious disease	0.3851	14	7	7
Axon guidance	0.3843	36	20	16
Fc epsilon receptor (FCERI) signaling	0.3788	131	97	34
Transport of small molecules	0.3603	40	30	10

**Table 4:** Top 30 canonical pathways based on 1975 common proteins. The pathways are sorted based on the *p*-value.

Ingenuity Canonical Pathway	*p*-Value
Eukaryotic translation initiation	5.01187 × 10^−76^
Processing of capped intron-containing pre-mRNA	5.01187 × 10^−69^
SRP-dependent co-translational protein targeting to membrane	1.58489 × 10^−64^
EIF2 Signaling	2.51189 × 10^−61^
Eukaryotic translation elongation	3.98107 × 10^−57^
Nonsense-mediated decay (NMD)	1.58489 × 10^−56^
Eukaryotic translation termination	3.16228 × 10^−56^
Response of EIF2AK4 (GCN2) to amino acid deficiency	1 × 10^−51^
Selenoamino acid metabolism	6.30957 × 10^−50^
Major pathway of rRNA processing in the nucleolus and cytosol	3.98107 × 10^−44^
Sirtuin signaling pathway	7.94328 × 10^−40^
Mitochondrial dysfunction	1 × 10^−37^
BAG2 signaling pathway	3.16228 × 10^−35^
RHO GTPase cycle	5.01187 × 10^−35^
Huntington’s disease signaling	1.99526 × 10^−34^
Intra-Golgi and retrograde Golgi-to-ER traffic	3.16228 × 10^−33^
Regulation of eIF4 and p70S6K Signaling	3.98107 × 10^−33^
Mitotic metaphase and anaphase	5.01187 × 10^−33^
Protein sorting signaling pathway	1.58489 × 10^−32^
Microautophagy signaling pathway	1.58489 × 10^−30^
Protein ubiquitination pathway	1.99526 × 10^−28^
NIK-noncanonical NF-kB signaling	3.16228 × 10^−28^
Electron transport, ATP synthesis, and heat production by uncoupling proteins	1.25893 × 10^−27^
Regulation of apoptosis	3.16228 × 10^−27^
Granzyme A signaling	7.94328 × 10^−27^
FAT10 signaling pathway	7.94328 × 10^−27^
COPI-mediated anterograde transport	1.25893 × 10^−26^
Hedgehog ligand biogenesis	2.51189 × 10^−26^
mTOR signaling	7.94328 × 10^−26^
Estrogen Receptor Signaling	3.16227 × 10^−25^

**Table 5:** Topological coefficients and degree values for nodes representing a single gene/protein. Data cutoff at topological coefficient ≥0.4 and degree ≥300. High topological coefficient coincides with low degree and high degree coincides with low topological coefficient.

Gene/Protein	Topological Coefficient	Degree
ANO10	0.765151515	2
ZNRD2	0.539386401	6
NADK2	0.534246575	2
DDT	0.528776978	2
TINAGL1	0.525974026	2
NIBAN2	0.52300885	2
TM9SF3	0.518518519	2
OXR1	0.516587678	2
COBLL1	0.511111111	2
ACP6	0.5	2
CYP20A1	0.5	2
DNPH1	0.5	2
IKBIP	0.5	2
LRRC1	0.5	2
MISP	0.450084602	3
HDGFL2	0.414609053	3
DTD1	0.410480349	3
LAD1	0.409883721	3
IRF2BP1	0.406926407	3
HERC4	0.403508772	3
RPS16	0.105157843	316
RPL4	0.102499291	320
RPSA	0.101460442	319
RPS3	0.101350676	341
RPL5	0.101052058	309
RPLP0	0.100468112	327
RPS20	0.100010894	335
EEF1A1	0.099431337	312
RPS9	0.098880581	319
RPS2	0.098282586	332
RACK1	0.098023878	317
EEF2	0.093276023	379
EFTUD2	0.092734482	356
HNRNPA1	0.091633486	336
EPRS1	0.089226511	313
NPM1	0.087729822	329
HSPA4	0.079882921	407
HSPA8	0.078043369	433
HSP90AB1	0.077569299	416
HSP90AA1	0.074854808	436
VCP	0.074248602	310
GAPDH	0.07078761	537
ACTB	0.066307787	492

## Data Availability

Data in connection with the figures are available in Supplementary Materials.

## Supplementary Materials

The following supporting information can be downloaded at: https://www.mdpi.com/article/10.3390/proteomes13020020/s1, Supplementary Table S1: Bootstrapped model validations in terms of R2, MAE and MSE; Data S1: Common proteins (pages 3–56); Data S2: hub proteins (page 57–104); Data S3: List of pathways (pages 105–167).
