# Supplementary material for: Knowledge Discovery in Databases of Proteomics by Systems Modeling in Translational Research on Pancreatic Cancer
Source: Proteomes. 2025 May 29;13(2):20. doi: 10.3390/proteomes13020020 (PMC12196815; doi:10.3390/proteomes13020020)
Supplement: Supplementary file 1 [file proteomes-13-00020-s001.zip › proteomes-3606848-supplementary.pdf]

proteomes

Knowledge Discovery in Databases of Proteomics by Systems Modeling in Translational Research on Pancreatic Cancer

Mathilde Resell[1], Elisabeth Pimpisa Graarud[1], Hanne-Line Rabben[1], Animesh Sharma[2], Lars Hagen[2], Linh Hoang[1], Nan T. Skogaker[1], Anne Aarvik[1], Magnus K. Svensson[1], Manoj Amrutkar[3], Caroline S. Verbeke[3,4], Surinder K. Batra[5], Gunnar Qvigstad[6], Timothy C. Wang[7], Anil Rustgi[7], Duan Chen[1], Chun-Mei Zhao[1,*]

1 Department of Clinical and Molecular Medicine, Norwegian University of Science and Technology, Trondheim, Norway
2 PROMEC - Proteomics and Modomics Experimental Core Facility at NTNU and the Central Norway Regional Health Authority, Trondheim, Norway
3 Department of Pathology, Oslo University Hospital, Oslo, Norway
4 Institute of Clinical Medicine, University of Oslo, Oslo, Norway
5 Department Biochemistry and Molecular Biology, University of Nebraska College of Medicine, Nebraska, USA
6 Department of Gastroenterology, St.Olav's Hospital, Trondheim, Norway; 7Divison of Digestive and Liver Diseases, Herbert Irving Comprehensive Cancer Center, Columbia University Irving Medical Center, New York, USA

*Correspondence: Chun-Mei Zhao (chun-mei.zhao@ntnu.no)

Supplementary information:

Supplementary Table 1: Bootstrapped model validations in terms of R2, MAE and MSE (page 2)

Supplementary Data 1: Common proteins (1975 matched proteins related to Fig. 3) (pages 3-56)

Supplementary Date 2: List of hub proteins (related to Fig. 4) (pages 57-104)

Supplementary Data 3: List of pathways (related to Fig.5 and Fig.7) (pages 105-167)

Citation: To be added by editorial staff during production.

Academic Editor: Firstname Lastname

Received: date
Revised: date
Accepted: date
Published: date

Copyright: © 2024 by the authors. Submitted for possible open access publication under the terms and conditions of the Creative Commons Attribution (CC BY) license (https://creativecommons.org/licenses/by/4.0/).

Supplementary Table 1: Bootstrapped model validations in terms of R^2, MAE and MSE

Model	R^2 mean	R^2 lower (CI)	R^2 upper (CI)
Random Forest	0.8207	0.7759	0.8599
Gradient Boosting	0.8191	0.7732	0.8591
CatBoost	0.8201	0.7745	0.8602
Gaussian Process	0.8102	0.7649	0.8505
Extra Trees	0.6394	0.5976	0.6767
Bayesian Ridge	0.0823	0.0724	0.0916
KNN	0.8102	0.7624	0.8498
Decision Tree	0.8208	0.7737	0.8580
ElasticNet	0.0808	0.0715	0.0908

Model	MAE mean	MAE lower (CI)	MAE upper (CI)
Random Forest	0.0241	0.0228	0.0254
Gradient Boosting	0.0243	0.0230	0.0257
CatBoost	0.0243	0.0230	0.0256
Gaussian Process	0.0252	0.0240	0.0266
Extra Trees	0.0326	0.0307	0.0345
Bayesian Ridge	0.0504	0.0473	0.0536
KNN	0.0248	0.0234	0.0262
Decision Tree	0.0240	0.0229	0.0254
ElasticNet	0.0501	0.0473	0.0532

Model	MSE mean	MSE lower (CI)	MSE upper (CI)
Random Forest	0.0014	0.0010	0.0019
Gradient Boosting	0.0014	0.0010	0.0020
CatBoost	0.0014	0.0010	0.0019
Gaussian Process	0.0015	0.0011	0.0020
Extra Trees	0.0029	0.0023	0.0035
Bayesian Ridge	0.0073	0.0060	0.0087
KNN	0.0015	0.0011	0.0020
Decision Tree	0.0014	0.0011	0.0019
ElasticNet	0.0073	0.0061	0.0085

Date 1: Common proteins

ID	Gene	Description	Location	Family	Drugs
O14964	HGS*	hepatocyte growth factor-regulated tyrosine kinase substrate	Cytoplasm	other	
P47897	QARS1	glutaminyl-tRNA synthetase 1	Cytoplasm	enzyme	
Q9C0E8-2	LNPK	lunapark, ER junction formation factor	Cytoplasm	other	
RPS11	RPS11	ribosomal protein S11	Cytoplasm	other	
RPS17	RPS17	ribosomal protein S17	Cytoplasm	other	
NDUFS6	NDUFS6	NADH:ubiquinone oxidoreductase subunit S6	Cytoplasm	enzyme	
GCSH	GCSH	glycine cleavage system protein H	Cytoplasm	enzyme	
ECHS1	ECHS1	enoyl-CoA hydratase, short chain 1	Cytoplasm	enzyme	
PFKP	PFKP	phosphofructokinase, platelet	Cytoplasm	kinase	
ATP5F1	ATP5PB	ATP synthase peripheral stalk-membrane subunit b	Cytoplasm	transporter	
DECR1	DECR1	2,4-dienoyl-CoA reductase 1	Cytoplasm	enzyme	
SLC25A3	SLC25A3	solute carrier family 25 member 3	Cytoplasm	transporter	
ESYT1	ESYT1	extended synaptotagmin 1	Cytoplasm	transporter	
LMAN1	LMAN1	lectin, mannose binding 1	Cytoplasm	other	
RAB1B	RAB1B	RAB1B, member RAS oncogene family	Cytoplasm	other	
GALNT3	GALNT3	polypeptide N-acetylgalactosaminyltransferase 3	Cytoplasm	enzyme	
RPL19	RPL19	ribosomal protein L19	Cytoplasm	other	
SOD2	SOD2	superoxide dismutase 2	Cytoplasm	enzyme	manganese
AGPAT1	AGPAT1	1-acylglycerol-3-phosphate O-acyltransferase 1	Cytoplasm	enzyme	
COPS7A	COPS7A	COP9 signalosome subunit 7A	Cytoplasm	other	
PROSC	PLPBP	pyridoxal phosphate binding protein	Cytoplasm	enzyme	
LONP1	LONP1	lon peptidase 1, mitochondrial	Cytoplasm	peptidase	
PTCD3	PTCD3	pentatricopeptide repeat domain 3	Cytoplasm	other	
RPL7A	RPL7A	ribosomal protein L7a	Cytoplasm	other	
UBA1	UBA1	ubiquitin like modifier activating enzyme 1	Cytoplasm	enzyme	MLN7243
ATXN10	ATXN10	ataxin 10	Cytoplasm	other	
ECH1	ECH1	enoyl-CoA hydratase 1	Cytoplasm	enzyme	
FAHD1	FAHD1	fumarylacetoacetate hydrolase domain containing 1	Cytoplasm	enzyme	
EPT1	SELENOI	selenoprotein I	Cytoplasm	enzyme	
TAOK3	TAOK3	TAO kinase 3	Cytoplasm	kinase	TAO3 inhibitor
KIAA1033	WASHC4	WASH complex subunit 4	Cytoplasm	other	
GRPEL1	GRPEL1	GrpE like 1, mitochondrial	Cytoplasm	other	
ROCK2	ROCK2	Rho associated coiled-coil containing protein kinase 2	Cytoplasm	kinase	ripasudil, GSK-269962A, CCT129524, belumosudil, H89, fasudil,
ANXA3	ANXA3	annexin A3	Cytoplasm	enzyme	
GTPBP1	GTPBP1	GTP binding protein 1	Cytoplasm	enzyme	
ALDOC	ALDOC	aldolase, fructose-bisphosphate C	Cytoplasm	enzyme	
ACBD3	ACBD3	acyl-CoA binding domain containing 3	Cytoplasm	other	
MFF	MFF	mitochondrial fission factor	Cytoplasm	other	
ABR	ABR	ABR activator of RhoGEF and GTPase	Cytoplasm	other	
UFM1	UFM1	ubiquitin fold modifier 1	Cytoplasm	other	
USP14	USP14	ubiquitin specific peptidase 14	Cytoplasm	peptidase	VLX1570

MAOA	MAOA	monoamine oxidase A	Cytoplasm	enzyme	fenfluramine/phentermine, moclobemide, methamphetamine, isocarboxazid, CX-1370, benzphetamine, phentermine/topiramate, N-(2-indanyl)glycinamide, iproniazid, phentermine, tranylcypromine, procainamide,
DSTN	DSTN	destrin, actin depolymerizing factor	Cytoplasm	other	
ALG2	ALG2	ALG2 alpha-1,3/1,6-mannosyltransferase	Cytoplasm	enzyme	
GRB2	GRB2	growth factor receptor bound protein 2	Cytoplasm	other	liposome-incorporated Grb2 antisense oligodeoxynucleotide
HSPD1	HSPD1	heat shock protein family D (Hsp60) member 1	Cytoplasm	enzyme	
RPS28	RPS28	ribosomal protein S28	Cytoplasm	other	
COQ9	COQ9	coenzyme Q9	Cytoplasm	other	
ADH5	ADH5	alcohol dehydrogenase 5 (class III), chi polypeptide	Cytoplasm	enzyme	N6022
PREP	PREP	prolyl endopeptidase	Cytoplasm	peptidase	Z 321
COPS4	COPS4	COP9 signalosome subunit 4	Cytoplasm	peptidase	
GCLM	GCLM	glutamate-cysteine ligase modifier subunit	Cytoplasm	enzyme	
PPM1A	PPM1A	protein phosphatase, Mg2+/Mn2+ dependent 1A	Cytoplasm	phosphatase	
NDUFS4	NDUFS4	NADH:ubiquinone oxidoreductase subunit S4	Cytoplasm	enzyme	
COG1	COG1	component of oligomeric golgi complex 1	Cytoplasm	transporter	
PSMD11	PSMD11	proteasome 26S subunit, non-ATPase 11	Cytoplasm	other	
SEC23IP	SEC23IP	SEC23 interacting protein	Cytoplasm	other	
UQCRC1	UQCRC1	ubiquinol-cytochrome c reductase core protein 1	Cytoplasm	enzyme	
SNX6	SNX6	sorting nexin 6	Cytoplasm	transporter	
EXOC2	EXOC2	exocyst complex component 2	Cytoplasm	transporter	
SAMM50	SAMM50	SAMM50 sorting and assembly machinery component	Cytoplasm	other	
MTX1	MTX1	metaxin 1	Cytoplasm	transporter	
NUCB1	NUCB1	nucleobindin 1	Cytoplasm	other	
CAPN1	CAPN1	calpain 1	Cytoplasm	peptidase	alicapistat, BLD-2660
MYO5C	MYO5C	myosin VC	Cytoplasm	other	
DOHH	DOHH	deoxyhypusine hydroxylase	Cytoplasm	enzyme	
COX7A2	COX7A2	cytochrome c oxidase subunit 7A2	Cytoplasm	enzyme	
SARS	SARS1	seryl-tRNA synthetase 1	Cytoplasm	enzyme	
VPS25	VPS25	vacuolar protein sorting 25 homolog	Cytoplasm	other	
UFL1	UFL1	UFM1 specific ligase 1	Cytoplasm	enzyme	
GLG1	GLG1	golgi glycoprotein 1	Cytoplasm	other	
L2HGDH	L2HGDH	L-2-hydroxyglutarate dehydrogenase	Cytoplasm	enzyme	
AGPS	AGPS	alkylglycerone phosphate synthase	Cytoplasm	enzyme	
PCCB	PCCB	propionyl-CoA carboxylase subunit beta	Cytoplasm	enzyme	
GSTP1	GSTP1	glutathione S-transferase pi 1	Cytoplasm	enzyme	zeaxanthin
HDHD2	HDHD2	haloacid dehalogenase like hydrolase domain containing 2	Cytoplasm	other	
GMFB	GMFB	glia maturation factor beta	Cytoplasm	growth factor	
ASAH1	ASAH1	N-acylsphingosine amidohydrolase 1	Cytoplasm	enzyme	
PSMD5	PSMD5	proteasome 26S subunit, non-ATPase 5	Cytoplasm	other	

RPL4	RPL4	ribosomal protein L4	Cytoplasm	enzyme	
RBPMS	RBPMS	RNA binding protein, mRNA processing factor	Cytoplasm	transcription regulator	
ATP6V1C1	ATP6V1C1	ATPase H+ transporting V1 subunit C1	Cytoplasm	transporter	
RPS21	RPS21	ribosomal protein S21	Cytoplasm	other	
ACO2	ACO2	aconitase 2	Cytoplasm	enzyme	
RTCB	RTCB	RNA 2',3'-cyclic phosphate and 5'-OH ligase	Cytoplasm	enzyme	
UBE2N	UBE2N	ubiquitin conjugating enzyme E2 N	Cytoplasm	enzyme	
GOT2	GOT2	glutamic-oxaloacetic transaminase 2	Cytoplasm	enzyme	
ACOT2	ACOT2	acyl-CoA thioesterase 2	Cytoplasm	enzyme	
CCT4	CCT4	chaperonin containing TCP1 subunit 4	Cytoplasm	other	
EXOC1	EXOC1	exocyst complex component 1	Cytoplasm	transporter	
CHMP6	CHMP6	charged multivesicular body protein 6	Cytoplasm	other	
ETFA	ETFA	electron transfer flavoprotein subunit alpha	Cytoplasm	transporter	
NDUFA13	NDUFA13	NADH:ubiquinone oxidoreductase subunit A13	Cytoplasm	enzyme	
SCAMP3	SCAMP3	secretory carrier membrane protein 3	Cytoplasm	transporter	
RDX	RDX	radixin	Cytoplasm	other	
PHB2	PHB2	prohibitin 2	Cytoplasm	transcription regulator	
DCTN1	DCTN1	dynactin subunit 1	Cytoplasm	other	
C1QBP	C1QBP	complement C1q binding protein	Cytoplasm	transcription regulator	
NDUFB8	NDUFB8	NADH:ubiquinone oxidoreductase subunit B8	Cytoplasm	enzyme	
DLST	DLST	dihydrolipoamide S-succinyltransferase	Cytoplasm	enzyme	
TCP1	TCP1	t-complex 1	Cytoplasm	other	
RER1	RER1	retention in endoplasmic reticulum sorting receptor 1	Cytoplasm	other	
RPN1	RPN1	ribophorin I	Cytoplasm	enzyme	
EIF3D	EIF3D	eukaryotic translation initiation factor 3 subunit D	Cytoplasm	translation regulator	
FAM120A	FAM120A	family with sequence similarity 120 member A	Cytoplasm	other	
HSPA2	HSPA2	heat shock protein family A (Hsp70) member 2	Cytoplasm	other	
LAMTOR3	LAMTOR3	late endosomal/lysosomal adaptor, MAPK and MTOR activator 3	Cytoplasm	other	
EIF2S1	EIF2S1	eukaryotic translation initiation factor 2 subunit alpha	Cytoplasm	translation regulator	
RAB5A	RAB5A	RAB5A, member RAS oncogene family	Cytoplasm	enzyme	
ACADVL	ACADVL	acyl-CoA dehydrogenase very long chain	Cytoplasm	enzyme	
ISCU	ISCU	iron-sulfur cluster assembly enzyme	Cytoplasm	other	
CECR5	HDHD5	haloacid dehalogenase like hydrolase domain containing 5	Cytoplasm	other	
PHGDH	PHGDH	phosphoglycerate dehydrogenase	Cytoplasm	enzyme	
TALDO1	TALDO1	transaldolase 1	Cytoplasm	enzyme	
NF1	NF1	neurofibromin 1	Cytoplasm	other	
EIF5A	EIF5A	eukaryotic translation initiation factor 5A	Cytoplasm	translation regulator	SNS01-T nanoparticles
SPG7	SPG7	SPG7 matrix AAA peptidase subunit, paraplegin	Cytoplasm	peptidase	
RMDN3	RMDN3	regulator of microtubule dynamics 3	Cytoplasm	other	
PPID	PPID	peptidylprolyl isomerase D	Cytoplasm	enzyme	
FIS1	FIS1	fission, mitochondrial 1	Cytoplasm	other	
TRIM56	TRIM56	tripartite motif containing 56	Cytoplasm	enzyme	
FAM129B	NIBAN2	niban apoptosis regulator 2	Cytoplasm	transcription regulator	
SAFB2	SAFB2	scaffold attachment factor B2	Cytoplasm	other	
ADD1	ADD1	adducin 1	Cytoplasm	other	
PSMD12	PSMD12	proteasome 26S subunit, non-ATPase 12	Cytoplasm	other	
ANP32A	ANP32A	acidic nuclear phosphoprotein 32 family member A	Cytoplasm	other	

ACLY	ACLY	ATP citrate lyase	Cytoplasm	enzyme	bempedoyl-CoA, bempedoic acid/ezetimibe,
SMS	SMS	spermine synthase	Cytoplasm	enzyme	(±)-2-hydroxyoleic
RRAGC	RRAGC	Ras related GTP binding C	Cytoplasm	enzyme	
GPX4	GPX4	glutathione peroxidase 4	Cytoplasm	enzyme	
CCAR2	CCAR2	cell cycle and apoptosis regulator 2	Cytoplasm	peptidase	
PHPT1	PHPT1	phosphohistidine phosphatase 1	Cytoplasm	phosphatase	
CDS2	CDS2	CDP-diacylglycerol synthase 2	Cytoplasm	enzyme	
TUFM	TUFM	Tu translation elongation factor, mitochondrial	Cytoplasm	translation regulator	
TOMM22	TOMM22	translocase of outer mitochondrial membrane 22	Cytoplasm	transporter	
RPLP2	RPLP2	ribosomal protein lateral stalk subunit P2	Cytoplasm	other	
RPL27	RPL27	ribosomal protein L27	Cytoplasm	other	
VCP	VCP	valosin containing protein	Cytoplasm	enzyme	CB-5083, CB-
RDH11	RDH11	retinol dehydrogenase 11	Cytoplasm	enzyme	
SH3GL1	SH3GL1	SH3 domain containing GRB2 like 1, endophilin A2	Cytoplasm	other	
EIF3H	EIF3H	eukaryotic translation initiation factor 3 subunit H	Cytoplasm	enzyme	
RALB	RALB	RAS like proto-oncogene B	Cytoplasm	enzyme	
GPD1L	GPD1L	glycerol-3-phosphate dehydrogenase 1 like	Cytoplasm	enzyme	
ABHD14B	ABHD14B	abhydrolase domain containing 14B	Cytoplasm	enzyme	
VPS37B	VPS37B	VPS37B subunit of ESCRT-I	Cytoplasm	other	
MRPL37	MRPL37	mitochondrial ribosomal protein L37	Cytoplasm	enzyme	
NDUFV2	NDUFV2	NADH:ubiquinone oxidoreductase core subunit V2	Cytoplasm	enzyme	
PSMA5	PSMA5	proteasome 20S subunit alpha 5	Cytoplasm	peptidase	
TRIP10	TRIP10	thyroid hormone receptor interactor 10	Cytoplasm	other	
SFXN3	SFXN3	sideroflexin 3	Cytoplasm	transporter	
LRRFIP1	LRRFIP1	LRR binding FLII interacting protein 1	Cytoplasm	transcription regulator	
GCLC	GCLC	glutamate-cysteine ligase catalytic subunit	Cytoplasm	enzyme	
MAN1A2	MAN1A2	mannosidase alpha class 1A member 2	Cytoplasm	enzyme	
SEC61A1	SEC61A1	SEC61 translocon subunit alpha 1	Cytoplasm	transporter	KZR-261
IKBKAP	ELP1	elongator acetyltransferase complex subunit 1	Cytoplasm	other	
BLVRB	BLVRB	biliverdin reductase B	Cytoplasm	enzyme	
STMN1	STMN1	stathmin 1	Cytoplasm	other	
EBP	EBP	EBP cholestenol delta-isomerase	Cytoplasm	enzyme	DSP-0390, SR 31747
SLC25A24	SLC25A24	solute carrier family 25 member 24	Cytoplasm	transporter	
DNAJB11	DNAJB11	DnaJ heat shock protein family (Hsp40) member B11	Cytoplasm	other	
ARMCX3	ARMCX3	armadillo repeat containing X-linked 3	Cytoplasm	other	
STK24	STK24	serine/threonine kinase 24	Cytoplasm	kinase	bosutinib, bosutinib/rituximab, bosutinib/imatinib
FH	FH	fumarate hydratase	Cytoplasm	enzyme	
ABCD3	ABCD3	ATP binding cassette subfamily D member 3	Cytoplasm	transporter	
PTPN23	PTPN23	protein tyrosine phosphatase non-receptor type 23	Cytoplasm	phosphatase	
GSR	GSR	glutathione-disulfide reductase	Cytoplasm	enzyme	carmustine/prednisone,
ATP6V1F	ATP6V1F	ATPase H+ transporting V1 subunit F	Cytoplasm	enzyme	
PDCD6	PDCD6	programmed cell death 6	Cytoplasm	other	
EIF2AK2	EIF2AK2	eukaryotic translation initiation factor 2 alpha kinase 2	Cytoplasm	kinase	etavopivat, mitapivat
LMO7	LMO7	LIM domain 7	Cytoplasm	enzyme	
MAN1A1	MAN1A1	mannosidase alpha class 1A member 1	Cytoplasm	enzyme	
EIF4E	EIF4E	eukaryotic translation initiation factor 4E	Cytoplasm	translation regulator	ISIS 183750

EXOC4	EXOC4	exocyst complex component 4	Cytoplasm	transporter	
ATP5A1	ATP5F1A	ATP synthase F1 subunit alpha	Cytoplasm	transporter	
ACADM	ACADM	acyl-CoA dehydrogenase medium chain	Cytoplasm	enzyme	
FMR1	FMR1	fragile X messenger ribonucleoprotein 1	Cytoplasm	translation regulator	
USP15	USP15	ubiquitin specific peptidase 15	Cytoplasm	peptidase	
NDUFB7	NDUFB7	NADH:ubiquinone oxidoreductase subunit B7	Cytoplasm	enzyme	
CNPY3	CNPY3	canopy FGF signaling regulator 3	Cytoplasm	other	
GOLGB1	GOLGB1	golgin B1	Cytoplasm	other	
PDLIM1	PDLIM1	PDZ and LIM domain 1	Cytoplasm	transcription regulator	
UQCR10	UQCR10	ubiquinol-cytochrome c reductase, complex III subunit X	Cytoplasm	enzyme	
RPS10	RPS10	ribosomal protein S10	Cytoplasm	other	
DPM1	DPM1	dolichyl-phosphate mannosyltransferase subunit 1, catalytic	Cytoplasm	enzyme	
PSMB7	PSMB7	proteasome 20S subunit beta 7	Cytoplasm	peptidase	
API5	API5	apoptosis inhibitor 5	Cytoplasm	other	
NAGLU	NAGLU	N-acetyl-alpha-glucosaminidase	Cytoplasm	enzyme	
HDHD3	HDHD3	haloacid dehalogenase like hydrolase domain containing 3	Cytoplasm	enzyme	
CAPNS1	CAPNS1	calpain small subunit 1	Cytoplasm	peptidase	
DYNC1LI1	DYNC1LI1	dynein cytoplasmic 1 light intermediate chain 1	Cytoplasm	other	
VPS4B	VPS4B	vacuolar protein sorting 4 homolog B	Cytoplasm	transporter	
SLC25A4	SLC25A4	solute carrier family 25 member 4	Cytoplasm	transporter	clodronic acid
APPL2	APPL2	adaptor protein, phosphotyrosine interacting with PH domain and leucine zipper 2	Cytoplasm	other	
MRPL46	MRPL46	mitochondrial ribosomal protein L46	Cytoplasm	other	
SNX5	SNX5	sorting nexin 5	Cytoplasm	transporter	
EEF1D	EEF1D	eukaryotic translation elongation factor 1 delta	Cytoplasm	translation regulator	
RAB6A	RAB6A	RAB6A, member RAS oncogene family	Cytoplasm	enzyme	
SOD1	SOD1	superoxide dismutase 1	Cytoplasm	enzyme	tofersen
CAPN2	CAPN2	calpain 2	Cytoplasm	peptidase	alicapistat, BLD-2660
TMX3	TMX3	thioredoxin related transmembrane protein 3	Cytoplasm	enzyme	
RMDN1	RMDN1	regulator of microtubule dynamics 1	Cytoplasm	other	
DBI	DBI	diazepam binding inhibitor, acyl-CoA binding protein	Cytoplasm	other	
ABCF1	ABCF1	ATP binding cassette subfamily F member 1	Cytoplasm	transporter	
AGL	AGL	amylo-alpha-1, 6-glucosidase, 4-alpha-glucanotransferase	Cytoplasm	enzyme	
EML4	EML4	EMAP like 4	Cytoplasm	other	
SGTA	SGTA	small glutamine rich tetratricopeptide repeat co-chaperone alpha	Cytoplasm	other	
PSMD6	PSMD6	proteasome 26S subunit, non-ATPase 6	Cytoplasm	enzyme	
TIMM23	TIMM23	translocase of inner mitochondrial membrane 23	Cytoplasm	transporter	
RPL28	RPL28	ribosomal protein L28	Cytoplasm	other	
RPL18A	RPL18A	ribosomal protein L18a	Cytoplasm	other	
HM13	HM13	histocompatibility minor 13	Cytoplasm	peptidase	
NUDC	NUDC	nuclear distribution C, dynein complex regulator	Cytoplasm	other	
PPP2R2A	PPP2R2A	protein phosphatase 2 regulatory subunit Balpha	Cytoplasm	phosphatase	
BCAT2	BCAT2	branched chain amino acid transaminase 2	Cytoplasm	enzyme	
BCKDHA	BCKDHA	branched chain keto acid dehydrogenase E1 subunit alpha	Cytoplasm	enzyme	
FARSA	FARSA	phenylalanyl-tRNA synthetase subunit alpha	Cytoplasm	enzyme	
UBE2H	UBE2H	ubiquitin conjugating enzyme E2 H	Cytoplasm	enzyme	
PPP1CB	PPP1CB	protein phosphatase 1 catalytic subunit beta	Cytoplasm	phosphatase	
ARF5	ARF5	ADP ribosylation factor 5	Cytoplasm	enzyme	

CCT3	CCT3	chaperonin containing TCP1 subunit 3	Cytoplasm	other	
MTHFD1L	MTHFD1L	methylenetetrahydrofolate dehydrogenase (NADP+ dependent) 1 like	Cytoplasm	enzyme	
PSMD10	PSMD10	proteasome 26S subunit, non-ATPase 10	Cytoplasm	transcription regulator	
RNPEP	RNPEP	arginyl aminopeptidase	Cytoplasm	peptidase	
IST1	IST1	IST1 factor associated with ESCRT-III	Cytoplasm	other	
STAU1	STAU1	staufen double-stranded RNA binding protein 1	Cytoplasm	transporter	
HADHB	HADHB	hydroxyacyl-CoA dehydrogenase trifunctional multienzyme complex subunit beta	Cytoplasm	enzyme	
METAP2	METAP2	methionyl aminopeptidase 2	Cytoplasm	peptidase	APL-1202, XMT-1107, O-(chloroacetylcarbamoyl)fumagillol, beloranib, APL-1501, PPI-2458, M8891, SDX-7320, nitroxoline,
NSFL1C	NSFL1C	NSFL1 cofactor	Cytoplasm	other	
RHOG	RHOG	ras homolog family member G	Cytoplasm	enzyme	
PDHX	PDHX	pyruvate dehydrogenase complex component X	Cytoplasm	enzyme	
CDC37	CDC37	cell division cycle 37, HSP90 cochaperone	Cytoplasm	other	
HIP1R	HIP1R	huntingtin interacting protein 1 related	Cytoplasm	other	
PTGES2	PTGES2	prostaglandin E synthase 2	Cytoplasm	transcription regulator	
LRRC1	LRRC1	leucine rich repeat containing 1	Cytoplasm	other	
TOMM70A	TOMM70	translocase of outer mitochondrial membrane 70	Cytoplasm	transporter	
FECH	FECH	ferrochelatase	Cytoplasm	enzyme	
TM9SF3	TM9SF3	transmembrane 9 superfamily member 3	Cytoplasm	transporter	
RNH1	RNH1	ribonuclease/angiogenin inhibitor 1	Cytoplasm	other	
RPL35A	RPL35A	ribosomal protein L35a	Cytoplasm	other	
DAK	TKFC	triokinase and FMN cyclase	Cytoplasm	kinase	
PDIA6	PDIA6	protein disulfide isomerase family A member 6	Cytoplasm	enzyme	
ACP1	ACP1	acid phosphatase 1	Cytoplasm	phosphatase	
EHD1	EHD1	EH domain containing 1	Cytoplasm	other	
CHCHD6	CHCHD6	coiled-coil-helix-coiled-coil-helix domain containing 6	Cytoplasm	other	
EIF2B1	EIF2B1	eukaryotic translation initiation factor 2B subunit alpha	Cytoplasm	translation regulator	
GSTZ1	GSTZ1	glutathione S-transferase zeta 1	Cytoplasm	enzyme	
TBC1D15	TBC1D15	TBC1 domain family member 15	Cytoplasm	other	
IMPA1	IMPA1	inositol monophosphatase 1	Cytoplasm	phosphatase	
ARPC5L	ARPC5L	actin related protein 2/3 complex subunit 5 like	Cytoplasm	other	
CSTB	CSTB	cystatin B	Cytoplasm	peptidase	
PTRH2	PTRH2	peptidyl-tRNA hydrolase 2	Cytoplasm	enzyme	
NDUFA12	NDUFA12	NADH:ubiquinone oxidoreductase subunit A12	Cytoplasm	enzyme	
SRM	SRM	spermidine synthase	Cytoplasm	enzyme	
HADHA	HADHA	hydroxyacyl-CoA dehydrogenase trifunctional multienzyme complex subunit alpha	Cytoplasm	enzyme	
MVB12A	MVB12A	multivesicular body subunit 12A	Cytoplasm	other	
TIMM50	TIMM50	translocase of inner mitochondrial membrane 50	Cytoplasm	phosphatase	
MIA3	MIA3	MIA SH3 domain ER export factor 3	Cytoplasm	other	
TOMM40	TOMM40	translocase of outer mitochondrial membrane 40	Cytoplasm	ion channel	
RPL23A	RPL23A	ribosomal protein L23a	Cytoplasm	other	
EIF3E	EIF3E	eukaryotic translation initiation factor 3 subunit E	Cytoplasm	translation regulator	
ADSS	ADSS2	adenylosuccinate synthase 2	Cytoplasm	enzyme	alanosine, adenylosuccinate synthetase inhibitor
RPL31	RPL31	ribosomal protein L31	Cytoplasm	other	

RAB10	RAB10	RAB10, member RAS oncogene family	Cytoplasm	enzyme	
PIGU	PIGU	phosphatidylinositol glycan anchor biosynthesis class U	Cytoplasm	enzyme	
RPN2	RPN2	ribophorin II	Cytoplasm	enzyme	
SDF2L1	SDF2L1	stromal cell derived factor 2 like 1	Cytoplasm	other	
HIBADH	HIBADH	3-hydroxyisobutyrate dehydrogenase	Cytoplasm	enzyme	
PSMA6	PSMA6	proteasome 20S subunit alpha 6	Cytoplasm	peptidase	
UPF2	UPF2	UPF2 regulator of nonsense mediated mRNA decay	Cytoplasm	other	
CPT1A	CPT1A	carnitine palmitoyltransferase 1A	Cytoplasm	enzyme	perhexiline
EIF3I	EIF3I	eukaryotic translation initiation factor 3 subunit I	Cytoplasm	translation regulator	
SUCLG2	SUCLG2	succinate-CoA ligase GDP-forming subunit beta	Cytoplasm	enzyme	
AMPD2	AMPD2	adenosine monophosphate deaminase 2	Cytoplasm	enzyme	
CYB5A	CYB5A	cytochrome b5 type A	Cytoplasm	enzyme	
LIN7C	LIN7C	lin-7 homolog C, crumbs cell polarity complex component	Cytoplasm	other	
TSFM	TSFM	Ts translation elongation factor, mitochondrial	Cytoplasm	translation regulator	
MAP1S	MAP1S	microtubule associated protein 1S	Cytoplasm	enzyme	
MTX2	MTX2	metaxin 2	Cytoplasm	transporter	
NDUFS3	NDUFS3	NADH:ubiquinone oxidoreductase core subunit S3	Cytoplasm	enzyme	
MYO1D	MYO1D	myosin ID	Cytoplasm	enzyme	
MAP2K3	MAP2K3	mitogen-activated protein kinase kinase 3	Cytoplasm	kinase	
FKBP11	FKBP11	FKBP prolyl isomerase 11	Cytoplasm	enzyme	
RPL38	RPL38	ribosomal protein L38	Cytoplasm	other	
NARS	NARS1	asparaginyl-tRNA synthetase 1	Cytoplasm	enzyme	
RPS16	RPS16	ribosomal protein S16	Cytoplasm	other	
AP2A2	AP2A2	adaptor related protein complex 2 subunit alpha 2	Cytoplasm	transporter	
PEBP1	PEBP1	phosphatidylethanolamine binding protein 1	Cytoplasm	other	
AAK1	AAK1	AP2 associated kinase 1	Cytoplasm	kinase	LP-935509, SM1-71
RPL14	RPL14	ribosomal protein L14	Cytoplasm	other	
TMOD3	TMOD3	tropomodulin 3	Cytoplasm	other	
EEA1	EEA1	early endosome antigen 1	Cytoplasm	other	
CCT6A	CCT6A	chaperonin containing TCP1 subunit 6A	Cytoplasm	other	
TBC1D8B	TBC1D8B	TBC1 domain family member 8B	Cytoplasm	other	
FKBP2	FKBP2	FKBP prolyl isomerase 2	Cytoplasm	enzyme	
RCN2	RCN2	reticulocalbin 2	Cytoplasm	other	
ARFGAP1	ARFGAP1	ADP ribosylation factor GTPase activating protein 1	Cytoplasm	enzyme	
SHMT2	SHMT2	serine hydroxymethyltransferase 2	Cytoplasm	enzyme	
NDUFA5	NDUFA5	NADH:ubiquinone oxidoreductase subunit A5	Cytoplasm	enzyme	
AP1S1	AP1S1	adaptor related protein complex 1 subunit sigma 1	Cytoplasm	transporter	
SDHA	SDHA	succinate dehydrogenase complex flavoprotein subunit A	Cytoplasm	enzyme	
UQCRC2	UQCRC2	ubiquinol-cytochrome c reductase core protein 2	Cytoplasm	enzyme	
DRG2	DRG2	developmentally regulated GTP binding protein 2	Cytoplasm	enzyme	
EIF2D	EIF2D	eukaryotic translation initiation factor 2D	Cytoplasm	transporter	
COG7	COG7	component of oligomeric golgi complex 7	Cytoplasm	transporter	
DARS2	DARS2	aspartyl-tRNA synthetase 2, mitochondrial	Cytoplasm	enzyme	
BAIAP2L1	BAIAP2L1	BAR/IMD domain containing adaptor protein 2 like 1	Cytoplasm	other	
DBNL	DBNL	drebrin like	Cytoplasm	other	
ERGIC1	ERGIC1	endoplasmic reticulum-golgi intermediate compartment 1	Cytoplasm	other	
ATOX1	ATOX1	antioxidant 1 copper chaperone	Cytoplasm	transporter	
MAP4	MAP4	microtubule associated protein 4	Cytoplasm	other	
BID	BID	BH3 interacting domain death agonist	Cytoplasm	other	

COX5A	COX5A	cytochrome c oxidase subunit 5A	Cytoplasm	enzyme	
EIF6	EIF6	eukaryotic translation initiation factor 6	Cytoplasm	translation regulator	
AIFM1	AIFM1	apoptosis inducing factor mitochondria associated 1	Cytoplasm	enzyme	
NUFIP2	NUFIP2	nuclear FMR1 interacting protein 2	Cytoplasm	other	
ADD3	ADD3	adducin 3	Cytoplasm	other	
ATP6V0D1	ATP6V0D1	ATPase H+ transporting V0 subunit d1	Cytoplasm	transporter	
TUBGCP3	TUBGCP3	tubulin gamma complex component 3	Cytoplasm	other	
AP1G1	AP1G1	adaptor related protein complex 1 subunit gamma 1	Cytoplasm	other	
ECHDC1	ECHDC1	ethylmalonyl-CoA decarboxylase 1	Cytoplasm	enzyme	
GNAI3	GNAI3	G protein subunit alpha i3	Cytoplasm	enzyme	
TPD52L2	TPD52L2	TPD52 like 2	Cytoplasm	other	
SDHB	SDHB	succinate dehydrogenase complex iron sulfur subunit B	Cytoplasm	enzyme	
DERA	DERA	deoxyribose-phosphate aldolase	Cytoplasm	enzyme	
TPP2	TPP2	tripeptidyl peptidase 2	Cytoplasm	peptidase	
DDOST	DDOST	dolichyl-diphosphooligosaccharide--protein glycosyltransferase non-catalytic subunit	Cytoplasm	enzyme	
ARHGAP17	ARHGAP17	Rho GTPase activating protein 17	Cytoplasm	other	
AKR1B10	AKR1B10	aldo-keto reductase family 1 member B10	Cytoplasm	enzyme	
PSMA1	PSMA1	proteasome 20S subunit alpha 1	Cytoplasm	peptidase	
PDLIM7	PDLIM7	PDZ and LIM domain 7	Cytoplasm	other	
ARHGEF12	ARHGEF12	Rho guanine nucleotide exchange factor 12	Cytoplasm	other	
LYPLA1	LYPLA1	lysophospholipase 1	Cytoplasm	enzyme	
RPS23	RPS23	ribosomal protein S23	Cytoplasm	translation regulator	
SCYL1	SCYL1	SCY1 like pseudokinase 1	Cytoplasm	kinase	
LNPEP	LNPEP	leucyl and cystinyl aminopeptidase	Cytoplasm	peptidase	
YARS2	YARS2	tyrosyl-tRNA synthetase 2	Cytoplasm	enzyme	
NAPG	NAPG	NSF attachment protein gamma	Cytoplasm	transporter	
HMOX2	HMOX2	heme oxygenase 2	Cytoplasm	enzyme	tin mesoporphyrin
PLP2	PLP2	proteolipid protein 2	Cytoplasm	transporter	
TAGLN2	TAGLN2	transgelin 2	Cytoplasm	other	
NEDD4	NEDD4	NEDD4 E3 ubiquitin protein ligase	Cytoplasm	enzyme	
MDH1	MDH1	malate dehydrogenase 1	Cytoplasm	enzyme	
PRKAR2A	PRKAR2A	protein kinase cAMP-dependent type II regulatory subunit alpha	Cytoplasm	kinase	
PEX14	PEX14	peroxisomal biogenesis factor 14	Cytoplasm	transcription regulator	
PAFAH1B1	PAFAH1B1	platelet activating factor acetylhydrolase 1b regulatory subunit 1	Cytoplasm	enzyme	
MYO6	MYO6	myosin VI	Cytoplasm	other	
SLC25A1	SLC25A1	solute carrier family 25 member 1	Cytoplasm	transporter	
SLC25A5	SLC25A5	solute carrier family 25 member 5	Cytoplasm	transporter	clodronic acid
MYO1C	MYO1C	myosin IC	Cytoplasm	enzyme	
PRKCI	PRKCI	protein kinase C iota	Cytoplasm	kinase	CRT0066854, myristoylated PKC-zeta pseudosubstrate peptide inhibitor, PKC-iota pseudosubstrate peptide
VWA8	VWA8	von Willebrand factor A domain containing 8	Cytoplasm	enzyme	
DDAH1	DDAH1	dimethylarginine dimethylaminohydrolase 1	Cytoplasm	enzyme	
ALDH4A1	ALDH4A1	aldehyde dehydrogenase 4 family member A1	Cytoplasm	enzyme	
COPA	COPA	COPI coat complex subunit alpha	Cytoplasm	transporter	
CKB	CKB	creatine kinase B	Cytoplasm	kinase	
RTN3	RTN3	reticulon 3	Cytoplasm	other	
PARVA	PARVA	parvin alpha	Cytoplasm	other	
TRMT112	TRMT112	tRNA methyltransferase activator subunit 11-2	Cytoplasm	enzyme	

GOLPH3	GOLPH3	golgi phosphoprotein 3	Cytoplasm	other	
CCT2	CCT2	chaperonin containing TCP1 subunit 2	Cytoplasm	kinase	
ARF4	ARF4	ADP ribosylation factor 4	Cytoplasm	enzyme	
HSD17B11	HSD17B11	hydroxysteroid 17-beta dehydrogenase 11	Cytoplasm	enzyme	
SH3GLB1	SH3GLB1	SH3 domain containing GRB2 like, endophilin B1	Cytoplasm	enzyme	
LARS	LARS1	leucyl-tRNA synthetase 1	Cytoplasm	enzyme	
MRPL28	MRPL28	mitochondrial ribosomal protein L28	Cytoplasm	other	
RAB14	RAB14	RAB14, member RAS oncogene family	Cytoplasm	enzyme	
LETM1	LETM1	leucine zipper and EF-hand containing transmembrane protein 1	Cytoplasm	transporter	
NIPSNAP1	NIPSNAP1	nipsnap homolog 1	Cytoplasm	enzyme	
B4GALT1	B4GALT1	beta-1,4-galactosyltransferase 1	Cytoplasm	enzyme	
BCAP31	BCAP31	B cell receptor associated protein 31	Cytoplasm	transporter	
DYNC1LI2	DYNC1LI2	dynein cytoplasmic 1 light intermediate chain 2	Cytoplasm	other	
GDI1	GDI1	GDP dissociation inhibitor 1	Cytoplasm	other	
PDXK	PDXK	pyridoxal kinase	Cytoplasm	kinase	
PTGES3	PTGES3	prostaglandin E synthase 3	Cytoplasm	enzyme	
CS	CS	citrate synthase	Cytoplasm	enzyme	
PRDX1	PRDX1	peroxiredoxin 1	Cytoplasm	enzyme	
CBR4	CBR4	carbonyl reductase 4	Cytoplasm	enzyme	
RPS13	RPS13	ribosomal protein S13	Cytoplasm	other	
LDAH	LDAH	lipid droplet associated hydrolase	Cytoplasm	enzyme	
PRKAA1	PRKAA1	protein kinase AMP-activated catalytic subunit alpha 1	Cytoplasm	kinase	phenformin
ATP2C1	ATP2C1	ATPase secretory pathway Ca2+ transporting 1	Cytoplasm	transporter	sevoflurane, isoflurane, desflurane, enflurane, Ca2+
AHCY	AHCY	adenosylhomocysteinase	Cytoplasm	enzyme	9-(2',3'-dihydroxycyclopent-4'-enyl)adenine, 3-deazaneplanocin, neplanocin A, 3-deazaaristeromycin, cyanovirin-N, 6'-C-methylneplano
GRSF1	GRSF1	G-rich RNA sequence binding factor 1	Cytoplasm	other	
ASPH	ASPH	aspartate beta-hydroxylase	Cytoplasm	enzyme	
OXCT1	OXCT1	3-oxoacid CoA-transferase 1	Cytoplasm	enzyme	
SH3BGRL	SH3BGRL	SH3 domain binding glutamate rich protein like	Cytoplasm	other	
ARPC5	ARPC5	actin related protein 2/3 complex subunit 5	Cytoplasm	other	
VPS51	VPS51	VPS51 subunit of GARP complex	Cytoplasm	other	faricimab
PITPNA	PITPNA	phosphatidylinositol transfer protein alpha	Cytoplasm	transporter	
NDUFS2	NDUFS2	NADH:ubiquinone oxidoreductase core subunit S2	Cytoplasm	enzyme	
STUB1	STUB1	STIP1 homology and U-box containing protein 1	Cytoplasm	enzyme	
SLC25A22	SLC25A22	solute carrier family 25 member 22	Cytoplasm	transporter	
MRPS7	MRPS7	mitochondrial ribosomal protein S7	Cytoplasm	other	

MAPK14	MAPK14	mitogen-activated protein kinase 14	Cytoplasm	kinase	pamapimod, UM101, RO-3201195, CGH2466, ARRY-371797, p38 MAP kinase inhibitor, AMG 2372, Sb202190, PH-797804, talmapimod, p38 MAP kinase inhibitor IV, Org 48762-
DYNLL2	DYNLL2	dynein light chain LC8-type 2	Cytoplasm	other	
AP2A1	AP2A1	adaptor related protein complex 2 subunit alpha 1	Cytoplasm	transporter	
VBP1	VBP1	VHL binding protein 1	Cytoplasm	other	
SLC25A13	SLC25A13	solute carrier family 25 member 13	Cytoplasm	transporter	
AKR7A2	AKR7A2	aldo-keto reductase family 7 member A2	Cytoplasm	enzyme	
MAP2K2	MAP2K2	mitogen-activated protein kinase kinase 2	Cytoplasm	kinase	U0126, pimasertib, binimetinib/vemurafenib, dabrafenib/trametinib, AS703988, dabrafenib/trametinib/vemurafenib, trametinib/vemurafenib, dabrafenib/pembrolizumab/trametinib, SM1-71, docetaxel/selumetinib, TAK 733, binimetinib/cetuximab/encorafenib, binimetinib/encorafenib/panitumumab, mirdametinib, PD184352, cetuximab/dabrafenib/trametinib, binimetinib, PD318088,
UQCRB	UQCRB	ubiquinol-cytochrome c reductase binding protein	Cytoplasm	enzyme	
SPR	SPR	sepiapterin reductase	Cytoplasm	enzyme	
ABCE1	ABCE1	ATP binding cassette subfamily E member 1	Cytoplasm	transporter	
LDHA	LDHA	lactate dehydrogenase A	Cytoplasm	enzyme	nedosiran
UBA3	UBA3	ubiquitin like modifier activating enzyme 3	Cytoplasm	enzyme	
AGK	AGK	acylglycerol kinase	Cytoplasm	kinase	SM1-71
NDUFB5	NDUFB5	NADH:ubiquinone oxidoreductase subunit B5	Cytoplasm	enzyme	
OXR1	OXR1	oxidation resistance 1	Cytoplasm	enzyme	
HK1	HK1	hexokinase 1	Cytoplasm	kinase	
SLIRP	SLIRP	SRA stem-loop interacting RNA binding protein	Cytoplasm	other	
CDC42	CDC42	cell division cycle 42	Cytoplasm	enzyme	
UACA	UACA	uveal autoantigen with coiled-coil domains and ankyrin repeats	Cytoplasm	other	
LRPPRC	LRPPRC	leucine rich pentatricopeptide repeat containing	Cytoplasm	other	
HEXB	HEXB	hexosaminidase subunit beta	Cytoplasm	enzyme	
SNX2	SNX2	sorting nexin 2	Cytoplasm	transporter	
COL4A3BP	CERT1	ceramide transporter 1	Cytoplasm	kinase	
PYCR1	PYCR1	pyrroline-5-carboxylate reductase 1	Cytoplasm	enzyme	
GRHPR	GRHPR	glyoxylate and hydroxypyruvate reductase	Cytoplasm	enzyme	GSK1278863A

MYO18A	MYO18A	myosin XVIIIA	Cytoplasm	other	
SUMF2	SUMF2	sulfatase modifying factor 2	Cytoplasm	other	
TMED5	TMED5	transmembrane p24 trafficking protein 5	Cytoplasm	other	
PPIB	PPIB	peptidylprolyl isomerase B	Cytoplasm	enzyme	
GOLGA5	GOLGA5	golgin A5	Cytoplasm	kinase	
ALDH7A1	ALDH7A1	aldehyde dehydrogenase 7 family member A1	Cytoplasm	enzyme	
VDAC3	VDAC3	voltage dependent anion channel 3	Cytoplasm	ion channel	
EIF3F	EIF3F	eukaryotic translation initiation factor 3 subunit F	Cytoplasm	translation regulator	
TMED2	TMED2	transmembrane p24 trafficking protein 2	Cytoplasm	transporter	
ACSL5	ACSL5	acyl-CoA synthetase long chain family member 5	Cytoplasm	enzyme	
PFKM	PFKM	phosphofructokinase, muscle	Cytoplasm	kinase	
GART	GART	phosphoribosylglycinamide formyltransferase, phosphoribosylglycinamide synthetase, phosphoribosylaminoimidazole synthetase	Cytoplasm	enzyme	pemetrexed, bevacizumab/erlotinib/pemetrexed, pelitrexol, bevacizumab/pemetrexed, pembrolizumab/pemetrexed, pemetrexed polyglutamate, gemcitabine/pemetrexed, bevacizumab/paclitaxel/pemet
THOP1	THOP1	thimet oligopeptidase 1	Cytoplasm	peptidase	
ARMC10	ARMC10	armadillo repeat containing 10	Cytoplasm	other	
PPT1	PPT1	palmitoyl-protein thioesterase 1	Cytoplasm	enzyme	ezurpimtrostat
BCL2L13	BCL2L13	BCL2 like 13	Cytoplasm	other	
GOSR1	GOSR1	golgi SNAP receptor complex member 1	Cytoplasm	transporter	
RPL30	RPL30	ribosomal protein L30	Cytoplasm	other	
PLOD3	PLOD3	procollagen-lysine,2-oxoglutarate 5-dioxygenase 3	Cytoplasm	enzyme	
DRG1	DRG1	developmentally regulated GTP binding protein 1	Cytoplasm	enzyme	
RQCD1	CNOT9	CCR4-NOT transcription complex subunit 9	Cytoplasm	transcription regulator	
SPCS1	SPCS1	signal peptidase complex subunit 1	Cytoplasm	peptidase	
SRP19	SRP19	signal recognition particle 19	Cytoplasm	other	
DYNLRB1	DYNLRB1	dynein light chain roadblock-type 1	Cytoplasm	other	
ATL3	ATL3	atlastin GTPase 3	Cytoplasm	other	
LASP1	LASP1	LIM and SH3 protein 1	Cytoplasm	transporter	
PLAA	PLAA	phospholipase A2 activating protein	Cytoplasm	other	
SEH1L	SEH1L	SEH1 like nucleoporin	Cytoplasm	transporter	
SHMT1	SHMT1	serine hydroxymethyltransferase 1	Cytoplasm	enzyme	mimosine
CAMK2G	CAMK2G	calcium/calmodulin dependent protein kinase II gamma	Cytoplasm	kinase	
KIF3B	KIF3B	kinesin family member 3B	Cytoplasm	transporter	
MCCC2	MCCC2	methylcrotonyl-CoA carboxylase subunit 2	Cytoplasm	enzyme	
CPT2	CPT2	carnitine palmitoyltransferase 2	Cytoplasm	enzyme	perhexiline
SEC31A	SEC31A	SEC31 homolog A, COPII coat complex component	Cytoplasm	other	
IVD	IVD	isovaleryl-CoA dehydrogenase	Cytoplasm	enzyme	
PSME1	PSME1	proteasome activator subunit 1	Cytoplasm	other	
NSDHL	NSDHL	NAD(P) dependent steroid dehydrogenase-like	Cytoplasm	enzyme	
TOR1B	TOR1B	torsin family 1 member B	Cytoplasm	enzyme	
ARHGEF40	ARHGEF40	Rho guanine nucleotide exchange factor 40	Cytoplasm	other	
FXR1	FXR1	FMR1 autosomal homolog 1	Cytoplasm	other	
RAB11B	RAB11B	RAB11B, member RAS oncogene family	Cytoplasm	enzyme	
YWHAB	YWHAB	tyrosine 3-monooxygenase/tryptophan 5-monooxygenase activation protein beta	Cytoplasm	other	

RNF213	RNF213	ring finger protein 213	Cytoplasm	enzyme	
PDIA5	PDIA5	protein disulfide isomerase family A member 5	Cytoplasm	enzyme	
SUCLG1	SUCLG1	succinate-CoA ligase GDP/ADP-forming subunit alpha	Cytoplasm	enzyme	
PKN2	PKN2	protein kinase N2	Cytoplasm	kinase	fasudil
PSMA2	PSMA2	proteasome 20S subunit alpha 2	Cytoplasm	peptidase	
DTD1	DTD1	D-aminoacyl-tRNA deacylase 1	Cytoplasm	enzyme	
EML2	EML2	EMAP like 2	Cytoplasm	other	
RTN4	RTN4	reticulon 4	Cytoplasm	other	
NAPA	NAPA	NSF attachment protein alpha	Cytoplasm	transporter	
RPL26	RPL26	ribosomal protein L26	Cytoplasm	other	
NDUFB10	NDUFB10	NADH:ubiquinone oxidoreductase subunit B10	Cytoplasm	enzyme	
NDUFV3	NDUFV3	NADH:ubiquinone oxidoreductase subunit V3	Cytoplasm	enzyme	
PGAM5	PGAM5	PGAM family member 5, mitochondrial serine/threonine protein phosphatase	Cytoplasm	enzyme	
EPPK1	EPPK1	epiplakin 1	Cytoplasm	other	
EEF2	EEF2	eukaryotic translation elongation factor 2	Cytoplasm	translation regulator	tagraxofusp, MDNA55
PRDX3	PRDX3	peroxiredoxin 3	Cytoplasm	enzyme	
LMAN2	LMAN2	lectin, mannose binding 2	Cytoplasm	transporter	
CHCHD3	CHCHD3	coiled-coil-helix-coiled-coil-helix domain containing 3	Cytoplasm	transcription regulator	
SCYL2	SCYL2	SCY1 like pseudokinase 2	Cytoplasm	other	
OSTC	OSTC	oligosaccharyltransferase complex non-catalytic subunit	Cytoplasm	enzyme	
VPS37C	VPS37C	VPS37C subunit of ESCRT-I	Cytoplasm	other	
ATP6V1B2	ATP6V1B2	ATPase H+ transporting V1 subunit B2	Cytoplasm	transporter	gallium nitrate
CAPZA1	CAPZA1	capping actin protein of muscle Z-line subunit alpha 1	Cytoplasm	other	
ERP29	ERP29	endoplasmic reticulum protein 29	Cytoplasm	transporter	
GLRX3	GLRX3	glutaredoxin 3	Cytoplasm	enzyme	
TOMM6	TOMM6	translocase of outer mitochondrial membrane 6	Cytoplasm	other	
AHSA1	AHSA1	activator of HSP90 ATPase activity 1	Cytoplasm	other	
LGMN	LGMN	legumain	Cytoplasm	peptidase	
HACD3	HACD3	3-hydroxyacyl-CoA dehydratase 3	Cytoplasm	enzyme	
SNX27	SNX27	sorting nexin 27	Cytoplasm	other	
NUBPL	NUBPL	NUBP iron-sulfur cluster assembly factor, mitochondrial	Cytoplasm	other	
HIBCH	HIBCH	3-hydroxyisobutyryl-CoA hydrolase	Cytoplasm	enzyme	
EIF5	EIF5	eukaryotic translation initiation factor 5	Cytoplasm	translation regulator	
NLN	NLN	neurolysin	Cytoplasm	peptidase	
TPP1	TPP1	tripeptidyl peptidase 1	Cytoplasm	peptidase	
RPL5	RPL5	ribosomal protein L5	Cytoplasm	other	
SEC24C	SEC24C	SEC24 homolog C, COPII coat complex component	Cytoplasm	transporter	
HSPA9	HSPA9	heat shock protein family A (Hsp70) member 9	Cytoplasm	other	SHetA2
SURF1	SURF1	SURF1 cytochrome c oxidase assembly factor	Cytoplasm	enzyme	
TUBGCP2	TUBGCP2	tubulin gamma complex component 2	Cytoplasm	peptidase	
RPS15A	RPS15A	ribosomal protein S15a	Cytoplasm	other	
TNPO3	TNPO3	transportin 3	Cytoplasm	other	
CYB5R1	CYB5R1	cytochrome b5 reductase 1	Cytoplasm	enzyme	
ATP6V1H	ATP6V1H	ATPase H+ transporting V1 subunit H	Cytoplasm	transporter	
IQGAP1	IQGAP1	IQ motif containing GTPase activating protein 1	Cytoplasm	other	
RPS12	RPS12	ribosomal protein S12	Cytoplasm	other	neomycin
PABPC4	PABPC4	poly(A) binding protein cytoplasmic 4	Cytoplasm	translation regulator	
VPS28	VPS28	VPS28 subunit of ESCRT-I	Cytoplasm	transporter	
AP3M1	AP3M1	adaptor related protein complex 3 subunit mu 1	Cytoplasm	transporter	
AKAP9	AKAP9	A-kinase anchoring protein 9	Cytoplasm	other	
TSG101	TSG101	tumor susceptibility 101	Cytoplasm	transcription regulator	

HPRT1	HPRT1	hypoxanthine phosphoribosyltransferase 1	Cytoplasm	enzyme	cytarabine/daunorubicin/thioguanine, 6-mercaptopurine/methotrexate, 6-mercaptopurine, 6-mercaptopurine/vincristine, hydroxyurea/6-mercaptopurine, imatinib/thioguanine, daunorubicin/etoposide/6-mercaptopurine/mitoxantrone/prednisolone/vindesine, L-asparaginase/cyclophosphamide/cytarabine/daunorubicin/6-mercaptopurine/prednisone/vincristine, 6-mercaptopurine/prednisone/thioguanine, cyclophospham
BAG2	BAG2	BAG cochaperone 2	Cytoplasm	other	
PSMA7	PSMA7	proteasome 20S subunit alpha 7	Cytoplasm	peptidase	
HMGCL	HMGCL	3-hydroxy-3-methylglutaryl-CoA lyase	Cytoplasm	enzyme	
MSI2	MSI2	musashi RNA binding protein 2	Cytoplasm	other	
PLEC	PLEC	plectin	Cytoplasm	other	ZB131
RPL18	RPL18	ribosomal protein L18	Cytoplasm	other	
PSMB5	PSMB5	proteasome 20S subunit beta 5	Cytoplasm	peptidase	bortezomib/thalidomide, bortezomib/dexamethasone/rituximab, dexamethasone/ixazomib/rituximab, ixazomib, bortezomib/cladribine/rituximab, bortezomib/dexamethasone/pomalidomide, bortezomib/doxorubicin, bortezomib/dexamethasone/thalidomide, bortezomib/paclitaxel, carfilzomib, bortezomib/sorafenib, bortezomib/fulvestrant, bortezomib/rituximab, bortezomib/lenalidomide, carfilzomib/dex
UBE4B	UBE4B	ubiquitination factor E4B	Cytoplasm	enzyme	
DBN1	DBN1	drebrin 1	Cytoplasm	other	
QTRTD1	QTRT2	queuine tRNA-ribosyltransferase accessory subunit 2	Cytoplasm	enzyme	
RPS19	RPS19	ribosomal protein S19	Cytoplasm	other	
LRRC59	LRRC59	leucine rich repeat containing 59	Cytoplasm	other	
EIF3L	EIF3L	eukaryotic translation initiation factor 3 subunit L	Cytoplasm	translation regulator	
GDI2	GDI2	GDP dissociation inhibitor 2	Cytoplasm	other	

ARL3	ARL3	ADP ribosylation factor like GTPase 3	Cytoplasm	enzyme	
BPHL	BPHL	biphenyl hydrolase like	Cytoplasm	enzyme	
S100A10	S100A10	S100 calcium binding protein A10	Cytoplasm	other	
COX5B	COX5B	cytochrome c oxidase subunit 5B	Cytoplasm	enzyme	
TMX1	TMX1	thioredoxin related transmembrane protein 1	Cytoplasm	enzyme	
PRDX2	PRDX2	peroxiredoxin 2	Cytoplasm	enzyme	
EFTUD1	EFL1	elongation factor like GTPase 1	Cytoplasm	translation regulator	
PPAT	PPAT	phosphoribosyl pyrophosphate amidotransferase	Cytoplasm	enzyme	L-asparaginase/cyclophosphamide/cytarabine/daunorubicin/6-mercaptopurine/prednisone/vincristine, 6-mercaptopurine/prednisone/thioguanine, cytarabine/daunorubicin/thioguanine, cyclophosphamide/cytarabine/6-mercaptopurine, cytarabine/thioguanine, 6-mercaptopurine, thioguanine, 6-mercaptopurine/vincristine, amidophosphoribosyltransferase inhibitor, 6-mercaptopurine/prednisone, hydroxyurea/6-
HSPH1	HSPH1	heat shock protein family H (Hsp110) member 1	Cytoplasm	other	
ACTB	ACTB	actin beta	Cytoplasm	other	
UBA2	UBA2	ubiquitin like modifier activating enzyme 2	Cytoplasm	enzyme	
KIF13B	KIF13B	kinesin family member 13B	Cytoplasm	other	
UBQLN1	UBQLN1	ubiquilin 1	Cytoplasm	other	
PSMD1	PSMD1	proteasome 26S subunit, non-ATPase 1	Cytoplasm	other	bortezomib/cladribine/rituximab, bortezomib/dexamethasone/pomalidomide, bortezomib/doxorubicin, bortezomib/dexamethasone/thalidomide, bortezomib/paclitaxel, bortezomib/sorafenib, bortezomib/fulvestrant, bortezomib/rituximab, bortezomib/lenalidomide, bortezomib/dexamethasone/lenalidomide, bortezomib/dexamethasone/doxorubicin, bortezomib/dexamethasone, bortezomib/prednisone, bortezomib/vor

TIMM44	TIMM44	translocase of inner mitochondrial membrane 44	Cytoplasm	transporter	
ILVBL	ILVBL	ilvB acetolactate synthase like	Cytoplasm	enzyme	
COPB1	COPB1	COPI coat complex subunit beta 1	Cytoplasm	transporter	
FAM162A	FAM162A	family with sequence similarity 162 member A	Cytoplasm	other	
HERC4	HERC4	HECT and RLD domain containing E3 ubiquitin protein ligase 4	Cytoplasm	enzyme	
PAICS	PAICS	phosphoribosylaminoimidazole carboxylase and phosphoribosylaminoimidazolesuccinocarboxamide synthase	Cytoplasm	enzyme	
NIT2	NIT2	nitrilase family member 2	Cytoplasm	enzyme	
SNX1	SNX1	sorting nexin 1	Cytoplasm	transporter	
GPD2	GPD2	glycerol-3-phosphate dehydrogenase 2	Cytoplasm	enzyme	
TOR1AIP2	TOR1AIP2	torsin 1A interacting protein 2	Cytoplasm	other	
CANX	CANX	calnexin	Cytoplasm	other	
TIMM13	TIMM13	translocase of inner mitochondrial membrane 13	Cytoplasm	transporter	
EIF4A2	EIF4A2	eukaryotic translation initiation factor 4A2	Cytoplasm	translation regulator	
PABPC1	PABPC1	poly(A) binding protein cytoplasmic 1	Cytoplasm	translation regulator	
HSD17B12	HSD17B12	hydroxysteroid 17-beta dehydrogenase 12	Cytoplasm	enzyme	
CTPS2	CTPS2	CTP synthase 2	Cytoplasm	enzyme	
NDUFA9	NDUFA9	NADH:ubiquinone oxidoreductase subunit A9	Cytoplasm	enzyme	
SCRIB	SCRIB	scribble planar cell polarity protein	Cytoplasm	other	
SLC25A12	SLC25A12	solute carrier family 25 member 12	Cytoplasm	transporter	
EIF3K	EIF3K	eukaryotic translation initiation factor 3 subunit K	Cytoplasm	translation regulator	
EIF2S2	EIF2S2	eukaryotic translation initiation factor 2 subunit beta	Cytoplasm	translation regulator	
UCHL5	UCHL5	ubiquitin C-terminal hydrolase L5	Cytoplasm	peptidase	VLX1570
MAP2K1	MAP2K1	mitogen-activated protein kinase kinase 1	Cytoplasm	kinase	binimetinib/vemurafenib, ARRY-424704, dabrafenib/trametinib, dabrafenib/pembrolizumab/trametinib, TAK 733, binimetinib/encorafenib/panitumumab, E 6201, refametinib, PD184352, binimetinib, trametinib, RO4927350, cobimetinib, U0126, pimasertib, AS703988, dabrafenib/trametinib/vemurafenib, trametinib/vemurafenib, SM1-71, docetaxel/selumetinib, binimetinib/cetuximab/encora
ST13	ST13	ST13 Hsp70 interacting protein	Cytoplasm	other	
MARS	MARS1	methionyl-tRNA synthetase 1	Cytoplasm	enzyme	
CCDC22	CCDC22	coiled-coil domain containing 22	Cytoplasm	other	
RARS	RARS1	arginyl-tRNA synthetase 1	Cytoplasm	enzyme	
PGLS	PGLS	6-phosphogluconolactonase	Cytoplasm	enzyme	
SAE1	SAE1	SUMO1 activating enzyme subunit 1	Cytoplasm	enzyme	
COX4I1	COX4I1	cytochrome c oxidase subunit 4I1	Cytoplasm	enzyme	
YKT6	YKT6	YKT6 v-SNARE homolog	Cytoplasm	enzyme	
CTSH	CTSH	cathepsin H	Cytoplasm	peptidase	

CTSZ	CTSZ	cathepsin Z	Cytoplasm	peptidase	
DLAT	DLAT	dihydrolipoamide S-acetyltransferase	Cytoplasm	enzyme	
TBCD	TBCD	tubulin folding cofactor D	Cytoplasm	other	
DCTN2	DCTN2	dynactin subunit 2	Cytoplasm	other	
WNK1	WNK1	WNK lysine deficient protein kinase 1	Cytoplasm	kinase	WNK463
AHNAK2	AHNAK2	AHNAK nucleoprotein 2	Cytoplasm	other	
OLA1	OLA1	Obg like ATPase 1	Cytoplasm	enzyme	
PPA1	PPA1	inorganic pyrophosphatase 1	Cytoplasm	enzyme	
SSBP1	SSBP1	single stranded DNA binding protein 1	Cytoplasm	other	
PDIA4	PDIA4	protein disulfide isomerase family A member 4	Cytoplasm	enzyme	
SERBP1	SERBP1	SERPINE1 mRNA binding protein 1	Cytoplasm	translation regulator	
PDHB	PDHB	pyruvate dehydrogenase E1 subunit beta	Cytoplasm	enzyme	
NAGK	NAGK	N-acetylglucosamine kinase	Cytoplasm	kinase	
VTA1	VTA1	vesicle trafficking 1	Cytoplasm	other	
CLPP	CLPP	caseinolytic mitochondrial matrix peptidase proteolytic subunit	Cytoplasm	peptidase	ONC206
TXNDC17	TXNDC17	thioredoxin domain containing 17	Cytoplasm	enzyme	
DDX3X	DDX3X	DEAD-box helicase 3 X-linked	Cytoplasm	enzyme	
CRKL	CRKL	CRK like proto-oncogene, adaptor protein	Cytoplasm	kinase	
PGD	PGD	phosphogluconate dehydrogenase	Cytoplasm	enzyme	
CUTA	CUTA	cutA divalent cation tolerance homolog	Cytoplasm	other	
GOLGA4	GOLGA4	golgin A4	Cytoplasm	other	
IDH3B	IDH3B	isocitrate dehydrogenase (NAD(+)) 3 non-catalytic subunit beta	Cytoplasm	enzyme	
SELENBP1	SELENBP1	selenium binding protein 1	Cytoplasm	enzyme	
EIF3G	EIF3G	eukaryotic translation initiation factor 3 subunit G	Cytoplasm	translation regulator	
TMED4	TMED4	transmembrane p24 trafficking protein 4	Cytoplasm	transporter	
ALDH9A1	ALDH9A1	aldehyde dehydrogenase 9 family member A1	Cytoplasm	enzyme	
PITPNB	PITPNB	phosphatidylinositol transfer protein beta	Cytoplasm	transporter	
CARKD	NAXD	NAD(P)HX dehydratase	Cytoplasm	enzyme	
AARS	AARS1	alanyl-tRNA synthetase 1	Cytoplasm	enzyme	
TRAP1	TRAP1	TNF receptor associated protein 1	Cytoplasm	enzyme	gamitrinib-TPP-OH
SAR1B	SAR1B	secretion associated Ras related GTPase 1B	Cytoplasm	enzyme	
PAK2	PAK2	p21 (RAC1) activated kinase 2	Cytoplasm	kinase	FRAX-597, FRAX486, FRAX355, FRAX120, FRAX-1036
EEF1G	EEF1G	eukaryotic translation elongation factor 1 gamma	Cytoplasm	translation regulator	
GARS	GARS1	glycyl-tRNA synthetase 1	Cytoplasm	enzyme	
PFDN2	PFDN2	prefoldin subunit 2	Cytoplasm	other	
NDUFB9	NDUFB9	NADH:ubiquinone oxidoreductase subunit B9	Cytoplasm	enzyme	
RAB7A	RAB7A	RAB7A, member RAS oncogene family	Cytoplasm	enzyme	
NADK2	NADK2	NAD kinase 2, mitochondrial	Cytoplasm	kinase	
ITPR3	ITPR3	inositol 1,4,5-trisphosphate receptor type 3	Cytoplasm	ion channel	
CBR1	CBR1	carbonyl reductase 1	Cytoplasm	enzyme	
ACTN4	ACTN4	actinin alpha 4	Cytoplasm	transcription regulator	
RAB2A	RAB2A	RAB2A, member RAS oncogene family	Cytoplasm	enzyme	
DLG1	DLG1	discs large MAGUK scaffold protein 1	Cytoplasm	other	
VPS33A	VPS33A	VPS33A core subunit of CORVET and HOPS complexes	Cytoplasm	transporter	
PDHA1	PDHA1	pyruvate dehydrogenase E1 subunit alpha 1	Cytoplasm	enzyme	
MMGT1	MMGT1	membrane magnesium transporter 1	Cytoplasm	transporter	
TKT	TKT	transketolase	Cytoplasm	enzyme	

HSPB1	HSPB1	heat shock protein family B (small) member 1	Cytoplasm	other	
PMPCA	PMPCA	peptidase, mitochondrial processing subunit alpha	Cytoplasm	peptidase	
DDT	DDT	D-dopachrome tautomerase	Cytoplasm	enzyme	
HSD17B10	HSD17B10	hydroxysteroid 17-beta dehydrogenase 10	Cytoplasm	enzyme	
PRKRA	PRKRA	protein activator of interferon induced protein kinase EIF2AK2	Cytoplasm	other	
USP47	USP47	ubiquitin specific peptidase 47	Cytoplasm	peptidase	
NDUFS1	NDUFS1	NADH:ubiquinone oxidoreductase core subunit S1	Cytoplasm	enzyme	
NDUFAB1	NDUFAB1	NADH:ubiquinone oxidoreductase subunit AB1	Cytoplasm	enzyme	
COX6C	COX6C	cytochrome c oxidase subunit 6C	Cytoplasm	enzyme	
GOT1	GOT1	glutamic-oxaloacetic transaminase 1	Cytoplasm	enzyme	
ATP5D	ATP5F1D	ATP synthase F1 subunit delta	Cytoplasm	transporter	
RPS5	RPS5	ribosomal protein S5	Cytoplasm	other	
PCYT2	PCYT2	phosphate cytidylyltransferase 2, ethanolamine	Cytoplasm	enzyme	
RHOT1	RHOT1	ras homolog family member T1	Cytoplasm	enzyme	
EPS8	EPS8	epidermal growth factor receptor pathway substrate 8	Cytoplasm	other	
DARS	DARS1	aspartyl-tRNA synthetase 1	Cytoplasm	enzyme	
SHC1	SHC1	SHC adaptor protein 1	Cytoplasm	other	
DAD1	DAD1	defender against cell death 1	Cytoplasm	other	
HOOK3	HOOK3	hook microtubule tethering protein 3	Cytoplasm	other	
CAND1	CAND1	cullin associated and neddylation dissociated 1	Cytoplasm	transcription regulator	
CLCC1	CLCC1	chloride channel CLIC like 1	Cytoplasm	ion channel	
ACSL3	ACSL3	acyl-CoA synthetase long chain family member 3	Cytoplasm	enzyme	
RPL35	RPL35	ribosomal protein L35	Cytoplasm	other	
IMPDH2	IMPDH2	inosine monophosphate dehydrogenase 2	Cytoplasm	enzyme	mycophenolic acid, thioguanine, mycophenolate mofetil, imatinib/thioguanine, VX-944, pegintron/ribavirin, mycophenolate mofetil/prednisone, methylprednisolone/mycophenolate mofetil, interferon alfacon-1/ribavirin, 6-mercaptopurine/prednisone/thioguanine, cytarabine/daunorubicin/thioguanine, cytarabine/thio
OGDH	OGDH	oxoglutarate dehydrogenase	Cytoplasm	enzyme	
PDIA3	PDIA3	protein disulfide isomerase family A member 3	Cytoplasm	peptidase	
GSS	GSS	glutathione synthetase	Cytoplasm	enzyme	N-acetyl-L-cysteine
PPP3CA	PPP3CA	protein phosphatase 3 catalytic subunit alpha	Cytoplasm	phosphatase	voclosporin, tacrolimus, prednisone/tacrolimus, methylprednisolone/tacrolimus, pimecrolimus, cyclosporin A/methotrexate, cyclosporin A
ALDH3A2	ALDH3A2	aldehyde dehydrogenase 3 family member A2	Cytoplasm	enzyme	
GOLGA3	GOLGA3	golgin A3	Cytoplasm	transporter	
SERPINB9	SERPINB9	serpin family B member 9	Cytoplasm	other	
DHRS7	DHRS7	dehydrogenase/reductase 7	Cytoplasm	enzyme	

LAMTOR2	LAMTOR2	late endosomal/lysosomal adaptor, MAPK and MTOR activator 2	Cytoplasm	other	
ALDH18A1	ALDH18A1	aldehyde dehydrogenase 18 family member A1	Cytoplasm	kinase	
TPI1	TPI1	triosephosphate isomerase 1	Cytoplasm	enzyme	
ALDH2	ALDH2	aldehyde dehydrogenase 2 family member	Cytoplasm	enzyme	disulfiram/gemcitabine, disulfiram, chlorpropamid
QDPR	QDPR	quinoid dihydropteridine reductase	Cytoplasm	enzyme	
NDUFA8	NDUFA8	NADH:ubiquinone oxidoreductase subunit A8	Cytoplasm	enzyme	
FABP5	FABP5	fatty acid binding protein 5	Cytoplasm	transporter	
HYOU1	HYOU1	hypoxia up-regulated 1	Cytoplasm	other	
YWHAQ	YWHAQ	tyrosine 3-monooxygenase/tryptophan 5-monooxygenase activation protein theta	Cytoplasm	other	
MCU	MCU	mitochondrial calcium uniporter	Cytoplasm	ion channel	
RPLP1	RPLP1	ribosomal protein lateral stalk subunit P1	Cytoplasm	other	
SCP2	SCP2	sterol carrier protein 2	Cytoplasm	transporter	
TRAM1	TRAM1	translocation associated membrane protein 1	Cytoplasm	other	
PSMD3	PSMD3	proteasome 26S subunit, non-ATPase 3	Cytoplasm	other	
RAB35	RAB35	RAB35, member RAS oncogene family	Cytoplasm	enzyme	
ERLIN2	ERLIN2	ER lipid raft associated 2	Cytoplasm	other	
PCK2	PCK2	phosphoenolpyruvate carboxykinase 2, mitochondrial	Cytoplasm	kinase	
CELF1	CELF1	CUGBP Elav-like family member 1	Cytoplasm	translation regulator	
CCT8	CCT8	chaperonin containing TCP1 subunit 8	Cytoplasm	other	
EIF3M	EIF3M	eukaryotic translation initiation factor 3 subunit M	Cytoplasm	translation regulator	
RAB5B	RAB5B	RAB5B, member RAS oncogene family	Cytoplasm	enzyme	
MAPRE1	MAPRE1	microtubule associated protein RP/EB family member 1	Cytoplasm	other	
TRIP12	TRIP12	thyroid hormone receptor interactor 12	Cytoplasm	enzyme	
USP19	USP19	ubiquitin specific peptidase 19	Cytoplasm	peptidase	
ERO1L	ERO1A	endoplasmic reticulum oxidoreductase 1 alpha	Cytoplasm	enzyme	
DYNC1H1	DYNC1H1	dynein cytoplasmic 1 heavy chain 1	Cytoplasm	peptidase	
RPL32	RPL32	ribosomal protein L32	Cytoplasm	other	
KIF2A	KIF2A	kinesin family member 2A	Cytoplasm	other	
HSPA8	HSPA8	heat shock protein family A (Hsp70) member 8	Cytoplasm	enzyme	SHetA2
NGDN	NGDN	neuroguidin	Cytoplasm	other	
PICALM	PICALM	phosphatidylinositol binding clathrin assembly protein	Cytoplasm	other	
ARPC3	ARPC3	actin related protein 2/3 complex subunit 3	Cytoplasm	other	
TMED9	TMED9	transmembrane p24 trafficking protein 9	Cytoplasm	transporter	
CARS	CARS1	cysteinyl-tRNA synthetase 1	Cytoplasm	enzyme	
MGST1	MGST1	microsomal glutathione S-transferase 1	Cytoplasm	enzyme	
PPP1R12A	PPP1R12A	protein phosphatase 1 regulatory subunit 12A	Cytoplasm	phosphatase	
YWHAZ	YWHAZ	tyrosine 3-monooxygenase/tryptophan 5-monooxygenase activation protein zeta	Cytoplasm	enzyme	
GAA	GAA	alpha glucosidase	Cytoplasm	enzyme	miglitol, acarbose, acarbose/orlist
ARHGEF16	ARHGEF16	Rho guanine nucleotide exchange factor 16	Cytoplasm	other	
ACSF2	ACSF2	acyl-CoA synthetase family member 2	Cytoplasm	enzyme	
OPA1	OPA1	OPA1 mitochondrial dynamin like GTPase	Cytoplasm	enzyme	
RPL10	RPL10	ribosomal protein L10	Cytoplasm	translation regulator	

PFKL	PFKL	phosphofructokinase, liver type	Cytoplasm	kinase	
PPL	PPL	periplakin	Cytoplasm	other	
MOGS	MOGS	mannosyl-oligosaccharide glucosidase	Cytoplasm	enzyme	
CCDC6	CCDC6	coiled-coil domain containing 6	Cytoplasm	other	
MTHFD1	MTHFD1	methylenetetrahydrofolate dehydrogenase, cyclohydrolase and formyltetrahydrofolate synthetase 1	Cytoplasm	enzyme	
TPT1	TPT1	tumor protein, translationally-controlled 1	Cytoplasm	other	
SEC63	SEC63	SEC63 homolog, protein translocation regulator	Cytoplasm	transporter	
YWHAH	YWHAH	tyrosine 3-monooxygenase/tryptophan 5-monooxygenase activation protein eta	Cytoplasm	transcription regulator	
PAFAH1B3	PAFAH1B3	platelet activating factor acetylhydrolase 1b catalytic subunit 3	Cytoplasm	enzyme	
ALDH6A1	ALDH6A1	aldehyde dehydrogenase 6 family member A1	Cytoplasm	enzyme	
BCKDK	BCKDK	branched chain keto acid dehydrogenase kinase	Cytoplasm	kinase	BT2F, BT2
CTSB	CTSB	cathepsin B	Cytoplasm	peptidase	
COG4	COG4	component of oligomeric golgi complex 4	Cytoplasm	transporter	
GANAB	GANAB	glucosidase II alpha subunit	Cytoplasm	enzyme	miglitol
DNAJC11	DNAJC11	DnaJ heat shock protein family (Hsp40) member C11	Cytoplasm	other	
PGAM1	PGAM1	phosphoglycerate mutase 1	Cytoplasm	phosphatase	
SEC13	SEC13	SEC13 homolog, nuclear pore and COPII coat complex component	Cytoplasm	transporter	
HAGH	HAGH	hydroxyacylglutathione hydrolase	Cytoplasm	enzyme	
CNDP2	CNDP2	carnosine dipeptidase 2	Cytoplasm	peptidase	
PSMD8	PSMD8	proteasome 26S subunit, non-ATPase 8	Cytoplasm	other	
LRBA	LRBA	LPS responsive beige-like anchor protein	Cytoplasm	other	
MEMO1	MEMO1	mediator of cell motility 1	Cytoplasm	other	
DPP7	DPP7	dipeptidyl peptidase 7	Cytoplasm	peptidase	
SPAG9	SPAG9	sperm associated antigen 9	Cytoplasm	other	
CORO1C	CORO1C	coronin 1C	Cytoplasm	other	
ATG7	ATG7	autophagy related 7	Cytoplasm	enzyme	
PLIN3	PLIN3	perilipin 3	Cytoplasm	other	
EPS15	EPS15	epidermal growth factor receptor pathway substrate 15	Cytoplasm	other	
FDX1	FDX1	ferredoxin 1	Cytoplasm	transporter	
EIF2B2	EIF2B2	eukaryotic translation initiation factor 2B subunit beta	Cytoplasm	translation regulator	
GNS	GNS	glucosamine (N-acetyl)-6-sulfatase	Cytoplasm	enzyme	
ARFGEF2	ARFGEF2	ADP ribosylation factor guanine nucleotide exchange factor 2	Cytoplasm	other	
GALNT2	GALNT2	polypeptide N-acetylgalactosaminyltransferase 2	Cytoplasm	enzyme	
STK3	STK3	serine/threonine kinase 3	Cytoplasm	kinase	XMU-MP-1
BAG3	BAG3	BAG cochaperone 3	Cytoplasm	other	
RIC8A	RIC8A	RIC8 guanine nucleotide exchange factor A	Cytoplasm	other	

PSMD2	PSMD2	proteasome 26S subunit ubiquitin receptor, non-ATPase 2	Cytoplasm	other	bortezomib/cladribine/rituximab, bortezomib/dexamethasone/pomalidomide, bortezomib/doxorubicin, bortezomib/dexamethasone/thalidomide, bortezomib/paclitaxel, bortezomib/sorafenib, bortezomib/fulvestrant, bortezomib/rituximab, bortezomib/lenalidomide, bortezomib/dexamethasone/lenalidomide, bortezomib/dexamethasone/doxorubicin, bortezomib/dexamethasone, bortezomib/prednisone, bortezomib/vor
RRAS	RRAS	RAS related	Cytoplasm	enzyme	
ZFPL1	ZFPL1	zinc finger protein like 1	Cytoplasm	other	
SURF4	SURF4	surfeit 4	Cytoplasm	other	
NACA	NACA	nascent polypeptide associated complex subunit alpha	Cytoplasm	transcription regulator	
GLOD4	GLOD4	glyoxalase domain containing 4	Cytoplasm	enzyme	
SCCPDH	SCCPDH	saccharopine dehydrogenase (putative)	Cytoplasm	other	
GIGYF2	GIGYF2	GRB10 interacting GYF protein 2	Cytoplasm	other	
RBM3	RBM3	RNA binding motif protein 3	Cytoplasm	other	
RPL17	RPL17	ribosomal protein L17	Cytoplasm	other	
SCFD1	SCFD1	sec1 family domain containing 1	Cytoplasm	transporter	
ACOT9	ACOT9	acyl-CoA thioesterase 9	Cytoplasm	enzyme	
UBE2K	UBE2K	ubiquitin conjugating enzyme E2 K	Cytoplasm	transcription regulator	
MDH2	MDH2	malate dehydrogenase 2	Cytoplasm	enzyme	
CRAT	CRAT	carnitine O-acetyltransferase	Cytoplasm	enzyme	
POR	POR	cytochrome p450 oxidoreductase	Cytoplasm	enzyme	carboplatin/doxorubicin, nitazoxanide, cisplatin/doxorubicin, cisplatin/doxorubicin/ifosfamide, daunorubicin/tretinoin, cisplatin/doxorubicin/methotrexate, daunorubicin, doxorubicin/streptozocin, dacarbazine/doxorubicin, doxorubicin/ifosfamide, cisplatin/doxorubicin/ifosfamide/methotrexate, dacarbazine/doxorubicin/ifosfamide, doxorubicin, doxorubicin/tre
USMG5	ATP5MK	ATP synthase membrane subunit k	Cytoplasm	other	
VPS53	VPS53	VPS53 subunit of GARP complex	Cytoplasm	other	

RUFY1	RUFY1	RUN and FYVE domain containing 1	Cytoplasm	other	
TXNDC12	TXNDC12	thioredoxin domain containing 12	Cytoplasm	enzyme	
ATP6V1E1	ATP6V1E1	ATPase H+ transporting V1 subunit E1	Cytoplasm	transporter	
SAR1A	SAR1A	secretion associated Ras related GTPase 1A	Cytoplasm	enzyme	
ACAA2	ACAA2	acetyl-CoA acyltransferase 2	Cytoplasm	enzyme	
PIGK	PIGK	phosphatidylinositol glycan anchor biosynthesis class K	Cytoplasm	peptidase	
PLS3	PLS3	plastin 3	Cytoplasm	other	
RPS24	RPS24	ribosomal protein S24	Cytoplasm	other	
RPL24	RPL24	ribosomal protein L24	Cytoplasm	other	
YES1	YES1	YES proto-oncogene 1, Src family tyrosine kinase	Cytoplasm	kinase	blinatumomab/dasatinib, dasatinib/hydrocortisone, SC-204303, PD162531, dasatinib/dexamethasone/vincristine, CH6953755, SM1-71, dasatinib/filgrastim, dasatinib/dexamethasone, JNJ-26483327, cytarabine/dasatinib/dexamethasone/methotrexate, corticosteroid/dasatinib, dasatinib/rituximab, cytarabine/dasatinib/filgrastim/fludarabine phosphate, dasatinib/6-mercaptopurine/methotrexate,
RCC1	RCC1	regulator of chromosome condensation 1	Cytoplasm	other	
BROX	BROX	BRO1 domain and CAAX motif containing	Cytoplasm	other	
METAP1	METAP1	methionyl aminopeptidase 1	Cytoplasm	peptidase	
SSR4	SSR4	signal sequence receptor subunit 4	Cytoplasm	other	
PPP1CA	PPP1CA	protein phosphatase 1 catalytic subunit alpha	Cytoplasm	phosphatase	
TRIP11	TRIP11	thyroid hormone receptor interactor 11	Cytoplasm	transcription regulator	
FKBP8	FKBP8	FKBP prolyl isomerase 8	Cytoplasm	other	
CCT7	CCT7	chaperonin containing TCP1 subunit 7	Cytoplasm	other	
MYO1E	MYO1E	myosin IE	Cytoplasm	enzyme	
UCHL3	UCHL3	ubiquitin C-terminal hydrolase L3	Cytoplasm	peptidase	
VPS29	VPS29	VPS29 retromer complex component	Cytoplasm	transporter	
CARHSP1	CARHSP1	calcium regulated heat stable protein 1	Cytoplasm	transcription regulator	
HDDC2	HDDC2	HD domain containing 2	Cytoplasm	other	
COPB2	COPB2	COPI coat complex subunit beta 2	Cytoplasm	transporter	
VIL1	VIL1	villin 1	Cytoplasm	other	
GGH	GGH	gamma-glutamyl hydrolase	Cytoplasm	peptidase	
MCCC1	MCCC1	methylcrotonyl-CoA carboxylase subunit 1	Cytoplasm	enzyme	
VPS35	VPS35	VPS35 retromer complex component	Cytoplasm	transporter	
VAC14	VAC14	VAC14 component of PIKFYVE complex	Cytoplasm	other	
PSMD9	PSMD9	proteasome 26S subunit, non-ATPase 9	Cytoplasm	transcription regulator	
GFPT1	GFPT1	glutamine--fructose-6-phosphate transaminase 1	Cytoplasm	enzyme	
ME2	ME2	malic enzyme 2	Cytoplasm	enzyme	

GLO1	GLO1	glyoxalase I	Cytoplasm	enzyme	
PPP2R4	PTPA	protein phosphatase 2 phosphatase activator	Cytoplasm	phosphatase	
TMED3	TMED3	transmembrane p24 trafficking protein 3	Cytoplasm	transporter	
PPIL3	PPIL3	peptidylprolyl isomerase like 3	Cytoplasm	enzyme	
RPLP0	RPLP0	ribosomal protein lateral stalk subunit P0	Cytoplasm	other	
CCS	CCS	copper chaperone for superoxide dismutase	Cytoplasm	enzyme	
YWHAG	YWHAG	tyrosine 3-monooxygenase/tryptophan 5-monooxygenase activation protein gamma	Cytoplasm	other	
EIF5B	EIF5B	eukaryotic translation initiation factor 5B	Cytoplasm	translation regulator	
ASL	ASL	argininosuccinate lyase	Cytoplasm	enzyme	
TFAM	TFAM	transcription factor A, mitochondrial	Cytoplasm	transcription regulator	
ACOT13	ACOT13	acyl-CoA thioesterase 13	Cytoplasm	enzyme	
RPS6	RPS6	ribosomal protein S6	Cytoplasm	other	
SFXN1	SFXN1	sideroflexin 1	Cytoplasm	transporter	
CYP2S1	CYP2S1	cytochrome P450 family 2 subfamily S member 1	Cytoplasm	enzyme	
TBCE	TBCE	tubulin folding cofactor E	Cytoplasm	other	
MAPK1	MAPK1	mitogen-activated protein kinase 1	Cytoplasm	kinase	pexmetinib, ulixertinib, HE3286, ASN007, tyrphostin AG 127, rineterkib, CAY10561, ASTX029, (5Z)-3-(2-aminoethyl)-5-[[4-(2-methylpropoxy)phenyl]methylidene]-1,3-thiazolidine-2,4-dione, tyrphostin AG 1288, HH2710, KO-947, ZSET 1446, SM1-71, MAP kinase1 inhibitor, temuterkib, ATG-017, Vx-11e, binimetinib, FR 180204, (5Z)-3-(2-aminoethyl)-5-[(4-phenylmethoxyphenyl)methylidene]-1,3-thiazolidine-2,4-
G3BP2	G3BP2	G3BP stress granule assembly factor 2	Cytoplasm	enzyme	
PYGB	PYGB	glycogen phosphorylase B	Cytoplasm	enzyme	
NHLRC2	NHLRC2	NHL repeat containing 2	Cytoplasm	enzyme	
NAE1	NAE1	NEDD8 activating enzyme E1 subunit 1	Cytoplasm	enzyme	
COPE	COPE	COPI coat complex subunit epsilon	Cytoplasm	transporter	
EIF3A	EIF3A	eukaryotic translation initiation factor 3 subunit A	Cytoplasm	translation regulator	
BLVRA	BLVRA	biliverdin reductase A	Cytoplasm	enzyme	
SEC24B	SEC24B	SEC24 homolog B, COPII coat complex component	Cytoplasm	transporter	
ARHGAP1	ARHGAP1	Rho GTPase activating protein 1	Cytoplasm	other	
SNX9	SNX9	sorting nexin 9	Cytoplasm	transporter	
NDUFV1	NDUFV1	NADH:ubiquinone oxidoreductase core subunit V1	Cytoplasm	enzyme	
RAB3GAP2	RAB3GAP2	RAB3 GTPase activating non-catalytic protein subunit 2	Cytoplasm	enzyme	
NAAA	NAAA	N-acylethanolamine acid amidase	Cytoplasm	enzyme	
MUT	MMUT	methylmalonyl-CoA mutase	Cytoplasm	enzyme	hydroxocobalamin, cyanocobalami

TAX1BP3	TAX1BP3	Tax1 binding protein 3	Cytoplasm	transcription regulator	
ALDH16A1	ALDH16A1	aldehyde dehydrogenase 16 family member A1	Cytoplasm	enzyme	
RPL34	RPL34	ribosomal protein L34	Cytoplasm	other	
MARK2	MARK2	microtubule affinity regulating kinase 2	Cytoplasm	kinase	SM1-71
CTSE	CTSE	cathepsin E	Cytoplasm	peptidase	
ARL6IP5	ARL6IP5	ADP ribosylation factor like GTPase 6 interacting protein 5	Cytoplasm	other	
COPG2	COPG2	COPI coat complex subunit gamma 2	Cytoplasm	transporter	
IARS	IARS1	isoleucyl-tRNA synthetase 1	Cytoplasm	enzyme	
AP1B1	AP1B1	adaptor related protein complex 1 subunit beta 1	Cytoplasm	other	
DYNLL1	DYNLL1	dynein light chain LC8-type 1	Cytoplasm	other	
ARFIP1	ARFIP1	ADP ribosylation factor interacting protein 1	Cytoplasm	other	
SLC25A11	SLC25A11	solute carrier family 25 member 11	Cytoplasm	transporter	
SRP54	SRP54	signal recognition particle 54	Cytoplasm	enzyme	
GOLGA2	GOLGA2	golgin A2	Cytoplasm	other	
TUBA1C	TUBA1C	tubulin alpha 1c	Cytoplasm	other	docetaxel/gemcitabine/vincristine, bevacizumab/paclitaxel/topotecan, colchicine/probenecid, cyclophosphamide/epirubicin/5-fluorouracil/paclitaxel, bevacizumab/docetaxel/prednisone/thalidomide/zoledronic acid, docetaxel/zoledronic acid, gemcitabine/paclitaxel, L-asparaginase/daunorubicin/dexamethasone/vincristine, prednisone/vincristine, docetaxel/5-fluorouracil, vinflunine, L-asparaginase/d[illegible]
AKR1B1	AKR1B1	aldo-keto reductase family 1 member B	Cytoplasm	enzyme	sorbinil
MRPS31	MRPS31	mitochondrial ribosomal protein S31	Cytoplasm	other	
STIP1	STIP1	stress induced phosphoprotein 1	Cytoplasm	other	lavendustin C
PSMB4	PSMB4	proteasome 20S subunit beta 4	Cytoplasm	peptidase	
XPNPEP3	XPNPEP3	X-prolyl aminopeptidase 3	Cytoplasm	peptidase	
MTDH	MTDH	metadherin	Cytoplasm	transcription regulator	
HSP90AB1	HSP90AB1	heat shock protein 90 alpha family class B member 1	Cytoplasm	enzyme	alvespimycin, retaspimycin, pimitespib, cisplatin
HSPE1	HSPE1	heat shock protein family E (Hsp10) member 1	Cytoplasm	enzyme	
PPP1R9B	PPP1R9B	protein phosphatase 1 regulatory subunit 9B	Cytoplasm	enzyme	
GSPT1	GSPT1	G1 to S phase transition 1	Cytoplasm	translation regulator	
ARPC1B	ARPC1B	actin related protein 2/3 complex subunit 1B	Cytoplasm	other	
PMPCB	PMPCB	peptidase, mitochondrial processing subunit beta	Cytoplasm	peptidase	
COPS3	COPS3	COP9 signalosome subunit 3	Cytoplasm	other	
VARS	VARS1	valyl-tRNA synthetase 1	Cytoplasm	enzyme	
MRPS16	MRPS16	mitochondrial ribosomal protein S16	Cytoplasm	other	

CLPX	CLPX	caseinolytic mitochondrial matrix peptidase chaperone subunit X	Cytoplasm	enzyme	
MRPS36	MRPS36	mitochondrial ribosomal protein S36	Cytoplasm	other	
BZW2	BZW2	basic leucine zipper and W2 domains 2	Cytoplasm	translation regulator	
PSMD4	PSMD4	proteasome 26S subunit ubiquitin receptor, non-ATPase 4	Cytoplasm	other	
TST	TST	thiosulfate sulfurtransferase	Cytoplasm	enzyme	
NDUFA7	NDUFA7	NADH:ubiquinone oxidoreductase subunit A7	Cytoplasm	enzyme	
PDXDC1	PDXDC1	pyridoxal dependent decarboxylase domain containing 1	Cytoplasm	other	
SRC	SRC	SRC proto-oncogene, non-receptor tyrosine kinase	Cytoplasm	kinase	bosutinib/corticosteroid, PP-121, tirbanibulin, CGP77675, WH-4-025, ZM 306416, SI163, corticosteroid/dasatinib, XL999, AZ12672857, PD173955, S7, AZM-475271, saracatinib, bosutinib/dexamethasone/vincristine, KX02, blinatumomab/dasatinib, bosutinib/rituximab, S29, WH-4-023, SC-204303, PD162531, dasatinib/dexamethasone/vincristine, SM1-71, CCT239065, dasatinib/dexamethasone, bosutinib, cytarabine/das
CYB5R3	CYB5R3	cytochrome b5 reductase 3	Cytoplasm	enzyme	
SSR1	SSR1	signal sequence receptor subunit 1	Cytoplasm	other	
NFS1	NFS1	NFS1 cysteine desulfurase	Cytoplasm	enzyme	
OSBPL9	OSBPL9	oxysterol binding protein like 9	Cytoplasm	transporter	
TMED10	TMED10	transmembrane p24 trafficking protein 10	Cytoplasm	transporter	
IDH3G	IDH3G	isocitrate dehydrogenase (NAD(+)) 3 non-catalytic subunit gamma	Cytoplasm	enzyme	
MAPRE2	MAPRE2	microtubule associated protein RP/EB family member 2	Cytoplasm	other	
ARFGAP3	ARFGAP3	ADP ribosylation factor GTPase activating protein 3	Cytoplasm	transporter	
MICU2	MICU2	mitochondrial calcium uptake 2	Cytoplasm	other	
SEC62	SEC62	SEC62 homolog, preprotein translocation factor	Cytoplasm	transporter	
ENO1	ENO1	enolase 1	Cytoplasm	enzyme	
TIPRL	TIPRL	TOR signaling pathway regulator	Cytoplasm	other	
P4HA1	P4HA1	prolyl 4-hydroxylase subunit alpha 1	Cytoplasm	enzyme	
NDUFB11	NDUFB11	NADH:ubiquinone oxidoreductase subunit B11	Cytoplasm	enzyme	
GLS	GLS	glutaminase	Cytoplasm	enzyme	telaglenastat
PEX5	PEX5	peroxisomal biogenesis factor 5	Cytoplasm	other	
EPS8L2	EPS8L2	EPS8 like 2	Cytoplasm	other	
OTUB1	OTUB1	OTU deubiquitinase, ubiquitin aldehyde binding 1	Cytoplasm	enzyme	
PLGRKT	PLGRKT	plasminogen receptor with a C-terminal lysine	Cytoplasm	other	
YARS	YARS1	tyrosyl-tRNA synthetase 1	Cytoplasm	enzyme	
RPS27L	RPS27L	ribosomal protein S27 like	Cytoplasm	translation regulator	
PPA2	PPA2	inorganic pyrophosphatase 2	Cytoplasm	enzyme	
SRPR	SRPRA	SRP receptor subunit alpha	Cytoplasm	other	

ATP6V0A1	ATP6V0A1	ATPase H+ transporting V0 subunit a1	Cytoplasm	transporter	
PPIP5K2	PPIP5K2	diphosphoinositol pentakisphosphate kinase 2	Cytoplasm	kinase	
CAT	CAT	catalase	Cytoplasm	enzyme	fomepizole
RPS6KA1	RPS6KA1	ribosomal protein S6 kinase A1	Cytoplasm	kinase	APIO-EE-07, BI-D1870, PMD-026, LJH685
GORASP2	GORASP2	golgi reassembly stacking protein 2	Cytoplasm	other	
CPNE1	CPNE1	copine 1	Cytoplasm	peptidase	
RPL37A	RPL37A	ribosomal protein L37a	Cytoplasm	other	
ELAVL1	ELAVL1	ELAV like RNA binding protein 1	Cytoplasm	other	
ALG5	ALG5	ALG5 dolichyl-phosphate beta-glucosyltransferase	Cytoplasm	enzyme	
USP5	USP5	ubiquitin specific peptidase 5	Cytoplasm	peptidase	
IGBP1	IGBP1	immunoglobulin binding protein 1	Cytoplasm	phosphatase	
PITRM1	PITRM1	pitrilysin metallopeptidase 1	Cytoplasm	peptidase	
GUK1	GUK1	guanylate kinase 1	Cytoplasm	kinase	
EIF4H	EIF4H	eukaryotic translation initiation factor 4H	Cytoplasm	translation regulator	
LAP3	LAP3	leucine aminopeptidase 3	Cytoplasm	peptidase	
PRKACA	PRKACA	protein kinase cAMP-activated catalytic subunit alpha	Cytoplasm	kinase	
ACP6	ACP6	acid phosphatase 6, lysophosphatidic	Cytoplasm	phosphatase	
RPL11	RPL11	ribosomal protein L11	Cytoplasm	other	
ETFB	ETFB	electron transfer flavoprotein subunit beta	Cytoplasm	transporter	
IDH1	IDH1	isocitrate dehydrogenase (NADP(+)) 1	Cytoplasm	enzyme	olutasidenib, IDH305, ivosidenib, DS-1001, HMPL-306, BAY1436032, KY100001, IDH1 inhibitor,
RPL8	RPL8	ribosomal protein L8	Cytoplasm	other	
TBRG4	TBRG4	transforming growth factor beta regulator 4	Cytoplasm	other	
RAB1A	RAB1A	RAB1A, member RAS oncogene family	Cytoplasm	enzyme	
USO1	USO1	USO1 vesicle transport factor	Cytoplasm	other	
CTSA	CTSA	cathepsin A	Cytoplasm	peptidase	
COLGALT1	COLGALT1	collagen beta(1-O)galactosyltransferase 1	Cytoplasm	enzyme	
PFDN4	PFDN4	prefoldin subunit 4	Cytoplasm	other	
RPS25	RPS25	ribosomal protein S25	Cytoplasm	other	
DNM1L	DNM1L	dynamin 1 like	Cytoplasm	enzyme	
EXOC7	EXOC7	exocyst complex component 7	Cytoplasm	transporter	
MPRIP	MPRIP	myosin phosphatase Rho interacting protein	Cytoplasm	other	
TRAPPC3	TRAPPC3	trafficking protein particle complex subunit 3	Cytoplasm	other	birabresib
GYG1	GYG1	glycogenin 1	Cytoplasm	enzyme	
RAB18	RAB18	RAB18, member RAS oncogene family	Cytoplasm	enzyme	
HSDL2	HSDL2	hydroxysteroid dehydrogenase like 2	Cytoplasm	transporter	
AKR1A1	AKR1A1	aldo-keto reductase family 1 member A1	Cytoplasm	enzyme	aldose reductase
GAPDH	GAPDH	glyceraldehyde-3-phosphate dehydrogenase	Cytoplasm	enzyme	
SEC61B	SEC61B	SEC61 translocon subunit beta	Cytoplasm	transporter	
COMMD9	COMMD9	COMM domain containing 9	Cytoplasm	other	
GALNT1	GALNT1	polypeptide N-acetylgalactosaminyltransferase 1	Cytoplasm	enzyme	
PSME2	PSME2	proteasome activator subunit 2	Cytoplasm	peptidase	
ARCN1	ARCN1	archain 1	Cytoplasm	other	
MECR	MECR	mitochondrial trans-2-enoyl-CoA reductase	Cytoplasm	enzyme	
SEC24A	SEC24A	SEC24 homolog A, COPII coat complex component	Cytoplasm	transporter	
GLB1	GLB1	galactosidase beta 1	Cytoplasm	enzyme	
GCN1L1	GCN1	GCN1 activator of EIF2AK4	Cytoplasm	translation regulator	
ERGIC2	ERGIC2	ERGIC and golgi 2	Cytoplasm	other	

MTCH2	MTCH2	mitochondrial carrier 2	Cytoplasm	other	
ME1	ME1	malic enzyme 1	Cytoplasm	enzyme	
NME1	NME1	NME/NM23 nucleoside diphosphate kinase 1	Cytoplasm	kinase	
RPS9	RPS9	ribosomal protein S9	Cytoplasm	translation regulator	
APRT	APRT	adenine phosphoribosyltransferase	Cytoplasm	enzyme	
HGS	HGS*	hepatocyte growth factor-regulated tyrosine kinase substrate	Cytoplasm	other	
ACSL4	ACSL4	acyl-CoA synthetase long chain family member 4	Cytoplasm	enzyme	
CAPZB	CAPZB	capping actin protein of muscle Z-line subunit beta	Cytoplasm	other	vacuolin-1
SCO1	SCO1	synthesis of cytochrome C oxidase 1	Cytoplasm	other	
COMT	COMT	catechol-O-methyltransferase	Cytoplasm	enzyme	carbidopa/entacapone/levodopa, nebicapone, opicapone, tolcapone, entacapone, carbidopa/levodopa/tolcapone, tyrphostin
PDLIM5	PDLIM5	PDZ and LIM domain 5	Cytoplasm	other	
TRADD	TRADD	TNFRSF1A associated via death domain	Cytoplasm	other	
STT3B	STT3B	STT3 oligosaccharyltransferase complex catalytic subunit B	Cytoplasm	enzyme	
LTA4H	LTA4H	leukotriene A4 hydrolase	Cytoplasm	enzyme	
CKAP4	CKAP4	cytoskeleton associated protein 4	Cytoplasm	other	
PGK1	PGK1	phosphoglycerate kinase 1	Cytoplasm	kinase	CBR-470-1
OAT	OAT	ornithine aminotransferase	Cytoplasm	enzyme	
IQGAP2	IQGAP2	IQ motif containing GTPase activating protein 2	Cytoplasm	other	
PLOD1	PLOD1	procollagen-lysine,2-oxoglutarate 5-dioxygenase 1	Cytoplasm	enzyme	
TBCB	TBCB	tubulin folding cofactor B	Cytoplasm	other	
MYO1B	MYO1B	myosin IB	Cytoplasm	other	
WASL	WASL	WASP like actin nucleation promoting factor	Cytoplasm	other	
TOMM20	TOMM20	translocase of outer mitochondrial membrane 20	Cytoplasm	transporter	
UGGT1	UGGT1	UDP-glucose glycoprotein glucosyltransferase 1	Cytoplasm	enzyme	
PACSIN2	PACSIN2	protein kinase C and casein kinase substrate in neurons 2	Cytoplasm	other	
RDH13	RDH13	retinol dehydrogenase 13	Cytoplasm	enzyme	
SPTLC1	SPTLC1	serine palmitoyltransferase long chain base subunit 1	Cytoplasm	enzyme	
NDUFA6	NDUFA6	NADH:ubiquinone oxidoreductase subunit A6	Cytoplasm	enzyme	
VCPIP1	VCPIP1	valosin containing protein interacting protein 1	Cytoplasm	peptidase	
SQRDL	SQOR	sulfide quinone oxidoreductase	Cytoplasm	enzyme	
GALK1	GALK1	galactokinase 1	Cytoplasm	kinase	
ARL1	ARL1	ADP ribosylation factor like GTPase 1	Cytoplasm	enzyme	
DPYSL2	DPYSL2	dihydropyrimidinase like 2	Cytoplasm	enzyme	
GOLM1	GOLM1	golgi membrane protein 1	Cytoplasm	other	
EIF3B	EIF3B	eukaryotic translation initiation factor 3 subunit B	Cytoplasm	translation regulator	
AK3	AK3	adenylate kinase 3	Cytoplasm	kinase	
RAB8B	RAB8B	RAB8B, member RAS oncogene family	Cytoplasm	enzyme	
FASN	FASN	fatty acid synthase	Cytoplasm	enzyme	TVB-2640, orlistat, cerulenin, 3-V bioscience-2640,
SFXN2	SFXN2	sideroflexin 2	Cytoplasm	transporter	
PRDX6	PRDX6	peroxiredoxin 6	Cytoplasm	enzyme	
RAB3GAP1	RAB3GAP1	RAB3 GTPase activating protein catalytic subunit 1	Cytoplasm	other	
MRI1	MRI1	methylthioribose-1-phosphate isomerase 1	Cytoplasm	translation regulator	
PSMA3	PSMA3	proteasome 20S subunit alpha 3	Cytoplasm	peptidase	

FARSB	FARSB	phenylalanyl-tRNA synthetase subunit beta	Cytoplasm	enzyme	
ASCC3	ASCC3	activating signal cointegrator 1 complex subunit 3	Cytoplasm	enzyme	
TGM2	TGM2	transglutaminase 2	Cytoplasm	enzyme	
STAM	STAM	signal transducing adaptor molecule	Cytoplasm	other	
ARHGEF7	ARHGEF7	Rho guanine nucleotide exchange factor 7	Cytoplasm	other	
VPS26A	VPS26A	VPS26 retromer complex component A	Cytoplasm	transporter	
SQSTM1	SQSTM1	sequestosome 1	Cytoplasm	transcription regulator	
SARS2	SARS2	seryl-tRNA synthetase 2, mitochondrial	Cytoplasm	enzyme	
ACAT1	ACAT1	acetyl-CoA acetyltransferase 1	Cytoplasm	enzyme	
TSSC1	EIPR1	EARP complex and GARP complex interacting protein 1	Cytoplasm	other	
DAP3	DAP3	death associated protein 3	Cytoplasm	other	
APEH	APEH	acylaminoacyl-peptide hydrolase	Cytoplasm	peptidase	
CAPZA2	CAPZA2	capping actin protein of muscle Z-line subunit alpha 2	Cytoplasm	other	
PCYT1A	PCYT1A	phosphate cytidylyltransferase 1A, choline	Cytoplasm	enzyme	
CYFIP1	CYFIP1	cytoplasmic FMR1 interacting protein 1	Cytoplasm	translation regulator	
GAN	GAN	gigaxonin	Cytoplasm	other	
SRI	SRI	sorcin	Cytoplasm	transporter	
SGPL1	SGPL1	sphingosine-1-phosphate lyase 1	Cytoplasm	enzyme	
YWHAE	YWHAE	tyrosine 3-monooxygenase/tryptophan 5-monooxygenase activation protein epsilon	Cytoplasm	other	
MRPL24	MRPL24	mitochondrial ribosomal protein L24	Cytoplasm	other	
AFG3L2	AFG3L2	AFG3 like matrix AAA peptidase subunit 2	Cytoplasm	peptidase	
NDUFS8	NDUFS8	NADH:ubiquinone oxidoreductase core subunit S8	Cytoplasm	enzyme	
EIF2B3	EIF2B3	eukaryotic translation initiation factor 2B subunit gamma	Cytoplasm	translation regulator	
EDC3	EDC3	enhancer of mRNA decapping 3	Cytoplasm	other	
DYNLT1	DYNLT1	dynein light chain Tctex-type 1	Cytoplasm	other	
COPG1	COPG1	COPI coat complex subunit gamma 1	Cytoplasm	transporter	
ACOX1	ACOX1	acyl-CoA oxidase 1	Cytoplasm	enzyme	
KIF5B	KIF5B	kinesin family member 5B	Cytoplasm	other	
TWF1	TWF1	twinfilin actin binding protein 1	Cytoplasm	kinase	
CSDE1	CSDE1	cold shock domain containing E1	Cytoplasm	enzyme	
MYH9	MYH9	myosin heavy chain 9	Cytoplasm	enzyme	
ATP5B	ATP5F1B	ATP synthase F1 subunit beta	Cytoplasm	transporter	
HSD17B4	HSD17B4	hydroxysteroid 17-beta dehydrogenase 4	Cytoplasm	enzyme	
IDH2	IDH2	isocitrate dehydrogenase (NADP(+)) 2	Cytoplasm	enzyme	SH1573, enasidenib, TQB3455, HMPL-306
COPZ1	COPZ1	COPI coat complex subunit zeta 1	Cytoplasm	transporter	
CAMK2D	CAMK2D	calcium/calmodulin dependent protein kinase II delta	Cytoplasm	kinase	
RPS4X	RPS4X	ribosomal protein S4 X-linked	Cytoplasm	other	
RPS3	RPS3	ribosomal protein S3	Cytoplasm	enzyme	
PLCB3	PLCB3	phospholipase C beta 3	Cytoplasm	enzyme	

ARHGEF2	ARHGEF2	Rho/Rac guanine nucleotide exchange factor 2	Cytoplasm	other	paclitaxel/topotecan, bevacizumab/paclitaxel, paclitaxel, lapatinib/paclitaxel, afatinib/paclitaxel, paclitaxel/pertuzumab/trastuzumab, paclitaxel/rituximab, bevacizumab/paclitaxel/topotecan, bortezomib/paclitaxel, paclitaxel/ramucirumab, docetaxel/paclitaxel, paclitaxel/trastuzumab, bevacizumab/paclitaxel/pemetrexed, neratinib/paclitaxel, epirubicin/paclitaxel,
ARF1	ARF1	ADP ribosylation factor 1	Cytoplasm	enzyme	
NECAP2	NECAP2	NECAP endocytosis associated 2	Cytoplasm	other	
ACTR1A	ACTR1A	actin related protein 1A	Cytoplasm	other	
CNN3	CNN3	calponin 3	Cytoplasm	other	
HSPA4	HSPA4	heat shock protein family A (Hsp70) member 4	Cytoplasm	other	
RHOA	RHOA	ras homolog family member A	Cytoplasm	enzyme	
NT5C	NT5C	5', 3'-nucleotidase, cytosolic	Cytoplasm	phosphatase	
GET4	GET4	guided entry of tail-anchored proteins factor 4	Cytoplasm	other	
MYL6	MYL6	myosin light chain 6	Cytoplasm	enzyme	
RPSA	RPSA	ribosomal protein SA	Cytoplasm	translation regulator	
NAGA	NAGA	alpha-N-acetylgalactosaminidase	Cytoplasm	enzyme	
DNAJC3	DNAJC3	DnaJ heat shock protein family (Hsp40) member C3	Cytoplasm	other	
TOM1L2	TOM1L2	target of myb1 like 2 membrane trafficking protein	Cytoplasm	transporter	
RAB21	RAB21	RAB21, member RAS oncogene family	Cytoplasm	enzyme	
RPS8	RPS8	ribosomal protein S8	Cytoplasm	other	
VILL	VILL	villin like	Cytoplasm	other	
CORO1B	CORO1B	coronin 1B	Cytoplasm	other	
COG2	COG2	component of oligomeric golgi complex 2	Cytoplasm	other	
RPS2	RPS2	ribosomal protein S2	Cytoplasm	other	
TXNRD2	TXNRD2	thioredoxin reductase 2	Cytoplasm	enzyme	
DDX42	DDX42	DEAD-box helicase 42	Cytoplasm	enzyme	
AP2M1	AP2M1	adaptor related protein complex 2 subunit mu 1	Cytoplasm	other	
CSK	CSK	C-terminal Src kinase	Cytoplasm	kinase	SC-204303, bosutinib, bosutinib/rituximab, bosutinib/imatinib
LIMA1	LIMA1	LIM domain and actin binding 1	Cytoplasm	other	
UQCRFS1	UQCRFS1	ubiquinol-cytochrome c reductase, Rieske iron-sulfur polypeptide 1	Cytoplasm	enzyme	
SNX4	SNX4	sorting nexin 4	Cytoplasm	transporter	
COPS2	COPS2	COP9 signalosome subunit 2	Cytoplasm	other	
CALR	CALR	calreticulin	Cytoplasm	transcription regulator	
WARS	WARS1	tryptophanyl-tRNA synthetase 1	Cytoplasm	enzyme	
BZW1	BZW1	basic leucine zipper and W2 domains 1	Cytoplasm	translation regulator	

PRKAR1A	PRKAR1A	protein kinase cAMP-dependent type I regulatory subunit alpha	Cytoplasm	kinase	
AP1M1	AP1M1	adaptor related protein complex 1 subunit mu 1	Cytoplasm	transporter	
CYC1	CYC1	cytochrome c1	Cytoplasm	enzyme	
RPL22	RPL22	ribosomal protein L22	Cytoplasm	translation regulator	
EPRS	EPRS1	glutamyl-prolyl-tRNA synthetase 1	Cytoplasm	enzyme	
GM2A	GM2A	ganglioside GM2 activator	Cytoplasm	enzyme	
CD2AP	CD2AP	CD2 associated protein	Cytoplasm	other	
RPS26	RPS26	ribosomal protein S26	Cytoplasm	other	
PCYOX1	PCYOX1	prenylcysteine oxidase 1	Cytoplasm	enzyme	
GFM1	GFM1	G elongation factor mitochondrial 1	Cytoplasm	translation regulator	
NAP1L4	NAP1L4	nucleosome assembly protein 1 like 4	Cytoplasm	other	
MRPS22	MRPS22	mitochondrial ribosomal protein S22	Cytoplasm	other	
TPM1	TPM1	tropomyosin 1	Cytoplasm	other	
GALM	GALM	galactose mutarotase	Cytoplasm	enzyme	
FLNB	FLNB	filamin B	Cytoplasm	other	
ACO1	ACO1	aconitase 1	Cytoplasm	enzyme	
TXNL1	TXNL1	thioredoxin like 1	Cytoplasm	enzyme	
GALNT7	GALNT7	polypeptide N-acetylgalactosaminyltransferase 7	Cytoplasm	enzyme	
CISD2	CISD2	CDGSH iron sulfur domain 2	Cytoplasm	other	
MAN2A1	MAN2A1	mannosidase alpha class 2A member 1	Cytoplasm	enzyme	
GBA	GBA1	glucosylceramidase beta 1	Cytoplasm	enzyme	
ESD	ESD	esterase D	Cytoplasm	enzyme	
RRBP1	RRBP1	ribosome binding protein 1	Cytoplasm	other	
MAT2B	MAT2B	methionine adenosyltransferase 2 non-catalytic beta subunit	Cytoplasm	enzyme	
PRDX5	PRDX5	peroxiredoxin 5	Cytoplasm	enzyme	auranofin
VDAC1	VDAC1	voltage dependent anion channel 1	Cytoplasm	ion channel	
PPIA	PPIA	peptidylprolyl isomerase A	Cytoplasm	enzyme	cyclosporin A/methotrexate, basiliximab/cyclosporine, cyclosporine A/sirolimus/tacrolimus, N-methyl-4-Ile-cyclosporin, alemtuzumab/cyclosporin A, RMC-6291, cyclosporin A,
ERLEC1	ERLEC1	endoplasmic reticulum lectin 1	Cytoplasm	other	
TXNDC5	TXNDC5	thioredoxin domain containing 5	Cytoplasm	enzyme	
NDUFB6	NDUFB6	NADH:ubiquinone oxidoreductase subunit B6	Cytoplasm	enzyme	
CTSD	CTSD	cathepsin D	Cytoplasm	peptidase	
SEC22B	SEC22B	SEC22 homolog B, vesicle trafficking protein	Cytoplasm	other	
OCIAD1	OCIAD1	OCIA domain containing 1	Cytoplasm	other	
AK2	AK2	adenylate kinase 2	Cytoplasm	kinase	
DYNC1I2	DYNC1I2	dynein cytoplasmic 1 intermediate chain 2	Cytoplasm	other	
ALDOA	ALDOA	aldolase, fructose-bisphosphate A	Cytoplasm	enzyme	
FAF2	FAF2	Fas associated factor family member 2	Cytoplasm	other	
M6PR	M6PR	mannose-6-phosphate receptor, cation dependent	Cytoplasm	transporter	alglucosidase alfa
S100A11	S100A11	S100 calcium binding protein A11	Cytoplasm	other	
COASY	COASY	Coenzyme A synthase	Cytoplasm	kinase	
CHMP4B	CHMP4B	charged multivesicular body protein 4B	Cytoplasm	other	
ACADS	ACADS	acyl-CoA dehydrogenase short chain	Cytoplasm	enzyme	
TFG	TFG	trafficking from ER to golgi regulator	Cytoplasm	other	
ECI2	ECI2	enoyl-CoA delta isomerase 2	Cytoplasm	enzyme	
KARS	KARS1	lysyl-tRNA synthetase 1	Cytoplasm	enzyme	

PSMD14	PSMD14	proteasome 26S subunit, non-ATPase 14	Cytoplasm	peptidase	
EEF1A1	EEF1A1	eukaryotic translation elongation factor 1 alpha 1	Cytoplasm	translation regulator	
MRPL44	MRPL44	mitochondrial ribosomal protein L44	Cytoplasm	enzyme	
ETF1	ETF1	eukaryotic translation termination factor 1	Cytoplasm	translation regulator	
EIF4G1	EIF4G1	eukaryotic translation initiation factor 4 gamma 1	Cytoplasm	translation regulator	
UBE4A	UBE4A	ubiquitination factor E4A	Cytoplasm	enzyme	
IDH3A	IDH3A	isocitrate dehydrogenase (NAD(+)) 3 catalytic subunit alpha	Cytoplasm	enzyme	
HSP90AA1	HSP90AA1	heat shock protein 90 alpha family class A member 1	Cytoplasm	enzyme	alvespimycin, retaspimycin, luminespib, pimitespib, cisplatin
NDUFC2	NDUFC2	NADH:ubiquinone oxidoreductase subunit C2	Cytoplasm	enzyme	
SEL1L	SEL1L	SEL1L adaptor subunit of SYVN1 ubiquitin ligase	Cytoplasm	other	
SH2D4A	SH2D4A	SH2 domain containing 4A	Cytoplasm	other	
EIF4B	EIF4B	eukaryotic translation initiation factor 4B	Cytoplasm	translation regulator	
RPS15	RPS15	ribosomal protein S15	Cytoplasm	other	
SEC23A	SEC23A	SEC23 homolog A, COPII coat complex component	Cytoplasm	transporter	
TPD52	TPD52	tumor protein D52	Cytoplasm	other	
PSMA4	PSMA4	proteasome 20S subunit alpha 4	Cytoplasm	peptidase	
PGM2	PGM2	phosphoglucomutase 2	Cytoplasm	enzyme	
ARPC2	ARPC2	actin related protein 2/3 complex subunit 2	Cytoplasm	other	
PRKCSH	PRKCSH	protein kinase C substrate 80K-H	Cytoplasm	enzyme	
GAPVD1	GAPVD1	GTPase activating protein and VPS9 domains 1	Cytoplasm	other	
GLUD1	GLUD1	glutamate dehydrogenase 1	Cytoplasm	enzyme	
GIT1	GIT1	GIT ArfGAP 1	Cytoplasm	other	
OGT	OGT	O-linked N-acetylglucosamine (GlcNAc) transferase	Cytoplasm	enzyme	
AARS2	AARS2	alanyl-tRNA synthetase 2, mitochondrial	Cytoplasm	enzyme	
NBEAL2	NBEAL2	neurobeachin like 2	Cytoplasm	other	
IKBIP	IKBIP	IKBKB interacting protein	Cytoplasm	other	
COTL1	COTL1	coactosin like F-actin binding protein 1	Cytoplasm	other	
UROD	UROD	uroporphyrinogen decarboxylase	Cytoplasm	enzyme	
DPP3	DPP3	dipeptidyl peptidase 3	Cytoplasm	peptidase	
TTLL12	TTLL12	tubulin tyrosine ligase like 12	Cytoplasm	other	
NSF	NSF	N-ethylmaleimide sensitive factor, vesicle fusing ATPase	Cytoplasm	transporter	
ACADSB	ACADSB	acyl-CoA dehydrogenase short/branched chain	Cytoplasm	enzyme	
SCPEP1	SCPEP1	serine carboxypeptidase 1	Cytoplasm	peptidase	
ACAD9	ACAD9	acyl-CoA dehydrogenase family member 9	Cytoplasm	enzyme	
ATP5O	ATP5PO	ATP synthase peripheral stalk subunit OSCP	Cytoplasm	transporter	
RPS20	RPS20	ribosomal protein S20	Cytoplasm	other	
NPEPPS	NPEPPS	aminopeptidase puromycin sensitive	Cytoplasm	peptidase	
RPL21	RPL21	ribosomal protein L21	Cytoplasm	other	
XPNPEP1	XPNPEP1	X-prolyl aminopeptidase 1	Cytoplasm	peptidase	
STARD10	STARD10	StAR related lipid transfer domain containing 10	Cytoplasm	other	
RAP1B	RAP1B	RAP1B, member of RAS oncogene family	Cytoplasm	enzyme	
CAPRIN1	CAPRIN1	cell cycle associated protein 1	Cytoplasm	translation regulator	
ETFDH	ETFDH	electron transfer flavoprotein dehydrogenase	Cytoplasm	enzyme	
PEA15	PEA15	proliferation and apoptosis adaptor protein 15	Cytoplasm	transporter	
EIF4A1	EIF4A1	eukaryotic translation initiation factor 4A1	Cytoplasm	translation regulator	
SPTLC2	SPTLC2	serine palmitoyltransferase long chain base subunit 2	Cytoplasm	enzyme	
UBA6	UBA6	ubiquitin like modifier activating enzyme 6	Cytoplasm	enzyme	
SYMPK	SYMPK	symplekin scaffold protein	Cytoplasm	other	

CLINT1	CLINT1	clathrin interactor 1	Cytoplasm	other	
STX12	STX12	syntaxin 12	Cytoplasm	other	
ABI1	ABI1	abl interactor 1	Cytoplasm	other	
HPCAL1	HPCAL1	hippocalcin like 1	Cytoplasm	other	
HINT2	HINT2	histidine triad nucleotide binding protein 2	Cytoplasm	enzyme	
CALU	CALU	calumenin	Cytoplasm	other	
PTGR1	PTGR1	prostaglandin reductase 1	Cytoplasm	enzyme	
ITPA	ITPA	inosine triphosphatase	Cytoplasm	enzyme	
ANKFY1	ANKFY1	ankyrin repeat and FYVE domain containing 1	Cytoplasm	transcription regulator	
HSPA5	HSPA5	heat shock protein family A (Hsp70) member 5	Cytoplasm	enzyme	SHetA2, PAT-SM6
SERPINB6	SERPINB6	serpin family B member 6	Cytoplasm	other	
H6PD	H6PD	hexose-6-phosphate dehydrogenase/glucose 1-dehydrogenase	Cytoplasm	enzyme	
RALA	RALA	RAS like proto-oncogene A	Cytoplasm	enzyme	
CAD	CAD	carbamoyl-phosphate synthetase 2, aspartate transcarbamylase, and dihydroorotase	Cytoplasm	enzyme	
ETHE1	ETHE1	ETHE1 persulfide dioxygenase	Cytoplasm	enzyme	
HADH	HADH	hydroxyacyl-CoA dehydrogenase	Cytoplasm	enzyme	
PPP2R1A	PPP2R1A	protein phosphatase 2 scaffold subunit Aalpha	Cytoplasm	phosphatase	
ACTR1B	ACTR1B	actin related protein 1B	Cytoplasm	other	
EEF1E1	EEF1E1	eukaryotic translation elongation factor 1 epsilon 1	Cytoplasm	translation regulator	
HK2	HK2	hexokinase 2	Cytoplasm	kinase	tuvatexib
EPHX1	EPHX1	epoxide hydrolase 1	Cytoplasm	peptidase	
IAH1	IAH1	isoamyl acetate hydrolyzing esterase 1 (putative)	Cytoplasm	other	
CPNE3	CPNE3	copine 3	Cytoplasm	kinase	
TNFAIP8	TNFAIP8	TNF alpha induced protein 8	Cytoplasm	other	
GNB2L1	RACK1	receptor for activated C kinase 1	Cytoplasm	enzyme	
BCL2L1	BCL2L1	BCL2 like 1	Cytoplasm	other	LP-118,
EIF2A	EIF2A	eukaryotic translation initiation factor 2A	Cytoplasm	translation regulator	
MAPK3	MAPK3	mitogen-activated protein kinase 3	Cytoplasm	kinase	ulixertinib, HE3286, ASN007, ASTX029, HH2710, tyrphostin AG 1288, KO-947, ZSET 1446, SM1-71, temuterkib, Vx-11e, ATG-017, FR 180204, (Z)-3-(2-aminoethyl)-
CTSL	CTSL	cathepsin L	Cytoplasm	peptidase	cathepsin L inhibitor
CRYZ	CRYZ	crystallin zeta	Cytoplasm	enzyme	
PAFAH1B2	PAFAH1B2	platelet activating factor acetylhydrolase 1b catalytic subunit 2	Cytoplasm	enzyme	
MRPS27	MRPS27	mitochondrial ribosomal protein S27	Cytoplasm	other	
PIGS	PIGS	phosphatidylinositol glycan anchor biosynthesis class S	Cytoplasm	enzyme	
EDC4	EDC4	enhancer of mRNA decapping 4	Cytoplasm	other	
HSP90B1	HSP90B1	heat shock protein 90 beta family member 1	Cytoplasm	other	alvespimycin, retaspimycin, cisplatin
EIF3C	EIF3C	eukaryotic translation initiation factor 3 subunit C	Cytoplasm	translation regulator	
SCAMP1	SCAMP1	secretory carrier membrane protein 1	Cytoplasm	transporter	
AP3D1	AP3D1	adaptor related protein complex 3 subunit delta 1	Cytoplasm	other	
ATP6V1G1	ATP6V1G1	ATPase H+ transporting V1 subunit G1	Cytoplasm	transporter	
RPL27A	RPL27A	ribosomal protein L27a	Cytoplasm	other	

CYP51A1	CYP51A1	cytochrome P450 family 51 subfamily A member 1	Cytoplasm	enzyme	voriconazole, efinaconazole, posaconazole, ketoconazole, terconazole, sertaconazole, bifonazole, oxiconazole, fluconazole, itraconazole, clotrimazole, miconazole, econazole, betamethasone /clotrimazole, luliconazole
SNX3	SNX3	sorting nexin 3	Cytoplasm	transporter	
GOLIM4	GOLIM4	golgi integral membrane protein 4	Cytoplasm	other	
ADSL	ADSL	adenylosuccinate lyase	Cytoplasm	enzyme	
UBXN1	UBXN1	UBX domain protein 1	Cytoplasm	other	
RAB5C	RAB5C	RAB5C, member RAS oncogene family	Cytoplasm	enzyme	
PIP4K2C	PIP4K2C	phosphatidylinositol-5-phosphate 4-kinase type 2 gamma	Cytoplasm	kinase	SM1-71
ERP44	ERP44	endoplasmic reticulum protein 44	Cytoplasm	enzyme	
CAPN5	CAPN5	calpain 5	Cytoplasm	peptidase	
ABHD11	ABHD11	abhydrolase domain containing 11	Cytoplasm	enzyme	
ARHGEF1	ARHGEF1	Rho guanine nucleotide exchange factor 1	Cytoplasm	other	
CLYBL	CLYBL	citramalyl-CoA lyase	Cytoplasm	enzyme	
PCCA	PCCA	propionyl-CoA carboxylase subunit alpha	Cytoplasm	enzyme	
RPL23	RPL23	ribosomal protein L23	Cytoplasm	other	
SDHC	SDHC	succinate dehydrogenase complex subunit C	Cytoplasm	enzyme	
GBF1	GBF1	golgi brefeldin A resistant guanine nucleotide exchange factor 1	Cytoplasm	other	
KLC1	KLC1	kinesin light chain 1	Cytoplasm	other	
GALE	GALE	UDP-galactose-4-epimerase	Cytoplasm	enzyme	
NT5C2	NT5C2	5'-nucleotidase, cytosolic II	Cytoplasm	phosphatase	
PDK1	PDK1	pyruvate dehydrogenase kinase 1	Cytoplasm	kinase	dichloroacetic acid, bis(4-morpholinyl thiocarbonyl)disulfide
AKAP13	AKAP13	A-kinase anchoring protein 13	Cytoplasm	other	
DLD	DLD	dihydrolipoamide dehydrogenase	Cytoplasm	enzyme	hexachlorophe
HTRA2	HTRA2	HtrA serine peptidase 2	Cytoplasm	peptidase	
HBS1L	HBS1L	HBS1 like translational GTPase	Cytoplasm	translation regulator	
CRK	CRK	CRK proto-oncogene, adaptor protein	Cytoplasm	other	
DENND4C	DENND4C	DENN domain containing 4C	Cytoplasm	other	
PKM	PKM	pyruvate kinase M1/2	Cytoplasm	kinase	TP-1454, CAP-
RPS7	RPS7	ribosomal protein S7	Cytoplasm	other	
HSPA1B	HSPA1A/HSPA1B	heat shock protein family A (Hsp70) member 1A	Cytoplasm	enzyme	
PSMD13	PSMD13	proteasome 26S subunit, non-ATPase 13	Cytoplasm	peptidase	
ARHGDIA	ARHGDIA	Rho GDP dissociation inhibitor alpha	Cytoplasm	other	
ARFGEF1	ARFGEF1	ADP ribosylation factor guanine nucleotide exchange factor 1	Cytoplasm	other	
AUP1	AUP1	AUP1 lipid droplet regulating VLDL assembly factor	Cytoplasm	other	
GSTO1	GSTO1	glutathione S-transferase omega 1	Cytoplasm	enzyme	
IARS2	IARS2	isoleucyl-tRNA synthetase 2, mitochondrial	Cytoplasm	enzyme	
CDC42BPB	CDC42BPB	CDC42 binding protein kinase beta	Cytoplasm	kinase	
SCAMP2	SCAMP2	secretory carrier membrane protein 2	Cytoplasm	other	
NDUFS5	NDUFS5	NADH:ubiquinone oxidoreductase subunit S5	Cytoplasm	enzyme	
CISD1	CISD1	CDGSH iron sulfur domain 1	Cytoplasm	enzyme	
CCT5	CCT5	chaperonin containing TCP1 subunit 5	Cytoplasm	other	

SPCS2	SPCS2	signal peptidase complex subunit 2	Cytoplasm	other	
FDXR	FDXR	ferredoxin reductase	Cytoplasm	enzyme	
CNP	CNP	2',3'-cyclic nucleotide 3' phosphodiesterase	Cytoplasm	enzyme	
NNT	NNT	nicotinamide nucleotide transhydrogenase	Cytoplasm	enzyme	
PSMD7	PSMD7	proteasome 26S subunit, non-ATPase 7	Cytoplasm	other	
PFN1	PFN1	profilin 1	Cytoplasm	other	
NUDT5	NUDT5	nudix hydrolase 5	Cytoplasm	phosphatase	
ATP5C1	ATP5F1C	ATP synthase F1 subunit gamma	Cytoplasm	transporter	
P4HB	P4HB	prolyl 4-hydroxylase subunit beta	Cytoplasm	enzyme	
GIPC1	GIPC1	GIPC PDZ domain containing family member 1	Cytoplasm	other	
PSMB1	PSMB1	proteasome 20S subunit beta 1	Cytoplasm	peptidase	bortezomib/cladribine/rituximab, bortezomib/dexamethasone/pomalidomide, bortezomib/doxorubicin, bortezomib/dexamethasone/thalidomide, bortezomib/paclitaxel, carfilzomib, bortezomib/sorafenib, bortezomib/fulvestrant, bortezomib/rituximab, bortezomib/lenalidomide, carfilzomib/dexamethasone/lenalidomide, bortezomib/dexamethasone/lenalidomide, bortezomib/dexamethasone/doxorubicin, bortezomib/de
TXN	TXN	thioredoxin	Cytoplasm	enzyme	PX-12
LRRC16A	CARMIL1	capping protein regulator and myosin 1 linker 1	Cytoplasm	enzyme	
FKBP9	FKBP9	FKBP prolyl isomerase 9	Cytoplasm	enzyme	
ATP6V1D	ATP6V1D	ATPase H+ transporting V1 subunit D	Cytoplasm	transporter	
EIF4G2	EIF4G2	eukaryotic translation initiation factor 4 gamma 2	Cytoplasm	translation regulator	
NDUFA10	NDUFA10	NADH:ubiquinone oxidoreductase subunit A10	Cytoplasm	transporter	
PSME3	PSME3	proteasome activator subunit 3	Cytoplasm	peptidase	
NME3	NME3	NME/NM23 nucleoside diphosphate kinase 3	Cytoplasm	kinase	
TMSB4X	TMSB10/TMSB4X	thymosin beta 4 X-linked	Cytoplasm	other	
PMM2	PMM2	phosphomannomutase 2	Cytoplasm	enzyme	
FMNL2	FMNL2	formin like 2	Cytoplasm	other	
VPS13C	VPS13C	vacuolar protein sorting 13 homolog C	Cytoplasm	other	
RPS14	RPS14	ribosomal protein S14	Cytoplasm	translation regulator	
ATP2A2	ATP2A2	ATPase sarcoplasmic/endoplasmic reticulum Ca2+ transporting 2	Cytoplasm	transporter	
ATIC	ATIC	5-aminoimidazole-4-carboxamide ribonucleotide formyltransferase/IMP cyclohydrolase	Cytoplasm	enzyme	gemcitabine/pemetrexed, pembrolizumab/pemetrexed, pemetrexed
STRN	STRN	striatin	Cytoplasm	other	
PGM3	PGM3	phosphoglucomutase 3	Cytoplasm	enzyme	
FLNA	FLNA	filamin A	Cytoplasm	other	
BAX	BAX	BCL2 associated X, apoptosis regulator	Cytoplasm	transporter	

FDPS	FDPS	farnesyl diphosphate synthase	Cytoplasm	enzyme	alendronate/cholecalciferol, zoledronic acid, minodronate, ibandronic acid, risedronic acid, alendronic acid, pamidronic
SUCLA2	SUCLA2	succinate-CoA ligase ADP-forming subunit beta	Cytoplasm	enzyme	
NANS	NANS	N-acetylneuraminate synthase	Cytoplasm	enzyme	
FKBP1A	FKBP1A	FKBP prolyl isomerase 1A	Cytoplasm	enzyme	cyclosporine A/sirolimus/tacrolimus, everolimus/fulvestrant, everolimus/pasireotide, corticosteroid/sirolimus, corticosteroid/everolimus/tacrolimus, everolimus, pimecrolimus, temsirolimus, everolimus/exemestane, cyclosporine A/tacrolimus, everolimus/ribociclib, everolimus/letrozole, lenalidomide/temsirolimus, sirolimus, everolimus/gefitinib, everolimus/tamoxifen, metformin/sirolimus,
FCHO2	FCHO2	FCH and mu domain containing endocytic adaptor 2	Cytoplasm	other	
NDUFS7	NDUFS7	NADH:ubiquinone oxidoreductase core subunit S7	Cytoplasm	enzyme	
PRKCA	PRKCA	protein kinase C alpha	Cytoplasm	kinase	aprinocarsen, Go6983, L-threo-safingol, Go 6976, Ro31-8220, ingenol mebutate,
MAT2A	MAT2A	methionine adenosyltransferase 2A	Cytoplasm	enzyme	IDE397, AG-270
NDRG3	NDRG3	NDRG family member 3	Cytoplasm	other	
S100A13	S100A13	S100 calcium binding protein A13	Cytoplasm	other	
VDAC2	VDAC2	voltage dependent anion channel 2	Cytoplasm	ion channel	
DNAJC13	DNAJC13	DnaJ heat shock protein family (Hsp40) member C13	Cytoplasm	other	
SNX17	SNX17	sorting nexin 17	Cytoplasm	transporter	
DNAJC10	DNAJC10	DnaJ heat shock protein family (Hsp40) member C10	Cytoplasm	enzyme	
RPS27	RPS27	ribosomal protein S27	Cytoplasm	other	empesertib
CTSC	CTSC	cathepsin C	Cytoplasm	peptidase	
MPST	MPST	mercaptopyruvate sulfurtransferase	Cytoplasm	enzyme	
MANF	MANF	mesencephalic astrocyte derived neurotrophic factor	Extracellular Space	other	
LGALS4	LGALS4	galectin 4	Extracellular Space	other	
FAM213A	PRXL2A	peroxiredoxin like 2A	Extracellular Space	other	
PLBD2	PLBD2	phospholipase B domain containing 2	Extracellular Space	other	
LGALS3	LGALS3	galectin 3	Extracellular Space	other	GCS-100, GB1211, GR-MD-02, GM-CT-

CFL2	CFL2	cofilin 2	Extracellular Space	other	
APOOL	APOOL	apolipoprotein O like	Extracellular Space	other	
APOO	APOO	apolipoprotein O	Extracellular Space	other	
MYDGF	MYDGF	myeloid derived growth factor	Extracellular Space	cytokine	
NID1	NID1	nidogen 1	Extracellular Space	other	
SCRN2	SCRN2	secernin 2	Extracellular Space	other	
GSN	GSN	gelsolin	Extracellular Space	other	
TMEM214	TMEM214	transmembrane protein 214	Extracellular Space	other	
PRPSAP1	PRPSAP1	phosphoribosyl pyrophosphate synthetase associated protein 1	Extracellular Space	other	
MYH14	MYH14	myosin heavy chain 14	Extracellular Space	enzyme	mavacamten
ENDOD1	ENDOD1	endonuclease domain containing 1	Extracellular Space	enzyme	
SDF2	SDF2	stromal cell derived factor 2	Extracellular Space	enzyme	
HDGF	HDGF	heparin binding growth factor	Extracellular Space	growth factor	
ATP13A1	ATP13A1	ATPase 13A1	Extracellular Space	transporter	
MESDC2	MESD	mesoderm development LRP chaperone	Extracellular Space	other	
NPC2	NPC2	NPC intracellular cholesterol transporter 2	Extracellular Space	transporter	
UBXN4	UBXN4	UBX domain protein 4	Extracellular Space	other	
FN1	FN1	fibronectin 1	Extracellular Space	other	ocriplasmin, bifikafusp alfa, L19TNFalpha, L19-IL2 monoclonal antibody-cytokine fusion protein/L19TNF alpha, AS1409, iodine I 131 anti-fibronectin antibody fragment L19-
TINAGL1	TINAGL1	tubulointerstitial nephritis antigen like 1	Extracellular Space	transporter	
POGLUT1	POGLUT1	protein O-glucosyltransferase 1	Extracellular Space	enzyme	
CCDC47	CCDC47	coiled-coil domain containing 47	Extracellular Space	other	
NAMPT	NAMPT	nicotinamide phosphoribosyltransferase	Extracellular Space	cytokine	OT-82, STF-118804, daporinad, KPT-9274
LAD1	LAD1	ladinin 1	Extracellular Space	other	
IDE	IDE	insulin degrading enzyme	Extracellular Space	peptidase	bacitracin
COBLL1	COBLL1	cordon-bleu WH2 repeat protein like 1	Extracellular Space	other	
SEC23B	SEC23B	SEC23 homolog B, COPII coat complex component	Extracellular Space	transporter	
LGALS1	LGALS1	galectin 1	Extracellular Space	other	OTX008
WDR1	WDR1	WD repeat domain 1	Extracellular Space	other	
GPI	GPI	glucose-6-phosphate isomerase	Extracellular Space	enzyme	
ERAP1	ERAP1	endoplasmic reticulum aminopeptidase 1	Extracellular Space	peptidase	
ECM1	ECM1	extracellular matrix protein 1	Extracellular Space	transporter	
LAMA5	LAMA5	laminin subunit alpha 5	Extracellular Space	other	
LAMC1	LAMC1	laminin subunit gamma 1	Extracellular Space	other	
TRABD	TRABD	TraB domain containing	Extracellular Space	other	
SERPINH1	SERPINH1	serpin family H member 1	Extracellular Space	other	

LAMB1	LAMB1	laminin subunit beta 1	Extracellular Space	other	
DDRGK1	DDRGK1	DDRGK domain containing 1	Extracellular Space	other	
REEP5	REEP5	receptor accessory protein 5	Extracellular Space	transporter	
AIMP1	AIMP1	aminoacyl tRNA synthetase complex interacting multifunctional protein 1	Extracellular Space	cytokine	
APOA1BP	NAXE	NAD(P)HX epimerase	Extracellular Space	enzyme	
MIF	MIF	macrophage migration inhibitory factor	Extracellular Space	cytokine	imalumab
HSPG2	HSPG2	heparan sulfate proteoglycan 2	Extracellular Space	enzyme	
FAM49B	CYRIB	CYFIP related Rac1 interactor B	Extracellular Space	other	
K7EPS6	HDGFL2	HDGF like 2	Nucleus	other	
DPY30	DPY30	dpy-30 histone methyltransferase complex regulatory subunit	Nucleus	other	
SUMO1	SUMO1	small ubiquitin like modifier 1	Nucleus	enzyme	
DDB1	DDB1	damage specific DNA binding protein 1	Nucleus	other	
XAB2	XAB2	XPA binding protein 2	Nucleus	other	
DDX5	DDX5	DEAD-box helicase 5	Nucleus	enzyme	
THRAP3	THRAP3	thyroid hormone receptor associated protein 3	Nucleus	transcription regulator	
IPO9	IPO9	importin 9	Nucleus	other	
NAT10	NAT10	N-acetyltransferase 10	Nucleus	enzyme	
SLK	SLK	STE20 like kinase	Nucleus	kinase	
BAZ1B	BAZ1B	bromodomain adjacent to zinc finger domain 1B	Nucleus	transcription regulator	
LMNB2	LMNB2	lamin B2	Nucleus	other	
LARP7	LARP7	La ribonucleoprotein 7, transcriptional regulator	Nucleus	other	
PDCD5	PDCD5	programmed cell death 5	Nucleus	other	
PDCD4	PDCD4	programmed cell death 4	Nucleus	other	
RAD23B	RAD23B	RAD23 homolog B, nucleotide excision repair protein	Nucleus	other	
VWA5A	VWA5A	von Willebrand factor A domain containing 5A	Nucleus	other	
TIAL1	TIAL1	TIA1 cytotoxic granule associated RNA binding protein like 1	Nucleus	transcription regulator	
HIST1H1E	H1-4	H1.4 linker histone, cluster member	Nucleus	other	
CCAR1	CCAR1	cell division cycle and apoptosis regulator 1	Nucleus	transcription regulator	
POLR2B	POLR2B	RNA polymerase II subunit B	Nucleus	enzyme	
SRSF11	SRSF11	serine and arginine rich splicing factor 11	Nucleus	other	
SF3B6	SF3B6	splicing factor 3b subunit 6	Nucleus	other	
XRN2	XRN2	5'-3' exoribonuclease 2	Nucleus	enzyme	
FUS	FUS	FUS RNA binding protein	Nucleus	transcription regulator	
ELAC2	ELAC2	elaC ribonuclease Z 2	Nucleus	enzyme	
DDX18	DDX18	DEAD-box helicase 18	Nucleus	enzyme	
LUC7L2	LUC7L2	LUC7 like 2, pre-mRNA splicing factor	Nucleus	other	
SRP72	SRP72	signal recognition particle 72	Nucleus	kinase	
BTF3	BTF3	basic transcription factor 3	Nucleus	transcription regulator	
HP1BP3	HP1BP3	heterochromatin protein 1 binding protein 3	Nucleus	other	
TSN	TSN	translin	Nucleus	other	
NOP58	NOP58	NOP58 ribonucleoprotein	Nucleus	enzyme	
RPRD1B	RPRD1B	regulation of nuclear pre-mRNA domain containing 1B	Nucleus	other	
FKBP4	FKBP4	FKBP prolyl isomerase 4	Nucleus	enzyme	
SF3A3	SF3A3	splicing factor 3a subunit 3	Nucleus	other	
HCFC1	HCFC1	host cell factor C1	Nucleus	transcription regulator	
AHNAK	AHNAK	AHNAK nucleoprotein	Nucleus	other	
OSGEP	OSGEP	O-sialoglycoprotein endopeptidase	Nucleus	peptidase	
EIF4A3	EIF4A3	eukaryotic translation initiation factor 4A3	Nucleus	enzyme	
STRIP1	STRIP1	striatin interacting protein 1	Nucleus	other	
SNRPC	SNRPC	small nuclear ribonucleoprotein polypeptide C	Nucleus	other	

SET	SET	SET nuclear proto-oncogene	Nucleus	phosphatase	
GPS1	GPS1	G protein pathway suppressor 1	Nucleus	other	
HNRNPK	HNRNPK	heterogeneous nuclear ribonucleoprotein K	Nucleus	other	
FKBP5	FKBP5	FKBP prolyl isomerase 5	Nucleus	enzyme	
CUL3	CUL3	cullin 3	Nucleus	enzyme	
ERH	ERH	ERH mRNA splicing and mitosis factor	Nucleus	other	
XRCC1	XRCC1	X-ray repair cross complementing 1	Nucleus	other	
AHCTF1	AHCTF1	AT-hook containing transcription factor 1	Nucleus	transcription regulator	
RUVBL2	RUVBL2	RuvB like AAA ATPase 2	Nucleus	transcription regulator	
RAVER1	RAVER1	ribonucleoprotein, PTB binding 1	Nucleus	other	
SRSF3	SRSF3	serine and arginine rich splicing factor 3	Nucleus	other	
GSK3B	GSK3B	glycogen synthase kinase 3 beta	Nucleus	kinase	aloisine A, enzastaurin, GSK-3beta inhibitor II, indirubin-3'-monoxime, glycogen synthase kinase-3beta inhibitor,
UPF1	UPF1	UPF1 RNA helicase and ATPase	Nucleus	enzyme	
CTDP1	CTDP1	CTD phosphatase subunit 1	Nucleus	phosphatase	
SNW1	SNW1	SNW domain containing 1	Nucleus	transcription regulator	
SETD3	SETD3	SET domain containing 3, actin N3(tau)-histidine methyltransferase	Nucleus	enzyme	
BANF1	BANF1	BAF nuclear assembly factor 1	Nucleus	other	
PES1	PES1	pescadillo ribosomal biogenesis factor 1	Nucleus	other	
PABPN1	PABPN1	poly(A) binding protein nuclear 1	Nucleus	enzyme	
YBX1	YBX1	Y-box binding protein 1	Nucleus	transcription regulator	
DCTN4	DCTN4	dynactin subunit 4	Nucleus	other	
NUTF2	NUTF2	nuclear transport factor 2	Nucleus	other	
HNRNPH1	HNRNPH1	heterogeneous nuclear ribonucleoprotein H1	Nucleus	other	
LMNB1	LMNB1	lamin B1	Nucleus	other	
PPP5C	PPP5C	protein phosphatase 5 catalytic subunit	Nucleus	phosphatase	
PTBP3	PTBP3	polypyrimidine tract binding protein 3	Nucleus	other	
NDRG1	NDRG1	N-myc downstream regulated 1	Nucleus	kinase	
TNPO1	TNPO1	transportin 1	Nucleus	transporter	
PCNP	PCNP	PEST proteolytic signal containing nuclear protein	Nucleus	other	
PURA	PURA	purine rich element binding protein A	Nucleus	transcription regulator	
RPS3A	RPS3A	ribosomal protein S3A	Nucleus	other	
DHX38	DHX38	DEAH-box helicase 38	Nucleus	enzyme	
SKIV2L2	MTREX	Mtr4 exosome RNA helicase	Nucleus	enzyme	
SMARCA4	SMARCA4	SWI/SNF related, matrix associated, actin dependent regulator of chromatin, subfamily a, member 4	Nucleus	transcription regulator	FHD-286
TMEM43	TMEM43	transmembrane protein 43	Nucleus	other	
PRPF8	PRPF8	pre-mRNA processing factor 8	Nucleus	other	
SSB	SSB	small RNA binding exonuclease protection factor La	Nucleus	enzyme	
RPL3	RPL3	ribosomal protein L3	Nucleus	other	cytarabine/daunorubicin/omacetaxine mepesuccinate, omacetaxine mepesuccinate, cytarabine/doxorubicin/filgrastim/omacetaxine
RBBP7	RBBP7	RB binding protein 7, chromatin remodeling factor	Nucleus	transcription regulator	
CMAS	CMAS	cytidine monophosphate N-acetylneuraminic acid synthetase	Nucleus	enzyme	

CWC22	CWC22	CWC22 spliceosome associated protein homolog	Nucleus	other	
RAD50	RAD50	RAD50 double strand break repair protein	Nucleus	enzyme	
TRIOBP	TRIOBP	TRIO and F-actin binding protein	Nucleus	other	
NCL	NCL	nucleolin	Nucleus	other	AGRO 100, IPP-204106N
MCM2	MCM2	minichromosome maintenance complex component 2	Nucleus	enzyme	
MCM4	MCM4	minichromosome maintenance complex component 4	Nucleus	enzyme	
RPL9	RPL9	ribosomal protein L9	Nucleus	other	
TARDBP	TARDBP	TAR DNA binding protein	Nucleus	transcription regulator	
DDX21	DDX21	DExD-box helicase 21	Nucleus	enzyme	
MTPN	MTPN	myotrophin	Nucleus	transcription regulator	
POLR2A	POLR2A	RNA polymerase II subunit A	Nucleus	enzyme	
DDX17	DDX17	DEAD-box helicase 17	Nucleus	enzyme	
ASH2L	ASH2L	ASH2 like, histone lysine methyltransferase complex subunit	Nucleus	transcription regulator	
CBX3	CBX3	chromobox 3	Nucleus	transcription regulator	
NONO	NONO	non-POU domain containing octamer binding	Nucleus	transcription regulator	
NPM3	NPM3	nucleophosmin/nucleoplasmin 3	Nucleus	other	
ITCH	ITCH	itchy E3 ubiquitin protein ligase	Nucleus	enzyme	
STAMBP	STAMBP	STAM binding protein	Nucleus	enzyme	
PPM1G	PPM1G	protein phosphatase, Mg2+/Mn2+ dependent 1G	Nucleus	phosphatase	
AKAP8	AKAP8	A-kinase anchoring protein 8	Nucleus	other	
SARNP	SARNP	SAP domain containing ribonucleoprotein	Nucleus	transcription regulator	
HDAC1	HDAC1	histone deacetylase 1	Nucleus	enzyme	pyroxamide, R 306465, bortezomib/vorinostat, OKI-179, tributyrin, purinostat, trametinib/vorinostat, belinostat, mocetinostat, HG146, HDAC class I inhibitors,
NEK9	NEK9	NIMA related kinase 9	Nucleus	kinase	
SNRPB2	SNRPB2	small nuclear ribonucleoprotein polypeptide B2	Nucleus	other	
GAK	GAK	cyclin G associated kinase	Nucleus	kinase	SM1-71
DEK	DEK	DEK proto-oncogene	Nucleus	transcription regulator	
PSPC1	PSPC1	paraspeckle component 1	Nucleus	transcription regulator	
HIST1H1D	H1-3	H1.3 linker histone, cluster member	Nucleus	other	
ACTN1	ACTN1	actinin alpha 1	Nucleus	transcription regulator	
HIST1H1C	H1-2	H1.2 linker histone, cluster member	Nucleus	other	
TPR	TPR	translocated promoter region, nuclear basket protein	Nucleus	other	
HIST1H4A	H4C1	H4 clustered histone 1	Nucleus	other	
TNPO2	TNPO2	transportin 2	Nucleus	transporter	
HINT1	HINT1	histidine triad nucleotide binding protein 1	Nucleus	enzyme	
HNRNPU	HNRNPU	heterogeneous nuclear ribonucleoprotein U	Nucleus	transporter	
FLII	FLII	FLII actin remodeling protein	Nucleus	other	
CSNK2A1	CSNK2A1	casein kinase 2 alpha 1	Nucleus	kinase	SM1-71
UBR4	UBR4	ubiquitin protein ligase E3 component n-recognin 4	Nucleus	enzyme	
MYBBP1A	MYBBP1A	MYB binding protein 1a	Nucleus	transcription regulator	
XPO7	XPO7	exportin 7	Nucleus	transporter	
CPSF2	CPSF2	cleavage and polyadenylation specific factor 2	Nucleus	other	
SAFB	SAFB	scaffold attachment factor B	Nucleus	other	

PNP	PNP	purine nucleoside phosphorylase	Nucleus	enzyme	forodesine, PD 141955, purine nucleoside phosphorylase inhibitor
HDLBP	HDLBP	high density lipoprotein binding protein	Nucleus	transporter	
MLLT4	AFDN	afadin, adherens junction formation factor	Nucleus	other	
HMGB1	HMGB1	high mobility group box 1	Nucleus	transcription regulator	
MMS19	MMS19	MMS19 homolog, cytosolic iron-sulfur assembly component	Nucleus	transcription regulator	
LPP	LPP	LIM domain containing preferred translocation partner in lipoma	Nucleus	other	
RAE1	RAE1	ribonucleic acid export 1	Nucleus	other	
RPL10A	RPL10A	ribosomal protein L10a	Nucleus	other	
MTA2	MTA2	metastasis associated 1 family member 2	Nucleus	transcription regulator	
SLTM	SLTM	SAFB like transcription modulator	Nucleus	other	
POLR1C	POLR1C	RNA polymerase I and III subunit C	Nucleus	enzyme	
SMARCC2	SMARCC2	SWI/SNF related, matrix associated, actin dependent regulator of chromatin subfamily c member 2	Nucleus	transcription regulator	
CTNNBL1	CTNNBL1	catenin beta like 1	Nucleus	other	
SMARCC1	SMARCC1	SWI/SNF related, matrix associated, actin dependent regulator of chromatin subfamily c member 1	Nucleus	transcription regulator	
PELP1	PELP1	proline, glutamate and leucine rich protein 1	Nucleus	other	
SFPQ	SFPQ	splicing factor proline and glutamine rich	Nucleus	other	
FUBP3	FUBP3	far upstream element binding protein 3	Nucleus	transcription regulator	
RALY	RALY	RALY heterogeneous nuclear ribonucleoprotein	Nucleus	transcription regulator	
NUP93	NUP93	nucleoporin 93	Nucleus	other	
SKP1	SKP1	S-phase kinase associated protein 1	Nucleus	transcription regulator	
PGRMC2	PGRMC2	progesterone receptor membrane component 2	Nucleus	transporter	
UBE2I	UBE2I	ubiquitin conjugating enzyme E2 I	Nucleus	enzyme	
OSTF1	OSTF1	osteoclast stimulating factor 1	Nucleus	transcription regulator	
PELO	PELO	pelota mRNA surveillance and ribosome rescue factor	Nucleus	other	
CSTF3	CSTF3	cleavage stimulation factor subunit 3	Nucleus	other	
RPL12	RPL12	ribosomal protein L12	Nucleus	other	
XRCC6	XRCC6	X-ray repair cross complementing 6	Nucleus	enzyme	
SUPT5H	SUPT5H	SPT5 homolog, DSIF elongation factor subunit	Nucleus	transcription regulator	
SND1	SND1	staphylococcal nuclease and tudor domain containing 1	Nucleus	transcription regulator	
WDR75	WDR75	WD repeat domain 75	Nucleus	other	
ZMYND8	ZMYND8	zinc finger MYND-type containing 8	Nucleus	transcription regulator	
SNRPA	SNRPA	small nuclear ribonucleoprotein polypeptide A	Nucleus	other	
EFTUD2	EFTUD2	elongation factor Tu GTP binding domain containing 2	Nucleus	enzyme	
SNRPF	SNRPF	small nuclear ribonucleoprotein polypeptide F	Nucleus	other	
EMC3	EMC3	ER membrane protein complex subunit 3	Nucleus	other	
SMC1A	SMC1A	structural maintenance of chromosomes 1A	Nucleus	enzyme	
RCC2	RCC2	regulator of chromosome condensation 2	Nucleus	other	
PSMC6	PSMC6	proteasome 26S subunit, ATPase 6	Nucleus	peptidase	
HUWE1	HUWE1	HECT, UBA and WWE domain containing E3 ubiquitin protein ligase 1	Nucleus	transcription regulator	
TMPO	TMPO	thymopoietin	Nucleus	other	
SRP68	SRP68	signal recognition particle 68	Nucleus	other	

NRBP1	NRBP1	nuclear receptor binding protein 1	Nucleus	kinase	
SMARCA5	SMARCA5	SWI/SNF related, matrix associated, actin dependent regulator of chromatin, subfamily a, member 5	Nucleus	transcription regulator	
CPSF3	CPSF3	cleavage and polyadenylation specific factor 3	Nucleus	enzyme	
MEPCE	MEPCE	methylphosphate capping enzyme	Nucleus	enzyme	
SUMO3	SUMO3	small ubiquitin like modifier 3	Nucleus	other	
STAT3	STAT3	signal transducer and activator of transcription 3	Nucleus	transcription regulator	CAS3/SS3, golotimod, OPB-31121, OPB-51602, danvatirsen, TTI-101, STAT3 inhibitor,
CAPG	CAPG	capping actin protein, gelsolin like	Nucleus	other	
RUVBL1	RUVBL1	RuvB like AAA ATPase 1	Nucleus	transcription regulator	
NAA10	NAA10	N-alpha-acetyltransferase 10, NatA catalytic subunit	Nucleus	enzyme	
PCID2	PCID2	PCI domain containing 2	Nucleus	transcription regulator	
AQR	AQR	aquarius intron-binding spliceosomal factor	Nucleus	enzyme	
CSE1L	CSE1L	chromosome segregation 1 like	Nucleus	transporter	
FTSJ3	FTSJ3	FtsJ RNA 2'-O-methyltransferase 3	Nucleus	enzyme	
U2AF1	LOC102724594/U2AF1	U2 small nuclear RNA auxiliary factor 1	Nucleus	other	
DKC1	DKC1	dyskerin pseudouridine synthase 1	Nucleus	enzyme	
RPL6	RPL6	ribosomal protein L6	Nucleus	other	
SUN2	SUN2	Sad1 and UNC84 domain containing 2	Nucleus	other	
EXOSC8	EXOSC8	exosome component 8	Nucleus	enzyme	
SRSF4	SRSF4	serine and arginine rich splicing factor 4	Nucleus	other	
HMGB2	HMGB2	high mobility group box 2	Nucleus	transcription regulator	
PTBP1	PTBP1	polypyrimidine tract binding protein 1	Nucleus	enzyme	
HEATR1	HEATR1	HEAT repeat containing 1	Nucleus	other	
H1F0	H1-0	H1.0 linker histone	Nucleus	other	
MVP	MVP	major vault protein	Nucleus	other	
HNRNPC	HNRNPC	heterogeneous nuclear ribonucleoprotein C	Nucleus	other	
ZC3H18	ZC3H18	zinc finger CCCH-type containing 18	Nucleus	other	
SF1	SF1	splicing factor 1	Nucleus	transcription regulator	
CUL1	CUL1	cullin 1	Nucleus	enzyme	
DHX9	DHX9	DExH-box helicase 9	Nucleus	enzyme	
PRPF40A	PRPF40A	pre-mRNA processing factor 40 homolog A	Nucleus	other	
DDX19A	DDX19A	DEAD-box helicase 19A	Nucleus	enzyme	
PTMA	PTMA	prothymosin alpha	Nucleus	other	
PRPF4	PRPF4	pre-mRNA processing factor 4	Nucleus	other	
DNAJA2	DNAJA2	DnaJ heat shock protein family (Hsp40) member A2	Nucleus	enzyme	
NOP56	NOP56	NOP56 ribonucleoprotein	Nucleus	other	
NUCB2	NUCB2	nucleobindin 2	Nucleus	other	
SNRPA1	SNRPA1	small nuclear ribonucleoprotein polypeptide A'	Nucleus	other	
HNRNPF	HNRNPF	heterogeneous nuclear ribonucleoprotein F	Nucleus	other	
PREB	PREB	prolactin regulatory element binding	Nucleus	transcription regulator	
SP1	SP1	Sp1 transcription factor	Nucleus	transcription regulator	
GTPBP4	GTPBP4	GTP binding protein 4	Nucleus	enzyme	
GTF2F1	GTF2F1	general transcription factor IIF subunit 1	Nucleus	transcription regulator	
IRF2BP1	IRF2BP1	interferon regulatory factor 2 binding protein 1	Nucleus	transcription regulator	
NUP160	NUP160	nucleoporin 160	Nucleus	other	
HNRNPDL	HNRNPDL	heterogeneous nuclear ribonucleoprotein D like	Nucleus	other	
EMD	EMD	emerin	Nucleus	other	

PUS7	PUS7	pseudouridine synthase 7	Nucleus	enzyme	
XPO1	XPO1	exportin 1	Nucleus	transporter	bortezomib/dexamethasone/selinexor, WJ01024, selinexor, felezonexor, eltanexor, dexamethasone/selinexor,
DDX6	DDX6	DEAD-box helicase 6	Nucleus	enzyme	
OS9	OS9	OS9 endoplasmic reticulum lectin	Nucleus	other	
HNRNPAB	HNRNPAB	heterogeneous nuclear ribonucleoprotein A/B	Nucleus	enzyme	
FEN1	FEN1	flap structure-specific endonuclease 1	Nucleus	enzyme	
SF3B1	SF3B1	splicing factor 3b subunit 1	Nucleus	other	H3B-8800
IPO7	IPO7	importin 7	Nucleus	transporter	
SNRPN	SNRPN	small nuclear ribonucleoprotein polypeptide N	Nucleus	other	
GLYR1	GLYR1	glyoxylate reductase 1 homolog	Nucleus	other	
SMU1	SMU1	SMU1 DNA replication regulator and spliceosomal factor	Nucleus	other	
CHD4	CHD4	chromodomain helicase DNA binding protein 4	Nucleus	enzyme	
RELA	RELA	RELA proto-oncogene, NF-kB subunit	Nucleus	transcription regulator	NF-kappaB decoy
DUT	DUT	deoxyuridine triphosphatase	Nucleus	enzyme	TAS-114
KHSRP	KHSRP	KH-type splicing regulatory protein	Nucleus	enzyme	
FBL	FBL	fibrillarin	Nucleus	enzyme	
RPL13	RPL13	ribosomal protein L13	Nucleus	other	
RRP12	RRP12	ribosomal RNA processing 12 homolog	Nucleus	other	
NOL9	NOL9	nucleolar protein 9	Nucleus	kinase	
SRSF9	SRSF9	serine and arginine rich splicing factor 9	Nucleus	enzyme	
ZFR	ZFR	zinc finger RNA binding protein	Nucleus	other	
MCM6	MCM6	minichromosome maintenance complex component 6	Nucleus	enzyme	
POLR2C	POLR2C	RNA polymerase II subunit C	Nucleus	enzyme	
CUX1	CUX1	cut like homeobox 1	Nucleus	transcription regulator	
SREK1	SREK1	splicing regulatory glutamic acid and lysine rich protein 1	Nucleus	other	
CBX5	CBX5	chromobox 5	Nucleus	transcription regulator	
ZNF326	ZNF326	zinc finger protein 326	Nucleus	transcription regulator	
CDC5L	CDC5L	cell division cycle 5 like	Nucleus	transcription regulator	
LMNA	LMNA	lamin A/C	Nucleus	other	
KPNB1	KPNB1	karyopherin subunit beta 1	Nucleus	other	
KHDRBS1	KHDRBS1	KH RNA binding domain containing, signal transduction associated 1	Nucleus	transcription regulator	CWP232291
KPNA6	KPNA6	karyopherin subunit alpha 6	Nucleus	other	
ANP32B	ANP32B	acidic nuclear phosphoprotein 32 family member B	Nucleus	other	
NUP62	NUP62	nucleoporin 62	Nucleus	transporter	dusquetide
HNRNPL	HNRNPL	heterogeneous nuclear ribonucleoprotein L	Nucleus	other	
NUDCD1	NUDCD1	NudC domain containing 1	Nucleus	other	
PRKAG1	PRKAG1	protein kinase AMP-activated non-catalytic subunit gamma 1	Nucleus	kinase	
RANGAP1	RANGAP1	Ran GTPase activating protein 1	Nucleus	other	
DCAF8	DCAF8	DDB1 and CUL4 associated factor 8	Nucleus	other	
H2AFY	MACROH2A1	macroH2A.1 histone	Nucleus	other	
UTP20	UTP20	UTP20 small subunit processome component	Nucleus	other	
U2SURP	U2SURP	U2 snRNP associated SURP domain containing	Nucleus	other	
G3BP1	G3BP1	G3BP stress granule assembly factor 1	Nucleus	enzyme	
NUP155	NUP155	nucleoporin 155	Nucleus	other	
RBM15	RBM15	RBM15 RNA binding motif protein 15	Nucleus	other	
TTC37	SKIC3	SKI3 subunit of superkiller complex	Nucleus	other	

ANP32E	ANP32E	acidic nuclear phosphoprotein 32 family member E	Nucleus	other	
PUF60	PUF60	poly(U) binding splicing factor 60	Nucleus	other	
NASP	NASP	nuclear autoantigenic sperm protein	Nucleus	other	
KPNA2	KPNA2	karyopherin subunit alpha 2	Nucleus	other	
SMC3	SMC3	structural maintenance of chromosomes 3	Nucleus	enzyme	
KPNA3	KPNA3	karyopherin subunit alpha 3	Nucleus	transporter	
BCLAF1	BCLAF1	BCL2 associated transcription factor 1	Nucleus	transcription regulator	
TRA2B	TRA2B	transformer 2 beta homolog	Nucleus	other	
PPIL4	PPIL4	peptidylprolyl isomerase like 4	Nucleus	enzyme	
MSH6	MSH6	mutS homolog 6	Nucleus	enzyme	
SRSF6	SRSF6	serine and arginine rich splicing factor 6	Nucleus	other	
NELFE	NELFE	negative elongation factor complex member E	Nucleus	other	
FAM98B	FAM98B	family with sequence similarity 98 member B	Nucleus	enzyme	
USP7	USP7	ubiquitin specific peptidase 7	Nucleus	peptidase	
HNRNPR	HNRNPR	heterogeneous nuclear ribonucleoprotein R	Nucleus	other	
NFKB2	NFKB2	nuclear factor kappa B subunit 2	Nucleus	transcription regulator	
ADI1	ADI1	acireductone dioxygenase 1	Nucleus	enzyme	
SON	SON	SON DNA and RNA binding protein	Nucleus	other	
NPM1	NPM1	nucleophosmin 1	Nucleus	transcription regulator	
PTMS	PTMS	parathymosin	Nucleus	other	
NUP88	NUP88	nucleoporin 88	Nucleus	other	
UBXN7	UBXN7	UBX domain protein 7	Nucleus	other	
DIDO1	DIDO1	death inducer-obliterator 1	Nucleus	other	
DHX15	DHX15	DEAH-box helicase 15	Nucleus	enzyme	
FUBP1	FUBP1	far upstream element binding protein 1	Nucleus	transcription regulator	
ASAP2	ASAP2	ArfGAP with SH3 domain, ankyrin repeat and PH domain 2	Nucleus	other	
LSM3	LSM3	LSM3 homolog, U6 small nuclear RNA and mRNA degradation associated	Nucleus	other	
SSRP1	SSRP1	structure specific recognition protein 1	Nucleus	transcription regulator	
TOR1AIP1	TOR1AIP1	torsin 1A interacting protein 1	Nucleus	other	
CSRP1	CSRP1	cysteine and glycine rich protein 1	Nucleus	other	
DDX39A	DDX39A	DExD-box helicase 39A	Nucleus	enzyme	
HNRNPA0	HNRNPA0	heterogeneous nuclear ribonucleoprotein A0	Nucleus	other	
PCBP1	PCBP1	poly(rC) binding protein 1	Nucleus	translation regulator	
PARK7	PARK7	Parkinsonism associated deglycase	Nucleus	enzyme	
PSMC4	PSMC4	proteasome 26S subunit, ATPase 4	Nucleus	peptidase	
NHP2L1	SNU13	small nuclear ribonucleoprotein 13	Nucleus	other	
RANBP1	RANBP1	RAN binding protein 1	Nucleus	other	
NELFCD	NELFCD	negative elongation factor complex member C/D	Nucleus	other	
CTNNB1	CTNNB1	catenin beta 1	Nucleus	transcription regulator	PRI-724, E7386
CNOT7	CNOT7	CCR4-NOT transcription complex subunit 7	Nucleus	transcription regulator	
PSIP1	PSIP1	PC4 and SRSF1 interacting protein 1	Nucleus	transcription regulator	
IPO5	IPO5	importin 5	Nucleus	transporter	
BCAS2	BCAS2	BCAS2 pre-mRNA processing factor	Nucleus	other	
PRMT1	PRMT1	protein arginine methyltransferase 1	Nucleus	enzyme	PRMT1 inhibitor,
SRRT	SRRT	serrate, RNA effector molecule	Nucleus	other	
SF3B2	SF3B2	splicing factor 3b subunit 2	Nucleus	other	
XPOT	XPOT	exportin for tRNA	Nucleus	other	
APEX1	APEX1	apurinic/apyrimidinic endodeoxyribonuclease 1	Nucleus	enzyme	lucanthone, E 3330
UBE2L3	UBE2L3	ubiquitin conjugating enzyme E2 L3	Nucleus	enzyme	

NAA15	NAA15	N-alpha-acetyltransferase 15, NatA auxiliary subunit	Nucleus	transcription regulator	
CLIC1	CLIC1	chloride intracellular channel 1	Nucleus	ion channel	
STAT1	STAT1	signal transducer and activator of transcription 1	Nucleus	transcription regulator	
BIN1	BIN1	bridging integrator 1	Nucleus	other	
SRRM1	SRRM1	serine and arginine repetitive matrix 1	Nucleus	other	
NAP1L1	NAP1L1	nucleosome assembly protein 1 like 1	Nucleus	other	
BUB3	BUB3	BUB3 mitotic checkpoint protein	Nucleus	other	
COPS6	COPS6	COP9 signalosome subunit 6	Nucleus	other	
ASNA1	GET3	guided entry of tail-anchored proteins factor 3, ATPase	Nucleus	transporter	
KPNA4	KPNA4	karyopherin subunit alpha 4	Nucleus	other	
WDR61	SKIC8	SKI8 subunit of superkiller complex	Nucleus	other	
HNRNPD	HNRNPD	heterogeneous nuclear ribonucleoprotein D	Nucleus	transcription regulator	
U2AF2	U2AF2	U2 small nuclear RNA auxiliary factor 2	Nucleus	other	
BPNT1	BPNT1	3'(2'), 5'-bisphosphate nucleotidase 1	Nucleus	phosphatase	
EMC2	EMC2	ER membrane protein complex subunit 2	Nucleus	other	
SRSF7	SRSF7	serine and arginine rich splicing factor 7	Nucleus	other	
HNRNPA2B1	HNRNPA2B1	heterogeneous nuclear ribonucleoprotein A2/B1	Nucleus	other	
UTP18	UTP18	UTP18 small subunit processome component	Nucleus	other	
ZC3HC1	ZC3HC1	zinc finger C3HC-type containing 1	Nucleus	other	
SF3A1	SF3A1	splicing factor 3a subunit 1	Nucleus	other	
PRPF31	PRPF31	pre-mRNA processing factor 31	Nucleus	other	
CFL1	CFL1	cofilin 1	Nucleus	other	
CPSF6	CPSF6	cleavage and polyadenylation specific factor 6	Nucleus	other	
TCEB1	ELOC	elongin C	Nucleus	transcription regulator	
TNKS1BP1	TNKS1BP1	tankyrase 1 binding protein 1	Nucleus	other	
SYNCRIP	SYNCRIP	synaptotagmin binding cytoplasmic RNA interacting protein	Nucleus	other	supinoxin
DNAJC8	DNAJC8	DnaJ heat shock protein family (Hsp40) member C8	Nucleus	other	
MTAP	MTAP	methylthioadenosine phosphorylase	Nucleus	enzyme	
SART1	SART1	spliceosome associated factor 1, recruiter of U4/U6.U5 tri-snRNP	Nucleus	other	
LSM8	LSM8	LSM8 homolog, U6 small nuclear RNA associated	Nucleus	other	
CDK5RAP3	CDK5RAP3	CDK5 regulatory subunit associated protein 3	Nucleus	other	
NPLOC4	NPLOC4	NPL4 homolog, ubiquitin recognition factor	Nucleus	other	
TRIM28	TRIM28	tripartite motif containing 28	Nucleus	transcription regulator	
NUP107	NUP107	nucleoporin 107	Nucleus	other	
RPA3	RPA3	replication protein A3	Nucleus	other	
LUC7L3	LUC7L3	LUC7 like 3 pre-mRNA splicing factor	Nucleus	other	
H2AFV	H2AZ2	H2A.Z variant histone 2	Nucleus	other	
SBDS	SBDS	SBDS ribosome maturation factor	Nucleus	other	
PCBD1	PCBD1	pterin-4 alpha-carbinolamine dehydratase 1	Nucleus	transcription regulator	
DDX1	DDX1	DEAD-box helicase 1	Nucleus	enzyme	
RNPS1	RNPS1	RNA binding protein with serine rich domain 1	Nucleus	other	
SH3BGRL3	SH3BGRL3	SH3 domain binding glutamate rich protein like 3	Nucleus	other	
PSMC5	PSMC5	proteasome 26S subunit, ATPase 5	Nucleus	transcription regulator	
PURB	PURB	purine rich element binding protein B	Nucleus	transcription regulator	
GTF2I	GTF2I	general transcription factor IIi	Nucleus	transcription regulator	
BRD2	BRD2	bromodomain containing 2	Nucleus	kinase	JAB-8263, BI 894999
CTNND1	CTNND1	catenin delta 1	Nucleus	other	

ZC3H4	ZC3H4	zinc finger CCCH-type containing 4	Nucleus	transcription regulator	
XRCC5	XRCC5	X-ray repair cross complementing 5	Nucleus	enzyme	
RBM39	RBM39	RNA binding motif protein 39	Nucleus	transcription regulator	
NFKB1	NFKB1	nuclear factor kappa B subunit 1	Nucleus	transcription regulator	dexamethasone/thalidomide, bortezomib/dexamethasone/thalidomide, rituximab/thalidomide, bortezomib/thalidomide, prednisone/thalidomide, triflusal,
NUP205	NUP205	nucleoporin 205	Nucleus	other	
ZMPSTE24	ZMPSTE24	zinc metallopeptidase STE24	Nucleus	peptidase	
DDX39B	DDX39B	DExD-box helicase 39B	Nucleus	enzyme	
UBE3A	UBE3A	ubiquitin protein ligase E3A	Nucleus	enzyme	
SNRNP200	SNRNP200	small nuclear ribonucleoprotein U5 subunit 200	Nucleus	enzyme	
UBTF	UBTF	upstream binding transcription factor	Nucleus	transcription regulator	
PHB	PHB1	prohibitin 1	Nucleus	transcription regulator	prohibitin-targeting peptide 1
RSL1D1	RSL1D1	ribosomal L1 domain containing 1	Nucleus	other	
RBM25	RBM25	RNA binding motif protein 25	Nucleus	other	
SRRM2	SRRM2	serine/arginine repetitive matrix 2	Nucleus	other	
HNRNPA1	HNRNPA1	heterogeneous nuclear ribonucleoprotein A1	Nucleus	other	
TCOF1	TCOF1	treacle ribosome biogenesis factor 1	Nucleus	transcription regulator	
SF3B3	SF3B3	splicing factor 3b subunit 3	Nucleus	other	
NUDT21	NUDT21	nudix hydrolase 21	Nucleus	other	
PPP1R7	PPP1R7	protein phosphatase 1 regulatory subunit 7	Nucleus	phosphatase	
MYADM	MYADM	myeloid associated differentiation marker	Nucleus	other	
NBAS	NBAS	NBAS subunit of NRZ tethering complex	Nucleus	other	
AGFG1	AGFG1	ArfGAP with FG repeats 1	Nucleus	other	
SYNE2	SYNE2	spectrin repeat containing nuclear envelope protein 2	Nucleus	other	
NUP214	NUP214	nucleoporin 214	Nucleus	transporter	
DNAJB1	DNAJB1	DnaJ heat shock protein family (Hsp40) member B1	Nucleus	transcription regulator	
ILF2	ILF2	interleukin enhancer binding factor 2	Nucleus	transcription regulator	
ACTL6A	ACTL6A	actin like 6A	Nucleus	other	
SNRNP70	SNRNP70	small nuclear ribonucleoprotein U1 subunit 70	Nucleus	other	
PSMC2	PSMC2	proteasome 26S subunit, ATPase 2	Nucleus	peptidase	
THOC2	THOC2	THO complex subunit 2	Nucleus	other	
PRPF19	PRPF19	pre-mRNA processing factor 19	Nucleus	enzyme	
SMARCB1	SMARCB1	SWI/SNF related, matrix associated, actin dependent regulator of chromatin, subfamily b, member 1	Nucleus	transcription regulator	
USP24	USP24	ubiquitin specific peptidase 24	Nucleus	peptidase	
NUP153	NUP153	nucleoporin 153	Nucleus	other	
NOP2	NOP2	NOP2 nucleolar protein	Nucleus	other	
CASP8	CASP8	caspase 8	Nucleus	peptidase	
DHX30	DHX30	DExH-box helicase 30	Nucleus	enzyme	
CPSF7	CPSF7	cleavage and polyadenylation specific factor 7	Nucleus	other	
MRE11A	MRE11	MRE11 homolog, double strand break repair nuclease	Nucleus	enzyme	
ZW10	ZW10	zw10 kinetochore protein	Nucleus	other	
PBRM1	PBRM1	polybromo 1	Nucleus	other	
RNF20	RNF20	ring finger protein 20	Nucleus	enzyme	
TARS	TARS1	threonyl-tRNA synthetase 1	Nucleus	enzyme	

MTOR	MTOR	mechanistic target of rapamycin kinase	Nucleus	kinase	tacrolimus, imatinib/sirolimus, everolimus/fulvestrant, apitolisib, CLL442, everolimus/pasireotide, rapalog, corticosteroid/sirolimus, PF-4691502, PP-121, GNE-493, AZD8055, torin-2, dactolisib, PKI-179, onatasertib, samotolisib, SF2523, everolimus/letrozole, HEC68498, everolimus/prednisone, everolimus/gefitinib, PI-540, methotrexate/sirolimus/tacrolimus, PI-620, prednisone/tacrolimus, CZ415,
SNRPE	SNRPE	small nuclear ribonucleoprotein polypeptide E	Nucleus	other	
RPL7	RPL7	ribosomal protein L7	Nucleus	transcription regulator	
MYOF	MYOF	myoferlin	Nucleus	other	
LUC7L	LUC7L	LUC7 like	Nucleus	other	
SUB1	SUB1	SUB1 regulator of transcription	Nucleus	transcription regulator	
PRPF6	PRPF6	pre-mRNA processing factor 6	Nucleus	transcription regulator	
SF3A2	SF3A2	splicing factor 3a subunit 2	Nucleus	other	
PRUNE	PRUNE1	prune exopolyphosphatase 1	Nucleus	enzyme	
SUGT1	SUGT1	SGT1 homolog, MIS12 kinetochore complex assembly cochaperone	Nucleus	other	
SNRPD1	SNRPD1	small nuclear ribonucleoprotein D1 polypeptide	Nucleus	other	
PCNA	PCNA	proliferating cell nuclear antigen	Nucleus	enzyme	AOH1996
RBBP4	RBBP4	RB binding protein 4, chromatin remodeling factor	Nucleus	enzyme	
CTBP1	CTBP1	C-terminal binding protein 1	Nucleus	enzyme	
DNAJA1	DNAJA1	DnaJ heat shock protein family (Hsp40) member A1	Nucleus	other	
BABAM1	BABAM1	BRISC and BRCA1 A complex member 1	Nucleus	other	
PDS5B	PDS5B	PDS5 cohesin associated factor B	Nucleus	other	
MFSD10	MFSD10	major facilitator superfamily domain containing 10	Nucleus	transporter	
HNRNPUL1	HNRNPUL1	heterogeneous nuclear ribonucleoprotein U like 1	Nucleus	other	
ARFGAP2	ARFGAP2	ADP ribosylation factor GTPase activating protein 2	Nucleus	other	
NELFB	NELFB	negative elongation factor complex member B	Nucleus	other	
GID8	GID8	GID complex subunit 8 homolog	Nucleus	other	
TP53BP1	TP53BP1	tumor protein p53 binding protein 1	Nucleus	transcription regulator	
DDX46	DDX46	DEAD-box helicase 46	Nucleus	enzyme	
PFDN5	PFDN5	prefoldin subunit 5	Nucleus	transcription regulator	
ILF3	ILF3	interleukin enhancer binding factor 3	Nucleus	transcription regulator	
AIP	AIP	aryl hydrocarbon receptor interacting protein	Nucleus	transcription regulator	
DIS3	DIS3	DIS3 homolog, exosome endoribonuclease and 3'-5' exoribonuclease	Nucleus	enzyme	
HNRNPH3	HNRNPH3	heterogeneous nuclear ribonucleoprotein H3	Nucleus	other	

SKIV2L	SKIC2	SKI2 subunit of superkiller complex	Nucleus	enzyme	
KPNA1	KPNA1	karyopherin subunit alpha 1	Nucleus	transporter	
PSMC1	PSMC1	proteasome 26S subunit, ATPase 1	Nucleus	peptidase	
RAN	RAN	RAN, member RAS oncogene family	Nucleus	enzyme	
HMGA2	HMGA2	high mobility group AT-hook 2	Nucleus	enzyme	
INTS3	INTS3	integrator complex subunit 3	Nucleus	other	
PARP1	PARP1	poly(ADP-ribose) polymerase 1	Nucleus	enzyme	SC10914, talazoparib, olaparib, ABT-767, rucaparib, TQB3823, iniparib, RP12146, [18F]fluorthanatrace, AMXI-5001, simmiparib, IDX-1197, veliparib, poly ADP ribose polymerase 1 inhibitor, CEP-9722, niraparib, 2X-121, amelparib, senaparib, fluzoparib,
BAG6	BAG6	BAG cochaperone 6	Nucleus	enzyme	
NSUN2	NSUN2	NOP2/Sun RNA methyltransferase 2	Nucleus	enzyme	
HIST1H1B	H1-5	H1.5 linker histone, cluster member	Nucleus	other	
NUP98	NUP98	nucleoporin 98 and 96 precursor	Nucleus	enzyme	
PML	PML	PML nuclear body scaffold	Nucleus	transcription regulator	arsenic trioxide
CBFB	CBFB	core-binding factor subunit beta	Nucleus	transcription regulator	
COPS5	COPS5	COP9 signalosome subunit 5	Nucleus	transcription regulator	
CSTF2	CSTF2	cleavage stimulation factor subunit 2	Nucleus	other	
CPSF1	CPSF1	cleavage and polyadenylation specific factor 1	Nucleus	other	
UGDH	UGDH	UDP-glucose 6-dehydrogenase	Nucleus	enzyme	
NUP188	NUP188	nucleoporin 188	Nucleus	other	
SMARCD2	SMARCD2	SWI/SNF related, matrix associated, actin dependent regulator of chromatin, subfamily d, member 2	Nucleus	transcription regulator	
CMPK1	CMPK1	cytidine/uridine monophosphate kinase 1	Nucleus	kinase	
FIP1L1	FIP1L1	factor interacting with PAPOLA and CPSF1	Nucleus	other	
HNRNPM	HNRNPM	heterogeneous nuclear ribonucleoprotein M	Nucleus	other	
SRSF1	SRSF1	serine and arginine rich splicing factor 1	Nucleus	other	
RPA1	RPA1	replication protein A1	Nucleus	other	
TROVE2	RO60	Ro60, Y RNA binding protein	Nucleus	other	
PPIL2	PPIL2	peptidylprolyl isomerase like 2	Nucleus	enzyme	
DNPH1	DNPH1	2'-deoxynucleoside 5'-phosphate N-hydrolase 1	Nucleus	enzyme	
RBM47	RBM47	RNA binding motif protein 47	Nucleus	other	
NCBP1	NCBP1	nuclear cap binding protein subunit 1	Nucleus	other	
ADK	ADK	adenosine kinase	Nucleus	kinase	adenosine kinase inhibitor, pegintron/ribavirin, PEG-interferon alfa-2a/ribavirin, nitazoxanide/peginterferon alfa-2a/ribavirin, interferon

HDAC2	HDAC2	histone deacetylase 2	Nucleus	transcription regulator	theophylline, trametinib/vorinostat, HG146, belinostat, domatinostat, vorinostat, histone deacetylase class II inhibitors, pyroxamide, chidamide, bortezomib/vorinostat,
UBAP2L	UBAP2L	ubiquitin associated protein 2 like	Nucleus	other	
TRRAP	TRRAP	transformation/transcription domain associated protein	Nucleus	transcription regulator	
DR1	DR1	down-regulator of transcription 1	Nucleus	transcription regulator	
MSH2	MSH2	mutS homolog 2	Nucleus	enzyme	
POLR2H	POLR2H	RNA polymerase II, I and III subunit H	Nucleus	enzyme	
PA2G4	PA2G4	proliferation-associated 2G4	Nucleus	transcription regulator	
NEDD8	NEDD8	NEDD8 ubiquitin like modifier	Nucleus	enzyme	
THUMPD1	THUMPD1	THUMP domain containing 1	Nucleus	other	
ACIN1	ACIN1	apoptotic chromatin condensation inducer 1	Nucleus	enzyme	
SRSF2	SRSF2	serine and arginine rich splicing factor 2	Nucleus	transcription regulator	
YLPM1	YLPM1	YLP motif containing 1	Nucleus	transcription regulator	
IPO11	IPO11	importin 11	Nucleus	other	
NUP54	NUP54	nucleoporin 54	Nucleus	other	
EWSR1	EWSR1	EWS RNA binding protein 1	Nucleus	other	
CENPV	CENPV	centromere protein V	Nucleus	other	
CTBP2	CTBP2	C-terminal binding protein 2	Nucleus	transcription regulator	
OXSR1	OXSR1	oxidative stress responsive kinase 1	Nucleus	kinase	
CUL2	CUL2	cullin 2	Nucleus	enzyme	
PDS5A	PDS5A	PDS5 cohesin associated factor A	Nucleus	other	
DDX23	DDX23	DEAD-box helicase 23	Nucleus	enzyme	
HNRNPA3	HNRNPA3	heterogeneous nuclear ribonucleoprotein A3	Nucleus	other	
CUL4A	CUL4A	cullin 4A	Nucleus	enzyme	
SART3	SART3	spliceosome associated factor 3, U4/U6 recycling protein	Nucleus	other	
SNRPD3	SNRPD3	small nuclear ribonucleoprotein D3 polypeptide	Nucleus	other	
PRKAB1	PRKAB1	protein kinase AMP-activated non-catalytic subunit beta 1	Nucleus	kinase	
SF3B4	SF3B4	splicing factor 3b subunit 4	Nucleus	other	
PSMC3	PSMC3	proteasome 26S subunit, ATPase 3	Nucleus	enzyme	
CERS2	CERS2	ceramide synthase 2	Nucleus	transcription regulator	
NUMA1	NUMA1	nuclear mitotic apparatus protein 1	Nucleus	other	
SUPT6H	SUPT6H	SPT6 homolog, histone chaperone and transcription elongation factor	Nucleus	transcription regulator	
EXOSC6	EXOSC6	exosome component 6	Nucleus	other	
GMPS	GMPS	guanine monophosphate synthase	Nucleus	enzyme	glutamine amidotransferase inhibitor
HNRNPH2	HNRNPH2	heterogeneous nuclear ribonucleoprotein H2	Nucleus	other	
GATAD2B	GATAD2B	GATA zinc finger domain containing 2B	Nucleus	transcription regulator	
RANBP2	RANBP2	RAN binding protein 2	Nucleus	enzyme	
ANXA11	ANXA11	annexin A11	Nucleus	other	

HDAC6	HDAC6	histone deacetylase 6	Nucleus	transcription regulator	trametinib/vorinostat, KA2507, belinostat, HG146, vorinostat, ricolinostat, pyroxamide, bortezomib/vorinostat, JBI-802, tributyrin, purinostat
XPO5	XPO5	exportin 5	Nucleus	other	
TOP1	TOP1	DNA topoisomerase I	Nucleus	enzyme	bevacizumab/irinotecan/oxaliplatin, SN-38, bevacizumab/paclitaxel/topotecan, delimotecan, belotecan, bevacizumab/irinotecan, 5-fluorouracil/irinotecan/oxaliplatin, BAY 56-3722, capecitabine/cetuximab/irinotecan, cyclophosphamide/topotecan, capecitabine/irinotecan/oxaliplatin, cyclophosphamide/temozolomide/topotecan, cetuximab/gimeracil/irinotecan/oxonic acid/tegafur, Genz-644282, edotecarin, IDEC-132, beta-
SNRPD2	SNRPD2	small nuclear ribonucleoprotein D2 polypeptide	Nucleus	other	
CHORDC1	CHORDC1	cysteine and histidine rich domain containing 1	Other	other	
SEPHS1	SEPHS1	selenophosphate synthetase 1	Other	enzyme	
PPP4R1	PPP4R1	protein phosphatase 4 regulatory subunit 1	Other	phosphatase	
KTI12	KTI12	KTI12 chromatin associated homolog	Other	other	
DAZAP1	DAZAP1	DAZ associated protein 1	Other	other	
OTUD6B	OTUD6B	OTU deubiquitinase 6B	Other	peptidase	
PBDC1	PBDC1	polysaccharide biosynthesis domain containing 1	Other	other	
FAM114A2	FAM114A2	family with sequence similarity 114 member A2	Other	other	
ABRACL	ABRACL	ABRA C-terminal like	Other	other	
UFSP2	UFSP2	UFM1 specific peptidase 2	Other	peptidase	
PCYOX1L	PCYOX1L	prenylcysteine oxidase 1 like	Other	other	
PRPSAP2	PRPSAP2	phosphoribosyl pyrophosphate synthetase associated protein 2	Other	other	
LMF2	LMF2	lipase maturation factor 2	Other	other	
TRMT1L	TRMT1L	tRNA methyltransferase 1 like	Other	enzyme	
KLC4	KLC4	kinesin light chain 4	Other	other	
KDELC2	POGLUT3	protein O-glucosyltransferase 3	Other	enzyme	
PRRC1	PRRC1	proline rich coiled-coil 1	Other	other	
LRRC47	LRRC47	leucine rich repeat containing 47	Other	other	
CYP20A1	CYP20A1	cytochrome P450 family 20 subfamily A member 1	Other	enzyme	
SSSCA1	ZNRD2	zinc ribbon domain containing 2	Other	other	
CD44	CD44	CD44 molecule (Indian blood group)	Plasma Membrane	other	SPL-108, A6 peptide, anti-CD44v7 antibody, AMC303,
SLMAP	SLMAP	sarcolemma associated protein	Plasma Membrane	other	

FARP1	FARP1	FERM, ARH/RhoGEF and pleckstrin domain protein 1	Plasma Membrane	other	
OSBPL8	OSBPL8	oxysterol binding protein like 8	Plasma Membrane	transporter	
TNS3	TNS3	tensin 3	Plasma Membrane	phosphatase	
PPIL1	PPIL1	peptidylprolyl isomerase like 1	Plasma Membrane	enzyme	
EXOC3	EXOC3	exocyst complex component 3	Plasma Membrane	transporter	
EMC1	EMC1	ER membrane protein complex subunit 1	Plasma Membrane	other	
SPTBN1	SPTBN1	spectrin beta, non-erythrocytic 1	Plasma Membrane	other	
GNA11	GNA11	G protein subunit alpha 11	Plasma Membrane	enzyme	
ABHD12	ABHD12	abhydrolase domain containing 12, lysophospholipase	Plasma Membrane	enzyme	
GNG12	GNG12	G protein subunit gamma 12	Plasma Membrane	enzyme	
SLC9A3R1	NHERF1	NHERF family PDZ scaffold protein 1	Plasma Membrane	transporter	
MPC2	MPC2	mitochondrial pyruvate carrier 2	Plasma Membrane	transporter	
B2M	B2M	beta-2-microglobulin	Plasma Membrane	transmembrane receptor	4'-iodo-4'-deoxydoxorubi
NCSTN	NCSTN	nicastrin	Plasma Membrane	peptidase	
ITGA2	ITGA2	integrin subunit alpha 2	Plasma Membrane	transmembrane receptor	
VAT1	VAT1	vesicle amine transport 1	Plasma Membrane	transporter	
ANXA2	ANXA2	annexin A2	Plasma Membrane	other	
CTNNA1	CTNNA1	catenin alpha 1	Plasma Membrane	other	
AGRN	AGRN	agrin	Plasma Membrane	other	
CLIC4	CLIC4	chloride intracellular channel 4	Plasma Membrane	ion channel	
LRP1	LRP1	LDL receptor related protein 1	Plasma Membrane	transmembrane receptor	paclitaxel-angiopep-2 conjugate, serpin peptide
SEC61G	SEC61G	SEC61 translocon subunit gamma	Plasma Membrane	transporter	
SLC3A2	SLC3A2	solute carrier family 3 member 2	Plasma Membrane	transporter	
PLXNB2	PLXNB2	plexin B2	Plasma Membrane	transmembrane receptor	
TM9SF2	TM9SF2	transmembrane 9 superfamily member 2	Plasma Membrane	transporter	
ESYT2	ESYT2	extended synaptotagmin 2	Plasma Membrane	other	
JUP	JUP	junction plakoglobin	Plasma Membrane	other	
PTGFRN	PTGFRN	prostaglandin F2 receptor inhibitor	Plasma Membrane	other	
GNB1	GNB1	G protein subunit beta 1	Plasma Membrane	other	
MBOAT7	MBOAT7	membrane bound O-acyltransferase domain containing 7	Plasma Membrane	enzyme	
DOCK7	DOCK7	dedicator of cytokinesis 7	Plasma Membrane	other	
CTTN	CTTN	cortactin	Plasma Membrane	other	
ACTR2	ACTR2	actin related protein 2	Plasma Membrane	other	
ACTR3	ACTR3	actin related protein 3	Plasma Membrane	other	
STT3A	STT3A	STT3 oligosaccharyltransferase complex catalytic subunit A	Plasma Membrane	enzyme	
DSP	DSP	desmoplakin	Plasma Membrane	other	
LAMP1	LAMP1	lysosomal associated membrane protein 1	Plasma Membrane	other	SAR428926
ITGB4	ITGB4	integrin subunit beta 4	Plasma Membrane	transmembrane receptor	
NCEH1	NCEH1	neutral cholesterol ester hydrolase 1	Plasma Membrane	enzyme	
TECR	TECR	trans-2,3-enoyl-CoA reductase	Plasma Membrane	enzyme	

SLC12A2	SLC12A2	solute carrier family 12 member 2	Plasma Membrane	transporter	bumetanide, quinethazone
FLOT2	FLOT2	flotillin 2	Plasma Membrane	other	
ILK	ILK	integrin linked kinase	Plasma Membrane	kinase	OSU-T315, KP-SD-1
PLS1	PLS1	plastin 1	Plasma Membrane	other	
LGALS3BP	LGALS3BP	galectin 3 binding protein	Plasma Membrane	transmembrane receptor	
EXOC8	EXOC8	exocyst complex component 8	Plasma Membrane	other	
CAP1	CAP1	cyclase associated actin cytoskeleton regulatory protein 1	Plasma Membrane	other	
STX7	STX7	syntaxin 7	Plasma Membrane	transporter	
CD9	CD9	CD9 molecule	Plasma Membrane	other	KBA1412
GNB2	GNB2	G protein subunit beta 2	Plasma Membrane	other	
NCKAP1	NCKAP1	NCK associated protein 1	Plasma Membrane	other	
NUDT2	NUDT2	nudix hydrolase 2	Plasma Membrane	phosphatase	
STXBP2	STXBP2	syntaxin binding protein 2	Plasma Membrane	transporter	
PGRMC1	PGRMC1	progesterone receptor membrane component 1	Plasma Membrane	transmembrane receptor	CT1812
SDCBP	SDCBP	syndecan binding protein	Plasma Membrane	enzyme	
FLOT1	FLOT1	flotillin 1	Plasma Membrane	other	
HNRNPLL	HNRNPLL	heterogeneous nuclear ribonucleoprotein L like	Plasma Membrane	other	
VTI1B	VTI1B	vesicle transport through interaction with t-SNAREs 1B	Plasma Membrane	transporter	
PNN	PNN	pinin, desmosome associated protein	Plasma Membrane	other	
ATP1B3	ATP1B3	ATPase Na+/K+ transporting subunit beta 3	Plasma Membrane	transporter	
STXBP3	STXBP3	syntaxin binding protein 3	Plasma Membrane	transporter	
APP	APP	amyloid beta precursor protein	Plasma Membrane	other	bapineuzumab, itanapraced, florbetapir F18, aducanumab, florbetaben F, lecanemab, flutemetamol
ATP2B1	ATP2B1	ATPase plasma membrane Ca2+ transporting 1	Plasma Membrane	transporter	
WASF2	WASF2	WASP family member 2	Plasma Membrane	other	
TLN1	TLN1	talin 1	Plasma Membrane	other	
ARF6	ARF6	ADP ribosylation factor 6	Plasma Membrane	transporter	
ITM2B	ITM2B	integral membrane protein 2B	Plasma Membrane	other	
UTRN	UTRN	utrophin	Plasma Membrane	transmembrane receptor	
CLTA	CLTA	clathrin light chain A	Plasma Membrane	other	
RHOC	RHOC	ras homolog family member C	Plasma Membrane	enzyme	
GNAS	GNAS	GNAS complex locus	Plasma Membrane	enzyme	
DNAJC5	DNAJC5	DnaJ heat shock protein family (Hsp40) member C5	Plasma Membrane	other	
ATAD1	ATAD1	ATPase family AAA domain containing 1	Plasma Membrane	enzyme	
PLIN2	PLIN2	perilipin 2	Plasma Membrane	other	
ALCAM	ALCAM	activated leukocyte cell adhesion molecule	Plasma Membrane	other	praluzatamab ravtansine
EZR	EZR	ezrin	Plasma Membrane	other	
TMEM14C	TMEM14C	transmembrane protein 14C	Plasma Membrane	other	
AP3B1	AP3B1	adaptor related protein complex 3 subunit beta 1	Plasma Membrane	transporter	

ATP1A1	ATP1A1	ATPase Na+/K+ transporting subunit alpha 1	Plasma Membrane	transporter	trichloromethiazide, perphenazine, ethacrynic acid, ciclopirox olamine, bretylium, reserpine/trichloromethiazide, ouabain, acetyldigitoxin derivative,
LANCL2	LANCL2	LanC like glutathione S-transferase 2	Plasma Membrane	other	
ITGB1	ITGB1	integrin subunit beta 1	Plasma Membrane	transmembrane receptor	OS2966
OSBPL3	OSBPL3	oxysterol binding protein like 3	Plasma Membrane	transporter	
ATP6V1A	ATP6V1A	ATPase H+ transporting V1 subunit A	Plasma Membrane	transporter	bafilomycin A1, bafilomycin b1
ANO10	ANO10	anoctamin 10	Plasma Membrane	ion channel	
ITGA6	ITGA6	integrin subunit alpha 6	Plasma Membrane	transmembrane receptor	
AP2B1	AP2B1	adaptor related protein complex 2 subunit beta 1	Plasma Membrane	transporter	
DAG1	DAG1	dystroglycan 1	Plasma Membrane	transmembrane receptor	
GNA13	GNA13	G protein subunit alpha 13	Plasma Membrane	enzyme	
SLC2A1	SLC2A1	solute carrier family 2 member 1	Plasma Membrane	transporter	canakinumab/metformin/sulfonylurea, insulin glargine/lixisenatide/metformin, metformin/vildagliptin, metformin/rosiglitazone/sulfonylurea, metformin/sulfonylurea/vildagliptin, metformin, metformin/pioglitazone/sulfonylurea, INS/metformin/sitagliptin, linagliptin/metformin, INS/metformin/pioglitazone, alogliptin/metformin/pioglitazone, metformin/sitagliptin/sulfonylurea, exenatide/metf
BSG	BSG	basigin (Ok blood group)	Plasma Membrane	transporter	metuximab
EHD4	EHD4	EH domain containing 4	Plasma Membrane	enzyme	
DNM2	DNM2	dynamin 2	Plasma Membrane	enzyme	
CNIH4	CNIH4	cornichon family AMPA receptor auxiliary protein 4	Plasma Membrane	other	
FKBP15	FKBP15	FKBP prolyl isomerase family member 15	Plasma Membrane	enzyme	
VASP	VASP	vasodilator stimulated phosphoprotein	Plasma Membrane	other	

CD47	CD47	CD47 molecule	Plasma Membrane	transmembrane receptor	lemzoparlimab, magrolimab, TTI-622, BAT7104, AO-176, PT886, ALX148, gentulizumab, VT1021, CPO107, PF-07257876, IBI322, anti-CD47 monoclonal antibody, CC-90002, MIL95, ZL-1201, NI-1801, TG-1801, STI-6643, IMM2902, 6MW3211, IMC-002, SGN-CD47M, recombinant fusion protein
ABCC1	ABCC1	ATP binding cassette subfamily C member 1	Plasma Membrane	transporter	sulfinpyrazone
IGF2R	IGF2R	insulin like growth factor 2 receptor	Plasma Membrane	transmembrane receptor	avalglucosidase alfa
ADPGK	ADPGK	ADP dependent glucokinase	Plasma Membrane	kinase	
ANXA7	ANXA7	annexin A7	Plasma Membrane	ion channel	
KDSR	KDSR	3-ketodihydrosphingosine reductase	Plasma Membrane	enzyme	
SLC16A1	SLC16A1	solute carrier family 16 member 1	Plasma Membrane	transporter	AZD-3965
EPHB4	EPHB4	EPH receptor B4	Plasma Membrane	kinase	tesevatinib, JI 101, AZ12672857,
ARL8B	ARL8B	ADP ribosylation factor like GTPase 8B	Plasma Membrane	enzyme	
CNPY2	CNPY2	canopy FGF signaling regulator 2	Plasma Membrane	other	
VAMP8	VAMP8	vesicle associated membrane protein 8	Plasma Membrane	transporter	
TSTA3	GFUS	GDP-L-fucose synthase	Plasma Membrane	enzyme	
RAC1	RAC1	Rac family small GTPase 1	Plasma Membrane	enzyme	
ANXA1	ANXA1	annexin A1	Plasma Membrane	enzyme	hydrocortisone, hydrocortisone/prednisone, hydrocortisone/mitoxantrone, cytarabine/hydrocortisone/methotrexate, dasatinib/hydrocortisone, hydrocortisone/imatinib, acyclovir/hydrocortisone
GNAI2	GNAI2	G protein subunit alpha i2	Plasma Membrane	enzyme	
SLC39A7	SLC39A7	solute carrier family 39 member 7	Plasma Membrane	transporter	
ATP1B1	ATP1B1	ATPase Na+/K+ transporting subunit beta 1	Plasma Membrane	transporter	
GRIPAP1	GRIPAP1	GRIP1 associated protein 1	Plasma Membrane	other	
CLPTM1	CLPTM1	CLPTM1 regulator of GABA type A receptor forward trafficking	Plasma Membrane	other	
STIM1	STIM1	stromal interaction molecule 1	Plasma Membrane	ion channel	
APMAP	APMAP	adipocyte plasma membrane associated protein	Plasma Membrane	enzyme	
SPTAN1	SPTAN1	spectrin alpha, non-erythrocytic 1	Plasma Membrane	other	
TMEM165	TMEM165	transmembrane protein 165	Plasma Membrane	other	
USP9X	USP9X	ubiquitin specific peptidase 9 X-linked	Plasma Membrane	peptidase	

VAPA	VAPA	VAMP associated protein A	Plasma Membrane	other	
VAPB	VAPB	VAMP associated protein B and C	Plasma Membrane	other	
ZYX	ZYX	zyxin	Plasma Membrane	other	
ITGAV	ITGAV	integrin subunit alpha V	Plasma Membrane	transmembrane receptor	abciximab, intetumumab, fluciclatide F 18, etaracizumab, abituzumab, 68Ga-NOTA-
ADAM10	ADAM10	ADAM metallopeptidase domain 10	Plasma Membrane	peptidase	aderbasib
STRAP	STRAP	serine/threonine kinase receptor associated protein	Plasma Membrane	other	
TJP1	TJP1	tight junction protein 1	Plasma Membrane	other	
TFRC	TFRC	transferrin receptor	Plasma Membrane	transporter	CALAA-01, CX-2029, oxaliplatin-encapsulated transferrin-conjugated N-glutaryl phosphatidylethanolamine liposome, monoclonal
TES	TES	testin LIM domain protein	Plasma Membrane	other	
TBL2	TBL2	transducin beta like 2	Plasma Membrane	other	
AIMP2	AIMP2	aminoacyl tRNA synthetase complex interacting multifunctional protein 2	Plasma Membrane	other	
CLTC	CLTC	clathrin heavy chain	Plasma Membrane	other	
ANXA5	ANXA5	annexin A5	Plasma Membrane	transporter	
MARCKS	MARCKS	myristoylated alanine rich protein kinase C substrate	Plasma Membrane	other	BIO-11006
SLC7A5	SLC7A5	solute carrier family 7 member 5	Plasma Membrane	transporter	QBS10072S
SNAP23	SNAP23	synaptosome associated protein 23	Plasma Membrane	transporter	
CDH1	CDH1	cadherin 1	Plasma Membrane	other	
GNAQ	GNAQ	G protein subunit alpha q	Plasma Membrane	enzyme	
LAMTOR1	LAMTOR1	late endosomal/lysosomal adaptor, MAPK and MTOR activator 1	Plasma Membrane	other	
SCARB2	SCARB2	scavenger receptor class B member 2	Plasma Membrane	transmembrane receptor	
GBAS	NIPSNAP2	nipsnap homolog 2	Plasma Membrane	other	
VAMP3	VAMP3	vesicle associated membrane protein 3	Plasma Membrane	other	
CD81	CD81	CD81 molecule	Plasma Membrane	other	
TCIRG1	TCIRG1	T cell immune regulator 1, ATPase H+ transporting V0 subunit a3	Plasma Membrane	enzyme	
STOML2	STOML2	stomatin like 2	Plasma Membrane	other	
VCL	VCL	vinculin	Plasma Membrane	other	
MLEC	MLEC	malectin	Plasma Membrane	other	
FERMT1	FERMT1	FERM domain containing kindlin 1	Plasma Membrane	other	
PPFIA1	PPFIA1	PTPRF interacting protein alpha 1	Plasma Membrane	phosphatase	
TJP2	TJP2	tight junction protein 2	Plasma Membrane	kinase	
CGN	CGN	cingulin	Plasma Membrane	other	
LPCAT3	LPCAT3	lysophosphatidylcholine acyltransferase 3	Plasma Membrane	enzyme	
CTNNA2	CTNNA2	catenin alpha 2	Plasma Membrane	other	

EPS15L1	EPS15L1	epidermal growth factor receptor pathway substrate 15 like 1	Plasma Membrane	other
ANXA4	ANXA4	annexin A4	Plasma Membrane	other
LRPAP1	LRPAP1	LDL receptor related protein associated protein 1	Plasma Membrane	other
ANO6	ANO6	anoctamin 6	Plasma Membrane	ion channel
MISP	MISP	mitotic spindle positioning	Plasma Membrane	other
STX4	STX4	syntaxin 4	Plasma Membrane	transporter
RAB8A	RAB8A	RAB8A, member RAS oncogene family	Plasma Membrane	enzyme

Data 2: Hub proteins

Gene	Betweenness Centrality	Description	Location	Family	Drugs
GAPDH	0,03	glyceraldehyde-3-phosphate dehydrogenase	Cytoplasm	enzyme	
ACTB	0,03	actin beta	Cytoplasm	other	
HSP90AA1	0,016	heat shock protein 90 alpha family class A member 1	Cytoplasm	enzyme	alvespimycin, retaspimycin, luminespib, pimitespib, cisplatin
HSPA8	0,02	heat shock protein family A (Hsp70) member 8	Cytoplasm	enzyme	SHetA2
HSP90AB1	0,01	heat shock protein 90 alpha family class B member 1	Cytoplasm	enzyme	alvespimycin, retaspimycin, pimitespib, cisplatin
HSPA4	0,01	heat shock protein family A (Hsp70) member 4	Cytoplasm	other	
EEF2	0,01	eukaryotic translation elongation factor 2	Cytoplasm	translation regulator	tagraxofusp, MDNA55
EFTUD2	0,01	elongation factor Tu GTP binding domain containing 2	Nucleus	enzyme	
RPS3	0,00	ribosomal protein S3	Cytoplasm	enzyme	
HNRNPA1	0,01	heterogeneous nuclear ribonucleoprotein A1	Nucleus	other	
RPS20	0,00	ribosomal protein S20	Cytoplasm	other	
RPS2	0,00	ribosomal protein S2	Cytoplasm	other	
NPM1	0,01	nucleophosmin 1	Nucleus	transcription regulator	
RPLP0	0,00	ribosomal protein lateral stalk subunit P0	Cytoplasm	other	
RPL4	0,00	ribosomal protein L4	Cytoplasm	enzyme	
RPSA	0,00	ribosomal protein SA	Cytoplasm	translation regulator	
RPS9	0,00	ribosomal protein S9	Cytoplasm	translation regulator	
RACK1	0,00	receptor for activated C kinase 1	Cytoplasm	enzyme	
RPS16	0,00	ribosomal protein S16	Cytoplasm	other	
EPRS1	0,01	glutamyl-prolyl-tRNA synthetase 1	Cytoplasm	enzyme	
EEF1A1	0,00	eukaryotic translation elongation factor 1 alpha 1	Cytoplasm	translation regulator	
VCP	0,01	valosin containing protein	Cytoplasm	enzyme	CB-5083, CB-5339
RPL5	0,00	ribosomal protein L5	Cytoplasm	other	
SRSF1	0,01	serine and arginine rich splicing factor 1	Nucleus	other	
RPL11	0,00	ribosomal protein L11	Cytoplasm	other	
RPL3	0,00	ribosomal protein L3	Nucleus	other	cytarabine/daunorubicin/omacetaxine mepesuccinate, omacetaxine mepesuccinate, cytarabine/doxorubicin/filgrastim/omacetaxine mepesuccinate
RPS11	0,00	ribosomal protein S11	Cytoplasm	other	
HSPA5	0,01	heat shock protein family A (Hsp70) member 5	Cytoplasm	enzyme	SHetA2, PAT-SM6
HSPA9	0,01	heat shock protein family A (Hsp70) member 9	Cytoplasm	other	SHetA2
RPL8	0,00	ribosomal protein L8	Cytoplasm	other	
ATP5F1A	0,00	ATP synthase F1 subunit alpha	Cytoplasm	transporter	
HNRNPC	0,00	heterogeneous nuclear ribonucleoprotein C	Nucleus	other	
CCT2	0,00	chaperonin containing TCP1 subunit 2	Cytoplasm	kinase	
RPS6	0,00	ribosomal protein S6	Cytoplasm	other	
ENO1	0,01	enolase 1	Cytoplasm	enzyme	
CCT7	0,00	chaperonin containing TCP1 subunit 7	Cytoplasm	other	
RPL9	0,00	ribosomal protein L9	Nucleus	other	
RPL23	0,00	ribosomal protein L23	Cytoplasm	other	
RPS14	0,00	ribosomal protein S14	Cytoplasm	translation regulator	
RPS5	0,00	ribosomal protein S5	Cytoplasm	other	
CTNNB1	0,01	catenin beta 1	Nucleus	transcription regulator	PRI-724, E7386
TARDBP	0,01	TAR DNA binding protein	Nucleus	transcription regulator	
TPI1	0,01	triosephosphate isomerase 1	Cytoplasm	enzyme	
PHB1	0,01	prohibitin 1	Nucleus	transcription regulator	prohibitin-targeting peptide 1
RPS13	0,00	ribosomal protein S13	Cytoplasm	other	
EIF4A3	0,00	eukaryotic translation initiation factor 4A3	Nucleus	enzyme	
EEF1G	0,00	eukaryotic translation elongation factor 1 gamma	Cytoplasm	translation regulator	

RPS3A	0,00	ribosomal protein S3A	Nucleus	other	
HNRNPA2B1	0,00	heterogeneous nuclear ribonucleoprotein A2/B1	Nucleus	other	
TCP1	0,00	t-complex 1	Cytoplasm	other	
EIF2S1	0,00	eukaryotic translation initiation factor 2 subunit alpha	Cytoplasm	translation regulator	
RPL7	0,00	ribosomal protein L7	Nucleus	transcription regulator	
RPL23A	0,00	ribosomal protein L23a	Cytoplasm	other	
CCT5	0,00	chaperonin containing TCP1 subunit 5	Cytoplasm	other	
RPS23	0,00	ribosomal protein S23	Cytoplasm	translation regulator	
PABPC1	0,00	poly(A) binding protein cytoplasmic 1	Cytoplasm	translation regulator	
HNRNPK	0,00	heterogeneous nuclear ribonucleoprotein K	Nucleus	other	
RPL12	0,00	ribosomal protein L12	Nucleus	other	
CCT4	0,00	chaperonin containing TCP1 subunit 4	Cytoplasm	other	
RPL6	0,00	ribosomal protein L6	Nucleus	other	
PKM	0,00	pyruvate kinase M1/2	Cytoplasm	kinase	TP-1454, CAP-232
SNRPD2	0,00	small nuclear ribonucleoprotein D2 polypeptide	Nucleus	other	
SNU13	0,00	small nuclear ribonucleoprotein 13	Nucleus	other	
YBX1	0,00	Y-box binding protein 1	Nucleus	transcription regulator	
CS	0,00	citrate synthase	Cytoplasm	enzyme	
H4C6	0,01	H4 clustered histone 6	Nucleus	other	
PA2G4	0,00	proliferation-associated 2G4	Nucleus	transcription regulator	
SDHA	0,00	succinate dehydrogenase complex flavoprotein subunit A	Cytoplasm	enzyme	
RPS8	0,00	ribosomal protein S8	Cytoplasm	other	
DHX9	0,00	DExH-box helicase 9	Nucleus	enzyme	
EIF4A1	0,00	eukaryotic translation initiation factor 4A1	Cytoplasm	translation regulator	
CCT8	0,00	chaperonin containing TCP1 subunit 8	Cytoplasm	other	
ATP5F1B	0,00	ATP synthase F1 subunit beta	Cytoplasm	transporter	
PSMD14	0,00	proteasome 26S subunit, non-ATPase 14	Cytoplasm	peptidase	
RPL7A	0,00	ribosomal protein L7a	Cytoplasm	other	
DDX39B	0,00	DExD-box helicase 39B	Nucleus	enzyme	
RPL26	0,00	ribosomal protein L26	Cytoplasm	other	
RBM39	0,00	RNA binding motif protein 39	Nucleus	transcription regulator	
GART	0,00	phosphoribosylglycinamide formyltransferase, phosphoribosylglycinamide synthetase, phosphoribosylaminoimidazole synthetase	Cytoplasm	enzyme	pemetrexed, bevacizumab/erlotinib/pemetrexed, pelitrexol, bevacizumab/pemetrexed, pembrolizumab/pemetrexed, pemetrexed polyglutamate, gemcitabine/pemetrexed, bevacizumab/paclitaxel/pemetrexed, lometrexol
TUFM	0,00	Tu translation elongation factor, mitochondrial	Cytoplasm	translation regulator	
LARP7	0,00	La ribonucleoprotein 7, transcriptional regulator	Nucleus	other	
SYNCRIP	0,00	synaptotagmin binding cytoplasmic RNA interacting protein	Nucleus	other	supinoxin
RPL19	0,00	ribosomal protein L19	Cytoplasm	other	
RPL27	0,00	ribosomal protein L27	Cytoplasm	other	
GFM1	0,00	G elongation factor mitochondrial 1	Cytoplasm	translation regulator	
CDC42	0,01	cell division cycle 42	Cytoplasm	enzyme	
ACO2	0,00	aconitase 2	Cytoplasm	enzyme	
RPS19	0,00	ribosomal protein S19	Cytoplasm	other	
EIF5B	0,00	eukaryotic translation initiation factor 5B	Cytoplasm	translation regulator	
EIF3B	0,00	eukaryotic translation initiation factor 3 subunit B	Cytoplasm	translation regulator	
RPL10A	0,00	ribosomal protein L10a	Nucleus	other	
RPL17	0,00	ribosomal protein L17	Cytoplasm	other	
ELAVL1	0,00	ELAV like RNA binding protein 1	Cytoplasm	other	
RPL18	0,00	ribosomal protein L18	Cytoplasm	other	
RPS15	0,00	ribosomal protein S15	Cytoplasm	other	

RUVBL1	0,00	RuvB like AAA ATPase 1	Nucleus	transcription regulator	
MRPS7	0,00	mitochondrial ribosomal protein S7	Cytoplasm	other	
RPS4X	0,00	ribosomal protein S4 X-linked	Cytoplasm	other	
EIF3I	0,00	eukaryotic translation initiation factor 3 subunit I	Cytoplasm	translation regulator	
FBL	0,00	fibrillarin	Nucleus	enzyme	
RPS12	0,00	ribosomal protein S12	Cytoplasm	other	neomycin
HSPD1	0,00	heat shock protein family D (Hsp60) member 1	Cytoplasm	enzyme	
RPL18A	0,00	ribosomal protein L18a	Cytoplasm	other	
PSMA3	0,00	proteasome 20S subunit alpha 3	Cytoplasm	peptidase	
NCL	0,01	nucleolin	Nucleus	other	AGRO 100, IPP-204106N
SRC	0,00	SRC proto-oncogene, non-receptor tyrosine kinase	Cytoplasm	kinase	bosutinib/corticosteroid, PP-121, tirbanibulin, CGP77675, WH-4-025, ZM 306416, SI163, corticosteroid/dasatinib, XL999, AZ12672857, PD173955, S7, AZM-475271, saracatinib, bosutinib/dexamethasone/vincristine, KX02, blinatumomab/dasatinib, bosutinib/rituximab, S29, WH-4-023, SC-204303, PD162531, dasatinib/dexamethasone/vincristine, SM1-71, CCT239065, dasatinib/dexamethasone, bosutinib, cytarabine/dasatinib/dexamethasone/methotrexate, PD 173958, dasatinib/rituximab, TPX-0046, bosutinib/imatinib, docetaxel/nintedanib, AZD0424, MO-26, nintedanib, pexmetinib, repotrectinib, dasatinib/inotuzumab ozogamicin, dasatinib
RPS28	0,00	ribosomal protein S28	Cytoplasm	other	
CCT3	0,00	chaperonin containing TCP1 subunit 3	Cytoplasm	other	
POLR2B	0,00	RNA polymerase II subunit B	Nucleus	enzyme	
RPL35	0,00	ribosomal protein L35	Cytoplasm	other	
RPL30	0,00	ribosomal protein L30	Cytoplasm	other	
RPL27A	0,00	ribosomal protein L27a	Cytoplasm	other	
RAN	0,00	RAN, member RAS oncogene family	Nucleus	enzyme	
VDAC1	0,01	voltage dependent anion channel 1	Cytoplasm	ion channel	
HNRNPH1	0,00	heterogeneous nuclear ribonucleoprotein H1	Nucleus	other	
SNRPE	0,00	small nuclear ribonucleoprotein polypeptide E	Nucleus	other	
SDHB	0,00	succinate dehydrogenase complex iron sulfur subunit B	Cytoplasm	enzyme	
RPS24	0,00	ribosomal protein S24	Cytoplasm	other	
ABCE1	0,00	ATP binding cassette subfamily E member 1	Cytoplasm	transporter	
RPL13	0,00	ribosomal protein L13	Nucleus	other	
HNRNPM	0,01	heterogeneous nuclear ribonucleoprotein M	Nucleus	other	
RPS15A	0,00	ribosomal protein S15a	Cytoplasm	other	
SEC61A1	0,00	SEC61 translocon subunit alpha 1	Cytoplasm	transporter	KZR-261
DHX15	0,00	DEAH-box helicase 15	Nucleus	enzyme	
HNRNPR	0,00	heterogeneous nuclear ribonucleoprotein R	Nucleus	other	
RPS7	0,00	ribosomal protein S7	Cytoplasm	other	
ATP5F1C	0,00	ATP synthase F1 subunit gamma	Cytoplasm	transporter	
PRPF8	0,00	pre-mRNA processing factor 8	Nucleus	other	
G3BP1	0,01	G3BP stress granule assembly factor 1	Nucleus	enzyme	
CDH1	0,00	cadherin 1	Plasma Membrane	other	
RHOA	0,01	ras homolog family member A	Cytoplasm	enzyme	
EIF3G	0,00	eukaryotic translation initiation factor 3 subunit G	Cytoplasm	translation regulator	
NCBP1	0,00	nuclear cap binding protein subunit 1	Nucleus	other	
KPNB1	0,00	karyopherin subunit beta 1	Nucleus	other	
RPL31	0,00	ribosomal protein L31	Cytoplasm	other	

ILF3	0,00	interleukin enhancer binding factor 3	Nucleus	transcription regulator	
RPL10	0,00	ribosomal protein L10	Cytoplasm	translation regulator	
RPL14	0,00	ribosomal protein L14	Cytoplasm	other	
NEDD8	0,00	NEDD8 ubiquitin like modifier	Nucleus	enzyme	
HSPE1	0,00	heat shock protein family E (Hsp10) member 1	Cytoplasm	enzyme	
CANX	0,01	calnexin	Cytoplasm	other	
PSMC5	0,00	proteasome 26S subunit, ATPase 5	Nucleus	transcription regulator	
SNRPF	0,00	small nuclear ribonucleoprotein polypeptide F	Nucleus	other	
PSMA2	0,00	proteasome 20S subunit alpha 2	Cytoplasm	peptidase	
HSP90B1	0,01	heat shock protein 90 beta family member 1	Cytoplasm	other	alvespimycin, retaspimycin, cisplatin
RPL35A	0,00	ribosomal protein L35a	Cytoplasm	other	
HNRNPU	0,00	heterogeneous nuclear ribonucleoprotein U	Nucleus	transporter	
CCT6A	0,00	chaperonin containing TCP1 subunit 6A	Cytoplasm	other	
MDH2	0,00	malate dehydrogenase 2	Cytoplasm	enzyme	
HDAC1	0,00	histone deacetylase 1	Nucleus	enzyme	pyroxamide, R 306465, bortezomib/vorinostat, OKI-179, tributyrin, purinostat, trametinib/vorinostat, belinostat, mocetinostat, HG146, HDAC class I inhibitors, vorinostat, domatinostat
PARK7	0,00	Parkinsonism associated deglycase	Nucleus	enzyme	
U2AF2	0,00	U2 small nuclear RNA auxiliary factor 2	Nucleus	other	
XPO1	0,00	exportin 1	Nucleus	transporter	bortezomib/dexamethasone/selinexor, WJ01024, selinexor, felezonexor, eltanexor, dexamethasone/selinexor, leptomycin B
NDUFS3	0,00	NADH:ubiquinone oxidoreductase core subunit S3	Cytoplasm	enzyme	
PGK1	0,00	phosphoglycerate kinase 1	Cytoplasm	kinase	CBR-470-1
DDX5	0,00	DEAD-box helicase 5	Nucleus	enzyme	
STAT3	0,01	signal transducer and activator of transcription 3	Nucleus	transcription regulator	CAS3/SS3, golotimod, OPB-31121, OPB-51602, danvatirsen, TTI-101, STAT3 inhibitor, NT219
RPL21	0,00	ribosomal protein L21	Cytoplasm	other	
RPS25	0,00	ribosomal protein S25	Cytoplasm	other	
SNRPD3	0,00	small nuclear ribonucleoprotein D3 polypeptide	Nucleus	other	
ATP5PO	0,00	ATP synthase peripheral stalk subunit OSCP	Cytoplasm	transporter	
FUS	0,00	FUS RNA binding protein	Nucleus	transcription regulator	
RPS26	0,00	ribosomal protein S26	Cytoplasm	other	
CLPP	0,00	caseinolytic mitochondrial matrix peptidase proteolytic subunit	Cytoplasm	peptidase	ONC206
EIF3F	0,00	eukaryotic translation initiation factor 3 subunit F	Cytoplasm	translation regulator	
POLR2C	0,00	RNA polymerase II subunit C	Nucleus	enzyme	
EIF5A	0,00	eukaryotic translation initiation factor 5A	Cytoplasm	translation regulator	SNS01-T nanoparticles
NDUFAB1	0,00	NADH:ubiquinone oxidoreductase subunit AB1	Cytoplasm	enzyme	
H6PD	0,00	hexose-6-phosphate dehydrogenase/glucose 1-dehydrogenase	Cytoplasm	enzyme	
NDUFS8	0,00	NADH:ubiquinone oxidoreductase core subunit S8	Cytoplasm	enzyme	
RPLP2	0,00	ribosomal protein lateral stalk subunit P2	Cytoplasm	other	
RPS10	0,00	ribosomal protein S10	Cytoplasm	other	
SRSF3	0,00	serine and arginine rich splicing factor 3	Nucleus	other	
HNRNPD	0,00	heterogeneous nuclear ribonucleoprotein D	Nucleus	transcription regulator	
SOD2	0,00	superoxide dismutase 2	Cytoplasm	enzyme	manganese
RPL34	0,00	ribosomal protein L34	Cytoplasm	other	
GSK3B	0,00	glycogen synthase kinase 3 beta	Nucleus	kinase	aloisine A, enzastaurin, GSK-3beta inhibitor II, indirubin-3'-monoxime, glycogen synthase kinase-3beta inhibitor, AZD2858
PSMC2	0,00	proteasome 26S subunit, ATPase 2	Nucleus	peptidase	

PCBP1	0,00	poly(rC) binding protein 1	Nucleus	translation regulator	
COPS5	0,00	COP9 signalosome subunit 5	Nucleus	transcription regulator	
RPS27	0,01	ribosomal protein S27	Cytoplasm	other	empesertib
PSMA7	0,00	proteasome 20S subunit alpha 7	Cytoplasm	peptidase	
ETF1	0,01	eukaryotic translation termination factor 1	Cytoplasm	translation regulator	
EIF4E	0,00	eukaryotic translation initiation factor 4E	Cytoplasm	translation regulator	ISIS 183750
EIF2S2	0,00	eukaryotic translation initiation factor 2 subunit beta	Cytoplasm	translation regulator	
TFRC	0,00	transferrin receptor	Plasma Membrane	transporter	CALAA-01, CX-2029, oxaliplatin-encapsulated transferrin-conjugated N-glutaryl phosphatidylethanolamine liposome, monoclonal antibody A27.15
PSMC4	0,00	proteasome 26S subunit, ATPase 4	Nucleus	peptidase	
PSMA6	0,00	proteasome 20S subunit alpha 6	Cytoplasm	peptidase	
NOP56	0,00	NOP56 ribonucleoprotein	Nucleus	other	
YARS1	0,00	tyrosyl-tRNA synthetase 1	Cytoplasm	enzyme	
MARS1	0,00	methionyl-tRNA synthetase 1	Cytoplasm	enzyme	
SOD1	0,00	superoxide dismutase 1	Cytoplasm	enzyme	tofersen
GRB2	0,00	growth factor receptor bound protein 2	Cytoplasm	other	liposome-incorporated Grb2 antisense oligodeoxynucleotide
RPL28	0,00	ribosomal protein L28	Cytoplasm	other	
MTOR	0,00	mechanistic target of rapamycin kinase	Nucleus	kinase	tacrolimus, imatinib/sirolimus, everolimus/fulvestrant, apitolisib, CLL442, everolimus/pasireotide, rapalog, corticosteroid/sirolimus, PF-4691502, PP-121, GNE-493, AZD8055, torin-2, dactolisib, PKI-179, onatasertib, samotolisib, SF2523, everolimus/letrozole, HEC68498, everolimus/prednisone, everolimus/gefitinib, PI-540, methotrexate/sirolimus/tacrolimus, PI-620, prednisone/tacrolimus, CZ415, methylprednisolone/tacrolimus, cyclosporine A/sirolimus/tacrolimus, PWT33597, mTOR inhibitor, everolimus/sorafenib, Ku-0063794, ETP-45658, WXFL10030390, bimiralisib, everolimus, pimecrolimus, panulisib, everolimus/exemestane, DS-7423, temsirolimus, PKI-402, OXA-01, gedatolisib, lenalidomide/temsirolimus, sirolimus, OSI-027, VS-5584, everolimus/tamoxifen, ridaforolimus, everolimus/lenvatinib, vistusertib, mTOR inhibitor/tyrosine kinase inhibitor, everolimus/paclitaxel, ME-344
RANBP2	0,00	RAN binding protein 2	Nucleus	enzyme	
SNRNP70	0,00	small nuclear ribonucleoprotein U1 subunit 70	Nucleus	other	
ILF2	0,00	interleukin enhancer binding factor 2	Nucleus	transcription regulator	
COX4I1	0,00	cytochrome c oxidase subunit 4I1	Cytoplasm	enzyme	
PSMD2	0,00	proteasome 26S subunit ubiquitin receptor, non-ATPase 2	Cytoplasm	other	bortezomib/cladribine/rituximab, bortezomib/dexamethasone/pomalidomide, bortezomib/doxorubicin, bortezomib/dexamethasone/thalidomide, bortezomib/paclitaxel, bortezomib/sorafenib, bortezomib/fulvestrant, bortezomib/rituximab, bortezomib/lenalidomide, bortezomib/dexamethasone/lenalidomide, bortezomib/dexamethasone/doxorubicin, bortezomib/dexamethasone, bortezomib/prednisone, bortezomib/vorinostat, bortezomib, bortezomib/thalidomide, bortezomib/dexamethasone/rituximab
PSMA5	0,00	proteasome 20S subunit alpha 5	Cytoplasm	peptidase	
SUCLG1	0,00	succinate-CoA ligase GDP/ADP-forming subunit alpha	Cytoplasm	enzyme	
PDHA1	0,00	pyruvate dehydrogenase E1 subunit alpha 1	Cytoplasm	enzyme	
HSPA1A/HSPA1B	0,00	heat shock protein family A (Hsp70) member 1A	Cytoplasm	enzyme	

PSMD1	0,01	proteasome 26S subunit, non-ATPase 1	Cytoplasm	other	bortezomib/cladribine/rituximab, bortezomib/dexamethasone/pomalidomide, bortezomib/doxorubicin, bortezomib/dexamethasone/thalidomide, bortezomib/paclitaxel, bortezomib/sorafenib, bortezomib/fulvestrant, bortezomib/rituximab, bortezomib/lenalidomide, bortezomib/dexamethasone/lenalidomide, bortezomib/dexamethasone/doxorubicin, bortezomib/dexamethasone, bortezomib/prednisone, bortezomib/vorinostat, bortezomib, bortezomib/thalidomide, bortezomib/dexamethasone/rituximab
RPS21	0,00	ribosomal protein S21	Cytoplasm	other	
APP	0,00	amyloid beta precursor protein	Plasma Membrane	other	bapineuzumab, itanapraced, florbetapir F18, aducanumab, florbetaben F, lecanemab, flutemetamol
PTBP1	0,00	polypyrimidine tract binding protein 1	Nucleus	enzyme	
CHD4	0,01	chromodomain helicase DNA binding protein 4	Nucleus	enzyme	
TRIM28	0,00	tripartite motif containing 28	Nucleus	transcription regulator	
ATP5F1D	0,00	ATP synthase F1 subunit delta	Cytoplasm	transporter	
CLTC	0,00	clathrin heavy chain	Plasma Membrane	other	
FH	0,00	fumarate hydratase	Cytoplasm	enzyme	
IDH1	0,00	isocitrate dehydrogenase (NADP(+)) 1	Cytoplasm	enzyme	olutasidenib, IDH305, ivosidenib, DS-1001, HMPL-306, BAY1436032, KY100001, IDH1 inhibitor, LY3410738
DDX3X	0,00	DEAD-box helicase 3 X-linked	Cytoplasm	enzyme	
MAPK3	0,01	mitogen-activated protein kinase 3	Cytoplasm	kinase	ulixertinib, HE3286, ASN007, ASTX029, HH2710, tyrphostin AG 1288, KO-947, ZSET 1446, SM1-71, temuterkib, Vx-11e, ATG-017, FR 180204, (Z)-3-(2-aminoethyl)-5-(4-ethoxybenzylidene)thiazolidine-2,4-dione, JSI-1187
EIF4A2	0,00	eukaryotic translation initiation factor 4A2	Cytoplasm	translation regulator	
EIF3A	0,00	eukaryotic translation initiation factor 3 subunit A	Cytoplasm	translation regulator	
EIF4G1	0,00	eukaryotic translation initiation factor 4 gamma 1	Cytoplasm	translation regulator	
SEC13	0,00	SEC13 homolog, nuclear pore and COPII coat complex component	Cytoplasm	transporter	
FN1	0,00	fibronectin 1	Extracellular Space	other	ocriplasmin, bifikafusp alfa, L19TNFalpha, L19-IL2 monoclonal antibody-cytokine fusion protein/L19TNFalpha, AS1409, iodine I 131 anti-fibronectin antibody fragment L19-SIP, dodekin
ALDH18A1	0,00	aldehyde dehydrogenase 18 family member A1	Cytoplasm	kinase	
MTREX	0,00	Mtr4 exosome RNA helicase	Nucleus	enzyme	
PSMC1	0,00	proteasome 26S subunit, ATPase 1	Nucleus	peptidase	
CDC5L	0,00	cell division cycle 5 like	Nucleus	transcription regulator	
YWHAZ	0,00	tyrosine 3-monooxygenase/tryptophan 5-monooxygenase activation protein zeta	Cytoplasm	enzyme	
RPL32	0,00	ribosomal protein L32	Cytoplasm	other	
SERBP1	0,00	SERPINE1 mRNA binding protein 1	Cytoplasm	translation regulator	
SF3B1	0,00	splicing factor 3b subunit 1	Nucleus	other	H3B-8800
UBQLN1	0,00	ubiquilin 1	Cytoplasm	other	
RPS17	0,00	ribosomal protein S17	Cytoplasm	other	
RPL38	0,00	ribosomal protein L38	Cytoplasm	other	
PSMA1	0,00	proteasome 20S subunit alpha 1	Cytoplasm	peptidase	
UPF1	0,00	UPF1 RNA helicase and ATPase	Nucleus	enzyme	
SNRPD1	0,00	small nuclear ribonucleoprotein D1 polypeptide	Nucleus	other	
RAD23B	0,00	RAD23 homolog B, nucleotide excision repair protein	Nucleus	other	
RUVBL2	0,00	RuvB like AAA ATPase 2	Nucleus	transcription regulator	

NFKB1	0,00	nuclear factor kappa B subunit 1	Nucleus	transcription regulator	dexamethasone/thalidomide, bortezomib/dexamethasone/thalidomide, rituximab/thalidomide, bortezomib/thalidomide, prednisone/thalidomide, triflusal, thalidomide
DLD	0,00	dihydrolipoamide dehydrogenase	Cytoplasm	enzyme	hexachlorophene
KARS1	0,00	lysyl-tRNA synthetase 1	Cytoplasm	enzyme	
TXN	0,00	thioredoxin	Cytoplasm	enzyme	PX-12
RPL24	0,00	ribosomal protein L24	Cytoplasm	other	
EIF6	0,00	eukaryotic translation initiation factor 6	Cytoplasm	translation regulator	
GMPS	0,00	guanine monophosphate synthase	Nucleus	enzyme	glutamine amidotransferase inhibitor
SUMO1	0,00	small ubiquitin like modifier 1	Nucleus	enzyme	
HNRNPL	0,00	heterogeneous nuclear ribonucleoprotein L	Nucleus	other	
CALR	0,00	calreticulin	Cytoplasm	transcription regulator	
DDX21	0,00	DExD-box helicase 21	Nucleus	enzyme	
NOP58	0,00	NOP58 ribonucleoprotein	Nucleus	enzyme	
BTF3	0,00	basic transcription factor 3	Nucleus	transcription regulator	
LOC102724594/U2AF1	0,00	U2 small nuclear RNA auxiliary factor 1	Nucleus	other	
MRPL24	0,00	mitochondrial ribosomal protein L24	Cytoplasm	other	
RPL22	0,00	ribosomal protein L22	Cytoplasm	translation regulator	
POLR1C	0,00	RNA polymerase I and III subunit C	Nucleus	enzyme	
SRSF2	0,00	serine and arginine rich splicing factor 2	Nucleus	transcription regulator	
IDH2	0,00	isocitrate dehydrogenase (NADP(+)) 2	Cytoplasm	enzyme	SH1573, enasidenib, TQB3455, HMPL-306
LDHA	0,00	lactate dehydrogenase A	Cytoplasm	enzyme	nedosiran
RPL37A	0,00	ribosomal protein L37a	Cytoplasm	other	
PSMA4	0,00	proteasome 20S subunit alpha 4	Cytoplasm	peptidase	
EIF3M	0,00	eukaryotic translation initiation factor 3 subunit M	Cytoplasm	translation regulator	
UQCRFS1	0,00	ubiquinol-cytochrome c reductase, Rieske iron-sulfur polypeptide 1	Cytoplasm	enzyme	
SDHC	0,00	succinate dehydrogenase complex subunit C	Cytoplasm	enzyme	
PSMD11	0,00	proteasome 26S subunit, non-ATPase 11	Cytoplasm	other	
EIF3E	0,00	eukaryotic translation initiation factor 3 subunit E	Cytoplasm	translation regulator	
GPI	0,00	glucose-6-phosphate isomerase	Extracellular Space	enzyme	
PDHB	0,00	pyruvate dehydrogenase E1 subunit beta	Cytoplasm	enzyme	
ACLY	0,00	ATP citrate lyase	Cytoplasm	enzyme	bempedoyl-CoA, bempedoic acid/ezetimibe, bempedoic acid
SRP54	0,00	signal recognition particle 54	Cytoplasm	enzyme	
QARS1	0,00	glutaminyl-tRNA synthetase 1	Cytoplasm	enzyme	
SNRPA	0,00	small nuclear ribonucleoprotein polypeptide A	Nucleus	other	
ARF1	0,00	ADP ribosylation factor 1	Cytoplasm	enzyme	
PARP1	0,00	poly(ADP-ribose) polymerase 1	Nucleus	enzyme	SC10914, talazoparib, olaparib, ABT-767, rucaparib, TQB3823, iniparib, RP12146, [18F]fluorthanatrace, AMXI-5001, simmiparib, IDX-1197, veliparib, poly ADP ribose polymerase 1 inhibitor, CEP-9722, niraparib, 2X-121, amelparib, senaparib, fluzoparib, AZD9574
KHDRBS1	0,00	KH RNA binding domain containing, signal transduction associated 1	Nucleus	transcription regulator	CWP232291
UQCRC1	0,00	ubiquinol-cytochrome c reductase core protein 1	Cytoplasm	enzyme	
EIF4H	0,00	eukaryotic translation initiation factor 4H	Cytoplasm	translation regulator	
PSMD4	0,00	proteasome 26S subunit ubiquitin receptor, non-ATPase 4	Cytoplasm	other	
HNRNPDL	0,00	heterogeneous nuclear ribonucleoprotein D like	Nucleus	other	
GTPBP4	0,00	GTP binding protein 4	Nucleus	enzyme	

EIF3C	0,00	eukaryotic translation initiation factor 3 subunit C	Cytoplasm	translation regulator	
SRSF6	0,00	serine and arginine rich splicing factor 6	Nucleus	other	
MAPK1	0,00	mitogen-activated protein kinase 1	Cytoplasm	kinase	pexmetinib, ulixertinib, HE3286, ASN007, tyrphostin AG 127, rineterkib, CAY10561, ASTX029, (5Z)-3-(2-aminoethyl)-5-[[4-(2-methylpropoxy)phenyl]methylidene]-1,3-thiazolidine-2,4-dione, tyrphostin AG 1288, HH2710, KO-947, ZSET 1446, SM1-71, MAP kinase1 inhibitor, temuterkib, ATG-017, Vx-11e, binimetinib, FR 180204, (5Z)-3-(2-aminoethyl)-5-[(4-phenylmethoxyphenyl)methylidene]-1,3-thiazolidine-2,4-dione, (Z)-3-(2-aminoethyl)-5-(4-ethoxybenzylidene)thiazolidine-2,4-dione, LY-3007113, JSI-1187
NDUFV1	0,00	NADH:ubiquinone oxidoreductase core subunit V1	Cytoplasm	enzyme	
ACO1	0,00	aconitase 1	Cytoplasm	enzyme	
SEC61B	0,00	SEC61 translocon subunit beta	Cytoplasm	transporter	
SF3B3	0,00	splicing factor 3b subunit 3	Nucleus	other	
SRSF7	0,00	serine and arginine rich splicing factor 7	Nucleus	other	
PRPF19	0,00	pre-mRNA processing factor 19	Nucleus	enzyme	
SNRPA1	0,00	small nuclear ribonucleoprotein polypeptide A'	Nucleus	other	
TRA2B	0,00	transformer 2 beta homolog	Nucleus	other	
CPSF6	0,00	cleavage and polyadenylation specific factor 6	Nucleus	other	
SHMT2	0,00	serine hydroxymethyltransferase 2	Cytoplasm	enzyme	
VARS1	0,00	valyl-tRNA synthetase 1	Cytoplasm	enzyme	
PPIA	0,00	peptidylprolyl isomerase A	Cytoplasm	enzyme	cyclosporin A/methotrexate, basiliximab/cyclosporine, cyclosporine A/sirolimus/tacrolimus, N-methyl-4-Ile-cyclosporin, alemtuzumab/cyclosporin A, RMC-6291, cyclosporin A, RMC-6236, cyclosporine A/tacrolimus
NDUFS2	0,00	NADH:ubiquinone oxidoreductase core subunit S2	Cytoplasm	enzyme	
PSMC3	0,00	proteasome 26S subunit, ATPase 3	Nucleus	enzyme	
NDUFS1	0,00	NADH:ubiquinone oxidoreductase core subunit S1	Cytoplasm	enzyme	
SSB	0,00	small RNA binding exonuclease protection factor La	Nucleus	enzyme	
PSMD12	0,00	proteasome 26S subunit, non-ATPase 12	Cytoplasm	other	
SRSF9	0,00	serine and arginine rich splicing factor 9	Nucleus	enzyme	
CFL1	0,00	cofilin 1	Nucleus	other	
UQCRC2	0,00	ubiquinol-cytochrome c reductase core protein 2	Cytoplasm	enzyme	
PSMD7	0,00	proteasome 26S subunit, non-ATPase 7	Cytoplasm	other	
SFPQ	0,00	splicing factor proline and glutamine rich	Nucleus	other	
GOT2	0,00	glutamic-oxaloacetic transaminase 2	Cytoplasm	enzyme	
RPS27L	0,00	ribosomal protein S27 like	Cytoplasm	translation regulator	
EIF3D	0,00	eukaryotic translation initiation factor 3 subunit D	Cytoplasm	translation regulator	
PFDN5	0,00	prefoldin subunit 5	Nucleus	transcription regulator	
DRG1	0,00	developmentally regulated GTP binding protein 1	Cytoplasm	enzyme	
RAB5A	0,00	RAB5A, member RAS oncogene family	Cytoplasm	enzyme	
DLAT	0,00	dihydrolipoamide S-acetyltransferase	Cytoplasm	enzyme	
RPLP1	0,00	ribosomal protein lateral stalk subunit P1	Cytoplasm	other	
EEF1D	0,00	eukaryotic translation elongation factor 1 delta	Cytoplasm	translation regulator	
TSFM	0,00	Ts translation elongation factor, mitochondrial	Cytoplasm	translation regulator	

ANXA2	0,00	annexin A2	Plasma Membrane	other	
RELA	0,00	RELA proto-oncogene, NF-kB subunit	Nucleus	transcription regulator	NF-kappaB decoy
TNPO1	0,00	transportin 1	Nucleus	transporter	
PRKACA	0,00	protein kinase cAMP-activated catalytic subunit alpha	Cytoplasm	kinase	
FLNA	0,00	filamin A	Cytoplasm	other	
IQGAP1	0,00	IQ motif containing GTPase activating protein 1	Cytoplasm	other	
SQSTM1	0,00	sequestosome 1	Cytoplasm	transcription regulator	
SNRNP200	0,00	small nuclear ribonucleoprotein U5 subunit 200	Nucleus	enzyme	
PPA1	0,00	inorganic pyrophosphatase 1	Cytoplasm	enzyme	
POLR2A	0,00	RNA polymerase II subunit A	Nucleus	enzyme	
DKC1	0,00	dyskerin pseudouridine synthase 1	Nucleus	enzyme	
DDX6	0,00	DEAD-box helicase 6	Nucleus	enzyme	
SF3A1	0,00	splicing factor 3a subunit 1	Nucleus	other	
SF3A3	0,00	splicing factor 3a subunit 3	Nucleus	other	
MRPS16	0,00	mitochondrial ribosomal protein S16	Cytoplasm	other	
CAPZB	0,00	capping actin protein of muscle Z-line subunit beta	Cytoplasm	other	vacuolin-1
PABPN1	0,00	poly(A) binding protein nuclear 1	Nucleus	enzyme	
YWHAB	0,00	tyrosine 3-monooxygenase/tryptophan 5-monooxygenase activation protein beta	Cytoplasm	other	
GCN1	0,00	GCN1 activator of EIF2AK4	Cytoplasm	translation regulator	
VCL	0,00	vinculin	Plasma Membrane	other	
UBE2I	0,00	ubiquitin conjugating enzyme E2 I	Nucleus	enzyme	
DDX17	0,00	DEAD-box helicase 17	Nucleus	enzyme	
FASN	0,00	fatty acid synthase	Cytoplasm	enzyme	TVB-2640, orlistat, cerulenin, 3-V bioscience-2640, acarbose/orlistat
IMPDH2	0,00	inosine monophosphate dehydrogenase 2	Cytoplasm	enzyme	mycophenolic acid, thioguanine, mycophenolate mofetil, imatinib/thioguanine, VX-944, pegintron/ribavirin, mycophenolate mofetil/prednisone, methylprednisolone/mycophenolate mofetil, interferon alfacon-1/ribavirin, 6-mercaptopurine/prednisone/thioguanine, cytarabine/daunorubicin/thioguanine, cytarabine/thioguanine, IFNA2B/ribavirin, ribavirin
NDUFV2	0,00	NADH:ubiquinone oxidoreductase core subunit V2	Cytoplasm	enzyme	
CYC1	0,00	cytochrome c1	Cytoplasm	enzyme	
ECHS1	0,00	enoyl-CoA hydratase, short chain 1	Cytoplasm	enzyme	
VDAC2	0,00	voltage dependent anion channel 2	Cytoplasm	ion channel	
TPT1	0,00	tumor protein, translationally-controlled 1	Cytoplasm	other	
SF3B2	0,00	splicing factor 3b subunit 2	Nucleus	other	
PDIA3	0,00	protein disulfide isomerase family A member 3	Cytoplasm	peptidase	
PHB2	0,00	prohibitin 2	Cytoplasm	transcription regulator	
KHSRP	0,00	KH-type splicing regulatory protein	Nucleus	enzyme	
SRRM1	0,00	serine and arginine repetitive matrix 1	Nucleus	other	
GSR	0,00	glutathione-disulfide reductase	Cytoplasm	enzyme	carmustine/prednisone, carmustine
HNRNPF	0,00	heterogeneous nuclear ribonucleoprotein F	Nucleus	other	
HNRNPA3	0,00	heterogeneous nuclear ribonucleoprotein A3	Nucleus	other	
SF1	0,00	splicing factor 1	Nucleus	transcription regulator	
EIF3H	0,00	eukaryotic translation initiation factor 3 subunit H	Cytoplasm	enzyme	
NDUFA9	0,00	NADH:ubiquinone oxidoreductase subunit A9	Cytoplasm	enzyme	

LAMP1	0,00	lysosomal associated membrane protein 1	Plasma Membrane	other	SAR428926
DDX1	0,00	DEAD-box helicase 1	Nucleus	enzyme	
PES1	0,00	pescadillo ribosomal biogenesis factor 1	Nucleus	other	
TPR	0,00	translocated promoter region, nuclear basket protein	Nucleus	other	
PSMC6	0,00	proteasome 26S subunit, ATPase 6	Nucleus	peptidase	
SKP1	0,00	S-phase kinase associated protein 1	Nucleus	transcription regulator	
PRDX1	0,00	peroxiredoxin 1	Cytoplasm	enzyme	
TKT	0,00	transketolase	Cytoplasm	enzyme	
STAU1	0,00	staufen double-stranded RNA binding protein 1	Cytoplasm	transporter	
DDX18	0,00	DEAD-box helicase 18	Nucleus	enzyme	
ERH	0,00	ERH mRNA splicing and mitosis factor	Nucleus	other	
RAB7A	0,00	RAB7A, member RAS oncogene family	Cytoplasm	enzyme	
NME1	0,00	NME/NM23 nucleoside diphosphate kinase 1	Cytoplasm	kinase	
CSNK2A1	0,00	casein kinase 2 alpha 1	Nucleus	kinase	SM1-71
RSL1D1	0,00	ribosomal L1 domain containing 1	Nucleus	other	
YWHAE	0,00	tyrosine 3-monooxygenase/tryptophan 5-monooxygenase activation protein epsilon	Cytoplasm	other	
XRCC6	0,00	X-ray repair cross complementing 6	Nucleus	enzyme	
TALDO1	0,00	transaldolase 1	Cytoplasm	enzyme	
P4HB	0,00	prolyl 4-hydroxylase subunit beta	Cytoplasm	enzyme	
EIF3L	0,00	eukaryotic translation initiation factor 3 subunit L	Cytoplasm	translation regulator	
ETFA	0,00	electron transfer flavoprotein subunit alpha	Cytoplasm	transporter	
CUL3	0,00	cullin 3	Nucleus	enzyme	
SNW1	0,00	SNW domain containing 1	Nucleus	transcription regulator	
PHGDH	0,00	phosphoglycerate dehydrogenase	Cytoplasm	enzyme	
PMPCB	0,00	peptidase, mitochondrial processing subunit beta	Cytoplasm	peptidase	
ITGB1	0,00	integrin subunit beta 1	Plasma Membrane	transmembrane receptor	OS2966
RNPS1	0,00	RNA binding protein with serine rich domain 1	Nucleus	other	
SRRT	0,00	serrate, RNA effector molecule	Nucleus	other	
EWSR1	0,00	EWS RNA binding protein 1	Nucleus	other	
TP53BP1	0,00	tumor protein p53 binding protein 1	Nucleus	transcription regulator	
NONO	0,00	non-POU domain containing octamer binding	Nucleus	transcription regulator	
RPN1	0,00	ribophorin I	Cytoplasm	enzyme	
HADHB	0,00	hydroxyacyl-CoA dehydrogenase trifunctional multienzyme complex subunit beta	Cytoplasm	enzyme	
USP14	0,00	ubiquitin specific peptidase 14	Cytoplasm	peptidase	VLX1570
METAP2	0,00	methionyl aminopeptidase 2	Cytoplasm	peptidase	APL-1202, XMT-1107, O-(chloroacetylcarbamoyl)fumagillol, beloranib, APL-1501, PPI-2458, M8891, SDX-7320, nitroxoline, aclimostat
ANXA5	0,00	annexin A5	Plasma Membrane	transporter	
IARS1	0,00	isoleucyl-tRNA synthetase 1	Cytoplasm	enzyme	
PFN1	0,00	profilin 1	Cytoplasm	other	
CUL1	0,00	cullin 1	Nucleus	enzyme	
NUDC	0,00	nuclear distribution C, dynein complex regulator	Cytoplasm	other	
EZR	0,00	ezrin	Plasma Membrane	other	
AIMP1	0,00	aminoacyl tRNA synthetase complex interacting multifunctional protein 1	Extracellular Space	cytokine	
PPA2	0,00	inorganic pyrophosphatase 2	Cytoplasm	enzyme	
XRCC5	0,00	X-ray repair cross complementing 5	Nucleus	enzyme	

ATIC	0,00	5-aminoimidazole-4-carboxamide ribonucleotide formyltransferase/IMP cyclohydrolase	Cytoplasm	enzyme	gemcitabine/pemetrexed, pembrolizumab/pemetrexed, pemetrexed
HADHA	0,00	hydroxyacyl-CoA dehydrogenase trifunctional multienzyme complex subunit alpha	Cytoplasm	enzyme	
PPP1CA	0,00	protein phosphatase 1 catalytic subunit alpha	Cytoplasm	phosphatase	
EIF5	0,00	eukaryotic translation initiation factor 5	Cytoplasm	translation regulator	
HNRNPA0	0,00	heterogeneous nuclear ribonucleoprotein A0	Nucleus	other	
OGDH	0,00	oxoglutarate dehydrogenase	Cytoplasm	enzyme	
STT3A	0,00	STT3 oligosaccharyltransferase complex catalytic subunit A	Plasma Membrane	enzyme	
RAC1	0,00	Rac family small GTPase 1	Plasma Membrane	enzyme	
MEPCE	0,00	methylphosphate capping enzyme	Nucleus	enzyme	
NUP153	0,00	nucleoporin 153	Nucleus	other	
DLST	0,00	dihydrolipoamide S-succinyltransferase	Cytoplasm	enzyme	
MYH9	0,00	myosin heavy chain 9	Cytoplasm	enzyme	
SUCLA2	0,00	succinate-CoA ligase ADP-forming subunit beta	Cytoplasm	enzyme	
DNAJC10	0,00	DnaJ heat shock protein family (Hsp40) member C10	Cytoplasm	enzyme	
PAK2	0,00	p21 (RAC1) activated kinase 2	Cytoplasm	kinase	FRAX-597, FRAX486, FRAX355, FRAX120, FRAX-1036
PSMB7	0,00	proteasome 20S subunit beta 7	Cytoplasm	peptidase	
PPP2R1A	0,00	protein phosphatase 2 scaffold subunit Aalpha	Cytoplasm	phosphatase	
ACAA2	0,00	acetyl-CoA acyltransferase 2	Cytoplasm	enzyme	
PRDX3	0,00	peroxiredoxin 3	Cytoplasm	enzyme	
COX5B	0,00	cytochrome c oxidase subunit 5B	Cytoplasm	enzyme	
SEC61G	0,00	SEC61 translocon subunit gamma	Plasma Membrane	transporter	
TOP1	0,00	DNA topoisomerase I	Nucleus	enzyme	bevacizumab/irinotecan/oxaliplatin, SN-38, bevacizumab/paclitaxel/topotecan, delimotecan, belotecan, bevacizumab/irinotecan, 5-fluorouracil/irinotecan/oxaliplatin, BAY 56-3722, capecitabine/cetuximab/irinotecan, cyclophosphamide/topotecan, capecitabine/irinotecan/oxaliplatin, cyclophosphamide/temozolomide/topotecan, cetuximab/gimeracil/irinotecan/oxonic acid/tegafur, Genz-644282, edotecarin, IDEC-132, beta-lapachone, elomotecan, gimeracil/irinotecan/oxonic acid/tegafur, stibogluconic acid, cisplatin/topotecan, mureletecan, irinotecan/temozolomide, irinotecan/vincristine, camptothecin derivative, nab-5404, bevacizumab/gimeracil/irinotecan/oxonic acid/tegafur, cyclophosphamide/irinotecan/temozolomide, cetuximab/irinotecan, lurtotecan, irinotecan/panitumumab, PEN-866, DXd, topotecan, diflomotecan, cytarabine/topotecan, cetuximab/irinotecan/vemurafenib, indotecan, camptothecin-20-O-propionate, HM30181AK/irinotecan, irinotecan/panitumumab/vemurafenib, elsamitrucin, karenitecin, gimatecan, irinotecan/temozolomide/vincristine, bevacizumab/5-fluorouracil/irinotecan/oxaliplatin, camptothecin, batracylin, irinotecan/leucovorin, docetaxel/irinotecan, NEV-801, bevacizumab/capecitabine/irinotecan/oxaliplatin, gemcitabine/irinotecan, DB-67, capecitabine/irinotecan, indimitecan, 5-fluorouracil/irinotecan, 5-fluorouracil/irinotecan/trastuzumab, intoplicine, exatecan, tafluposide, TAS-103, irinotecan/oxaliplatin, MJ-III-65, namitecan, rubitecan, DS-6000a,
NAT10	0,00	N-acetyltransferase 10	Nucleus	enzyme	
NUP98	0,00	nucleoporin 98 and 96 precursor	Nucleus	enzyme	
PRPF40A	0,00	pre-mRNA processing factor 40 homolog A	Nucleus	other	
SNRPC	0,00	small nuclear ribonucleoprotein polypeptide C	Nucleus	other	
STUB1	0,00	STIP1 homology and U-box containing protein 1	Cytoplasm	enzyme	

AFG3L2	0,00	AFG3 like matrix AAA peptidase subunit 2	Cytoplasm	peptidase	
COPB1	0,00	COPI coat complex subunit beta 1	Cytoplasm	transporter	
XRN2	0,00	5'-3' exoribonuclease 2	Nucleus	enzyme	
PRMT1	0,00	protein arginine methyltransferase 1	Nucleus	enzyme	PRMT1 inhibitor, GSK3368715
NUDT21	0,00	nudix hydrolase 21	Nucleus	other	
TIAL1	0,00	TIA1 cytotoxic granule associated RNA binding protein like 1	Nucleus	transcription regulator	
PSME3	0,00	proteasome activator subunit 3	Cytoplasm	peptidase	
HUWE1	0,00	HECT, UBA and WWE domain containing E3 ubiquitin protein ligase 1	Nucleus	transcription regulator	
COX5A	0,00	cytochrome c oxidase subunit 5A	Cytoplasm	enzyme	
PEBP1	0,00	phosphatidylethanolamine binding protein 1	Cytoplasm	other	
EIF4B	0,00	eukaryotic translation initiation factor 4B	Cytoplasm	translation regulator	
VAMP3	0,00	vesicle associated membrane protein 3	Plasma Membrane	other	
**CD44**	0,00	CD44 molecule (Indian blood group)	Plasma Membrane	other	SPL-108, A6 peptide, anti-CD44v7 antibody, AMC303, CA102N, RO5429083
SKIC2	0,00	SKI2 subunit of superkiller complex	Nucleus	enzyme	
THOC2	0,00	THO complex subunit 2	Nucleus	other	
IDH3A	0,00	isocitrate dehydrogenase (NAD(+)) 3 catalytic subunit alpha	Cytoplasm	enzyme	
NAA10	0,00	N-alpha-acetyltransferase 10, NatA catalytic subunit	Nucleus	enzyme	
SRSF4	0,00	serine and arginine rich splicing factor 4	Nucleus	other	
LMNB1	0,00	lamin B1	Nucleus	other	
GLUD1	0,00	glutamate dehydrogenase 1	Cytoplasm	enzyme	
IDH3G	0,00	isocitrate dehydrogenase (NAD(+)) 3 non-catalytic subunit gamma	Cytoplasm	enzyme	
UBA1	0,00	ubiquitin like modifier activating enzyme 1	Cytoplasm	enzyme	MLN7243
ARCN1	0,00	archain 1	Cytoplasm	other	
HSPA2	0,00	heat shock protein family A (Hsp70) member 2	Cytoplasm	other	
GSPT1	0,00	G1 to S phase transition 1	Cytoplasm	translation regulator	
SF3A2	0,00	splicing factor 3a subunit 2	Nucleus	other	
ELOC	0,00	elongin C	Nucleus	transcription regulator	
SUCLG2	0,00	succinate-CoA ligase GDP-forming subunit beta	Cytoplasm	enzyme	
ACADM	0,00	acyl-CoA dehydrogenase medium chain	Cytoplasm	enzyme	
STT3B	0,00	STT3 oligosaccharyltransferase complex catalytic subunit B	Cytoplasm	enzyme	
PDHX	0,00	pyruvate dehydrogenase complex component X	Cytoplasm	enzyme	
METAP1	0,00	methionyl aminopeptidase 1	Cytoplasm	peptidase	
RAB1A	0,00	RAB1A, member RAS oncogene family	Cytoplasm	enzyme	
EIF3K	0,00	eukaryotic translation initiation factor 3 subunit K	Cytoplasm	translation regulator	
ARF6	0,00	ADP ribosylation factor 6	Plasma Membrane	transporter	
SRRM2	0,00	serine/arginine repetitive matrix 2	Nucleus	other	
CTNNBL1	0,00	catenin beta like 1	Nucleus	other	
SNRPB2	0,00	small nuclear ribonucleoprotein polypeptide B2	Nucleus	other	
NDUFA8	0,00	NADH:ubiquinone oxidoreductase subunit A8	Cytoplasm	enzyme	
PRDX2	0,00	peroxiredoxin 2	Cytoplasm	enzyme	
SSBP1	0,00	single stranded DNA binding protein 1	Cytoplasm	other	
LMAN1	0,00	lectin, mannose binding 1	Cytoplasm	other	
DHX38	0,00	DEAH-box helicase 38	Nucleus	enzyme	
DDX23	0,00	DEAD-box helicase 23	Nucleus	enzyme	
DNAJB1	0,00	DnaJ heat shock protein family (Hsp40) member B1	Nucleus	transcription regulator	

SHMT1	0,00	serine hydroxymethyltransferase 1	Cytoplasm	enzyme	mimosine
NDUFA5	0,00	NADH:ubiquinone oxidoreductase subunit A5	Cytoplasm	enzyme	
RAB6A	0,00	RAB6A, member RAS oncogene family	Cytoplasm	enzyme	
NF1	0,00	neurofibromin 1	Cytoplasm	other	
DCTN1	0,00	dynactin subunit 1	Cytoplasm	other	
KPNA2	0,00	karyopherin subunit alpha 2	Nucleus	other	
HDAC6	0,00	histone deacetylase 6	Nucleus	transcription regulator	trametinib/vorinostat, KA2507, belinostat, HG146, vorinostat, ricolinostat, pyroxamide, bortezomib/vorinostat, JBI-802, tributyrin, purinostat
PTGES3	0,00	prostaglandin E synthase 3	Cytoplasm	enzyme	
STIP1	0,00	stress induced phosphoprotein 1	Cytoplasm	other	lavendustin C
FMR1	0,00	fragile X messenger ribonucleoprotein 1	Cytoplasm	translation regulator	
TOMM20	0,00	translocase of outer mitochondrial membrane 20	Cytoplasm	transporter	
NUP62	0,00	nucleoporin 62	Nucleus	transporter	dusquetide
OLA1	0,00	Obg like ATPase 1	Cytoplasm	enzyme	
HSPB1	0,00	heat shock protein family B (small) member 1	Cytoplasm	other	
GOLPH3	0,00	golgi phosphoprotein 3	Cytoplasm	other	
FXR1	0,00	FMR1 autosomal homolog 1	Cytoplasm	other	
TFAM	0,00	transcription factor A, mitochondrial	Cytoplasm	transcription regulator	
CTTN	0,00	cortactin	Plasma Membrane	other	
UBE3A	0,00	ubiquitin protein ligase E3A	Nucleus	enzyme	
TMPO	0,00	thymopoietin	Nucleus	other	
SRP68	0,00	signal recognition particle 68	Nucleus	other	
RBM25	0,00	RNA binding motif protein 25	Nucleus	other	
SSRP1	0,00	structure specific recognition protein 1	Nucleus	transcription regulator	
AHCY	0,00	adenosylhomocysteinase	Cytoplasm	enzyme	9-(2',3'-dihydroxycyclopent-4'-enyl)adenine, 3-deazaneplanocin, neplanocin A, 3-deazaaristeromycin, cyanovirin-N, 6'-C-methylneplanocin A
DRG2	0,00	developmentally regulated GTP binding protein 2	Cytoplasm	enzyme	
UBE2N	0,00	ubiquitin conjugating enzyme E2 N	Cytoplasm	enzyme	
LARS1	0,00	leucyl-tRNA synthetase 1	Cytoplasm	enzyme	
PFDN2	0,00	prefoldin subunit 2	Cytoplasm	other	
PSMD8	0,00	proteasome 26S subunit, non-ATPase 8	Cytoplasm	other	
CFL2	0,00	cofilin 2	Extracellular Space	other	
LSM3	0,00	LSM3 homolog, U6 small nuclear RNA and mRNA degradation associated	Nucleus	other	
SRSF11	0,00	serine and arginine rich splicing factor 11	Nucleus	other	
HDAC2	0,00	histone deacetylase 2	Nucleus	transcription regulator	theophylline, trametinib/vorinostat, HG146, belinostat, domatinostat, vorinostat, histone deacetylase class II inhibitors, pyroxamide, chidamide, bortezomib/vorinostat, tributyrin, purinostat
NUP214	0,00	nucleoporin 214	Nucleus	transporter	
IPO7	0,00	importin 7	Nucleus	transporter	
NDUFB8	0,00	NADH:ubiquinone oxidoreductase subunit B8	Cytoplasm	enzyme	
NDUFS7	0,00	NADH:ubiquinone oxidoreductase core subunit S7	Cytoplasm	enzyme	
SPCS2	0,00	signal peptidase complex subunit 2	Cytoplasm	other	
FTSJ3	0,00	FtsJ RNA 2'-O-methyltransferase 3	Nucleus	enzyme	
DDB1	0,00	damage specific DNA binding protein 1	Nucleus	other	
ALDOA	0,00	aldolase, fructose-bisphosphate A	Cytoplasm	enzyme	
NACA	0,00	nascent polypeptide associated complex subunit alpha	Cytoplasm	transcription regulator	
ATP5PB	0,00	ATP synthase peripheral stalk-membrane subunit b	Cytoplasm	transporter	
ZC3H18	0,00	zinc finger CCCH-type containing 18	Nucleus	other	

MYBBP1A	0,00	MYB binding protein 1a	Nucleus	transcription regulator	
FARSB	0,00	phenylalanyl-tRNA synthetase subunit beta	Cytoplasm	enzyme	
NDUFA13	0,00	NADH:ubiquinone oxidoreductase subunit A13	Cytoplasm	enzyme	
EIF2AK2	0,00	eukaryotic translation initiation factor 2 alpha kinase 2	Cytoplasm	kinase	etavopivat, mitapivat
ACTN4	0,00	actinin alpha 4	Cytoplasm	transcription regulator	
TSG101	0,00	tumor susceptibility 101	Cytoplasm	transcription regulator	
SEC63	0,00	SEC63 homolog, protein translocation regulator	Cytoplasm	transporter	
HNRNPAB	0,00	heterogeneous nuclear ribonucleoprotein A/B	Nucleus	enzyme	
NUP107	0,00	nucleoporin 107	Nucleus	other	
NDUFB9	0,00	NADH:ubiquinone oxidoreductase subunit B9	Cytoplasm	enzyme	
TRAP1	0,00	TNF receptor associated protein 1	Cytoplasm	enzyme	gamitrinib-TPP-OH
PGD	0,00	phosphogluconate dehydrogenase	Cytoplasm	enzyme	
PRDX6	0,00	peroxiredoxin 6	Cytoplasm	enzyme	
CAPZA1	0,00	capping actin protein of muscle Z-line subunit alpha 1	Cytoplasm	other	
RAE1	0,00	ribonucleic acid export 1	Nucleus	other	
USP7	0,00	ubiquitin specific peptidase 7	Nucleus	peptidase	
SMARCA4	0,00	SWI/SNF related, matrix associated, actin dependent regulator of chromatin, subfamily a, member 4	Nucleus	transcription regulator	FHD-286
PRPF6	0,00	pre-mRNA processing factor 6	Nucleus	transcription regulator	
RARS1	0,00	arginyl-tRNA synthetase 1	Cytoplasm	enzyme	
TOMM40	0,00	translocase of outer mitochondrial membrane 40	Cytoplasm	ion channel	
GRPEL1	0,00	GrpE like 1, mitochondrial	Cytoplasm	other	
GOLGA2	0,00	golgin A2	Cytoplasm	other	
PSMB1	0,00	proteasome 20S subunit beta 1	Cytoplasm	peptidase	bortezomib/cladribine/rituximab, bortezomib/dexamethasone/pomalidomide, bortezomib/doxorubicin, bortezomib/dexamethasone/thalidomide, bortezomib/paclitaxel, carfilzomib, bortezomib/sorafenib, bortezomib/fulvestrant, bortezomib/rituximab, bortezomib/lenalidomide, carfilzomib/dexamethasone/lenalidomide, bortezomib/dexamethasone/lenalidomide, bortezomib/dexamethasone/doxorubicin, bortezomib/dexamethasone, bortezomib/prednisone, bortezomib/vorinostat, bortezomib, carfilzomib/dexamethasone, carfilzomib/dexamethasone/rituximab, bortezomib/thalidomide, bortezomib/dexamethasone/rituximab
DDX39A	0,00	DExD-box helicase 39A	Nucleus	enzyme	
RBBP7	0,00	RB binding protein 7, chromatin remodeling factor	Nucleus	transcription regulator	
MAT2B	0,00	methionine adenosyltransferase 2 non-catalytic beta subunit	Cytoplasm	enzyme	
PCCB	0,00	propionyl-CoA carboxylase subunit beta	Cytoplasm	enzyme	
IARS2	0,00	isoleucyl-tRNA synthetase 2, mitochondrial	Cytoplasm	enzyme	
IDH3B	0,00	isocitrate dehydrogenase (NAD(+)) 3 non-catalytic subunit beta	Cytoplasm	enzyme	
HEATR1	0,00	HEAT repeat containing 1	Nucleus	other	
GLO1	0,00	glyoxalase I	Cytoplasm	enzyme	
NDUFS4	0,00	NADH:ubiquinone oxidoreductase subunit S4	Cytoplasm	enzyme	
BAG3	0,00	BAG cochaperone 3	Cytoplasm	other	
CAPZA2	0,00	capping actin protein of muscle Z-line subunit alpha 2	Cytoplasm	other	
YWHAG	0,00	tyrosine 3-monooxygenase/tryptophan 5-monooxygenase activation protein gamma	Cytoplasm	other	

SPCS1	0,00	signal peptidase complex subunit 1	Cytoplasm	peptidase	
HBS1L	0,00	HBS1 like translational GTPase	Cytoplasm	translation regulator	
SF3B6	0,00	splicing factor 3b subunit 6	Nucleus	other	
PAICS	0,00	phosphoribosylaminoimidazole carboxylase and phosphoribosylaminoimidazolesucc inocarboxamide synthase	Cytoplasm	enzyme	
ADSL	0,00	adenylosuccinate lyase	Cytoplasm	enzyme	
NAPA	0,00	NSF attachment protein alpha	Cytoplasm	transporter	
ACTR2	0,00	actin related protein 2	Plasma Membrane	other	
SMARCA5	0,00	SWI/SNF related, matrix associated, actin dependent regulator of chromatin, subfamily a, member 5	Nucleus	transcription regulator	
ACAD9	0,00	acyl-CoA dehydrogenase family member 9	Cytoplasm	enzyme	
ATG7	0,00	autophagy related 7	Cytoplasm	enzyme	
AK2	0,00	adenylate kinase 2	Cytoplasm	kinase	
SRPRA	0,00	SRP receptor subunit alpha	Cytoplasm	other	
NSF	0,00	N-ethylmaleimide sensitive factor, vesicle fusing ATPase	Cytoplasm	transporter	
DIS3	0,00	DIS3 homolog, exosome endoribonuclease and 3'-5' exoribonuclease	Nucleus	enzyme	
DNAJA1	0,00	DnaJ heat shock protein family (Hsp40) member A1	Nucleus	other	
LMNA	0,00	lamin A/C	Nucleus	other	
COPS6	0,00	COP9 signalosome subunit 6	Nucleus	other	
PDCD5	0,00	programmed cell death 5	Nucleus	other	
ACTN1	0,00	actinin alpha 1	Nucleus	transcription regulator	
TUBA1C	0,00	tubulin alpha 1c	Cytoplasm	other	docetaxel/gemcitabine/vincristine, bevacizumab/paclitaxel/topotecan, colchicine/probenecid, cyclophosphamide/epirubicin/5-fluorouracil/paclitaxel, bevacizumab/docetaxel/prednisone/thalidomide/zoledronic acid, docetaxel/zoledronic acid, gemcitabine/paclitaxel, L-asparaginase/daunorubicin/dexamethasone/vincristine, prednisone/vincristine, docetaxel/5-fluorouracil, vinflunine, L-asparaginase/dexamethasone/imatinib/vincristine, L-asparaginase/daunorubicin/dexamethasone/imatinib/vincristine, L-asparaginase/daunorubicin/imatinib/prednisone/vincristine, cyclophosphamide/docetaxel/epirubicin/5-fluorouracil/trastuzumab, docetaxel/5-fluorouracil/oxaliplatin, vincristine, docetaxel/paclitaxel, cevipabulin, cyclophosphamide/epirubicin/paclitaxel, L-asparaginase/daunorubicin/prednisone/vincristine, cyclophosphamide/docetaxel/trastuzumab, bevacizumab/paclitaxel/pemetrexed, irinotecan/vincristine, L-asparaginase/imatinib/prednisone/vincristine, cyclophosphamide/gemcitabine/prednisolone/rituximab/vincristine, cyclophosphamide/prednisone/vincristine, docetaxel/hydrocortisone, docetaxel/gemcitabine/vinorelbine, epirubicin/paclitaxel, capecitabine/docetaxel/gemcitabine, cyclophosphamide/docetaxel, docetaxel/gemcitabine, bevacizumab/docetaxel, cyclophosphamide/daunorubicin/imatinib/prednisolone/vincristine, docetaxel/prednisone, vinorelbine,
SRP19	0,00	signal recognition particle 19	Cytoplasm	other	
CDC37	0,00	cell division cycle 37, HSP90 cochaperone	Cytoplasm	other	
TLN1	0,00	talin 1	Plasma Membrane	other	
SF3B4	0,00	splicing factor 3b subunit 4	Nucleus	other	
NUP205	0,00	nucleoporin 205	Nucleus	other	
STAT1	0,00	signal transducer and activator of transcription 1	Nucleus	transcription regulator	
PPIB	0,00	peptidylprolyl isomerase B	Cytoplasm	enzyme	
ADSS2	0,00	adenylosuccinate synthase 2	Cytoplasm	enzyme	alanosine, adenylosuccinate synthetase inhibitor
PSMD6	0,00	proteasome 26S subunit, non-ATPase 6	Cytoplasm	enzyme	

BCL2L1	0,00	BCL2 like 1	Cytoplasm	other	LP-118, AZD0466
SEH1L	0,00	SEH1 like nucleoporin	Cytoplasm	transporter	
NDUFA10	0,00	NADH:ubiquinone oxidoreductase subunit A10	Cytoplasm	transporter	
SKIC8	0,00	SKI8 subunit of superkiller complex	Nucleus	other	
BCLAF1	0,00	BCL2 associated transcription factor 1	Nucleus	transcription regulator	
UQCRB	0,00	ubiquinol-cytochrome c reductase binding protein	Cytoplasm	enzyme	
DNM1L	0,00	dynamin 1 like	Cytoplasm	enzyme	
NDUFB10	0,00	NADH:ubiquinone oxidoreductase subunit B10	Cytoplasm	enzyme	
PRKCA	0,00	protein kinase C alpha	Cytoplasm	kinase	aprinocarsen, Go6983, L-threo-safingol, Go 6976, Ro31-8220, ingenol mebutate, midostaurin
WASL	0,00	WASP like actin nucleation promoting factor	Cytoplasm	other	
DDX46	0,00	DEAD-box helicase 46	Nucleus	enzyme	
PCNA	0,00	proliferating cell nuclear antigen	Nucleus	enzyme	AOH1996
RANBP1	0,00	RAN binding protein 1	Nucleus	other	
NUP93	0,00	nucleoporin 93	Nucleus	other	
YKT6	0,00	YKT6 v-SNARE homolog	Cytoplasm	enzyme	
HSDL2	0,00	hydroxysteroid dehydrogenase like 2	Cytoplasm	transporter	
RAB8A	0,00	RAB8A, member RAS oncogene family	Plasma Membrane	enzyme	
NUDCD1	0,00	NudC domain containing 1	Nucleus	other	
FUBP1	0,00	far upstream element binding protein 1	Nucleus	transcription regulator	
ALDH3A2	0,00	aldehyde dehydrogenase 3 family member A2	Cytoplasm	enzyme	
DDOST	0,00	dolichyl-diphosphooligosaccharide--protein glycosyltransferase non-catalytic subunit	Cytoplasm	enzyme	
NDUFB7	0,00	NADH:ubiquinone oxidoreductase subunit B7	Cytoplasm	enzyme	
MAP2K1	0,00	mitogen-activated protein kinase kinase 1	Cytoplasm	kinase	binimetinib/vemurafenib, ARRY-424704, dabrafenib/trametinib, dabrafenib/pembrolizumab/trametinib, TAK 733, binimetinib/encorafenib/panitumumab, E 6201, refametinib, PD184352, binimetinib, trametinib, RO4927350, cobimetinib, U0126, pimasertib, AS703988, dabrafenib/trametinib/vemurafenib, trametinib/vemurafenib, SM1-71, docetaxel/selumetinib, binimetinib/cetuximab/encorafenib, MKK1 inhibitor, mirdametinib, cetuximab/dabrafenib/trametinib, PD318088, cobimetinib/vemurafenib, cobimetinib/dabrafenib, selumetinib, binimetinib/encorafenib, FCN-159, trametinib/vorinostat
PSMB4	0,00	proteasome 20S subunit beta 4	Cytoplasm	peptidase	
PMPCA	0,00	peptidase, mitochondrial processing subunit alpha	Cytoplasm	peptidase	
SLC25A3	0,00	solute carrier family 25 member 3	Cytoplasm	transporter	
PTMA	0,00	prothymosin alpha	Nucleus	other	
PUF60	0,00	poly(U) binding splicing factor 60	Nucleus	other	
ACADVL	0,00	acyl-CoA dehydrogenase very long chain	Cytoplasm	enzyme	
NDUFA12	0,00	NADH:ubiquinone oxidoreductase subunit A12	Cytoplasm	enzyme	
VDAC3	0,00	voltage dependent anion channel 3	Cytoplasm	ion channel	
PFKM	0,00	phosphofructokinase, muscle	Cytoplasm	kinase	
LONP1	0,00	lon peptidase 1, mitochondrial	Cytoplasm	peptidase	
SPG7	0,00	SPG7 matrix AAA peptidase subunit, paraplegin	Cytoplasm	peptidase	
TMED10	0,00	transmembrane p24 trafficking protein 10	Cytoplasm	transporter	
POLR2H	0,00	RNA polymerase II, I and III subunit H	Nucleus	enzyme	
HNRNPH2	0,00	heterogeneous nuclear ribonucleoprotein H2	Nucleus	other	
LUC7L3	0,00	LUC7 like 3 pre-mRNA splicing factor	Nucleus	other	

NUP54	0,00	nucleoporin 54	Nucleus	other	
NUP155	0,00	nucleoporin 155	Nucleus	other	
RANGAP1	0,00	Ran GTPase activating protein 1	Nucleus	other	
XAB2	0,00	XPA binding protein 2	Nucleus	other	
TXNRD2	0,00	thioredoxin reductase 2	Cytoplasm	enzyme	
DNAJB11	0,00	DnaJ heat shock protein family (Hsp40) member B11	Cytoplasm	other	
KIF5B	0,00	kinesin family member 5B	Cytoplasm	other	
SLC25A5	0,00	solute carrier family 25 member 5	Cytoplasm	transporter	clodronic acid
LRRC47	0,00	leucine rich repeat containing 47	Other	other	
CUL4A	0,00	cullin 4A	Nucleus	enzyme	
HNRNPH3	0,00	heterogeneous nuclear ribonucleoprotein H3	Nucleus	other	
NOP2	0,00	NOP2 nucleolar protein	Nucleus	other	
CPSF1	0,00	cleavage and polyadenylation specific factor 1	Nucleus	other	
ACTR3	0,00	actin related protein 3	Plasma Membrane	other	
SCARB2	0,00	scavenger receptor class B member 2	Plasma Membrane	transmembrane receptor	
SNAP23	0,00	synaptosome associated protein 23	Plasma Membrane	transporter	
CUL2	0,00	cullin 2	Nucleus	enzyme	
SMU1	0,00	SMU1 DNA replication regulator and spliceosomal factor	Nucleus	other	
SART1	0,00	spliceosome associated factor 1, recruiter of U4/U6.U5 tri-snRNP	Nucleus	other	
NUP160	0,00	nucleoporin 160	Nucleus	other	
ACAT1	0,00	acetyl-CoA acetyltransferase 1	Cytoplasm	enzyme	
HADH	0,00	hydroxyacyl-CoA dehydrogenase	Cytoplasm	enzyme	
FARSA	0,00	phenylalanyl-tRNA synthetase subunit alpha	Cytoplasm	enzyme	
SEC22B	0,00	SEC22 homolog B, vesicle trafficking protein	Cytoplasm	other	
HYOU1	0,00	hypoxia up-regulated 1	Cytoplasm	other	
PGAM1	0,00	phosphoglycerate mutase 1	Cytoplasm	phosphatase	
**B2M**	0,00	beta-2-microglobulin	Plasma Membrane	transmembrane receptor	4'-iodo-4'-deoxydoxorubicin
LSM8	0,00	LSM8 homolog, U6 small nuclear RNA associated	Nucleus	other	
NUP88	0,00	nucleoporin 88	Nucleus	other	
ACTL6A	0,00	actin like 6A	Nucleus	other	
BCKDHA	0,00	branched chain keto acid dehydrogenase E1 subunit alpha	Cytoplasm	enzyme	
NDUFS5	0,00	NADH:ubiquinone oxidoreductase subunit S5	Cytoplasm	enzyme	
SAMM50	0,00	SAMM50 sorting and assembly machinery component	Cytoplasm	other	
SHC1	0,00	SHC adaptor protein 1	Cytoplasm	other	
PSMD3	0,00	proteasome 26S subunit, non-ATPase 3	Cytoplasm	other	
VPS35	0,00	VPS35 retromer complex component	Cytoplasm	transporter	
FDX1	0,00	ferredoxin 1	Cytoplasm	transporter	
TMED2	0,00	transmembrane p24 trafficking protein 2	Cytoplasm	transporter	
NSDHL	0,00	NAD(P) dependent steroid dehydrogenase-like	Cytoplasm	enzyme	
PDIA4	0,00	protein disulfide isomerase family A member 4	Cytoplasm	enzyme	
TRIP12	0,00	thyroid hormone receptor interactor 12	Cytoplasm	enzyme	
ALDH2	0,00	aldehyde dehydrogenase 2 family member	Cytoplasm	enzyme	disulfiram/gemcitabine, disulfiram, chlorpropamide, ANS-6637
NDUFA6	0,00	NADH:ubiquinone oxidoreductase subunit A6	Cytoplasm	enzyme	
EIF4G2	0,00	eukaryotic translation initiation factor 4 gamma 2	Cytoplasm	translation regulator	
RBBP4	0,00	RB binding protein 4, chromatin remodeling factor	Nucleus	enzyme	
CMPK1	0,00	cytidine/uridine monophosphate kinase 1	Nucleus	kinase	
BUB3	0,00	BUB3 mitotic checkpoint protein	Nucleus	other	

SBDS	0,00	SBDS ribosome maturation factor	Nucleus	other	
RRP12	0,00	ribosomal RNA processing 12 homolog	Nucleus	other	
CASP8	0,00	caspase 8	Nucleus	peptidase	
COX7A2	0,00	cytochrome c oxidase subunit 7A2	Cytoplasm	enzyme	
NDUFS6	0,00	NADH:ubiquinone oxidoreductase subunit S6	Cytoplasm	enzyme	
DAP3	0,00	death associated protein 3	Cytoplasm	other	
DYNC1H1	0,00	dynein cytoplasmic 1 heavy chain 1	Cytoplasm	peptidase	
TOMM22	0,00	translocase of outer mitochondrial membrane 22	Cytoplasm	transporter	
GOSR1	0,00	golgi SNAP receptor complex member 1	Cytoplasm	transporter	
CSTF3	0,00	cleavage stimulation factor subunit 3	Nucleus	other	
ALDH9A1	0,00	aldehyde dehydrogenase 9 family member A1	Cytoplasm	enzyme	
NDUFB5	0,00	NADH:ubiquinone oxidoreductase subunit B5	Cytoplasm	enzyme	
ACOX1	0,00	acyl-CoA oxidase 1	Cytoplasm	enzyme	
G3BP2	0,00	G3BP stress granule assembly factor 2	Cytoplasm	enzyme	
HMGCL	0,00	3-hydroxy-3-methylglutaryl-CoA lyase	Cytoplasm	enzyme	
EEA1	0,00	early endosome antigen 1	Cytoplasm	other	
DNM2	0,00	dynamin 2	Plasma Membrane	enzyme	
**SLC2A1**	0,00	solute carrier family 2 member 1	Plasma Membrane	transporter	canakinumab/metformin/sulfonylurea, insulin glargine/lixisenatide/metformin, metformin/vildagliptin, metformin/rosiglitazone/sulfonylurea, metformin/sulfonylurea/vildagliptin, metformin, metformin/pioglitazone/sulfonylurea, INS/metformin/sitagliptin, linagliptin/metformin, INS/metformin/pioglitazone, alogliptin/metformin/pioglitazone, metformin/sitagliptin/sulfonylurea, exenatide/metformin/sulfonylurea, dapagliflozin/metformin/saxagliptin, exenatide/INS/metformin/pioglitazone, metformin/sirolimus, metformin/saxagliptin, exenatide/metformin, insulin glargine/metformin, INS/metformin/saxagliptin, metformin/pioglitazone, insulin glargine/metformin/sulfonylurea, empagliflozin/linagliptin/metformin, canakinumab/metformin, canagliflozin/metformin, metformin/sulfonylurea, alogliptin/metformin, BAY-876, dapagliflozin/metformin, rosiglitazone-metformin combination, empagliflozin/metformin, INS/linagliptin/metformin, exenatide/INS/metformin, INS/metformin/vildagliptin, INS/metformin, ertugliflozin/metformin, metformin/sitagliptin, dapagliflozin/INS/metformin, alogliptin/INS/metformin
NPLOC4	0,00	NPL4 homolog, ubiquitin recognition factor	Nucleus	other	
GOT1	0,00	glutamic-oxaloacetic transaminase 1	Cytoplasm	enzyme	
CTPS2	0,00	CTP synthase 2	Cytoplasm	enzyme	
DARS2	0,00	aspartyl-tRNA synthetase 2, mitochondrial	Cytoplasm	enzyme	
PFKL	0,00	phosphofructokinase, liver type	Cytoplasm	kinase	
RAB1B	0,00	RAB1B, member RAS oncogene family	Cytoplasm	other	
VBP1	0,00	VHL binding protein 1	Cytoplasm	other	
PSMD13	0,00	proteasome 26S subunit, non-ATPase 13	Cytoplasm	peptidase	
CSTF2	0,00	cleavage stimulation factor subunit 2	Nucleus	other	
PELO	0,00	pelota mRNA surveillance and ribosome rescue factor	Nucleus	other	
DYNLL1	0,00	dynein light chain LC8-type 1	Cytoplasm	other	

YWHAQ	0,00	tyrosine 3-monooxygenase/tryptophan 5-monooxygenase activation protein theta	Cytoplasm	other	
AP2M1	0,00	adaptor related protein complex 2 subunit mu 1	Cytoplasm	other	
ETFB	0,00	electron transfer flavoprotein subunit beta	Cytoplasm	transporter	
COPB2	0,00	COPI coat complex subunit beta 2	Cytoplasm	transporter	
TOMM70	0,00	translocase of outer mitochondrial membrane 70	Cytoplasm	transporter	
GSN	0,00	gelsolin	Extracellular Space	other	
BCAS2	0,00	BCAS2 pre-mRNA processing factor	Nucleus	other	
RALY	0,00	RALY heterogeneous nuclear ribonucleoprotein	Nucleus	transcription regulator	
PDIA6	0,00	protein disulfide isomerase family A member 6	Cytoplasm	enzyme	
PFKP	0,00	phosphofructokinase, platelet	Cytoplasm	kinase	
ACTR1A	0,00	actin related protein 1A	Cytoplasm	other	
CTSD	0,00	cathepsin D	Cytoplasm	peptidase	
PPP1CB	0,00	protein phosphatase 1 catalytic subunit beta	Cytoplasm	phosphatase	
**IGF2R**	0,00	insulin like growth factor 2 receptor	Plasma Membrane	transmembrane receptor	avalglucosidase alfa
SAFB	0,00	scaffold attachment factor B	Nucleus	other	
UBXN7	0,00	UBX domain protein 7	Nucleus	other	
U2SURP	0,00	U2 snRNP associated SURP domain containing	Nucleus	other	
PRPF31	0,00	pre-mRNA processing factor 31	Nucleus	other	
COX6C	0,00	cytochrome c oxidase subunit 6C	Cytoplasm	enzyme	
UQCR10	0,00	ubiquinol-cytochrome c reductase, complex III subunit X	Cytoplasm	enzyme	
GRHPR	0,00	glyoxylate and hydroxypyruvate reductase	Cytoplasm	enzyme	GSK1278863A
MAPK14	0,00	mitogen-activated protein kinase 14	Cytoplasm	kinase	pamapimod, UM101, RO-3201195, CGH2466, ARRY-371797, p38 MAP kinase inhibitor, AMG 2372, Sb202190, PH-797804, talmapimod, p38 MAP kinase inhibitor IV, Org 48762-0, ralimetinib
MRPL46	0,00	mitochondrial ribosomal protein L46	Cytoplasm	other	
HGS	0,00	hepatocyte growth factor-regulated tyrosine kinase substrate	Cytoplasm	other	
VAPA	0,00	VAMP associated protein A	Plasma Membrane	other	
ACIN1	0,00	apoptotic chromatin condensation inducer 1	Nucleus	enzyme	
APEX1	0,00	apurinic/apyrimidinic endodeoxyribonuclease 1	Nucleus	enzyme	lucanthone, E 3330
PRPF4	0,00	pre-mRNA processing factor 4	Nucleus	other	
PELP1	0,00	proline, glutamate and leucine rich protein 1	Nucleus	other	
ECI2	0,00	enoyl-CoA delta isomerase 2	Cytoplasm	enzyme	
OAT	0,00	ornithine aminotransferase	Cytoplasm	enzyme	
FKBP1A	0,00	FKBP prolyl isomerase 1A	Cytoplasm	enzyme	cyclosporine A/sirolimus/tacrolimus, everolimus/fulvestrant, everolimus/pasireotide, corticosteroid/sirolimus, corticosteroid/everolimus/tacrolimus, everolimus, pimecrolimus, temsirolimus, everolimus/exemestane, cyclosporine A/tacrolimus, everolimus/ribociclib, everolimus/letrozole, lenalidomide/temsirolimus, sirolimus, everolimus/gefitinib, everolimus/tamoxifen, metformin/sirolimus, everolimus/paclitaxel, methotrexate/sirolimus/tacrolimus, prednisone/tacrolimus, methylprednisolone/tacrolimus, imatinib/sirolimus, tacrolimus
RTCB	0,00	RNA 2',3'-cyclic phosphate and 5'-OH ligase	Cytoplasm	enzyme	
HIBCH	0,00	3-hydroxyisobutyryl-CoA hydrolase	Cytoplasm	enzyme	
CARS1	0,00	cysteinyl-tRNA synthetase 1	Cytoplasm	enzyme	
ABCF1	0,00	ATP binding cassette subfamily F member 1	Cytoplasm	transporter	

CCDC47	0,00	coiled-coil domain containing 47	Extracellular Space	other	
AIFM1	0,00	apoptosis inducing factor mitochondria associated 1	Cytoplasm	enzyme	
BCAT2	0,00	branched chain amino acid transaminase 2	Cytoplasm	enzyme	
SYMPK	0,00	symplekin scaffold protein	Cytoplasm	other	
EPS15	0,00	epidermal growth factor receptor pathway substrate 15	Cytoplasm	other	
PSMB5	0,00	proteasome 20S subunit beta 5	Cytoplasm	peptidase	bortezomib/thalidomide, bortezomib/dexamethasone/rituximab, dexamethasone/ixazomib/rituximab, ixazomib, bortezomib/cladribine/rituximab, bortezomib/dexamethasone/pomalidomide, bortezomib/doxorubicin, bortezomib/dexamethasone/thalidomide, bortezomib/paclitaxel, carfilzomib, bortezomib/sorafenib, bortezomib/fulvestrant, bortezomib/rituximab, bortezomib/lenalidomide, carfilzomib/dexamethasone/lenalidomide, bortezomib/dexamethasone/lenalidomide, bortezomib/dexamethasone/doxorubicin, dexamethasone/ixazomib/lenalidomide, bortezomib/dexamethasone, bortezomib/prednisone, bortezomib/vorinostat, bortezomib, carfilzomib/dexamethasone, carfilzomib/dexamethasone/rituximab, dexamethasone/ixazomib/pomalidomide
GFUS	0,00	GDP-L-fucose synthase	Plasma Membrane	enzyme	
WDR75	0,00	WD repeat domain 75	Nucleus	other	
IPO5	0,00	importin 5	Nucleus	transporter	
AK3	0,00	adenylate kinase 3	Cytoplasm	kinase	
UCHL5	0,00	ubiquitin C-terminal hydrolase L5	Cytoplasm	peptidase	VLX1570
CAPRIN1	0,00	cell cycle associated protein 1	Cytoplasm	translation regulator	
VAMP8	0,00	vesicle associated membrane protein 8	Plasma Membrane	transporter	
CPSF3	0,00	cleavage and polyadenylation specific factor 3	Nucleus	enzyme	
SKIC3	0,00	SKI3 subunit of superkiller complex	Nucleus	other	
UTP18	0,00	UTP18 small subunit processome component	Nucleus	other	
SUPT5H	0,00	SPT5 homolog, DSIF elongation factor subunit	Nucleus	transcription regulator	
SARNP	0,00	SAP domain containing ribonucleoprotein	Nucleus	transcription regulator	
HMGB1	0,00	high mobility group box 1	Nucleus	transcription regulator	
KLC1	0,00	kinesin light chain 1	Cytoplasm	other	
SEC31A	0,00	SEC31 homolog A, COPII coat complex component	Cytoplasm	other	
ANXA1	0,00	annexin A1	Plasma Membrane	enzyme	hydrocortisone, hydrocortisone/prednisone, hydrocortisone/mitoxantrone, cytarabine/hydrocortisone/methotrexate, dasatinib/hydrocortisone, hydrocortisone/imatinib, acyclovir/hydrocortisone
APMAP	0,00	adipocyte plasma membrane associated protein	Plasma Membrane	enzyme	
RHOC	0,00	ras homolog family member C	Plasma Membrane	enzyme	
TJP1	0,00	tight junction protein 1	Plasma Membrane	other	
H2AZ2	0,00	H2A.Z variant histone 2	Nucleus	other	
DNAJC8	0,00	DnaJ heat shock protein family (Hsp40) member C8	Nucleus	other	
MRPL44	0,00	mitochondrial ribosomal protein L44	Cytoplasm	enzyme	
NDUFB6	0,00	NADH:ubiquinone oxidoreductase subunit B6	Cytoplasm	enzyme	
GOLGB1	0,00	golgin B1	Cytoplasm	other	
PABPC4	0,00	poly(A) binding protein cytoplasmic 4	Cytoplasm	translation regulator	
SLC25A4	0,00	solute carrier family 25 member 4	Cytoplasm	transporter	clodronic acid
VAPB	0,00	VAMP associated protein B and C	Plasma Membrane	other	

VASP	0,00	vasodilator stimulated phosphoprotein	Plasma Membrane	other	
CSE1L	0,00	chromosome segregation 1 like	Nucleus	transporter	
ETFDH	0,00	electron transfer flavoprotein dehydrogenase	Cytoplasm	enzyme	
USO1	0,00	USO1 vesicle transport factor	Cytoplasm	other	
RDX	0,00	radixin	Cytoplasm	other	
UCHL3	0,00	ubiquitin C-terminal hydrolase L3	Cytoplasm	peptidase	
VPS29	0,00	VPS29 retromer complex component	Cytoplasm	transporter	
BCAP31	0,00	B cell receptor associated protein 31	Cytoplasm	transporter	
LGALS3	0,00	galectin 3	Extracellular Space	other	GCS-100, GB1211, GR-MD-02, GM-CT-01
BIN1	0,00	bridging integrator 1	Nucleus	other	
CWC22	0,00	CWC22 spliceosome associated protein homolog	Nucleus	other	
KPNA1	0,00	karyopherin subunit alpha 1	Nucleus	transporter	
HPRT1	0,00	hypoxanthine phosphoribosyltransferase 1	Cytoplasm	enzyme	cytarabine/daunorubicin/thioguanine, 6-mercaptopurine/methotrexate, 6-mercaptopurine, 6-mercaptopurine/vincristine, hydroxyurea/6-mercaptopurine, imatinib/thioguanine, daunorubicin/etoposide/6-mercaptopurine/mitoxantrone/prednisolone/vindesine, L-asparaginase/cyclophosphamide/cytarabine/daunorubicin/6-mercaptopurine/prednisone/vincristine, 6-mercaptopurine/prednisone/thioguanine, cyclophosphamide/cytarabine/6-mercaptopurine, dasatinib/6-mercaptopurine/methotrexate, cytarabine/thioguanine, thioguanine, azathioprine, 6-mercaptopurine/prednisone, 6-mercaptopurine/methotrexate/tretinoin
DBN1	0,00	drebrin 1	Cytoplasm	other	
UBE2K	0,00	ubiquitin conjugating enzyme E2 K	Cytoplasm	transcription regulator	
PNN	0,00	pinin, desmosome associated protein	Plasma Membrane	other	
ATP6V1A	0,00	ATPase H+ transporting V1 subunit A	Plasma Membrane	transporter	bafilomycin A1, bafilomycin b1
DHX30	0,00	DExH-box helicase 30	Nucleus	enzyme	
STAMBP	0,00	STAM binding protein	Nucleus	enzyme	
TARS1	0,00	threonyl-tRNA synthetase 1	Nucleus	enzyme	
NOL9	0,00	nucleolar protein 9	Nucleus	kinase	
SRP72	0,00	signal recognition particle 72	Nucleus	kinase	
SNRPN	0,00	small nuclear ribonucleoprotein polypeptide N	Nucleus	other	
COASY	0,00	Coenzyme A synthase	Cytoplasm	kinase	
MRPS22	0,00	mitochondrial ribosomal protein S22	Cytoplasm	other	
C1QBP	0,00	complement C1q binding protein	Cytoplasm	transcription regulator	
SCP2	0,00	sterol carrier protein 2	Cytoplasm	transporter	
COPG1	0,00	COPI coat complex subunit gamma 1	Cytoplasm	transporter	
ILK	0,00	integrin linked kinase	Plasma Membrane	kinase	OSU-T315, KP-SD-1
AP2B1	0,00	adaptor related protein complex 2 subunit beta 1	Plasma Membrane	transporter	
SMC1A	0,00	structural maintenance of chromosomes 1A	Nucleus	enzyme	
DNAJA2	0,00	DnaJ heat shock protein family (Hsp40) member A2	Nucleus	enzyme	
GAK	0,00	cyclin G associated kinase	Nucleus	kinase	SM1-71
KPNA4	0,00	karyopherin subunit alpha 4	Nucleus	other	
HSD17B10	0,00	hydroxysteroid 17-beta dehydrogenase 10	Cytoplasm	enzyme	
PGM2	0,00	phosphoglucomutase 2	Cytoplasm	enzyme	
LRPPRC	0,00	leucine rich pentatricopeptide repeat containing	Cytoplasm	other	
CELF1	0,00	CUGBP Elav-like family member 1	Cytoplasm	translation regulator	
NUP188	0,00	nucleoporin 188	Nucleus	other	
LUC7L2	0,00	LUC7 like 2, pre-mRNA splicing factor	Nucleus	other	

NFKB2	0,00	nuclear factor kappa B subunit 2	Nucleus	transcription regulator	
MCCC2	0,00	methylcrotonyl-CoA carboxylase subunit 2	Cytoplasm	enzyme	
ACADS	0,00	acyl-CoA dehydrogenase short chain	Cytoplasm	enzyme	
FLNB	0,00	filamin B	Cytoplasm	other	
PSME1	0,00	proteasome activator subunit 1	Cytoplasm	other	
NGDN	0,00	neuroguidin	Cytoplasm	other	
FIS1	0,00	fission, mitochondrial 1	Cytoplasm	other	
CTSB	0,00	cathepsin B	Cytoplasm	peptidase	
CHCHD3	0,00	coiled-coil-helix-coiled-coil-helix domain containing 3	Cytoplasm	transcription regulator	
COPA	0,00	COPI coat complex subunit alpha	Cytoplasm	transporter	
SEC24B	0,00	SEC24 homolog B, COPII coat complex component	Cytoplasm	transporter	
TIMM23	0,00	translocase of inner mitochondrial membrane 23	Cytoplasm	transporter	
ITCH	0,00	itchy E3 ubiquitin protein ligase	Nucleus	enzyme	
FKBP4	0,00	FKBP prolyl isomerase 4	Nucleus	enzyme	
CPSF2	0,00	cleavage and polyadenylation specific factor 2	Nucleus	other	
GSTO1	0,00	glutathione S-transferase omega 1	Cytoplasm	enzyme	
ROCK2	0,00	Rho associated coiled-coil containing protein kinase 2	Cytoplasm	kinase	ripasudil, GSK-269962A, CCT129524, belumosudil, H89, fasudil, RKI 1447
DCTN2	0,00	dynactin subunit 2	Cytoplasm	other	
PSME2	0,00	proteasome activator subunit 2	Cytoplasm	peptidase	
COPS4	0,00	COP9 signalosome subunit 4	Cytoplasm	peptidase	
SEC24C	0,00	SEC24 homolog C, COPII coat complex component	Cytoplasm	transporter	
JUP	0,00	junction plakoglobin	Plasma Membrane	other	
CLTA	0,00	clathrin light chain A	Plasma Membrane	other	
AIMP2	0,00	aminoacyl tRNA synthetase complex interacting multifunctional protein 2	Plasma Membrane	other	
SPTAN1	0,00	spectrin alpha, non-erythrocytic 1	Plasma Membrane	other	
HNRNPUL1	0,00	heterogeneous nuclear ribonucleoprotein U like 1	Nucleus	other	
EMD	0,00	emerin	Nucleus	other	
THRAP3	0,00	thyroid hormone receptor associated protein 3	Nucleus	transcription regulator	
ALDH7A1	0,00	aldehyde dehydrogenase 7 family member A1	Cytoplasm	enzyme	
ACADSB	0,00	acyl-CoA dehydrogenase short/branched chain	Cytoplasm	enzyme	
NDUFB11	0,00	NADH:ubiquinone oxidoreductase subunit B11	Cytoplasm	enzyme	
SURF4	0,00	surfeit 4	Cytoplasm	other	
RCC1	0,00	regulator of chromosome condensation 1	Cytoplasm	other	
PSMD9	0,00	proteasome 26S subunit, non-ATPase 9	Cytoplasm	transcription regulator	
COPE	0,00	COPI coat complex subunit epsilon	Cytoplasm	transporter	
NSUN2	0,00	NOP2/Sun RNA methyltransferase 2	Nucleus	enzyme	
SND1	0,00	staphylococcal nuclease and tudor domain containing 1	Nucleus	transcription regulator	
ME1	0,00	malic enzyme 1	Cytoplasm	enzyme	
ATP6V1F	0,00	ATPase H+ transporting V1 subunit F	Cytoplasm	enzyme	
CLPX	0,00	caseinolytic mitochondrial matrix peptidase chaperone subunit X	Cytoplasm	enzyme	
AARS1	0,00	alanyl-tRNA synthetase 1	Cytoplasm	enzyme	
TNPO3	0,00	transportin 3	Cytoplasm	other	
FAF2	0,00	Fas associated factor family member 2	Cytoplasm	other	
AP2A1	0,00	adaptor related protein complex 2 subunit alpha 1	Cytoplasm	transporter	
CD9	0,00	CD9 molecule	Plasma Membrane	other	KBA1412
LAMTOR1	0,00	late endosomal/lysosomal adaptor, MAPK and MTOR activator 1	Plasma Membrane	other	

HINT1	0,00	histidine triad nucleotide binding protein 1	Nucleus	enzyme	
FEN1	0,00	flap structure-specific endonuclease 1	Nucleus	enzyme	
FIP1L1	0,00	factor interacting with PAPOLA and CPSF1	Nucleus	other	
CPSF7	0,00	cleavage and polyadenylation specific factor 7	Nucleus	other	
XPOT	0,00	exportin for tRNA	Nucleus	other	
WARS1	0,00	tryptophanyl-tRNA synthetase 1	Cytoplasm	enzyme	
MYO1C	0,00	myosin IC	Cytoplasm	enzyme	
APRT	0,00	adenine phosphoribosyltransferase	Cytoplasm	enzyme	
ACSF2	0,00	acyl-CoA synthetase family member 2	Cytoplasm	enzyme	
MTHFD1	0,00	methylenetetrahydrofolate dehydrogenase, cyclohydrolase and formyltetrahydrofolate synthetase 1	Cytoplasm	enzyme	
ALDH4A1	0,00	aldehyde dehydrogenase 4 family member A1	Cytoplasm	enzyme	
GPX4	0,00	glutathione peroxidase 4	Cytoplasm	enzyme	
AHSA1	0,00	activator of HSP90 ATPase activity 1	Cytoplasm	other	
PSMD10	0,00	proteasome 26S subunit, non-ATPase 10	Cytoplasm	transcription regulator	
SCFD1	0,00	sec1 family domain containing 1	Cytoplasm	transporter	
NAPG	0,00	NSF attachment protein gamma	Cytoplasm	transporter	
CD81	0,00	CD81 molecule	Plasma Membrane	other	
XPO5	0,00	exportin 5	Nucleus	other	
SUPT6H	0,00	SPT6 homolog, histone chaperone and transcription elongation factor	Nucleus	transcription regulator	
GLOD4	0,00	glyoxalase domain containing 4	Cytoplasm	enzyme	
ASCC3	0,00	activating signal cointegrator 1 complex subunit 3	Cytoplasm	enzyme	
NDUFA7	0,00	NADH:ubiquinone oxidoreductase subunit A7	Cytoplasm	enzyme	
HK1	0,00	hexokinase 1	Cytoplasm	kinase	
FKBP8	0,00	FKBP prolyl isomerase 8	Cytoplasm	other	
DYNLL2	0,00	dynein light chain LC8-type 2	Cytoplasm	other	
PICALM	0,00	phosphatidylinositol binding clathrin assembly protein	Cytoplasm	other	
CRK	0,00	CRK proto-oncogene, adaptor protein	Cytoplasm	other	
GNB1	0,00	G protein subunit beta 1	Plasma Membrane	other	
VTI1B	0,00	vesicle transport through interaction with t-SNAREs 1B	Plasma Membrane	transporter	
UTP20	0,00	UTP20 small subunit processome component	Nucleus	other	
DCTN4	0,00	dynactin subunit 4	Nucleus	other	
SET	0,00	SET nuclear proto-oncogene	Nucleus	phosphatase	
PML	0,00	PML nuclear body scaffold	Nucleus	transcription regulator	arsenic trioxide
ARFGAP1	0,00	ADP ribosylation factor GTPase activating protein 1	Cytoplasm	enzyme	
CPT2	0,00	carnitine palmitoyltransferase 2	Cytoplasm	enzyme	perhexiline
RAB5C	0,00	RAB5C, member RAS oncogene family	Cytoplasm	enzyme	
RAB10	0,00	RAB10, member RAS oncogene family	Cytoplasm	enzyme	
HK2	0,00	hexokinase 2	Cytoplasm	kinase	tuvatexib
ARHGDIA	0,00	Rho GDP dissociation inhibitor alpha	Cytoplasm	other	
GBF1	0,00	golgi brefeldin A resistant guanine nucleotide exchange factor 1	Cytoplasm	other	
AQR	0,00	aquarius intron-binding spliceosomal factor	Nucleus	enzyme	
FECH	0,00	ferrochelatase	Cytoplasm	enzyme	
GANAB	0,00	glucosidase II alpha subunit	Cytoplasm	enzyme	miglitol
PPID	0,00	peptidylprolyl isomerase D	Cytoplasm	enzyme	

TXNDC5	0,00	thioredoxin domain containing 5	Cytoplasm	enzyme	
ARF5	0,00	ADP ribosylation factor 5	Cytoplasm	enzyme	
MAT2A	0,00	methionine adenosyltransferase 2A	Cytoplasm	enzyme	IDE397, AG-270
ITPR3	0,00	inositol 1,4,5-trisphosphate receptor type 3	Cytoplasm	ion channel	
PRKAR1A	0,00	protein kinase cAMP-dependent type I regulatory subunit alpha	Cytoplasm	kinase	
PCK2	0,00	phosphoenolpyruvate carboxykinase 2, mitochondrial	Cytoplasm	kinase	
DYNC1I2	0,00	dynein cytoplasmic 1 intermediate chain 2	Cytoplasm	other	
ITGAV	0,00	integrin subunit alpha V	Plasma Membrane	transmembrane receptor	abciximab, intetumumab, fluciclatide F 18, etaracizumab, abituzumab, 68Ga-NOTA-BBN-RGD, cilengitide
EXOSC8	0,00	exosome component 8	Nucleus	enzyme	
SMC3	0,00	structural maintenance of chromosomes 3	Nucleus	enzyme	
DEK	0,00	DEK proto-oncogene	Nucleus	transcription regulator	
DDX42	0,00	DEAD-box helicase 42	Cytoplasm	enzyme	
HSD17B4	0,00	hydroxysteroid 17-beta dehydrogenase 4	Cytoplasm	enzyme	
ARF4	0,00	ADP ribosylation factor 4	Cytoplasm	enzyme	
PDK1	0,00	pyruvate dehydrogenase kinase 1	Cytoplasm	kinase	dichloroacetic acid, bis(4-morpholinyl thiocarbonyl)disulfide
HSPH1	0,00	heat shock protein family H (Hsp110) member 1	Cytoplasm	other	
ISCU	0,00	iron-sulfur cluster assembly enzyme	Cytoplasm	other	
SP1	0,00	Sp1 transcription factor	Nucleus	transcription regulator	
SMARCB1	0,00	SWI/SNF related, matrix associated, actin dependent regulator of chromatin, subfamily b, member 1	Nucleus	transcription regulator	
RHOG	0,00	ras homolog family member G	Cytoplasm	enzyme	
RAB2A	0,00	RAB2A, member RAS oncogene family	Cytoplasm	enzyme	
MAPRE1	0,00	microtubule associated protein RP/EB family member 1	Cytoplasm	other	
PLEC	0,00	plectin	Cytoplasm	other	ZB131
GORASP2	0,00	golgi reassembly stacking protein 2	Cytoplasm	other	
HTRA2	0,00	HtrA serine peptidase 2	Cytoplasm	peptidase	
CYFIP1	0,00	cytoplasmic FMR1 interacting protein 1	Cytoplasm	translation regulator	
SNX9	0,00	sorting nexin 9	Cytoplasm	transporter	
SDCBP	0,00	syndecan binding protein	Plasma Membrane	enzyme	
NAMPT	0,00	nicotinamide phosphoribosyltransferase	Extracellular Space	cytokine	OT-82, STF-118804, daporinad, KPT-9274
BANF1	0,00	BAF nuclear assembly factor 1	Nucleus	other	
OS9	0,00	OS9 endoplasmic reticulum lectin	Nucleus	other	
CCAR1	0,00	cell division cycle and apoptosis regulator 1	Nucleus	transcription regulator	
YARS2	0,00	tyrosyl-tRNA synthetase 2	Cytoplasm	enzyme	
HIBADH	0,00	3-hydroxyisobutyrate dehydrogenase	Cytoplasm	enzyme	
OTUB1	0,00	OTU deubiquitinase, ubiquitin aldehyde binding 1	Cytoplasm	enzyme	
NDUFC2	0,00	NADH:ubiquinone oxidoreductase subunit C2	Cytoplasm	enzyme	
BLVRB	0,00	biliverdin reductase B	Cytoplasm	enzyme	
ARPC2	0,00	actin related protein 2/3 complex subunit 2	Cytoplasm	other	
SNX1	0,00	sorting nexin 1	Cytoplasm	transporter	
NUMA1	0,00	nuclear mitotic apparatus protein 1	Nucleus	other	
RPA1	0,00	replication protein A1	Nucleus	other	
KPNA6	0,00	karyopherin subunit alpha 6	Nucleus	other	
PPP5C	0,00	protein phosphatase 5 catalytic subunit	Nucleus	phosphatase	
OSTC	0,00	oligosaccharyltransferase complex non-catalytic subunit	Cytoplasm	enzyme	
RPN2	0,00	ribophorin II	Cytoplasm	enzyme	

ME2	0,00	malic enzyme 2	Cytoplasm	enzyme	
GLRX3	0,00	glutaredoxin 3	Cytoplasm	enzyme	
USP5	0,00	ubiquitin specific peptidase 5	Cytoplasm	peptidase	
USP9X	0,00	ubiquitin specific peptidase 9 X-linked	Plasma Membrane	peptidase	
WDR1	0,00	WD repeat domain 1	Extracellular Space	other	
SART3	0,00	spliceosome associated factor 3, U4/U6 recycling protein	Nucleus	other	
MECR	0,00	mitochondrial trans-2-enoyl-CoA reductase	Cytoplasm	enzyme	
PPAT	0,00	phosphoribosyl pyrophosphate amidotransferase	Cytoplasm	enzyme	L-asparaginase/cyclophosphamide/cytarabine/daunorubicin/6-mercaptopurine/prednisone/vincristine, 6-mercaptopurine/prednisone/thioguanine, cytarabine/daunorubicin/thioguanine, cyclophosphamide/cytarabine/6-mercaptopurine, cytarabine/thioguanine, 6-mercaptopurine, thioguanine, 6-mercaptopurine/vincristine, amidophosphoribosyltransferase inhibitor, 6-mercaptopurine/prednisone, hydroxyurea/6-mercaptopurine, azathioprine, imatinib/thioguanine
TRMT112	0,00	tRNA methyltransferase activator subunit 11-2	Cytoplasm	enzyme	
DNAJC3	0,00	DnaJ heat shock protein family (Hsp40) member C3	Cytoplasm	other	
TPM1	0,00	tropomyosin 1	Cytoplasm	other	
RBM3	0,00	RNA binding motif protein 3	Cytoplasm	other	
PSMD5	0,00	proteasome 26S subunit, non-ATPase 5	Cytoplasm	other	
DYNC1LI1	0,00	dynein cytoplasmic 1 light intermediate chain 1	Cytoplasm	other	
TMED9	0,00	transmembrane p24 trafficking protein 9	Cytoplasm	transporter	
SNX2	0,00	sorting nexin 2	Cytoplasm	transporter	
TIMM44	0,00	translocase of inner mitochondrial membrane 44	Cytoplasm	transporter	
FLOT1	0,00	flotillin 1	Plasma Membrane	other	
WASF2	0,00	WASP family member 2	Plasma Membrane	other	
AP3B1	0,00	adaptor related protein complex 3 subunit beta 1	Plasma Membrane	transporter	
CTBP1	0,00	C-terminal binding protein 1	Nucleus	enzyme	
MCM4	0,00	minichromosome maintenance complex component 4	Nucleus	enzyme	
ADK	0,00	adenosine kinase	Nucleus	kinase	adenosine kinase inhibitor, pegintron/ribavirin, PEG-interferon alfa-2a/ribavirin, nitazoxanide/peginterferon alfa-2a/ribavirin, interferon alfacon-1/ribavirin, IFNA2B/ribavirin, ribavirin
ZW10	0,00	zw10 kinetochore protein	Nucleus	other	
UBA3	0,00	ubiquitin like modifier activating enzyme 3	Cytoplasm	enzyme	
NDUFV3	0,00	NADH:ubiquinone oxidoreductase subunit V3	Cytoplasm	enzyme	
ACSL4	0,00	acyl-CoA synthetase long chain family member 4	Cytoplasm	enzyme	
GBA1	0,00	glucosylceramidase beta 1	Cytoplasm	enzyme	
AARS2	0,00	alanyl-tRNA synthetase 2, mitochondrial	Cytoplasm	enzyme	
ARHGEF7	0,00	Rho guanine nucleotide exchange factor 7	Cytoplasm	other	
EML4	0,00	EMAP like 4	Cytoplasm	other	
CTSZ	0,00	cathepsin Z	Cytoplasm	peptidase	
TMED3	0,00	transmembrane p24 trafficking protein 3	Cytoplasm	transporter	
COG4	0,00	component of oligomeric golgi complex 4	Cytoplasm	transporter	
PPIL1	0,00	peptidylprolyl isomerase like 1	Plasma Membrane	enzyme	
DNAJC5	0,00	DnaJ heat shock protein family (Hsp40) member C5	Plasma Membrane	other	
SREK1	0,00	splicing regulatory glutamic acid and lysine rich protein 1	Nucleus	other	
H1-0	0,00	H1.0 linker histone	Nucleus	other	
SUMO3	0,00	small ubiquitin like modifier 3	Nucleus	other	

PPM1G	0,00	protein phosphatase, Mg2+/Mn2+ dependent 1G	Nucleus	phosphatase	
CAD	0,00	carbamoyl-phosphate synthetase 2, aspartate transcarbamylase, and dihydroorotase	Cytoplasm	enzyme	
MCCC1	0,00	methylcrotonyl-CoA carboxylase subunit 1	Cytoplasm	enzyme	
PCCA	0,00	propionyl-CoA carboxylase subunit alpha	Cytoplasm	enzyme	
PAFAH1B1	0,00	platelet activating factor acetylhydrolase 1b regulatory subunit 1	Cytoplasm	enzyme	
PYGB	0,00	glycogen phosphorylase B	Cytoplasm	enzyme	
MAP2K2	0,00	mitogen-activated protein kinase kinase 2	Cytoplasm	kinase	U0126, pimasertib, binimetinib/vemurafenib, dabrafenib/trametinib, AS703988, dabrafenib/trametinib/vemurafenib, trametinib/vemurafenib, dabrafenib/pembrolizumab/trametinib, SM1-71, docetaxel/selumetinib, TAK 733, binimetinib/cetuximab/encorafenib, binimetinib/encorafenib/panitumumab, mirdametinib, PD184352, cetuximab/dabrafenib/trametinib, binimetinib, PD318088, trametinib, RO4927350, cobimetinib, selumetinib, binimetinib/encorafenib, FCN-159, trametinib/vorinostat
ABI1	0,00	abl interactor 1	Cytoplasm	other	
DNAJC11	0,00	DnaJ heat shock protein family (Hsp40) member C11	Cytoplasm	other	
SSR1	0,00	signal sequence receptor subunit 1	Cytoplasm	other	
ARPC3	0,00	actin related protein 2/3 complex subunit 3	Cytoplasm	other	
ATP6V1B2	0,00	ATPase H+ transporting V1 subunit B2	Cytoplasm	transporter	gallium nitrate
AP2A2	0,00	adaptor related protein complex 2 subunit alpha 2	Cytoplasm	transporter	
GNB2	0,00	G protein subunit beta 2	Plasma Membrane	other	
FLOT2	0,00	flotillin 2	Plasma Membrane	other	
MSH2	0,00	mutS homolog 2	Nucleus	enzyme	
LUC7L	0,00	LUC7 like	Nucleus	other	
LMNB2	0,00	lamin B2	Nucleus	other	
PSIP1	0,00	PC4 and SRSF1 interacting protein 1	Nucleus	transcription regulator	
SARS1	0,00	seryl-tRNA synthetase 1	Cytoplasm	enzyme	
ECHDC1	0,00	ethylmalonyl-CoA decarboxylase 1	Cytoplasm	enzyme	
RAB14	0,00	RAB14, member RAS oncogene family	Cytoplasm	enzyme	
GFPT1	0,00	glutamine--fructose-6-phosphate transaminase 1	Cytoplasm	enzyme	
MYL6	0,00	myosin light chain 6	Cytoplasm	enzyme	
NFS1	0,00	NFS1 cysteine desulfurase	Cytoplasm	enzyme	
TXNL1	0,00	thioredoxin like 1	Cytoplasm	enzyme	
TWF1	0,00	twinfilin actin binding protein 1	Cytoplasm	kinase	
AP1G1	0,00	adaptor related protein complex 1 subunit gamma 1	Cytoplasm	other	
DYNC1LI2	0,00	dynein cytoplasmic 1 light intermediate chain 2	Cytoplasm	other	
SEL1L	0,00	SEL1L adaptor subunit of SYVN1 ubiquitin ligase	Cytoplasm	other	
UPF2	0,00	UPF2 regulator of nonsense mediated mRNA decay	Cytoplasm	other	
HIP1R	0,00	huntingtin interacting protein 1 related	Cytoplasm	other	
GIT1	0,00	GIT ArfGAP 1	Cytoplasm	other	
RRBP1	0,00	ribosome binding protein 1	Cytoplasm	other	
TIMM50	0,00	translocase of inner mitochondrial membrane 50	Cytoplasm	phosphatase	
YWHAH	0,00	tyrosine 3-monooxygenase/tryptophan 5-monooxygenase activation protein eta	Cytoplasm	transcription regulator	
VPS26A	0,00	VPS26 retromer complex component A	Cytoplasm	transporter	
SEC23A	0,00	SEC23 homolog A, COPII coat complex component	Cytoplasm	transporter	
STOML2	0,00	stomatin like 2	Plasma Membrane	other	

PNP	0,00	purine nucleoside phosphorylase	Nucleus	enzyme	forodesine, PD 141955, purine nucleoside phosphorylase inhibitor
SMARCC1	0,00	SWI/SNF related, matrix associated, actin dependent regulator of chromatin subfamily c member 1	Nucleus	transcription regulator	
NAE1	0,00	NEDD8 activating enzyme E1 subunit 1	Cytoplasm	enzyme	
MOGS	0,00	mannosyl-oligosaccharide glucosidase	Cytoplasm	enzyme	
UBA2	0,00	ubiquitin like modifier activating enzyme 2	Cytoplasm	enzyme	
FDXR	0,00	ferredoxin reductase	Cytoplasm	enzyme	
CSDE1	0,00	cold shock domain containing E1	Cytoplasm	enzyme	
ITPA	0,00	inosine triphosphatase	Cytoplasm	enzyme	
ACSL3	0,00	acyl-CoA synthetase long chain family member 3	Cytoplasm	enzyme	
CSK	0,00	C-terminal Src kinase	Cytoplasm	kinase	SC-204303, bosutinib, bosutinib/rituximab, bosutinib/imatinib
STRN	0,00	striatin	Cytoplasm	other	
SGTA	0,00	small glutamine rich tetratricopeptide repeat co-chaperone alpha	Cytoplasm	other	
PPP1R12A	0,00	protein phosphatase 1 regulatory subunit 12A	Cytoplasm	phosphatase	
CTNNA1	0,00	catenin alpha 1	Plasma Membrane	other	
BSG	0,00	basigin (Ok blood group)	Plasma Membrane	transporter	metuximab
PPIL2	0,00	peptidylprolyl isomerase like 2	Nucleus	enzyme	
DDX19A	0,00	DEAD-box helicase 19A	Nucleus	enzyme	
NAP1L1	0,00	nucleosome assembly protein 1 like 1	Nucleus	other	
NUTF2	0,00	nuclear transport factor 2	Nucleus	other	
CBX3	0,00	chromobox 3	Nucleus	transcription regulator	
ZNF326	0,00	zinc finger protein 326	Nucleus	transcription regulator	
PGM3	0,00	phosphoglucomutase 3	Cytoplasm	enzyme	
ECH1	0,00	enoyl-CoA hydratase 1	Cytoplasm	enzyme	
ARHGAP1	0,00	Rho GTPase activating protein 1	Cytoplasm	other	
COPS3	0,00	COP9 signalosome subunit 3	Cytoplasm	other	
AP1B1	0,00	adaptor related protein complex 1 subunit beta 1	Cytoplasm	other	
ATP6V1E1	0,00	ATPase H+ transporting V1 subunit E1	Cytoplasm	transporter	
EIF2D	0,00	eukaryotic translation initiation factor 2D	Cytoplasm	transporter	
COG7	0,00	component of oligomeric golgi complex 7	Cytoplasm	transporter	
SLC25A11	0,00	solute carrier family 25 member 11	Cytoplasm	transporter	
MSH6	0,00	mutS homolog 6	Nucleus	enzyme	
ARFGAP2	0,00	ADP ribosylation factor GTPase activating protein 2	Nucleus	other	
KPNA3	0,00	karyopherin subunit alpha 3	Nucleus	transporter	
IVD	0,00	isovaleryl-CoA dehydrogenase	Cytoplasm	enzyme	
COG2	0,00	component of oligomeric golgi complex 2	Cytoplasm	other	
GDI2	0,00	GDP dissociation inhibitor 2	Cytoplasm	other	
ARFGAP3	0,00	ADP ribosylation factor GTPase activating protein 3	Cytoplasm	transporter	
LETM1	0,00	leucine zipper and EF-hand containing transmembrane protein 1	Cytoplasm	transporter	
COG1	0,00	component of oligomeric golgi complex 1	Cytoplasm	transporter	
ATP2A2	0,00	ATPase sarcoplasmic/endoplasmic reticulum Ca2+ transporting 2	Cytoplasm	transporter	
COPZ1	0,00	COPI coat complex subunit zeta 1	Cytoplasm	transporter	
UBAP2L	0,00	ubiquitin associated protein 2 like	Nucleus	other	
PREB	0,00	prolactin regulatory element binding	Nucleus	transcription regulator	
CBX5	0,00	chromobox 5	Nucleus	transcription regulator	
NARS1	0,00	asparaginyl-tRNA synthetase 1	Cytoplasm	enzyme	

SURF1	0,00	SURF1 cytochrome c oxidase assembly factor	Cytoplasm	enzyme	
AKR1B1	0,00	aldo-keto reductase family 1 member B	Cytoplasm	enzyme	sorbinil
NEDD4	0,00	NEDD4 E3 ubiquitin protein ligase	Cytoplasm	enzyme	
GPD2	0,00	glycerol-3-phosphate dehydrogenase 2	Cytoplasm	enzyme	
RPS6KA1	0,00	ribosomal protein S6 kinase A1	Cytoplasm	kinase	APIO-EE-07, BI-D1870, PMD-026, LJH685
STAM	0,00	signal transducing adaptor molecule	Cytoplasm	other	
ADD3	0,00	adducin 3	Cytoplasm	other	
TFG	0,00	trafficking from ER to golgi regulator	Cytoplasm	other	
COPS2	0,00	COP9 signalosome subunit 2	Cytoplasm	other	
EIF2A	0,00	eukaryotic translation initiation factor 2A	Cytoplasm	translation regulator	
SNX5	0,00	sorting nexin 5	Cytoplasm	transporter	
ITGB4	0,00	integrin subunit beta 4	Plasma Membrane	transmembrane receptor	
DPYSL2	0,00	dihydropyrimidinase like 2	Cytoplasm	enzyme	
DECR1	0,00	2,4-dienoyl-CoA reductase 1	Cytoplasm	enzyme	
GUK1	0,00	guanylate kinase 1	Cytoplasm	kinase	
CRKL	0,00	CRK like proto-oncogene, adaptor protein	Cytoplasm	kinase	
LMAN2	0,00	lectin, mannose binding 2	Cytoplasm	transporter	
GNAQ	0,00	G protein subunit alpha q	Plasma Membrane	enzyme	
SEC23B	0,00	SEC23 homolog B, COPII coat complex component	Extracellular Space	transporter	
NELFCD	0,00	negative elongation factor complex member C/D	Nucleus	other	
PRDX5	0,00	peroxiredoxin 5	Cytoplasm	enzyme	auranofin
GSTP1	0,00	glutathione S-transferase pi 1	Cytoplasm	enzyme	zeaxanthin
OXCT1	0,00	3-oxoacid CoA-transferase 1	Cytoplasm	enzyme	
GCLC	0,00	glutamate-cysteine ligase catalytic subunit	Cytoplasm	enzyme	
UBA6	0,00	ubiquitin like modifier activating enzyme 6	Cytoplasm	enzyme	
CLINT1	0,00	clathrin interactor 1	Cytoplasm	other	
SCRIB	0,00	scribble planar cell polarity protein	Cytoplasm	other	
AP1M1	0,00	adaptor related protein complex 1 subunit mu 1	Cytoplasm	transporter	
NCKAP1	0,00	NCK associated protein 1	Plasma Membrane	other	
STX7	0,00	syntaxin 7	Plasma Membrane	transporter	
PDS5A	0,00	PDS5 cohesin associated factor A	Nucleus	other	
CTBP2	0,00	C-terminal binding protein 2	Nucleus	transcription regulator	
ALDOC	0,00	aldolase, fructose-bisphosphate C	Cytoplasm	enzyme	
CPT1A	0,00	carnitine palmitoyltransferase 1A	Cytoplasm	enzyme	perhexiline
UGGT1	0,00	UDP-glucose glycoprotein glucosyltransferase 1	Cytoplasm	enzyme	
BCKDK	0,00	branched chain keto acid dehydrogenase kinase	Cytoplasm	kinase	BT2F, BT2
SDF2L1	0,00	stromal cell derived factor 2 like 1	Cytoplasm	other	
NUFIP2	0,00	nuclear FMR1 interacting protein 2	Cytoplasm	other	
EXOC7	0,00	exocyst complex component 7	Cytoplasm	transporter	
COPG2	0,00	COPI coat complex subunit gamma 2	Cytoplasm	transporter	
TIMM13	0,00	translocase of inner mitochondrial membrane 13	Cytoplasm	transporter	
STX4	0,00	syntaxin 4	Plasma Membrane	transporter	
UBE2L3	0,00	ubiquitin conjugating enzyme E2 L3	Nucleus	enzyme	
MCM6	0,00	minichromosome maintenance complex component 6	Nucleus	enzyme	
PRKAG1	0,00	protein kinase AMP-activated non-catalytic subunit gamma 1	Nucleus	kinase	
AFDN	0,00	afadin, adherens junction formation factor	Nucleus	other	
GPS1	0,00	G protein pathway suppressor 1	Nucleus	other	

GTF2F1	0,00	general transcription factor IIF subunit 1	Nucleus	transcription regulator	
HDLBP	0,00	high density lipoprotein binding protein	Nucleus	transporter	
DERA	0,00	deoxyribose-phosphate aldolase	Cytoplasm	enzyme	
ACSL5	0,00	acyl-CoA synthetase long chain family member 5	Cytoplasm	enzyme	
RALA	0,00	RAS like proto-oncogene A	Cytoplasm	enzyme	
RAP1B	0,00	RAP1B, member of RAS oncogene family	Cytoplasm	enzyme	
RAB18	0,00	RAB18, member RAS oncogene family	Cytoplasm	enzyme	
ALDH6A1	0,00	aldehyde dehydrogenase 6 family member A1	Cytoplasm	enzyme	
GOLGA5	0,00	golgin A5	Cytoplasm	kinase	
NME3	0,00	NME/NM23 nucleoside diphosphate kinase 3	Cytoplasm	kinase	
TAGLN2	0,00	transgelin 2	Cytoplasm	other	
ATP5MK	0,00	ATP synthase membrane subunit k	Cytoplasm	other	
ARHGEF2	0,00	Rho/Rac guanine nucleotide exchange factor 2	Cytoplasm	other	paclitaxel/topotecan, bevacizumab/paclitaxel, paclitaxel, lapatinib/paclitaxel, afatinib/paclitaxel, paclitaxel/pertuzumab/trastuzumab, paclitaxel/rituximab, bevacizumab/paclitaxel/topotecan, bortezomib/paclitaxel, paclitaxel/ramucirumab, docetaxel/paclitaxel, paclitaxel/trastuzumab, bevacizumab/paclitaxel/pemetrexed, neratinib/paclitaxel, epirubicin/paclitaxel, atezolizumab/paclitaxel, everolimus/paclitaxel
SLIRP	0,00	SRA stem-loop interacting RNA binding protein	Cytoplasm	other	
ARPC1B	0,00	actin related protein 2/3 complex subunit 1B	Cytoplasm	other	
EIF2B3	0,00	eukaryotic translation initiation factor 2B subunit gamma	Cytoplasm	translation regulator	
SLC25A1	0,00	solute carrier family 25 member 1	Cytoplasm	transporter	
AP1S1	0,00	adaptor related protein complex 1 subunit sigma 1	Cytoplasm	transporter	
SPTBN1	0,00	spectrin beta, non-erythrocytic 1	Plasma Membrane	other	
KLC4	0,00	kinesin light chain 4	Other	other	
DAZAP1	0,00	DAZ associated protein 1	Other	other	
PUS7	0,00	pseudouridine synthase 7	Nucleus	enzyme	
SUB1	0,00	SUB1 regulator of transcription	Nucleus	transcription regulator	
UBE4B	0,00	ubiquitination factor E4B	Cytoplasm	enzyme	
MYO6	0,00	myosin VI	Cytoplasm	other	
STMN1	0,00	stathmin 1	Cytoplasm	other	
SNX27	0,00	sorting nexin 27	Cytoplasm	other	
AKAP9	0,00	A-kinase anchoring protein 9	Cytoplasm	other	
SCO1	0,00	synthesis of cytochrome C oxidase 1	Cytoplasm	other	
CAND1	0,00	cullin associated and neddylation dissociated 1	Cytoplasm	transcription regulator	
SNX4	0,00	sorting nexin 4	Cytoplasm	transporter	
EXOC2	0,00	exocyst complex component 2	Cytoplasm	transporter	
M6PR	0,00	mannose-6-phosphate receptor, cation dependent	Cytoplasm	transporter	alglucosidase alfa
SEC24A	0,00	SEC24 homolog A, COPII coat complex component	Cytoplasm	transporter	
TJP2	0,00	tight junction protein 2	Plasma Membrane	kinase	
LGALS4	0,00	galectin 4	Extracellular Space	other	
MRE11	0,00	MRE11 homolog, double strand break repair nuclease	Nucleus	enzyme	
XRCC1	0,00	X-ray repair cross complementing 1	Nucleus	other	
EMC2	0,00	ER membrane protein complex subunit 2	Nucleus	other	
MACROH2A1	0,00	macroH2A.1 histone	Nucleus	other	
UBTF	0,00	upstream binding transcription factor	Nucleus	transcription regulator	

ASL	0,00	argininosuccinate lyase	Cytoplasm	enzyme	
SAE1	0,00	SUMO1 activating enzyme subunit 1	Cytoplasm	enzyme	
SAR1B	0,00	secretion associated Ras related GTPase 1B	Cytoplasm	enzyme	
MRPS31	0,00	mitochondrial ribosomal protein S31	Cytoplasm	other	
EDC3	0,00	enhancer of mRNA decapping 3	Cytoplasm	other	
TRIP10	0,00	thyroid hormone receptor interactor 10	Cytoplasm	other	
ADD1	0,00	adducin 1	Cytoplasm	other	
BAG2	0,00	BAG cochaperone 2	Cytoplasm	other	
PLIN3	0,00	perilipin 3	Cytoplasm	other	
API5	0,00	apoptosis inhibitor 5	Cytoplasm	other	
USP15	0,00	ubiquitin specific peptidase 15	Cytoplasm	peptidase	
EXOC4	0,00	exocyst complex component 4	Cytoplasm	transporter	
GNAS	0,00	GNAS complex locus	Plasma Membrane	enzyme	
ZYX	0,00	zyxin	Plasma Membrane	other	
CD47	0,00	CD47 molecule	Plasma Membrane	transmembrane receptor	lemzoparlimab, magrolimab, TTI-622, BAT7104, AO-176, PT886, ALX148, gentulizumab, VT1021, CPO107, PF-07257876, IBI322, anti-CD47 monoclonal antibody, CC-90002, MIL95, ZL-1201, NI-1801, TG-1801, STI-6643, IMM2902, 6MW3211, IMC-002, SGN-CD47M, recombinant fusion protein IMM01, HX009, IMM0306, DSP107, letaplimab, TTI-621, SL-172154
MIF	0,00	macrophage migration inhibitory factor	Extracellular Space	cytokine	imalumab
IDE	0,00	insulin degrading enzyme	Extracellular Space	peptidase	bacitracin
RNF20	0,00	ring finger protein 20	Nucleus	enzyme	
BRD2	0,00	bromodomain containing 2	Nucleus	kinase	JAB-8263, BI 894999
RBM15	0,00	RNA binding motif protein 15	Nucleus	other	
SARS2	0,00	seryl-tRNA synthetase 2, mitochondrial	Cytoplasm	enzyme	
GLS	0,00	glutaminase	Cytoplasm	enzyme	telaglenastat
LMO7	0,00	LIM domain 7	Cytoplasm	enzyme	
GCSH	0,00	glycine cleavage system protein H	Cytoplasm	enzyme	
EPS8	0,00	epidermal growth factor receptor pathway substrate 8	Cytoplasm	other	
PLAA	0,00	phospholipase A2 activating protein	Cytoplasm	other	
CALU	0,00	calumenin	Cytoplasm	other	
GRSF1	0,00	G-rich RNA sequence binding factor 1	Cytoplasm	other	
ARPC5	0,00	actin related protein 2/3 complex subunit 5	Cytoplasm	other	
EDC4	0,00	enhancer of mRNA decapping 4	Cytoplasm	other	
GDI1	0,00	GDP dissociation inhibitor 1	Cytoplasm	other	
SERPINH1	0,00	serpin family H member 1	Extracellular Space	other	
FKBP5	0,00	FKBP prolyl isomerase 5	Nucleus	enzyme	
GALE	0,00	UDP-galactose-4-epimerase	Cytoplasm	enzyme	
CAT	0,00	catalase	Cytoplasm	enzyme	fomepizole
RAB5B	0,00	RAB5B, member RAS oncogene family	Cytoplasm	enzyme	
ILVBL	0,00	ilvB acetolactate synthase like	Cytoplasm	enzyme	
HSD17B12	0,00	hydroxysteroid 17-beta dehydrogenase 12	Cytoplasm	enzyme	
MTHFD1L	0,00	methylenetetrahydrofolate dehydrogenase (NADP+ dependent) 1 like	Cytoplasm	enzyme	
ERLEC1	0,00	endoplasmic reticulum lectin 1	Cytoplasm	other	
COQ9	0,00	coenzyme Q9	Cytoplasm	other	
SEC23IP	0,00	SEC23 interacting protein	Cytoplasm	other	
NSFL1C	0,00	NSFL1 cofactor	Cytoplasm	other	
MTCH2	0,00	mitochondrial carrier 2	Cytoplasm	other	
CCAR2	0,00	cell cycle and apoptosis regulator 2	Cytoplasm	peptidase	
ATOX1	0,00	antioxidant 1 copper chaperone	Cytoplasm	transporter	

ATP6V0D1	0,00	ATPase H+ transporting V0 subunit d1	Cytoplasm	transporter	
BAX	0,00	BCL2 associated X, apoptosis regulator	Cytoplasm	transporter	
GNA13	0,00	G protein subunit alpha 13	Plasma Membrane	enzyme	
GNG12	0,00	G protein subunit gamma 12	Plasma Membrane	enzyme	
EMC1	0,00	ER membrane protein complex subunit 1	Plasma Membrane	other	
NIPSNAP2	0,00	nipsnap homolog 2	Plasma Membrane	other	
PLIN2	0,00	perilipin 2	Plasma Membrane	other	
ITGA6	0,00	integrin subunit alpha 6	Plasma Membrane	transmembrane receptor	
PRPSAP2	0,00	phosphoribosyl pyrophosphate synthetase associated protein 2	Other	other	
PURA	0,00	purine rich element binding protein A	Nucleus	transcription regulator	
HCFC1	0,00	host cell factor C1	Nucleus	transcription regulator	
DPM1	0,00	dolichyl-phosphate mannosyltransferase subunit 1, catalytic	Cytoplasm	enzyme	
SSR4	0,00	signal sequence receptor subunit 4	Cytoplasm	other	
COPS7A	0,00	COP9 signalosome subunit 7A	Cytoplasm	other	
CTSC	0,00	cathepsin C	Cytoplasm	peptidase	
EEF1E1	0,00	eukaryotic translation elongation factor 1 epsilon 1	Cytoplasm	translation regulator	
SEC62	0,00	SEC62 homolog, preprotein translocation factor	Cytoplasm	transporter	
EPS15L1	0,00	epidermal growth factor receptor pathway substrate 15 like 1	Plasma Membrane	other	
HNRNPLL	0,00	heterogeneous nuclear ribonucleoprotein L like	Plasma Membrane	other	
MCM2	0,00	minichromosome maintenance complex component 2	Nucleus	enzyme	
MTAP	0,00	methylthioadenosine phosphorylase	Nucleus	enzyme	
TSN	0,00	translin	Nucleus	other	
CTNND1	0,00	catenin delta 1	Nucleus	other	
OGT	0,00	O-linked N-acetylglucosamine (GlcNAc) transferase	Cytoplasm	enzyme	
CD2AP	0,00	CD2 associated protein	Cytoplasm	other	
CISD2	0,00	CDGSH iron sulfur domain 2	Cytoplasm	other	
RTN4	0,00	reticulon 4	Cytoplasm	other	
MRPS27	0,00	mitochondrial ribosomal protein S27	Cytoplasm	other	
EXOC3	0,00	exocyst complex component 3	Plasma Membrane	transporter	
APOO	0,00	apolipoprotein O	Extracellular Space	other	
PRPSAP1	0,00	phosphoribosyl pyrophosphate synthetase associated protein 1	Extracellular Space	other	
EXOSC6	0,00	exosome component 6	Nucleus	other	
NELFE	0,00	negative elongation factor complex member E	Nucleus	other	
NBAS	0,00	NBAS subunit of NRZ tethering complex	Nucleus	other	
MAOA	0,00	monoamine oxidase A	Cytoplasm	enzyme	fenfluramine/phentermine, moclobemide, methamphetamine, isocarboxazid, CX-1370, benzphetamine, phentermine/topiramate, N-(2-indanyl)glycinamide, iproniazid, phentermine, tranylcypromine, procainamide, MAO-A inhibitor, ladostigil, phenelzine, dextroamphetamine
PPIL3	0,00	peptidylprolyl isomerase like 3	Cytoplasm	enzyme	
MMUT	0,00	methylmalonyl-CoA mutase	Cytoplasm	enzyme	hydroxocobalamin, cyanocobalamin
L2HGDH	0,00	L-2-hydroxyglutarate dehydrogenase	Cytoplasm	enzyme	
GIGYF2	0,00	GRB10 interacting GYF protein 2	Cytoplasm	other	
CORO1C	0,00	coronin 1C	Cytoplasm	other	
PDCD6	0,00	programmed cell death 6	Cytoplasm	other	
BAIAP2L1	0,00	BAR/IMD domain containing adaptor protein 2 like 1	Cytoplasm	other	

CTSA	0,00	cathepsin A	Cytoplasm	peptidase	
PPP3CA	0,00	protein phosphatase 3 catalytic subunit alpha	Cytoplasm	phosphatase	voclosporin, tacrolimus, prednisone/tacrolimus, methylprednisolone/tacrolimus, pimecrolimus, cyclosporin A/methotrexate, cyclosporin A
MTX2	0,00	metaxin 2	Cytoplasm	transporter	
SLC25A24	0,00	solute carrier family 25 member 24	Cytoplasm	transporter	
ERP29	0,00	endoplasmic reticulum protein 29	Cytoplasm	transporter	
ITGA2	0,00	integrin subunit alpha 2	Plasma Membrane	transmembrane receptor	
RPA3	0,00	replication protein A3	Nucleus	other	
AGFG1	0,00	ArfGAP with FG repeats 1	Nucleus	other	
DIDO1	0,00	death inducer-obliterator 1	Nucleus	other	
CBFB	0,00	core-binding factor subunit beta	Nucleus	transcription regulator	
BAZ1B	0,00	bromodomain adjacent to zinc finger domain 1B	Nucleus	transcription regulator	
ERO1A	0,00	endoplasmic reticulum oxidoreductase 1 alpha	Cytoplasm	enzyme	
DOHH	0,00	deoxyhypusine hydroxylase	Cytoplasm	enzyme	
GCLM	0,00	glutamate-cysteine ligase modifier subunit	Cytoplasm	enzyme	
ERP44	0,00	endoplasmic reticulum protein 44	Cytoplasm	enzyme	
MICOS13	0,00	mitochondrial contact site and cristae organizing system subunit 13	Cytoplasm	other	
ANP32A	0,00	acidic nuclear phosphoprotein 32 family member A	Cytoplasm	other	
ARHGEF12	0,00	Rho guanine nucleotide exchange factor 12	Cytoplasm	other	
ACP1	0,00	acid phosphatase 1	Cytoplasm	phosphatase	
SLC25A13	0,00	solute carrier family 25 member 13	Cytoplasm	transporter	
ATP6V1D	0,00	ATPase H+ transporting V1 subunit D	Cytoplasm	transporter	
TCIRG1	0,00	T cell immune regulator 1, ATPase H+ transporting V0 subunit a3	Plasma Membrane	enzyme	
STIM1	0,00	stromal interaction molecule 1	Plasma Membrane	ion channel	
ADAM10	0,00	ADAM metallopeptidase domain 10	Plasma Membrane	peptidase	aderbasib
AHNAK	0,00	AHNAK nucleoprotein	Nucleus	other	
IPO9	0,00	importin 9	Nucleus	other	
SON	0,00	SON DNA and RNA binding protein	Nucleus	other	
DPY30	0,00	dpy-30 histone methyltransferase complex regulatory subunit	Nucleus	other	
GLYR1	0,00	glyoxylate reductase 1 homolog	Nucleus	other	
TRRAP	0,00	transformation/transcription domain associated protein	Nucleus	transcription regulator	
SMARCC2	0,00	SWI/SNF related, matrix associated, actin dependent regulator of chromatin subfamily c member 2	Nucleus	transcription regulator	
RHOT1	0,00	ras homolog family member T1	Cytoplasm	enzyme	
RAB35	0,00	RAB35, member RAS oncogene family	Cytoplasm	enzyme	
UBE4A	0,00	ubiquitination factor E4A	Cytoplasm	enzyme	
PRKCSH	0,00	protein kinase C substrate 80K-H	Cytoplasm	enzyme	
GOLGA4	0,00	golgin A4	Cytoplasm	other	
PTCD3	0,00	pentatricopeptide repeat domain 3	Cytoplasm	other	
VPS53	0,00	VPS53 subunit of GARP complex	Cytoplasm	other	
SH3GL1	0,00	SH3 domain containing GRB2 like 1, endophilin A2	Cytoplasm	other	
STX12	0,00	syntaxin 12	Cytoplasm	other	
VCPIP1	0,00	valosin containing protein interacting protein 1	Cytoplasm	peptidase	
EIF2B1	0,00	eukaryotic translation initiation factor 2B subunit alpha	Cytoplasm	translation regulator	
NHERF1	0,00	NHERF family PDZ scaffold protein 1	Plasma Membrane	transporter	

NASP	0,00	nuclear autoantigenic sperm protein	Nucleus	other	
PSPC1	0,00	paraspeckle component 1	Nucleus	transcription regulator	
TNPO2	0,00	transportin 2	Nucleus	transporter	
GET3	0,00	guided entry of tail-anchored proteins factor 3, ATPase	Nucleus	transporter	
RRAGC	0,00	Ras related GTP binding C	Cytoplasm	enzyme	
AGK	0,00	acylglycerol kinase	Cytoplasm	kinase	SM1-71
AUP1	0,00	AUP1 lipid droplet regulating VLDL assembly factor	Cytoplasm	other	
ERLIN2	0,00	ER lipid raft associated 2	Cytoplasm	other	
DBNL	0,00	drebrin like	Cytoplasm	other	
RER1	0,00	retention in endoplasmic reticulum sorting receptor 1	Cytoplasm	other	
RAB3GAP1	0,00	RAB3 GTPase activating protein catalytic subunit 1	Cytoplasm	other	
LAMTOR2	0,00	late endosomal/lysosomal adaptor, MAPK and MTOR activator 2	Cytoplasm	other	
CAPN1	0,00	calpain 1	Cytoplasm	peptidase	alicapistat, BLD-2660
CTSL	0,00	cathepsin L	Cytoplasm	peptidase	cathepsin L inhibitor
ATP6V1H	0,00	ATPase H+ transporting V1 subunit H	Cytoplasm	transporter	
VPS28	0,00	VPS28 subunit of ESCRT-I	Cytoplasm	transporter	
EXOC1	0,00	exocyst complex component 1	Cytoplasm	transporter	
LRP1	0,00	LDL receptor related protein 1	Plasma Membrane	transmembrane receptor	paclitaxel-angiopep-2 conjugate, serpin peptide 16
SLC3A2	0,00	solute carrier family 3 member 2	Plasma Membrane	transporter	
SDF2	0,00	stromal cell derived factor 2	Extracellular Space	enzyme	
DUT	0,00	deoxyuridine triphosphatase	Nucleus	enzyme	TAS-114
H1-2	0,00	H1.2 linker histone, cluster member	Nucleus	other	
NAA15	0,00	N-alpha-acetyltransferase 15, NatA auxiliary subunit	Nucleus	transcription regulator	
TXNDC12	0,00	thioredoxin domain containing 12	Cytoplasm	enzyme	
RRAS	0,00	RAS related	Cytoplasm	enzyme	
P4HA1	0,00	prolyl 4-hydroxylase subunit alpha 1	Cytoplasm	enzyme	
ALG5	0,00	ALG5 dolichyl-phosphate beta-glucosyltransferase	Cytoplasm	enzyme	
SAR1A	0,00	secretion associated Ras related GTPase 1A	Cytoplasm	enzyme	
PEX5	0,00	peroxisomal biogenesis factor 5	Cytoplasm	other	
ARFGEF2	0,00	ADP ribosylation factor guanine nucleotide exchange factor 2	Cytoplasm	other	
CHMP4B	0,00	charged multivesicular body protein 4B	Cytoplasm	other	
KIF2A	0,00	kinesin family member 2A	Cytoplasm	other	
CCDC22	0,00	coiled-coil domain containing 22	Cytoplasm	other	
HM13	0,00	histocompatibility minor 13	Cytoplasm	peptidase	
PPP2R2A	0,00	protein phosphatase 2 regulatory subunit Balpha	Cytoplasm	phosphatase	
STRAP	0,00	serine/threonine kinase receptor associated protein	Plasma Membrane	other	
UGDH	0,00	UDP-glucose 6-dehydrogenase	Nucleus	enzyme	
SUN2	0,00	Sad1 and UNC84 domain containing 2	Nucleus	other	
AHCTF1	0,00	AT-hook containing transcription factor 1	Nucleus	transcription regulator	
FUBP3	0,00	far upstream element binding protein 3	Nucleus	transcription regulator	
CYB5A	0,00	cytochrome b5 type A	Cytoplasm	enzyme	
MAN2A1	0,00	mannosidase alpha class 2A member 1	Cytoplasm	enzyme	
SRM	0,00	spermidine synthase	Cytoplasm	enzyme	
OPA1	0,00	OPA1 mitochondrial dynamin like GTPase	Cytoplasm	enzyme	
TST	0,00	thiosulfate sulfurtransferase	Cytoplasm	enzyme	
PRKAA1	0,00	protein kinase AMP-activated catalytic subunit alpha 1	Cytoplasm	kinase	phenformin
PLS3	0,00	plastin 3	Cytoplasm	other	

FCHO2	0,00	FCH and mu domain containing endocytic adaptor 2	Cytoplasm	other	
VTA1	0,00	vesicle trafficking 1	Cytoplasm	other	
DAD1	0,00	defender against cell death 1	Cytoplasm	other	
LAMTOR3	0,00	late endosomal/lysosomal adaptor, MAPK and MTOR activator 3	Cytoplasm	other	
GOLIM4	0,00	golgi integral membrane protein 4	Cytoplasm	other	
ELP1	0,00	elongator acetyltransferase complex subunit 1	Cytoplasm	other	
TRAPPC3	0,00	trafficking protein particle complex subunit 3	Cytoplasm	other	birabresib
DSTN	0,00	destrin, actin depolymerizing factor	Cytoplasm	other	
USP19	0,00	ubiquitin specific peptidase 19	Cytoplasm	peptidase	
LAP3	0,00	leucine aminopeptidase 3	Cytoplasm	peptidase	
EIF2B2	0,00	eukaryotic translation initiation factor 2B subunit beta	Cytoplasm	translation regulator	
ATP6V0A1	0,00	ATPase H+ transporting V0 subunit a1	Cytoplasm	transporter	
ESYT1	0,00	extended synaptotagmin 1	Cytoplasm	transporter	
AP3M1	0,00	adaptor related protein complex 3 subunit mu 1	Cytoplasm	transporter	
GNAI2	0,00	G protein subunit alpha i2	Plasma Membrane	enzyme	
GNA11	0,00	G protein subunit alpha 11	Plasma Membrane	enzyme	
ELAC2	0,00	elaC ribonuclease Z 2	Nucleus	enzyme	
PBRM1	0,00	polybromo 1	Nucleus	other	
ZMPSTE24	0,00	zinc metallopeptidase STE24	Nucleus	peptidase	
CUX1	0,00	cut like homeobox 1	Nucleus	transcription regulator	
SPTLC1	0,00	serine palmitoyltransferase long chain base subunit 1	Cytoplasm	enzyme	
DARS1	0,00	aspartyl-tRNA synthetase 1	Cytoplasm	enzyme	
ADH5	0,00	alcohol dehydrogenase 5 (class III), chi polypeptide	Cytoplasm	enzyme	N6022
COMT	0,00	catechol-O-methyltransferase	Cytoplasm	enzyme	carbidopa/entacapone/levodopa, nebicapone, opicapone, tolcapone, entacapone, carbidopa/levodopa/tolcapone, tyrphostin AG 1288
VPS51	0,00	VPS51 subunit of GARP complex	Cytoplasm	other	faricimab
DBI	0,00	diazepam binding inhibitor, acyl-CoA binding protein	Cytoplasm	other	
LRRC59	0,00	leucine rich repeat containing 59	Cytoplasm	other	
ARHGEF1	0,00	Rho guanine nucleotide exchange factor 1	Cytoplasm	other	
NUBPL	0,00	NUBP iron-sulfur cluster assembly factor, mitochondrial	Cytoplasm	other	
PFDN4	0,00	prefoldin subunit 4	Cytoplasm	other	
ST13	0,00	ST13 Hsp70 interacting protein	Cytoplasm	other	
KIF3B	0,00	kinesin family member 3B	Cytoplasm	transporter	
ATAD1	0,00	ATPase family AAA domain containing 1	Plasma Membrane	enzyme	
DAG1	0,00	dystroglycan 1	Plasma Membrane	transmembrane receptor	
STXBP2	0,00	syntaxin binding protein 2	Plasma Membrane	transporter	
HSPG2	0,00	heparan sulfate proteoglycan 2	Extracellular Space	enzyme	
PDCD4	0,00	programmed cell death 4	Nucleus	other	
ASH2L	0,00	ASH2 like, histone lysine methyltransferase complex subunit	Nucleus	transcription regulator	
PPT1	0,00	palmitoyl-protein thioesterase 1	Cytoplasm	enzyme	ezurpimtrostat
FKBP2	0,00	FKBP prolyl isomerase 2	Cytoplasm	enzyme	
CBR1	0,00	carbonyl reductase 1	Cytoplasm	enzyme	
MCU	0,00	mitochondrial calcium uniporter	Cytoplasm	ion channel	
CAMK2G	0,00	calcium/calmodulin dependent protein kinase II gamma	Cytoplasm	kinase	
CKB	0,00	creatine kinase B	Cytoplasm	kinase	

YES1	0,00	YES proto-oncogene 1, Src family tyrosine kinase	Cytoplasm	kinase	blinatumomab/dasatinib, dasatinib/hydrocortisone, SC-204303, PD162531, dasatinib/dexamethasone/vincristine, CH6953755, SM1-71, dasatinib/filgrastim, dasatinib/dexamethasone, JNJ-26483327, cytarabine/dasatinib/dexamethasone/methotrexate, corticosteroid/dasatinib, dasatinib/rituximab, cytarabine/dasatinib/filgrastim/fludarabine phosphate, dasatinib/6-mercaptopurine/methotrexate, dasatinib/nilotinib, cytarabine/dasatinib/filgrastim/fludarabine phosphate/idarubicin, dasatinib/erlotinib, dasatinib/prednisone, PD173955, cytarabine/dasatinib, dasatinib/inotuzumab ozogamicin, dasatinib, afatinib/dasatinib
GAPVD1	0,00	GTPase activating protein and VPS9 domains 1	Cytoplasm	other	
TPD52	0,00	tumor protein D52	Cytoplasm	other	
DLG1	0,00	discs large MAGUK scaffold protein 1	Cytoplasm	other	
SLC25A12	0,00	solute carrier family 25 member 12	Cytoplasm	transporter	
ATP6V1G1	0,00	ATPase H+ transporting V1 subunit G1	Cytoplasm	transporter	
SNX17	0,00	sorting nexin 17	Cytoplasm	transporter	
UBR4	0,00	ubiquitin protein ligase E3 component n-recognin 4	Nucleus	enzyme	
PRKAB1	0,00	protein kinase AMP-activated non-catalytic subunit beta 1	Nucleus	kinase	
SUGT1	0,00	SGT1 homolog, MIS12 kinetochore complex assembly cochaperone	Nucleus	other	
ZFR	0,00	zinc finger RNA binding protein	Nucleus	other	
GALM	0,00	galactose mutarotase	Cytoplasm	enzyme	
RAB3GAP2	0,00	RAB3 GTPase activating non-catalytic protein subunit 2	Cytoplasm	enzyme	
CISD1	0,00	CDGSH iron sulfur domain 1	Cytoplasm	enzyme	
NNT	0,00	nicotinamide nucleotide transhydrogenase	Cytoplasm	enzyme	
CRYZ	0,00	crystallin zeta	Cytoplasm	enzyme	
AKR1B10	0,00	aldo-keto reductase family 1 member B10	Cytoplasm	enzyme	
CHMP6	0,00	charged multivesicular body protein 6	Cytoplasm	other	
S100A10	0,00	S100 calcium binding protein A10	Cytoplasm	other	
ACTR1B	0,00	actin related protein 1B	Cytoplasm	other	
PITRM1	0,00	pitrilysin metallopeptidase 1	Cytoplasm	peptidase	
SNX3	0,00	sorting nexin 3	Cytoplasm	transporter	
VPS4B	0,00	vacuolar protein sorting 4 homolog B	Cytoplasm	transporter	
SNX6	0,00	sorting nexin 6	Cytoplasm	transporter	
AGRN	0,00	agrin	Plasma Membrane	other	
EXOC8	0,00	exocyst complex component 8	Plasma Membrane	other	
MANF	0,00	mesencephalic astrocyte derived neurotrophic factor	Extracellular Space	other	
TNKS1BP1	0,00	tankyrase 1 binding protein 1	Nucleus	other	
XPO7	0,00	exportin 7	Nucleus	transporter	
NIT2	0,00	nitrilase family member 2	Cytoplasm	enzyme	
ALDH16A1	0,00	aldehyde dehydrogenase 16 family member A1	Cytoplasm	enzyme	
PGLS	0,00	6-phosphogluconolactonase	Cytoplasm	enzyme	
SQOR	0,00	sulfide quinone oxidoreductase	Cytoplasm	enzyme	
RALB	0,00	RAS like proto-oncogene B	Cytoplasm	enzyme	
CBR4	0,00	carbonyl reductase 4	Cytoplasm	enzyme	
SGPL1	0,00	sphingosine-1-phosphate lyase 1	Cytoplasm	enzyme	
AGPS	0,00	alkylglycerone phosphate synthase	Cytoplasm	enzyme	
MARK2	0,00	microtubule affinity regulating kinase 2	Cytoplasm	kinase	SM1-71
MYO5C	0,00	myosin VC	Cytoplasm	other	

ARFGEF1	0,00	ADP ribosylation factor guanine nucleotide exchange factor 1	Cytoplasm	other	
RMDN3	0,00	regulator of microtubule dynamics 3	Cytoplasm	other	
CKAP4	0,00	cytoskeleton associated protein 4	Cytoplasm	other	
PDLIM7	0,00	PDZ and LIM domain 7	Cytoplasm	other	
EHD1	0,00	EH domain containing 1	Cytoplasm	other	
USP47	0,00	ubiquitin specific peptidase 47	Cytoplasm	peptidase	
TRIP11	0,00	thyroid hormone receptor interactor 11	Cytoplasm	transcription regulator	
DSP	0,00	desmoplakin	Plasma Membrane	other	
UTRN	0,00	utrophin	Plasma Membrane	transmembrane receptor	
LGALS1	0,00	galectin 1	Extracellular Space	other	OTX008
DDRGK1	0,00	DDRGK domain containing 1	Extracellular Space	other	
APOOL	0,00	apolipoprotein O like	Extracellular Space	other	
TCOF1	0,00	treacle ribosome biogenesis factor 1	Nucleus	transcription regulator	
MTA2	0,00	metastasis associated 1 family member 2	Nucleus	transcription regulator	
ARL3	0,00	ADP ribosylation factor like GTPase 3	Cytoplasm	enzyme	
GALNT2	0,00	polypeptide N-acetylgalactosaminyltransferase 2	Cytoplasm	enzyme	
ARL1	0,00	ADP ribosylation factor like GTPase 1	Cytoplasm	enzyme	
CNP	0,00	2',3'-cyclic nucleotide 3' phosphodiesterase	Cytoplasm	enzyme	
MAP2K3	0,00	mitogen-activated protein kinase kinase 3	Cytoplasm	kinase	
CAMK2D	0,00	calcium/calmodulin dependent protein kinase II delta	Cytoplasm	kinase	
MIA3	0,00	MIA SH3 domain ER export factor 3	Cytoplasm	other	
MYO18A	0,00	myosin XVIIIA	Cytoplasm	other	
DNAJC13	0,00	DnaJ heat shock protein family (Hsp40) member C13	Cytoplasm	other	
TBC1D8B	0,00	TBC1 domain family member 8B	Cytoplasm	other	
ABCD3	0,00	ATP binding cassette subfamily D member 3	Cytoplasm	transporter	
ATP6V1C1	0,00	ATPase H+ transporting V1 subunit C1	Cytoplasm	transporter	
NCSTN	0,00	nicastrin	Plasma Membrane	peptidase	
ATP1A1	0,00	ATPase Na+/K+ transporting subunit alpha 1	Plasma Membrane	transporter	trichloromethiazide, perphenazine, ethacrynic acid, ciclopirox olamine, bretylium, reserpine/trichloromethiazide, ouabain, acetyldigitoxin derivative, digoxin, digitoxin
BAG6	0,00	BAG cochaperone 6	Nucleus	enzyme	
RAD50	0,00	RAD50 double strand break repair protein	Nucleus	enzyme	
NEK9	0,00	NIMA related kinase 9	Nucleus	kinase	
NELFB	0,00	negative elongation factor complex member B	Nucleus	other	
ANXA11	0,00	annexin A11	Nucleus	other	
PTBP3	0,00	polypyrimidine tract binding protein 3	Nucleus	other	
OSGEP	0,00	O-sialoglycoprotein endopeptidase	Nucleus	peptidase	
CTDP1	0,00	CTD phosphatase subunit 1	Nucleus	phosphatase	
ESD	0,00	esterase D	Cytoplasm	enzyme	
PMM2	0,00	phosphomannomutase 2	Cytoplasm	enzyme	
PYCR1	0,00	pyrroline-5-carboxylate reductase 1	Cytoplasm	enzyme	
MAN1A1	0,00	mannosidase alpha class 1A member 1	Cytoplasm	enzyme	
HAGH	0,00	hydroxyacylglutathione hydrolase	Cytoplasm	enzyme	

POR	0,00	cytochrome p450 oxidoreductase	Cytoplasm	enzyme	carboplatin/doxorubicin, nitazoxanide, cisplatin/doxorubicin, cisplatin/doxorubicin/ifosfamide, daunorubicin/tretinoin, cisplatin/doxorubicin/methotrexate, daunorubicin, doxorubicin/streptozocin, dacarbazine/doxorubicin, doxorubicin/ifosfamide, cisplatin/doxorubicin/ifosfamide/methotrexate, dacarbazine/doxorubicin/ifosfamide, doxorubicin, doxorubicin/tretinoin, nitazoxanide/peginterferon alfa-2a/ribavirin
FAHD1	0,00	fumarylacetoacetate hydrolase domain containing 1	Cytoplasm	enzyme	
ALG2	0,00	ALG2 alpha-1,3/1,6-mannosyltransferase	Cytoplasm	enzyme	
CLYBL	0,00	citramalyl-CoA lyase	Cytoplasm	enzyme	
AKR1A1	0,00	aldo-keto reductase family 1 member A1	Cytoplasm	enzyme	aldose reductase inhibitor
ETHE1	0,00	ETHE1 persulfide dioxygenase	Cytoplasm	enzyme	
PRKCI	0,00	protein kinase C iota	Cytoplasm	kinase	CRT0066854, myristoylated PKC-zeta pseudosubstrate peptide inhibitor, PKC-iota pseudosubstrate peptide inhibitor, ingenol mebutate
CDC42BPB	0,00	CDC42 binding protein kinase beta	Cytoplasm	kinase	
GALK1	0,00	galactokinase 1	Cytoplasm	kinase	
CHCHD6	0,00	coiled-coil-helix-coiled-coil-helix domain containing 6	Cytoplasm	other	
CORO1B	0,00	coronin 1B	Cytoplasm	other	
ERGIC2	0,00	ERGIC and golgi 2	Cytoplasm	other	
UBXN1	0,00	UBX domain protein 1	Cytoplasm	other	
TOMM6	0,00	translocase of outer mitochondrial membrane 6	Cytoplasm	other	
IGBP1	0,00	immunoglobulin binding protein 1	Cytoplasm	phosphatase	
STXBP3	0,00	syntaxin binding protein 3	Plasma Membrane	transporter	
HDGF	0,00	heparin binding growth factor	Extracellular Space	growth factor	
IPO11	0,00	importin 11	Nucleus	other	
H1-4	0,00	H1.4 linker histone, cluster member	Nucleus	other	
GPD1L	0,00	glycerol-3-phosphate dehydrogenase 1 like	Cytoplasm	enzyme	
CRAT	0,00	carnitine O-acetyltransferase	Cytoplasm	enzyme	
GLB1	0,00	galactosidase beta 1	Cytoplasm	enzyme	
RAB11B	0,00	RAB11B, member RAS oncogene family	Cytoplasm	enzyme	
MPST	0,00	mercaptopyruvate sulfurtransferase	Cytoplasm	enzyme	
ACOT13	0,00	acyl-CoA thioesterase 13	Cytoplasm	enzyme	
PKN2	0,00	protein kinase N2	Cytoplasm	kinase	fasudil
GIPC1	0,00	GIPC PDZ domain containing family member 1	Cytoplasm	other	
MRPS36	0,00	mitochondrial ribosomal protein S36	Cytoplasm	other	
MFF	0,00	mitochondrial fission factor	Cytoplasm	other	
AP3D1	0,00	adaptor related protein complex 3 subunit delta 1	Cytoplasm	other	
CAPNS1	0,00	calpain small subunit 1	Cytoplasm	peptidase	
TPP1	0,00	tripeptidyl peptidase 1	Cytoplasm	peptidase	
LNPEP	0,00	leucyl and cystinyl aminopeptidase	Cytoplasm	peptidase	
MMGT1	0,00	membrane magnesium transporter 1	Cytoplasm	transporter	
ANXA7	0,00	annexin A7	Plasma Membrane	ion channel	
PLS1	0,00	plastin 1	Plasma Membrane	other	
HMGA2	0,00	high mobility group AT-hook 2	Nucleus	enzyme	
SYNE2	0,00	spectrin repeat containing nuclear envelope protein 2	Nucleus	other	
PLCB3	0,00	phospholipase C beta 3	Cytoplasm	enzyme	
SMS	0,00	spermine synthase	Cytoplasm	enzyme	(±)-2-hydroxyoleic acid

CYP51A1	0,00	cytochrome P450 family 51 subfamily A member 1	Cytoplasm	enzyme	voriconazole, efinaconazole, posaconazole, ketoconazole, terconazole, sertaconazole, bifonazole, oxiconazole, fluconazole, itraconazole, clotrimazole, miconazole, econazole, betamethasone/clotrimazole, luliconazole
GNAI3	0,00	G protein subunit alpha i3	Cytoplasm	enzyme	
AMPD2	0,00	adenosine monophosphate deaminase 2	Cytoplasm	enzyme	
AAK1	0,00	AP2 associated kinase 1	Cytoplasm	kinase	LP-935509, SM1-71
PARVA	0,00	parvin alpha	Cytoplasm	other	
TRADD	0,00	TNFRSF1A associated via death domain	Cytoplasm	other	
LIMA1	0,00	LIM domain and actin binding 1	Cytoplasm	other	
MVB12A	0,00	multivesicular body subunit 12A	Cytoplasm	other	
COMMD9	0,00	COMM domain containing 9	Cytoplasm	other	
IQGAP2	0,00	IQ motif containing GTPase activating protein 2	Cytoplasm	other	
AKAP13	0,00	A-kinase anchoring protein 13	Cytoplasm	other	
UFM1	0,00	ubiquitin fold modifier 1	Cytoplasm	other	
GET4	0,00	guided entry of tail-anchored proteins factor 4	Cytoplasm	other	
PRKRA	0,00	protein activator of interferon induced protein kinase EIF2AK2	Cytoplasm	other	
RCN2	0,00	reticulocalbin 2	Cytoplasm	other	
CAPN2	0,00	calpain 2	Cytoplasm	peptidase	alicapistat, BLD-2660
EPHX1	0,00	epoxide hydrolase 1	Cytoplasm	peptidase	
PTPN23	0,00	protein tyrosine phosphatase non-receptor type 23	Cytoplasm	phosphatase	
PHPT1	0,00	phosphohistidine phosphatase 1	Cytoplasm	phosphatase	
IMPA1	0,00	inositol monophosphatase 1	Cytoplasm	phosphatase	
MTX1	0,00	metaxin 1	Cytoplasm	transporter	
EPHB4	0,00	EPH receptor B4	Plasma Membrane	kinase	tesevatinib, JI 101, AZ12672857, NVP-BHG712
CAP1	0,00	cyclase associated actin cytoskeleton regulatory protein 1	Plasma Membrane	other	
LAMC1	0,00	laminin subunit gamma 1	Extracellular Space	other	
NDRG1	0,00	N-myc downstream regulated 1	Nucleus	kinase	
SLTM	0,00	SAFB like transcription modulator	Nucleus	other	
CNOT7	0,00	CCR4-NOT transcription complex subunit 7	Nucleus	transcription regulator	
SMARCD2	0,00	SWI/SNF related, matrix associated, actin dependent regulator of chromatin, subfamily d, member 2	Nucleus	transcription regulator	
VPS25	0,00	vacuolar protein sorting 25 homolog	Cytoplasm	other	
TMOD3	0,00	tropomodulin 3	Cytoplasm	other	
TBC1D15	0,00	TBC1 domain family member 15	Cytoplasm	other	
DYNLT1	0,00	dynein light chain Tctex-type 1	Cytoplasm	other	
MRPL28	0,00	mitochondrial ribosomal protein L28	Cytoplasm	other	
RTN3	0,00	reticulon 3	Cytoplasm	other	
TMSB10/TMSB4X	0,00	thymosin beta 4 X-linked	Cytoplasm	other	
S100A11	0,00	S100 calcium binding protein A11	Cytoplasm	other	
NT5C	0,00	5', 3'-nucleotidase, cytosolic	Cytoplasm	phosphatase	
PEX14	0,00	peroxisomal biogenesis factor 14	Cytoplasm	transcription regulator	
ANKFY1	0,00	ankyrin repeat and FYVE domain containing 1	Cytoplasm	transcription regulator	
ARL8B	0,00	ADP ribosylation factor like GTPase 8B	Plasma Membrane	enzyme	
NCEH1	0,00	neutral cholesterol ester hydrolase 1	Plasma Membrane	enzyme	
ALCAM	0,00	activated leukocyte cell adhesion molecule	Plasma Membrane	other	praluzatamab ravtansine
TOR1AIP1	0,00	torsin 1A interacting protein 1	Nucleus	other	
INTS3	0,00	integrator complex subunit 3	Nucleus	other	

RAVER1	0,00	ribonucleoprotein, PTB binding 1	Nucleus	other	
PGAM5	0,00	PGAM family member 5, mitochondrial serine/threonine protein phosphatase	Cytoplasm	enzyme	
FDPS	0,00	farnesyl diphosphate synthase	Cytoplasm	enzyme	alendronate/cholecalciferol, zoledronic acid, minodronate, ibandronic acid, risedronic acid, alendronic acid, pamidronic acid, docetaxel/zoledronic acid
GALNT1	0,00	polypeptide N-acetylgalactosaminyltransferase 1	Cytoplasm	enzyme	
AGL	0,00	amylo-alpha-1, 6-glucosidase, 4-alpha-glucanotransferase	Cytoplasm	enzyme	
TGM2	0,00	transglutaminase 2	Cytoplasm	enzyme	
GNS	0,00	glucosamine (N-acetyl)-6-sulfatase	Cytoplasm	enzyme	
VPS37B	0,00	VPS37B subunit of ESCRT-I	Cytoplasm	other	
PACSIN2	0,00	protein kinase C and casein kinase substrate in neurons 2	Cytoplasm	other	
IST1	0,00	IST1 factor associated with ESCRT-III	Cytoplasm	other	
NECAP2	0,00	NECAP endocytosis associated 2	Cytoplasm	other	
MICU2	0,00	mitochondrial calcium uptake 2	Cytoplasm	other	
RIC8A	0,00	RIC8 guanine nucleotide exchange factor A	Cytoplasm	other	
MPRIP	0,00	myosin phosphatase Rho interacting protein	Cytoplasm	other	
ABCC1	0,00	ATP binding cassette subfamily C member 1	Plasma Membrane	transporter	sulfinpyrazone
PPIL4	0,00	peptidylprolyl isomerase like 4	Nucleus	enzyme	
TRIOBP	0,00	TRIO and F-actin binding protein	Nucleus	other	
ANP32E	0,00	acidic nuclear phosphoprotein 32 family member E	Nucleus	other	
HMGB2	0,00	high mobility group box 2	Nucleus	transcription regulator	
GATAD2B	0,00	GATA zinc finger domain containing 2B	Nucleus	transcription regulator	
PCID2	0,00	PCI domain containing 2	Nucleus	transcription regulator	
GTF2I	0,00	general transcription factor IIi	Nucleus	transcription regulator	
PGRMC2	0,00	progesterone receptor membrane component 2	Nucleus	transporter	
AKR7A2	0,00	aldo-keto reductase family 7 member A2	Cytoplasm	enzyme	
FKBP9	0,00	FKBP prolyl isomerase 9	Cytoplasm	enzyme	
QDPR	0,00	quinoid dihydropteridine reductase	Cytoplasm	enzyme	
STK24	0,00	serine/threonine kinase 24	Cytoplasm	kinase	bosutinib, bosutinib/rituximab, bosutinib/imatinib
MYO1B	0,00	myosin IB	Cytoplasm	other	
ARPC5L	0,00	actin related protein 2/3 complex subunit 5 like	Cytoplasm	other	
TBRG4	0,00	transforming growth factor beta regulator 4	Cytoplasm	other	
KIF13B	0,00	kinesin family member 13B	Cytoplasm	other	
TRAM1	0,00	translocation associated membrane protein 1	Cytoplasm	other	
NT5C2	0,00	5'-nucleotidase, cytosolic II	Cytoplasm	phosphatase	
CNOT9	0,00	CCR4-NOT transcription complex subunit 9	Cytoplasm	transcription regulator	
SRI	0,00	sorcin	Cytoplasm	transporter	
VPS33A	0,00	VPS33A core subunit of CORVET and HOPS complexes	Cytoplasm	transporter	
CTNNA2	0,00	catenin alpha 2	Plasma Membrane	other	
UBXN4	0,00	UBX domain protein 4	Extracellular Space	other	
TMEM43	0,00	transmembrane protein 43	Nucleus	other	
RCC2	0,00	regulator of chromosome condensation 2	Nucleus	other	
CCS	0,00	copper chaperone for superoxide dismutase	Cytoplasm	enzyme	
PDIA5	0,00	protein disulfide isomerase family A member 5	Cytoplasm	enzyme	
MAP1S	0,00	microtubule associated protein 1S	Cytoplasm	enzyme	

RAB8B	0,00	RAB8B, member RAS oncogene family	Cytoplasm	enzyme	
MRPL37	0,00	mitochondrial ribosomal protein L37	Cytoplasm	enzyme	
RAB21	0,00	RAB21, member RAS oncogene family	Cytoplasm	enzyme	
ACOT2	0,00	acyl-CoA thioesterase 2	Cytoplasm	enzyme	
CERT1	0,00	ceramide transporter 1	Cytoplasm	kinase	
PDXK	0,00	pyridoxal kinase	Cytoplasm	kinase	
DYNLRB1	0,00	dynein light chain roadblock-type 1	Cytoplasm	other	
MSI2	0,00	musashi RNA binding protein 2	Cytoplasm	other	
NAP1L4	0,00	nucleosome assembly protein 1 like 4	Cytoplasm	other	
FAM120A	0,00	family with sequence similarity 120 member A	Cytoplasm	other	
GOLGA3	0,00	golgin A3	Cytoplasm	transporter	
FABP5	0,00	fatty acid binding protein 5	Cytoplasm	transporter	
LASP1	0,00	LIM and SH3 protein 1	Cytoplasm	transporter	
LPCAT3	0,00	lysophosphatidylcholine acyltransferase 3	Plasma Membrane	enzyme	
ADPGK	0,00	ADP dependent glucokinase	Plasma Membrane	kinase	
LRPAP1	0,00	LDL receptor related protein associated protein 1	Plasma Membrane	other	
CHORDC1	0,00	cysteine and histidine rich domain containing 1	Other	other	
EMC3	0,00	ER membrane protein complex subunit 3	Nucleus	other	
H1-5	0,00	H1.5 linker histone, cluster member	Nucleus	other	
CYB5R3	0,00	cytochrome b5 reductase 3	Cytoplasm	enzyme	
UROD	0,00	uroporphyrinogen decarboxylase	Cytoplasm	enzyme	
TMX1	0,00	thioredoxin related transmembrane protein 1	Cytoplasm	enzyme	
MAN1A2	0,00	mannosidase alpha class 1A member 2	Cytoplasm	enzyme	
ASAH1	0,00	N-acylsphingosine amidohydrolase 1	Cytoplasm	enzyme	
PRKAR2A	0,00	protein kinase cAMP-dependent type II regulatory subunit alpha	Cytoplasm	kinase	
WASHC4	0,00	WASH complex subunit 4	Cytoplasm	other	
SAFB2	0,00	scaffold attachment factor B2	Cytoplasm	other	
ATL3	0,00	atlastin GTPase 3	Cytoplasm	other	
CCDC6	0,00	coiled-coil domain containing 6	Cytoplasm	other	
BID	0,00	BH3 interacting domain death agonist	Cytoplasm	other	
VPS37C	0,00	VPS37C subunit of ESCRT-I	Cytoplasm	other	
LGMN	0,00	legumain	Cytoplasm	peptidase	
CNDP2	0,00	carnosine dipeptidase 2	Cytoplasm	peptidase	
PREP	0,00	prolyl endopeptidase	Cytoplasm	peptidase	Z 321
PTPA	0,00	protein phosphatase 2 phosphatase activator	Cytoplasm	phosphatase	
DOCK7	0,00	dedicator of cytokinesis 7	Plasma Membrane	other	
MLEC	0,00	malectin	Plasma Membrane	other	
LGALS3BP	0,00	galectin 3 binding protein	Plasma Membrane	transmembrane receptor	
SLC7A5	0,00	solute carrier family 7 member 5	Plasma Membrane	transporter	QBS10072S
MPC2	0,00	mitochondrial pyruvate carrier 2	Plasma Membrane	transporter	
LAMA5	0,00	laminin subunit alpha 5	Extracellular Space	other	
REEP5	0,00	receptor accessory protein 5	Extracellular Space	transporter	
NPC2	0,00	NPC intracellular cholesterol transporter 2	Extracellular Space	transporter	
YLPM1	0,00	YLP motif containing 1	Nucleus	transcription regulator	
MMS19	0,00	MMS19 homolog, cytosolic iron-sulfur assembly component	Nucleus	transcription regulator	
ZC3H4	0,00	zinc finger CCCH-type containing 4	Nucleus	transcription regulator	
GTPBP1	0,00	GTP binding protein 1	Cytoplasm	enzyme	
PTRH2	0,00	peptidyl-tRNA hydrolase 2	Cytoplasm	enzyme	
TAOK3	0,00	TAO kinase 3	Cytoplasm	kinase	TAO3 inhibitor

ACBD3	0,00	acyl-CoA binding domain containing 3	Cytoplasm	other	
SPAG9	0,00	sperm associated antigen 9	Cytoplasm	other	
UACA	0,00	uveal autoantigen with coiled-coil domains and ankyrin repeats	Cytoplasm	other	
GGH	0,00	gamma-glutamyl hydrolase	Cytoplasm	peptidase	
APEH	0,00	acylaminoacyl-peptide hydrolase	Cytoplasm	peptidase	
FKBP15	0,00	FKBP prolyl isomerase family member 15	Plasma Membrane	enzyme	
MARCKS	0,00	myristoylated alanine rich protein kinase C substrate	Plasma Membrane	other	BIO-11006
ATP1B3	0,00	ATPase Na+/K+ transporting subunit beta 3	Plasma Membrane	transporter	
NAXE	0,00	NAD(P)HX epimerase	Extracellular Space	enzyme	
LAMB1	0,00	laminin subunit beta 1	Extracellular Space	other	
TRMT1L	0,00	tRNA methyltransferase 1 like	Other	enzyme	
FAM114A2	0,00	family with sequence similarity 114 member A2	Other	other	
SLK	0,00	STE20 like kinase	Nucleus	kinase	
DCAF8	0,00	DDB1 and CUL4 associated factor 8	Nucleus	other	
RO60	0,00	Ro60, Y RNA binding protein	Nucleus	other	
BABAM1	0,00	BRISC and BRCA1 A complex member 1	Nucleus	other	
MYO1E	0,00	myosin IE	Cytoplasm	enzyme	
ACOT9	0,00	acyl-CoA thioesterase 9	Cytoplasm	enzyme	
UBE2H	0,00	ubiquitin conjugating enzyme E2 H	Cytoplasm	enzyme	
PPP1R9B	0,00	protein phosphatase 1 regulatory subunit 9B	Cytoplasm	enzyme	
ABHD11	0,00	abhydrolase domain containing 11	Cytoplasm	enzyme	
NANS	0,00	N-acetylneuraminate synthase	Cytoplasm	enzyme	
SCYL1	0,00	SCY1 like pseudokinase 1	Cytoplasm	kinase	
WNK1	0,00	WNK lysine deficient protein kinase 1	Cytoplasm	kinase	WNK463
S100A13	0,00	S100 calcium binding protein A13	Cytoplasm	other	
VAC14	0,00	VAC14 component of PIKFYVE complex	Cytoplasm	other	
MAPRE2	0,00	microtubule associated protein RP/EB family member 2	Cytoplasm	other	
CTSH	0,00	cathepsin H	Cytoplasm	peptidase	
MTDH	0,00	metadherin	Cytoplasm	transcription regulator	
SFXN1	0,00	sideroflexin 1	Cytoplasm	transporter	
ERAP1	0,00	endoplasmic reticulum aminopeptidase 1	Extracellular Space	peptidase	
AKAP8	0,00	A-kinase anchoring protein 8	Nucleus	other	
ANP32B	0,00	acidic nuclear phosphoprotein 32 family member B	Nucleus	other	
GSS	0,00	glutathione synthetase	Cytoplasm	enzyme	N-acetyl-L-cysteine
RNF213	0,00	ring finger protein 213	Cytoplasm	enzyme	
MGST1	0,00	microsomal glutathione S-transferase 1	Cytoplasm	enzyme	
UFL1	0,00	UFM1 specific ligase 1	Cytoplasm	enzyme	
THOP1	0,00	thimet oligopeptidase 1	Cytoplasm	peptidase	
LRRFIP1	0,00	LRR binding FLII interacting protein 1	Cytoplasm	transcription regulator	
PDLIM1	0,00	PDZ and LIM domain 1	Cytoplasm	transcription regulator	
TMED4	0,00	transmembrane p24 trafficking protein 4	Cytoplasm	transporter	
TECR	0,00	trans-2,3-enoyl-CoA reductase	Plasma Membrane	enzyme	
FARP1	0,00	FERM, ARH/RhoGEF and pleckstrin domain protein 1	Plasma Membrane	other	
CGN	0,00	cingulin	Plasma Membrane	other	
FERMT1	0,00	FERM domain containing kindlin 1	Plasma Membrane	other	
PGRMC1	0,00	progesterone receptor membrane component 1	Plasma Membrane	transmembrane receptor	CT1812
ATP1B1	0,00	ATPase Na+/K+ transporting subunit beta 1	Plasma Membrane	transporter	

ATP2B1	0,00	ATPase plasma membrane Ca2+ transporting 1	Plasma Membrane	transporter	
MYH14	0,00	myosin heavy chain 14	Extracellular Space	enzyme	mavacamten
OTUD6B	0,00	OTU deubiquitinase 6B	Other	peptidase	
HP1BP3	0,00	heterochromatin protein 1 binding protein 3	Nucleus	other	
FLII	0,00	FLII actin remodeling protein	Nucleus	other	
ZMYND8	0,00	zinc finger MYND-type containing 8	Nucleus	transcription regulator	
SH3GLB1	0,00	SH3 domain containing GRB2 like, endophilin B1	Cytoplasm	enzyme	
CYB5R1	0,00	cytochrome b5 reductase 1	Cytoplasm	enzyme	
HMOX2	0,00	heme oxygenase 2	Cytoplasm	enzyme	tin mesoporphyrin
GSTZ1	0,00	glutathione S-transferase zeta 1	Cytoplasm	enzyme	
NIPSNAP1	0,00	nipsnap homolog 1	Cytoplasm	enzyme	
GAN	0,00	gigaxonin	Cytoplasm	other	
COTL1	0,00	coactosin like F-actin binding protein 1	Cytoplasm	other	
VPS13C	0,00	vacuolar protein sorting 13 homolog C	Cytoplasm	other	
EPS8L2	0,00	EPS8 like 2	Cytoplasm	other	
PPM1A	0,00	protein phosphatase, Mg2+/Mn2+ dependent 1A	Cytoplasm	phosphatase	
BZW1	0,00	basic leucine zipper and W2 domains 1	Cytoplasm	translation regulator	
SCAMP1	0,00	secretory carrier membrane protein 1	Cytoplasm	transporter	
SLC16A1	0,00	solute carrier family 16 member 1	Plasma Membrane	transporter	AZD-3965
TMEM214	0,00	transmembrane protein 214	Extracellular Space	other	
LPP	0,00	LIM domain containing preferred translocation partner in lipoma	Nucleus	other	
CAPG	0,00	capping actin protein, gelsolin like	Nucleus	other	
RPRD1B	0,00	regulation of nuclear pre-mRNA domain containing 1B	Nucleus	other	
CSRP1	0,00	cysteine and glycine rich protein 1	Nucleus	other	
MYOF	0,00	myoferlin	Nucleus	other	
USP24	0,00	ubiquitin specific peptidase 24	Nucleus	peptidase	
AIP	0,00	aryl hydrocarbon receptor interacting protein	Nucleus	transcription regulator	
SPTLC2	0,00	serine palmitoyltransferase long chain base subunit 2	Cytoplasm	enzyme	
BLVRA	0,00	biliverdin reductase A	Cytoplasm	enzyme	
AGPAT1	0,00	1-acylglycerol-3-phosphate O-acyltransferase 1	Cytoplasm	enzyme	
PCYT1A	0,00	phosphate cytidylyltransferase 1A, choline	Cytoplasm	enzyme	
TOR1B	0,00	torsin family 1 member B	Cytoplasm	enzyme	
PLPBP	0,00	pyridoxal phosphate binding protein	Cytoplasm	enzyme	
SELENBP1	0,00	selenium binding protein 1	Cytoplasm	enzyme	
SPR	0,00	sepiapterin reductase	Cytoplasm	enzyme	
CARMIL1	0,00	capping protein regulator and myosin 1 linker 1	Cytoplasm	enzyme	
MAP4	0,00	microtubule associated protein 4	Cytoplasm	other	
PDLIM5	0,00	PDZ and LIM domain 5	Cytoplasm	other	
HOOK3	0,00	hook microtubule tethering protein 3	Cytoplasm	other	
TBCD	0,00	tubulin folding cofactor D	Cytoplasm	other	
ZFPL1	0,00	zinc finger protein like 1	Cytoplasm	other	
CSTB	0,00	cystatin B	Cytoplasm	peptidase	
DPP7	0,00	dipeptidyl peptidase 7	Cytoplasm	peptidase	
DPP3	0,00	dipeptidyl peptidase 3	Cytoplasm	peptidase	
NUDT5	0,00	nudix hydrolase 5	Cytoplasm	phosphatase	
PTGES2	0,00	prostaglandin E synthase 2	Cytoplasm	transcription regulator	
MRI1	0,00	methylthioribose-1-phosphate isomerase 1	Cytoplasm	translation regulator	
ATP2C1	0,00	ATPase secretory pathway Ca2+ transporting 1	Cytoplasm	transporter	sevoflurane, isoflurane, desflurane, enflurane, Ca2+
TMEM14C	0,00	transmembrane protein 14C	Plasma Membrane	other	

TMEM165	0,00	transmembrane protein 165	Plasma Membrane	other	
OSBPL8	0,00	oxysterol binding protein like 8	Plasma Membrane	transporter	
SEPHS1	0,00	selenophosphate synthetase 1	Other	enzyme	
CLIC1	0,00	chloride intracellular channel 1	Nucleus	ion channel	
CERS2	0,00	ceramide synthase 2	Nucleus	transcription regulator	
PCYT2	0,00	phosphate cytidylyltransferase 2, ethanolamine	Cytoplasm	enzyme	
HEXB	0,00	hexosaminidase subunit beta	Cytoplasm	enzyme	
NAGA	0,00	alpha-N-acetylgalactosaminidase	Cytoplasm	enzyme	
GM2A	0,00	ganglioside GM2 activator	Cytoplasm	enzyme	
PLOD1	0,00	procollagen-lysine,2-oxoglutarate 5-dioxygenase 1	Cytoplasm	enzyme	
TUBGCP3	0,00	tubulin gamma complex component 3	Cytoplasm	other	
XPNPEP1	0,00	X-prolyl aminopeptidase 1	Cytoplasm	peptidase	
TUBGCP2	0,00	tubulin gamma complex component 2	Cytoplasm	peptidase	
NPEPPS	0,00	aminopeptidase puromycin sensitive	Cytoplasm	peptidase	
SLMAP	0,00	sarcolemma associated protein	Plasma Membrane	other	
NUDT2	0,00	nudix hydrolase 2	Plasma Membrane	phosphatase	
NID1	0,00	nidogen 1	Extracellular Space	other	
ATP13A1	0,00	ATPase 13A1	Extracellular Space	transporter	
PRRC1	0,00	proline rich coiled-coil 1	Other	other	
PPP4R1	0,00	protein phosphatase 4 regulatory subunit 1	Other	phosphatase	
MTPN	0,00	myotrophin	Nucleus	transcription regulator	
FKBP11	0,00	FKBP prolyl isomerase 11	Cytoplasm	enzyme	
BCL2L13	0,00	BCL2 like 13	Cytoplasm	other	
PPL	0,00	periplakin	Cytoplasm	other	
IAH1	0,00	isoamyl acetate hydrolyzing esterase 1 (putative)	Cytoplasm	other	
NUCB1	0,00	nucleobindin 1	Cytoplasm	other	
DENND4C	0,00	DENN domain containing 4C	Cytoplasm	other	
FMNL2	0,00	formin like 2	Cytoplasm	other	
ARHGAP17	0,00	Rho GTPase activating protein 17	Cytoplasm	other	
TPP2	0,00	tripeptidyl peptidase 2	Cytoplasm	peptidase	
PITPNA	0,00	phosphatidylinositol transfer protein alpha	Cytoplasm	transporter	
TES	0,00	testin LIM domain protein	Plasma Membrane	other	
TNS3	0,00	tensin 3	Plasma Membrane	phosphatase	
SLC12A2	0,00	solute carrier family 12 member 2	Plasma Membrane	transporter	bumetanide, quinethazone
ADI1	0,00	acireductone dioxygenase 1	Nucleus	enzyme	
H1-3	0,00	H1.3 linker histone, cluster member	Nucleus	other	
MVP	0,00	major vault protein	Nucleus	other	
PPP1R7	0,00	protein phosphatase 1 regulatory subunit 7	Nucleus	phosphatase	
TMX3	0,00	thioredoxin related transmembrane protein 3	Cytoplasm	enzyme	
HACD3	0,00	3-hydroxyacyl-CoA dehydratase 3	Cytoplasm	enzyme	
GAA	0,00	alpha glucosidase	Cytoplasm	enzyme	miglitol, acarbose, acarbose/orlistat
TXNDC17	0,00	thioredoxin domain containing 17	Cytoplasm	enzyme	
ASPH	0,00	aspartate beta-hydroxylase	Cytoplasm	enzyme	
PIP4K2C	0,00	phosphatidylinositol-5-phosphate 4-kinase type 2 gamma	Cytoplasm	kinase	SM1-71
NAGK	0,00	N-acetylglucosamine kinase	Cytoplasm	kinase	
ATXN10	0,00	ataxin 10	Cytoplasm	other	
LIN7C	0,00	lin-7 homolog C, crumbs cell polarity complex component	Cytoplasm	other	
PIGK	0,00	phosphatidylinositol glycan anchor biosynthesis class K	Cytoplasm	peptidase	
CTSE	0,00	cathepsin E	Cytoplasm	peptidase	

XPNPEP3	0,00	X-prolyl aminopeptidase 3	Cytoplasm	peptidase
SCAMP3	0,00	secretory carrier membrane protein 3	Cytoplasm	transporter
ANXA4	0,00	annexin A4	Plasma Membrane	other
MYDGF	0,00	myeloid derived growth factor	Extracellular Space	cytokine
ENDOD1	0,00	endonuclease domain containing 1	Extracellular Space	enzyme
PRUNE1	0,00	prune exopolyphosphatase 1	Nucleus	enzyme
OXSR1	0,00	oxidative stress responsive kinase 1	Nucleus	kinase
PAFAH1B2	0,00	platelet activating factor acetylhydrolase 1b catalytic subunit 2	Cytoplasm	enzyme
LYPLA1	0,00	lysophospholipase 1	Cytoplasm	enzyme
NAGLU	0,00	N-acetyl-alpha-glucosaminidase	Cytoplasm	enzyme
PCYOX1	0,00	prenylcysteine oxidase 1	Cytoplasm	enzyme
B4GALT1	0,00	beta-1,4-galactosyltransferase 1	Cytoplasm	enzyme
SELENOI	0,00	selenoprotein I	Cytoplasm	enzyme
HSD17B11	0,00	hydroxysteroid 17-beta dehydrogenase 11	Cytoplasm	enzyme
ANXA3	0,00	annexin A3	Cytoplasm	enzyme
CLCC1	0,00	chloride channel CLIC like 1	Cytoplasm	ion channel
TIPRL	0,00	TOR signaling pathway regulator	Cytoplasm	other
LNPK	0,00	lunapark, ER junction formation factor	Cytoplasm	other
ARFIP1	0,00	ADP ribosylation factor interacting protein 1	Cytoplasm	other
SCAMP2	0,00	secretory carrier membrane protein 2	Cytoplasm	other
AHNAK2	0,00	AHNAK nucleoprotein 2	Cytoplasm	other
ERGIC1	0,00	endoplasmic reticulum-golgi intermediate compartment 1	Cytoplasm	other
GLG1	0,00	golgi glycoprotein 1	Cytoplasm	other
BROX	0,00	BRO1 domain and CAAX motif containing	Cytoplasm	other
OCIAD1	0,00	OCIA domain containing 1	Cytoplasm	other
CPNE1	0,00	copine 1	Cytoplasm	peptidase
ESYT2	0,00	extended synaptotagmin 2	Plasma Membrane	other
CNIH4	0,00	cornichon family AMPA receptor auxiliary protein 4	Plasma Membrane	other
PTGFRN	0,00	prostaglandin F2 receptor inhibitor	Plasma Membrane	other
PPFIA1	0,00	PTPRF interacting protein alpha 1	Plasma Membrane	phosphatase
SCRN2	0,00	secernin 2	Extracellular Space	other
CMAS	0,00	cytidine monophosphate N-acetylneuraminic acid synthetase	Nucleus	enzyme
PDS5B	0,00	PDS5 cohesin associated factor B	Nucleus	other
CDK5RAP3	0,00	CDK5 regulatory subunit associated protein 3	Nucleus	other
NHLRC2	0,00	NHL repeat containing 2	Cytoplasm	enzyme
LTA4H	0,00	leukotriene A4 hydrolase	Cytoplasm	enzyme
NAXD	0,00	NAD(P)HX dehydratase	Cytoplasm	enzyme
TKFC	0,00	triokinase and FMN cyclase	Cytoplasm	kinase
ARHGEF16	0,00	Rho guanine nucleotide exchange factor 16	Cytoplasm	other
ARL6IP5	0,00	ADP ribosylation factor like GTPase 6 interacting protein 5	Cytoplasm	other
TBCB	0,00	tubulin folding cofactor B	Cytoplasm	other
RNPEP	0,00	arginyl aminopeptidase	Cytoplasm	peptidase
TAX1BP3	0,00	Tax1 binding protein 3	Cytoplasm	transcription regulator
PITPNB	0,00	phosphatidylinositol transfer protein beta	Cytoplasm	transporter
KDSR	0,00	3-ketodihydrosphingosine reductase	Plasma Membrane	enzyme
TM9SF2	0,00	transmembrane 9 superfamily member 2	Plasma Membrane	transporter
PRXL2A	0,00	peroxiredoxin like 2A	Extracellular Space	other

SETD3	0,00	SET domain containing 3, actin N3(tau)-histidine methyltransferase	Nucleus	enzyme	
FAM98B	0,00	family with sequence similarity 98 member B	Nucleus	enzyme	
CENPV	0,00	centromere protein V	Nucleus	other	
RBM47	0,00	RNA binding motif protein 47	Nucleus	other	
ZC3HC1	0,00	zinc finger C3HC-type containing 1	Nucleus	other	
SH3BGRL3	0,00	SH3 domain binding glutamate rich protein like 3	Nucleus	other	
MYO1D	0,00	myosin ID	Cytoplasm	enzyme	
ABHD14B	0,00	abhydrolase domain containing 14B	Cytoplasm	enzyme	
VWA8	0,00	von Willebrand factor A domain containing 8	Cytoplasm	enzyme	
STK3	0,00	serine/threonine kinase 3	Cytoplasm	kinase	XMU-MP-1
TBCE	0,00	tubulin folding cofactor E	Cytoplasm	other	
ARHGEF40	0,00	Rho guanine nucleotide exchange factor 40	Cytoplasm	other	
CUTA	0,00	cutA divalent cation tolerance homolog	Cytoplasm	other	
NLN	0,00	neurolysin	Cytoplasm	peptidase	
CARHSP1	0,00	calcium regulated heat stable protein 1	Cytoplasm	transcription regulator	
GRIPAP1	0,00	GRIP1 associated protein 1	Plasma Membrane	other	
TBL2	0,00	transducin beta like 2	Plasma Membrane	other	
OSBPL3	0,00	oxysterol binding protein like 3	Plasma Membrane	transporter	
NPM3	0,00	nucleophosmin/nucleoplasmin 3	Nucleus	other	
GID8	0,00	GID complex subunit 8 homolog	Nucleus	other	
STRIP1	0,00	striatin interacting protein 1	Nucleus	other	
PCBD1	0,00	pterin-4 alpha-carbinolamine dehydratase 1	Nucleus	transcription regulator	
PURB	0,00	purine rich element binding protein B	Nucleus	transcription regulator	
DR1	0,00	down-regulator of transcription 1	Nucleus	transcription regulator	
GYG1	0,00	glycogenin 1	Cytoplasm	enzyme	
EBP	0,00	EBP cholestenol delta-isomerase	Cytoplasm	enzyme	DSP-0390, SR 31747
HINT2	0,00	histidine triad nucleotide binding protein 2	Cytoplasm	enzyme	
RDH11	0,00	retinol dehydrogenase 11	Cytoplasm	enzyme	
PTGR1	0,00	prostaglandin reductase 1	Cytoplasm	enzyme	
HDHD3	0,00	haloacid dehalogenase like hydrolase domain containing 3	Cytoplasm	enzyme	
PIGU	0,00	phosphatidylinositol glycan anchor biosynthesis class U	Cytoplasm	enzyme	
SUMF2	0,00	sulfatase modifying factor 2	Cytoplasm	other	
RNH1	0,00	ribonuclease/angiogenin inhibitor 1	Cytoplasm	other	
LRBA	0,00	LPS responsive beige-like anchor protein	Cytoplasm	other	
TOR1AIP2	0,00	torsin 1A interacting protein 2	Cytoplasm	other	
TMED5	0,00	transmembrane p24 trafficking protein 5	Cytoplasm	other	
CLIC4	0,00	chloride intracellular channel 4	Plasma Membrane	ion channel	
ITM2B	0,00	integral membrane protein 2B	Plasma Membrane	other	
CNPY2	0,00	canopy FGF signaling regulator 2	Plasma Membrane	other	
KTI12	0,00	KTI12 chromatin associated homolog	Other	other	
ZNRD2	0,00	zinc ribbon domain containing 2	Other	other	
PBDC1	0,00	polysaccharide biosynthesis domain containing 1	Other	other	
UFSP2	0,00	UFM1 specific peptidase 2	Other	peptidase	
ASAP2	0,00	ArfGAP with SH3 domain, ankyrin repeat and PH domain 2	Nucleus	other	
BPNT1	0,00	3'(2'), 5'-bisphosphate nucleotidase 1	Nucleus	phosphatase	
OSTF1	0,00	osteoclast stimulating factor 1	Nucleus	transcription regulator	

BPHL	0,00	biphenyl hydrolase like	Cytoplasm	enzyme
QTRT2	0,00	queuine tRNA-ribosyltransferase accessory subunit 2	Cytoplasm	enzyme
CYP2S1	0,00	cytochrome P450 family 2 subfamily S member 1	Cytoplasm	enzyme
PLOD3	0,00	procollagen-lysine,2-oxoglutarate 5-dioxygenase 3	Cytoplasm	enzyme
PIGS	0,00	phosphatidylinositol glycan anchor biosynthesis class S	Cytoplasm	enzyme
RDH13	0,00	retinol dehydrogenase 13	Cytoplasm	enzyme
PAFAH1B3	0,00	platelet activating factor acetylhydrolase 1b catalytic subunit 3	Cytoplasm	enzyme
GMFB	0,00	glia maturation factor beta	Cytoplasm	growth factor
ABR	0,00	ABR activator of RhoGEF and GTPase	Cytoplasm	other
APPL2	0,00	adaptor protein, phosphotyrosine interacting with PH domain and leucine zipper 2	Cytoplasm	other
CNN3	0,00	calponin 3	Cytoplasm	other
EIPR1	0,00	EARP complex and GARP complex interacting protein 1	Cytoplasm	other
EPPK1	0,00	epiplakin 1	Cytoplasm	other
RMDN1	0,00	regulator of microtubule dynamics 1	Cytoplasm	other
SCYL2	0,00	SCY1 like pseudokinase 2	Cytoplasm	other
SH2D4A	0,00	SH2 domain containing 4A	Cytoplasm	other
RBPMS	0,00	RNA binding protein, mRNA processing factor	Cytoplasm	transcription regulator
PEA15	0,00	proliferation and apoptosis adaptor protein 15	Cytoplasm	transporter
TOM1L2	0,00	target of myb1 like 2 membrane trafficking protein	Cytoplasm	transporter
ABHD12	0,00	abhydrolase domain containing 12, lysophospholipase	Plasma Membrane	enzyme
PLXNB2	0,00	plexin B2	Plasma Membrane	transmembrane receptor
VAT1	0,00	vesicle amine transport 1	Plasma Membrane	transporter
TRABD	0,00	TraB domain containing	Extracellular Space	other
ECM1	0,00	extracellular matrix protein 1	Extracellular Space	transporter
NRBP1	0,00	nuclear receptor binding protein 1	Nucleus	kinase
PCNP	0,00	PEST proteolytic signal containing nuclear protein	Nucleus	other
THUMPD1	0,00	THUMP domain containing 1	Nucleus	other
ELF1	0,00	E74 like ETS transcription factor 1	Nucleus	transcription regulator
TRIM56	0,00	tripartite motif containing 56	Cytoplasm	enzyme
COLGALT1	0,00	collagen beta(1-O)galactosyltransferase 1	Cytoplasm	enzyme
CDS2	0,00	CDP-diacylglycerol synthase 2	Cytoplasm	enzyme
HDDC2	0,00	HD domain containing 2	Cytoplasm	other
HDHD5	0,00	haloacid dehalogenase like hydrolase domain containing 5	Cytoplasm	other
HDHD2	0,00	haloacid dehalogenase like hydrolase domain containing 2	Cytoplasm	other
GOLM1	0,00	golgi membrane protein 1	Cytoplasm	other
ARMCX3	0,00	armadillo repeat containing X-linked 3	Cytoplasm	other
TTLL12	0,00	tubulin tyrosine ligase like 12	Cytoplasm	other
NBEAL2	0,00	neurobeachin like 2	Cytoplasm	other
BZW2	0,00	basic leucine zipper and W2 domains 2	Cytoplasm	translation regulator
OSBPL9	0,00	oxysterol binding protein like 9	Cytoplasm	transporter
PLP2	0,00	proteolipid protein 2	Cytoplasm	transporter
EHD4	0,00	EH domain containing 4	Plasma Membrane	enzyme
SLC39A7	0,00	solute carrier family 39 member 7	Plasma Membrane	transporter

CYRIB	0,00	CYFIP related Rac1 interactor B	Extracellular Space	other
MYADM	0,00	myeloid associated differentiation marker	Nucleus	other
PTMS	0,00	parathymosin	Nucleus	other
NUCB2	0,00	nucleobindin 2	Nucleus	other
NAAA	0,00	N-acylethanolamine acid amidase	Cytoplasm	enzyme
CPNE3	0,00	copine 3	Cytoplasm	kinase
SH3BGRL	0,00	SH3 domain binding glutamate rich protein like	Cytoplasm	other
HPCAL1	0,00	hippocalcin like 1	Cytoplasm	other
SCCPDH	0,00	saccharopine dehydrogenase (putative)	Cytoplasm	other
TPD52L2	0,00	TPD52 like 2	Cytoplasm	other
NDRG3	0,00	NDRG family member 3	Cytoplasm	other
RUFY1	0,00	RUN and FYVE domain containing 1	Cytoplasm	other
PDXDC1	0,00	pyridoxal dependent decarboxylase domain containing 1	Cytoplasm	other
MEMO1	0,00	mediator of cell motility 1	Cytoplasm	other
PLGRKT	0,00	plasminogen receptor with a C-terminal lysine	Cytoplasm	other
SCPEP1	0,00	serine carboxypeptidase 1	Cytoplasm	peptidase
SFXN3	0,00	sideroflexin 3	Cytoplasm	transporter
SFXN2	0,00	sideroflexin 2	Cytoplasm	transporter
CLPTM1	0,00	CLPTM1 regulator of GABA type A receptor forward trafficking	Plasma Membrane	other
LANCL2	0,00	LanC like glutathione S-transferase 2	Plasma Membrane	other
MESD	0,00	mesoderm development LRP chaperone	Extracellular Space	other
HDGFL2	0,00	HDGF like 2	Nucleus	other
IRF2BP1	0,00	interferon regulatory factor 2 binding protein 1	Nucleus	transcription regulator
DDAH1	0,00	dimethylarginine dimethylaminohydrolase 1	Cytoplasm	enzyme
DHRS7	0,00	dehydrogenase/reductase 7	Cytoplasm	enzyme
DTD1	0,00	D-aminoacyl-tRNA deacylase 1	Cytoplasm	enzyme
HERC4	0,00	HECT and RLD domain containing E3 ubiquitin protein ligase 4	Cytoplasm	enzyme
STARD10	0,00	StAR related lipid transfer domain containing 10	Cytoplasm	other
VIL1	0,00	villin 1	Cytoplasm	other
SERPINB9	0,00	serpin family B member 9	Cytoplasm	other
CNPY3	0,00	canopy FGF signaling regulator 3	Cytoplasm	other
CAPN5	0,00	calpain 5	Cytoplasm	peptidase
SLC25A22	0,00	solute carrier family 25 member 22	Cytoplasm	transporter
MISP	0,00	mitotic spindle positioning	Plasma Membrane	other
POGLUT1	0,00	protein O-glucosyltransferase 1	Extracellular Space	enzyme
LAD1	0,00	ladinin 1	Extracellular Space	other
PLBD2	0,00	phospholipase B domain containing 2	Extracellular Space	other
CYP20A1	0,00	cytochrome P450 family 20 subfamily A member 1	Other	enzyme
DNPH1	0,00	2'-deoxynucleoside 5'-phosphate N-hydrolase 1	Nucleus	enzyme
OXR1	0,00	oxidation resistance 1	Cytoplasm	enzyme
DDT	0,00	D-dopachrome tautomerase	Cytoplasm	enzyme
NADK2	0,00	NAD kinase 2, mitochondrial	Cytoplasm	kinase
IKBIP	0,00	IKBKB interacting protein	Cytoplasm	other
LRRC1	0,00	leucine rich repeat containing 1	Cytoplasm	other
ACP6	0,00	acid phosphatase 6, lysophosphatidic	Cytoplasm	phosphatase
NIBAN2	0,00	niban apoptosis regulator 2	Cytoplasm	transcription regulator
TM9SF3	0,00	transmembrane 9 superfamily member 3	Cytoplasm	transporter
ANO10	0,00	anoctamin 10	Plasma Membrane	ion channel
COBLL1	0,00	cordon-bleu WH2 repeat protein like 1	Extracellular Space	other
TINAGL1	0,00	tubulointerstitial nephritis antigen like 1	Extracellular Space	transporter

LMF2	0,00	lipase maturation factor 2	Other	other
VWA5A	0,00	von Willebrand factor A domain containing 5A	Nucleus	other
MFSD10	0,00	major facilitator superfamily domain containing 10	Nucleus	transporter
GALNT7	0,00	polypeptide N-acetylgalactosaminyltransferase 7	Cytoplasm	enzyme
PPIP5K2	0,00	diphosphoinositol pentakisphosphate kinase 2	Cytoplasm	kinase
ARMC10	0,00	armadillo repeat containing 10	Cytoplasm	other
SERPINB6	0,00	serpin family B member 6	Cytoplasm	other
TNFAIP8	0,00	TNF alpha induced protein 8	Cytoplasm	other
FAM162A	0,00	family with sequence similarity 162 member A	Cytoplasm	other
MBOAT7	0,00	membrane bound O-acyltransferase domain containing 7	Plasma Membrane	enzyme
ANO6	0,00	anoctamin 6	Plasma Membrane	ion channel
POGLUT3	0,00	protein O-glucosyltransferase 3	Other	enzyme
ABRACL	0,00	ABRA C-terminal like	Other	other
PCYOX1L	0,00	prenylcysteine oxidase 1 like	Other	other
LDAH	0,00	lipid droplet associated hydrolase	Cytoplasm	enzyme
GALNT3	0,00	polypeptide N-acetylgalactosaminyltransferase 3	Cytoplasm	enzyme
VILL	0,00	villin like	Cytoplasm	other
EML2	0,00	EMAP like 2	Cytoplasm	other

Data 3: List of pathways

BetweennessCentrality	Degree	display name	name	NumberOf Undirected
0.032626856208181496	537	GAPDH	9606.ENSP00000380070	537
0.034634885964155385	492	ACTB	9606.ENSP00000494750	492
0.016219000309822606	436	HSP90AA1	9606.ENSP00000335153	436
0.02005014152322088	433	HSPA8	9606.ENSP00000437125	433
0.012603352228973746	416	HSP90AB1	9606.ENSP00000360609	416
0.012197513506210983	407	HSPA4	9606.ENSP00000302961	407
0.0071405504912215485	379	EEF2	9606.ENSP00000307940	379
0.006678592929379127	356	EFTUD2	9606.ENSP00000392094	356
0.0022583410436458166	341	RPS3	9606.ENSP00000278572	341
0.00552476367278461	336	HNRNPA1	9606.ENSP00000341826	336
0.0034063123696121507	335	RPS20	9606.ENSP00000429374	335
0.003216242600298285	332	RPS2	9606.ENSP00000341885	332
0.007455340005272649	329	NPM1	9606.ENSP00000296930	329
0.0028462202774560664	327	RPLP0	9606.ENSP00000449328	327
0.002018247449203724	320	RPL4	9606.ENSP00000311430	320
0.002586752619606727	319	RPS9	9606.ENSP00000375632	319
0.003423961003313311	319	RPSA	9606.ENSP00000389351	319
0.004396523804928083	317	RACK1	9606.ENSP00000426909	317
0.0016648444137993697	316	RPS16	9606.ENSP00000367806	316
0.008035124772249231	313	EPRS1	9606.ENSP00000355890	313
0.004615828194099861	312	EEF1A1	9606.ENSP00000339063	312
0.010412253434076145	310	VCP	9606.ENSP00000351777	310
0.002166493910947622	309	RPL5	9606.ENSP00000359345	309
0.006315581818315603	299	SRSF1	9606.ENSP00000258962	299
0.002080516675199908	298	RPL11	9606.ENSP00000496250	298
0.0017941232471117186	297	RPL3	9606.ENSP00000346001	297
0.002387050805681876	296	RPS11	9606.ENSP00000270625	296
0.011093004415330262	294	HSPA5	9606.ENSP00000324173	294
0.007949958553893213	293	HSPA9	9606.ENSP00000297185	293
0.0020118530746640454	291	RPL8	9606.ENSP00000262584	291
0.004897953257675654	287	ATP5F1A	9606.ENSP00000381736	287
0.002805391800351992	286	CCT2	9606.ENSP00000299300	286
0.0022650411534148917	286	HNRNPC	9606.ENSP00000451291	286
0.002300450802092406	284	RPS6	9606.ENSP00000369757	284
0.007893092581908024	283	ENO1	9606.ENSP00000495530	283
0.002786303018729959	283	CCT7	9606.ENSP00000258091	283
0.0016831569787440195	282	RPL9	9606.ENSP00000494697	282
0.0023263720424481008	281	RPL23	9606.ENSP00000420311	281
0.0019576159096988868	281	RPS14	9606.ENSP00000385958	281
0.0016343241574105724	280	RPS5	9606.ENSP00000472985	280
0.010542173042702776	278	CTNNB1	9606.ENSP00000495360	278
0.005827645176030023	276	TARDBP	9606.ENSP00000240185	276
0.006731701922340427	274	TPI1	9606.ENSP00000229270	274
0.007360521991329793	273	PHB	9606.ENSP00000479488	273
0.0010342343544792294	272	RPS13	9606.ENSP00000435777	272
0.002374173823640473	271	EIF4A3	9606.ENSP00000497641	271

0.0025906751950421254	270	EEF1G	9606.ENSP00000331901	270
0.0011216899377840765	269	RPS3A	9606.ENSP00000346050	269
0.0021975951811706138	267	HNRNPA2B1	9606.ENSP00000346694	267
0.003524500239490532	266	TCP1	9606.ENSP00000317334	266
0.0018047308802783114	263	EIF2S1	9606.ENSP00000256383	263
0.0010113529933697366	262	RPL7	9606.ENSP00000339795	262
0.0013208317645441398	262	RPL23A	9606.ENSP00000389103	262
0.0029826764909024837	261	CCT5	9606.ENSP00000280326	261
0.0012309747091107637	260	RPS23	9606.ENSP00000296674	260
0.0023387326663557636	259	PABPC1	9606.ENSP00000313007	259
0.003172476558481178	258	HNRNPK	9606.ENSP00000365458	258
0.0021079263657298097	254	CCT4	9606.ENSP00000377958	254
0.001191565559396379	254	RPL12	9606.ENSP00000354739	254
0.0012129933492146202	253	RPL6	9606.ENSP00000403172	253
0.004841413301615871	252	PKM	9606.ENSP00000320171	252
0.0019396317538542443	251	SNRPD2	9606.ENSP00000342374	251
0.0017278041953583435	249	SNU13	9606.ENSP00000383949	249
0.004168885397045824	249	CS	9606.ENSP00000342056	249
0.003472886497408473	249	YBX1	9606.ENSP00000361626	249
0.006753171961741211	248	H4C6	9606.ENSP00000244537	248
0.0011130067105617819	247	RPS8	9606.ENSP00000379888	247
0.004628476864549878	247	SDHA	9606.ENSP00000264932	247
0.0011596702538326858	247	PA2G4	9606.ENSP00000302886	247
0.0023456675267935004	246	DHX9	9606.ENSP00000356520	246
0.00129527112803926	244	EIF4A1	9606.ENSP00000293831	244
0.003825557710994947	243	ATP5F1B	9606.ENSP00000262030	243
0.0019494888998515733	243	CCT8	9606.ENSP00000286788	243
0.0031240543725618345	242	PSMD14	9606.ENSP00000386541	242
0.0010611732771084092	241	RPL7A	9606.ENSP00000361076	241
0.001703831826894782	240	DDX39B	9606.ENSP00000416269	240
0.0016985386692165468	240	RPL26	9606.ENSP00000463784	240
0.0018288977844297324	237	RBM39	9606.ENSP00000253363	237
0.0031146300109934273	236	TUFM	9606.ENSP00000322439	236
0.004523238835609506	236	GART	9606.ENSP00000371253	236
0.0027755600184204195	235	LARP7	9606.ENSP00000422626	235
8.027729676251912E-4	233	RPL19	9606.ENSP00000225430	233
0.0011311346415476906	233	SYNCRIP	9606.ENSP00000358635	233
0.0019517202021438367	232	GFM1	9606.ENSP00000264263	232
7.124086328142934E-4	232	RPL27	9606.ENSP00000464813	232
8.128614110436088E-4	231	RPS19	9606.ENSP00000470972	231
8.650394814144344E-4	231	EIF3B	9606.ENSP00000354125	231
0.0013966981790479909	231	EIF5B	9606.ENSP00000289371	231
0.008195921539108173	231	CDC42	9606.ENSP00000497733	231
0.0038087015975437617	231	ACO2	9606.ENSP00000216254	231
5.592077783847846E-4	229	RPL17	9606.ENSP00000463842	229
7.552842760101719E-4	229	RPL10A	9606.ENSP00000363018	229
6.980367345859609E-4	228	RPS15	9606.ENSP00000466010	228
0.002729202314557531	228	ELAVL1	9606.ENSP00000385269	228

0.0010215219038485724	228	RPL18	9606.ENSP00000447001	228
0.001544185675847802	227	MRPS7	9606.ENSP00000245539	227
0.001941480311837037	227	RUVBL1	9606.ENSP00000318297	227
7.538121566390099E-4	227	RPS4X	9606.ENSP00000362744	227
8.094275520562027E-4	226	EIF3I	9606.ENSP00000362688	226
4.993880492018433E-4	225	RPS12	9606.ENSP00000230050	225
0.0013200513254988948	225	FBL	9606.ENSP00000221801	225
0.004125036461479745	223	HSPD1	9606.ENSP00000340019	223
7.435290398439978E-4	223	RPL18A	9606.ENSP00000222247	223
0.0013564275321467408	223	PSMA3	9606.ENSP00000216455	223
0.006594074756410445	222	SRC	9606.ENSP00000362680	222
0.0010252720213067047	222	RPS28	9606.ENSP00000472469	222
0.002568853333604883	222	NCL	9606.ENSP00000318195	222
0.0016898959567896955	221	CCT3	9606.ENSP00000295688	221
0.0035258624173698273	220	POLR2B	9606.ENSP00000370625	220
5.038668473967316E-4	220	RPL27A	9606.ENSP00000346015	220
8.134155109445077E-4	220	RPL35	9606.ENSP00000259469	220
6.060341235147805E-4	220	RPL30	9606.ENSP00000428085	220
0.0017621028350670361	219	RAN	9606.ENSP00000446215	219
0.005350071377308354	218	VDAC1	9606.ENSP00000378487	218
0.0035011658062677913	217	HNRNPH1	9606.ENSP00000349168	217
0.0020007795366761057	217	ABCE1	9606.ENSP00000296577	217
0.0031545550805850784	217	SDHB	9606.ENSP00000364649	217
9.255479201403712E-4	217	SNRPE	9606.ENSP00000400591	217
7.57506012080461E-4	217	RPS24	9606.ENSP00000414321	217
5.085363602009551E-4	215	RPL13	9606.ENSP00000307889	215
0.006188683523731264	214	SEC61A1	9606.ENSP00000243253	214
4.533951396821984E-4	214	RPS15A	9606.ENSP00000318646	214
0.001626588223349458	214	HNRNPM	9606.ENSP00000325376	214
0.0012962910467687121	213	DHX15	9606.ENSP00000336741	213
0.0022164550228788555	212	ATP5F1C	9606.ENSP00000349142	212
0.0012609974767874114	212	HNRNPR	9606.ENSP00000363745	212
5.973057064788017E-4	212	RPS7	9606.ENSP00000495273	212
0.002447463765124212	211	PRPF8	9606.ENSP00000460348	211
0.0032746672003624044	210	G3BP1	9606.ENSP00000377681	210
0.005341427696600244	210	CDH1	9606.ENSP00000261769	210
7.548322597967093E-4	209	EIF3G	9606.ENSP00000253108	209
0.00865909912156199	209	RHOA	9606.ENSP00000400175	209
0.0016683400186661241	208	NCBP1	9606.ENSP00000364289	208
6.737020163576004E-4	208	RPL31	9606.ENSP00000386717	208
0.0012667810756757905	208	KPNB1	9606.ENSP00000290158	208
5.122841670213131E-4	206	RPL10	9606.ENSP00000413436	206
0.0012282090082151936	206	ILF3	9606.ENSP00000404121	206
3.7689794593733254E-4	205	RPL14	9606.ENSP00000379506	205
0.003147285359767449	204	NEDD8	9606.ENSP00000250495	204
0.003649117872032022	203	HSPE1	9606.ENSP00000233893	203
0.009817535884737275	202	CANX	9606.ENSP00000247461	202
0.001305922907453607	200	PSMC5	9606.ENSP00000310572	200

0.0016755763120104468	199	SNRPF		9606.ENSP00000266735	199
0.001268456359111112	199	PSMA2		9606.ENSP00000223321	199
0.005094037388283547	198	HSP90B1		9606.ENSP00000299767	198
3.985540703554248E-4	198	RPL35A		9606.ENSP00000495672	198
0.0018432188421762061	197	CCT6A		9606.ENSP00000275603	197
0.0010402502299339397	197	HNRNPU		9606.ENSP00000491215	197
0.0022235728350755413	196	MDH2		9606.ENSP00000327070	196
8.837077130478177E-4	195	U2AF2		9606.ENSP00000307863	195
0.003368501879482469	195	HDAC1		9606.ENSP00000362649	195
0.0023787518629284727	195	XPO1		9606.ENSP00000384863	195
0.0024235161182552807	195	NDUFS3		9606.ENSP00000263774	195
0.004102832297033566	195	PARK7		9606.ENSP00000340278	195
0.00250558077071464	195	PGK1		9606.ENSP00000362413	195
0.006150524467875296	194	STAT3		9606.ENSP00000264657	194
0.0015098062853119605	194	DDX5		9606.ENSP00000225792	194
3.268349857516205E-4	194	RPL21		9606.ENSP00000346027	194
3.897254422372742E-4	193	RPS25		9606.ENSP00000435096	193
0.0017903662715860749	191	ATP5PO		9606.ENSP00000290299	191
9.895445721704362E-4	191	SNRPD3		9606.ENSP00000215829	191
0.003130249213197992	190	CLPP		9606.ENSP00000245816	190
0.0016449900566063375	190	FUS		9606.ENSP00000254108	190
2.2968784915018226E-4	190	RPS26		9606.ENSP00000348849	190
3.44482227819903E-4	189	EIF3F		9606.ENSP00000431800	189
6.759591242296018E-4	188	EIF5A		9606.ENSP00000336702	188
0.0019175731688935063	188	POLR2C		9606.ENSP00000219252	188
3.576547201140085E-4	187	RPS10		9606.ENSP00000481646	187
0.0022427841989282175	187	NDUFS8		9606.ENSP00000315774	187
0.003015836693367842	187	H6PD		9606.ENSP00000473348	187
3.9315731738518664E-4	187	RPLP2		9606.ENSP00000431240	187
0.0036895749767987787	187	NDUFAB1		9606.ENSP00000458770	187
0.0013606487230023718	186	HNRNPD		9606.ENSP00000313199	186
7.516369196140541E-4	186	SRSF3		9606.ENSP00000362820	186
0.002077087248091892	186	SOD2		9606.ENSP00000446252	186
3.707024465328563E-4	185	RPL34		9606.ENSP00000378163	185
0.0013147762837938265	183	PCBP1		9606.ENSP00000305556	183
0.001332782131011574	183	PSMC2		9606.ENSP00000391211	183
0.0033707447294652033	183	GSK3B		9606.ENSP00000324806	183
5.161239103733402E-4	182	RPS27		9606.ENSP00000499044	182
0.0055302554938086775	182	COPS5		9606.ENSP00000350512	182
6.238267173281893E-4	181	ETF1		9606.ENSP00000353741	181
0.0017392975113947311	181	EIF4E		9606.ENSP00000425561	181
0.006592246323799698	181	TFRC		9606.ENSP00000376197	181
0.0011959313124069637	181	EIF2S2		9606.ENSP00000364119	181
6.192979218776579E-4	181	PSMA7		9606.ENSP00000359910	181
8.138350150360757E-4	180	PSMA6		9606.ENSP00000261479	180
6.671772820900887E-4	180	PSMC4		9606.ENSP00000157812	180
0.0049375724027960225	179	GRB2		9606.ENSP00000376345	179
0.001485084618550695	179		mar.01	9606.ENSP00000262027	179

2.461226203534082E-4	179	RPL28	9606.ENSP00000452763	179
0.0029519508054533456	179	YARS1	9606.ENSP00000362576	179
0.0011091054071504405	179	NOP56	9606.ENSP00000370589	179
0.003668355298654599	179	SOD1	9606.ENSP00000270142	179
0.004541331408186567	178	MTOR	9606.ENSP00000354558	178
7.855345437540164E-4	177	PSMA5	9606.ENSP00000271308	177
0.0016727670642810587	177	COX4I1	9606.ENSP00000457513	177
0.0010019395439133405	177	ILF2	9606.ENSP00000355011	177
0.001194204646849158	177	PSMD2	9606.ENSP00000310129	177
9.80903467280188E-4	177	SNRNP70	9606.ENSP00000472998	177
0.002869624032359678	177	RANBP2	9606.ENSP00000283195	177
0.0023072709971110154	176	SUCLG1	9606.ENSP00000377446	176
0.0019051372124233234	175	HSPA1B	9606.ENSP00000364801	175
0.006807684642700758	175	APP	9606.ENSP00000284981	175
9.875570223196873E-4	175	PSMD1	9606.ENSP00000309474	175
0.001350135036012752	175	PDHA1	9606.ENSP00000369134	175
2.245502315153935E-4	175	RPS21	9606.ENSP00000345957	175
6.757273482616259E-4	174	PTBP1	9606.ENSP00000349428	174
0.0020287088360973027	173	TRIM28	9606.ENSP00000253024	173
0.0013819388749925672	173	CHD4	9606.ENSP00000440542	173
0.005578805153473204	173	CLTC	9606.ENSP00000479606	173
0.001273536588806241	173	ATP5F1D	9606.ENSP00000215375	173
9.085861953331735E-4	171	EIF3A	9606.ENSP00000358140	171
0.0012981793134318627	171	FH	9606.ENSP00000355518	171
0.005202167188496696	171	SEC13	9606.ENSP00000373312	171
0.0024873612235863725	171	IDH1	9606.ENSP00000390265	171
0.0013060388070606185	171	EIF4G1	9606.ENSP00000416255	171
0.004340214737421364	171	FN1	9606.ENSP00000346839	171
6.121704900280295E-4	171	EIF4A2	9606.ENSP00000326381	171
0.0034533244440514976	171	MAPK3	9606.ENSP00000263025	171
9.253037654401124E-4	171	DDX3X	9606.ENSP00000494040	171
0.0036794074621323116	170	ALDH18A1	9606.ENSP00000360268	170
9.781596431900148E-4	169	SERBP1	9606.ENSP00000360034	169
7.401220343038173E-4	169	PSMC1	9606.ENSP00000261303	169
0.0030796480621314783	169	YWHAZ	9606.ENSP00000379287	169
8.095422791530664E-4	169	MTREX	9606.ENSP00000230640	169
8.221268973426007E-4	169	RPL32	9606.ENSP00000416429	169
0.00193772844039821	169	CDC5L	9606.ENSP00000360532	169
0.001166626078155256	168	SF3B1	9606.ENSP00000335321	168
4.766222662840751E-4	167	RPS17	9606.ENSP00000498019	167
0.0020809761591586906	167	UBQLN1	9606.ENSP00000365576	167
2.608431993612991E-4	167	RPL38	9606.ENSP00000309830	167
6.920959074125255E-4	167	PSMA1	9606.ENSP00000414359	167
4.6455666677803386E-4	166	EIF6	9606.ENSP00000363559	166
0.0013984114033995776	166	RUVBL2	9606.ENSP00000473172	166
0.0018031913058169989	166	DLD	9606.ENSP00000205402	166
0.0011928352673371753	166	UPF1	9606.ENSP00000470142	166
2.6447785350196165E-4	166	RPL24	9606.ENSP00000377640	166

0.0028790869075092135	166 NFKB1	9606.ENSP00000226574	166
0.0011756263223669002	166 KARS1	9606.ENSP00000325448	166
0.004979613218565146	166 TXN	9606.ENSP00000363641	166
6.336782510369076E-4	166 SNRPD1	9606.ENSP00000300413	166
0.0013250087394584642	166 RAD23B	9606.ENSP00000350708	166
0.0047049416425361335	165 CALR	9606.ENSP00000320866	165
0.0010744012848906798	165 HNRNPL	9606.ENSP00000221419	165
0.002434054312122707	165 GMPS	9606.ENSP00000419851	165
0.0018219471720176178	165 SUMO1	9606.ENSP00000376077	165
6.120098139681542E-4	164 NOP58	9606.ENSP00000264279	164
8.849114415377328E-4	164 DDX21	9606.ENSP00000346120	164
4.891303500645594E-4	164 BTF3	9606.ENSP00000369965	164
2.4066614987594822E-4	163 RPL22	9606.ENSP00000346088	163
6.413579146925411E-4	163 MRPL24	9606.ENSP00000354525	163
7.809148336261086E-4	163 U2AF1	9606.ENSP00000291552	163
7.651353962578603E-4	162 SRSF2	9606.ENSP00000376276	162
0.0010816863918657424	162 POLR1C	9606.ENSP00000496044	162
0.0016603341153460895	161 LDHA	9606.ENSP00000445175	161
0.001836506856113708	161 IDH2	9606.ENSP00000331897	161
2.0843989535601734E-4	161 RPL37A	9606.ENSP00000418082	161
5.360644376206206E-4	161 PSMA4	9606.ENSP00000044462	161
3.050407840461181E-4	160 EIF3M	9606.ENSP00000436049	160
0.0013736905167162777	159 UQCRFS1	9606.ENSP00000306397	159
0.0014703602106117568	158 GPI	9606.ENSP00000405573	158
4.6437407579763963E-4	158 PSMD11	9606.ENSP00000261712	158
1.454145562067258E-4	158 EIF3E	9606.ENSP00000220849	158
0.0022284139855804066	158 SDHC	9606.ENSP00000356953	158
0.0014487093001380135	157 SRP54	9606.ENSP00000451818	157
0.0012578092126773546	157 PDHB	9606.ENSP00000307241	157
0.0017760590875884911	157 ACLY	9606.ENSP00000466259	157
5.160914451466822E-4	157 QARS1	9606.ENSP00000307567	157
0.004393578228950641	156 ARF1	9606.ENSP00000440005	156
3.921125420676365E-4	156 SNRPA	9606.ENSP00000243563	156
0.0016426954193403598	155 UQCRC1	9606.ENSP00000203407	155
0.003054521105404286	155 PARP1	9606.ENSP00000355759	155
0.0010180023778126506	155 KHDRBS1	9606.ENSP00000313829	155
6.590212470755124E-4	155 EIF4H	9606.ENSP00000265753	155
9.046837228141745E-4	154 PSMD4	9606.ENSP00000357876	154
7.301043126377087E-4	153 HNRNPDL	9606.ENSP00000483254	153
0.0014987670513084575	152 GTPBP4	9606.ENSP00000354040	152
4.861265582738715E-4	152 EIF3C	9606.ENSP00000332604	152
4.2103421196279436E-4	151 SRSF6	9606.ENSP00000244020	151
0.00278594686873948	151 MAPK1	9606.ENSP00000215832	151
0.0016025469662543864	150 NDUFV1	9606.ENSP00000497587	150
9.059880892231727E-4	150 ACO1	9606.ENSP00000309477	150
0.001990154654444316	150 SEC61B	9606.ENSP00000223641	150
4.0110530828561136E-4	149 SRSF7	9606.ENSP00000325905	149
0.0011072780951909534	149 SF3B3	9606.ENSP00000305790	149

0.001174982497854003	148	VARS1	9606.ENSP00000364815	148
0.0024206373202549777	148	SHMT2	9606.ENSP00000333667	148
7.115140641353537E-4	148	PRPF19	9606.ENSP00000227524	148
0.001030248232498771	148	PPIA	9606.ENSP00000419425	148
6.996306513581879E-4	148	SNRPA1	9606.ENSP00000254193	148
8.29272490745675E-4	148	CPSF6	9606.ENSP00000266679	148
8.957492117798204E-4	148	NDUFS2	9606.ENSP00000356972	148
3.022402611735913E-4	148	TRA2B	9606.ENSP00000416959	148
0.0016073208706235663	147	NDUFS1	9606.ENSP00000392709	147
7.394835151834134E-4	147	PSMC3	9606.ENSP00000481029	147
4.800240567657057E-4	145	SSB	9606.ENSP00000386636	145
4.512087802692498E-4	145	PSMD12	9606.ENSP00000348442	145
0.0026916013185546693	144	CFL1	9606.ENSP00000432660	144
0.0011895156584997286	144	SRSF9	9606.ENSP00000229390	144
9.48338511394653E-4	144	UQCRC2	9606.ENSP00000268379	144
5.879110372931606E-4	144	PSMD7	9606.ENSP00000219313	144
5.668087540564215E-4	143	SFPQ	9606.ENSP00000349748	143
1.461963650948295E-4	142	EIF3D	9606.ENSP00000216190	142
4.033655137747524E-4	142	RPS27L	9606.ENSP00000331019	142
0.0016728517473084453	142	GOT2	9606.ENSP00000245206	142
2.2999539066235165E-4	141	PFDN5	9606.ENSP00000447942	141
4.087177126136083E-4	141	DRG1	9606.ENSP00000329715	141
2.3455724241517068E-4	140	EEF1D	9606.ENSP00000410059	140
0.003807114660129412	140	ANXA2	9606.ENSP00000346032	140
1.3490108668552756E-4	140	RPLP1	9606.ENSP00000346037	140
0.00478057711723418	140	RAB5A	9606.ENSP00000273047	140
4.694719011025591E-4	140	TSFM	9606.ENSP00000313877	140
8.772080418359643E-4	140	DLAT	9606.ENSP00000280346	140
0.0020994315886231026	139	IQGAP1	9606.ENSP00000268182	139
0.00383096088268806	139	PRKACA	9606.ENSP00000309591	139
6.094078037954383E-4	139	TNPO1	9606.ENSP00000336712	139
0.002624478130129309	139	FLNA	9606.ENSP00000358866	139
0.0025759934743324374	139	RELA	9606.ENSP00000384273	139
0.0033726664988846136	138	SQSTM1	9606.ENSP00000374455	138
7.104720378470558E-4	137	SNRNP200	9606.ENSP00000317123	137
0.001517089351768733	137	PPA1	9606.ENSP00000362329	137
0.0010325148516982059	136	DKC1	9606.ENSP00000358563	136
0.0013903797143098027	136	POLR2A	9606.ENSP00000461879	136
9.714600975655336E-4	136	DDX6	9606.ENSP00000478754	136
5.806985824299415E-4	136	SF3A1	9606.ENSP00000215793	136
4.7311692838211694E-4	135	SF3A3	9606.ENSP00000362110	135
9.099266026970926E-4	135	MRPS16	9606.ENSP00000362036	135
0.0034382127952674664	135	CAPZB	9606.ENSP00000401010	135
7.461248646967452E-4	134	GCN1	9606.ENSP00000300648	134
2.634676686193977E-4	134	PABPN1	9606.ENSP00000216727	134
0.002659566337528434	134	YWHAB	9606.ENSP00000361930	134
0.0028598875569076218	134	VCL	9606.ENSP00000211998	134
7.010549413461918E-4	133	DDX17	9606.ENSP00000380033	133

8.695635901400694E-4	133	CYC1	9606.ENSP00000317159	133
0.001880290861524578	133	IMPDH2	9606.ENSP00000321584	133
0.0038501514231864117	133	FASN	9606.ENSP00000304592	133
8.813282188696771E-4	133	NDUFV2	9606.ENSP00000327268	133
0.0017494045847929022	133	UBE2I	9606.ENSP00000348056	133
0.0010192835002308441	132	ECHS1	9606.ENSP00000357535	132
0.0023686539582924763	132	VDAC2	9606.ENSP00000361635	132
2.675043648287737E-4	132	TPT1	9606.ENSP00000477781	132
5.005250343741915E-4	131	SF3B2	9606.ENSP00000318861	131
0.0036218205006247526	131	PDIA3	9606.ENSP00000300289	131
0.0016809127988080053	131	PHB2	9606.ENSP00000441875	131
8.175239072660765E-4	130	KHSRP	9606.ENSP00000381216	130
2.8003064293514855E-4	130	SRRM1	9606.ENSP00000326261	130
0.0016690754947700631	130	GSR	9606.ENSP00000221130	130
8.067139114793238E-4	129	SF1	9606.ENSP00000366604	129
1.0645754444782196E-4	129	EIF3H	9606.ENSP00000429931	129
2.672725061792375E-4	129	HNRNPA3	9606.ENSP00000376309	129
2.1956749257467776E-4	129	HNRNPF	9606.ENSP00000400433	129
0.003760736646289417	128	LAMP1	9606.ENSP00000333298	128
6.081984064804956E-4	128	NDUFA9	9606.ENSP00000266544	128
0.0010066345202296054	127	PRDX1	9606.ENSP00000262746	127
0.001243590450181888	127	TKT	9606.ENSP00000391481	127
5.1687655462009E-4	127	PSMC6	9606.ENSP00000484998	127
3.565124670378314E-4	127	PES1	9606.ENSP00000346725	127
0.0013184157594560125	127	TPR	9606.ENSP00000356448	127
0.0012842707210404555	127	SKP1	9606.ENSP00000231487	127
0.0011101757581256845	127	STAU1	9606.ENSP00000360922	127
6.473274090684171E-4	127	DDX1	9606.ENSP00000370745	127
0.002433402792888477	126	RAB7A	9606.ENSP00000265062	126
2.7774007031020656E-4	126	ERH	9606.ENSP00000451080	126
3.796574294505122E-4	126	DDX18	9606.ENSP00000263239	126
0.0012844984785715004	126	NME1	9606.ENSP00000337060	126
1.8157648700874862E-4	125	RSL1D1	9606.ENSP00000460871	125
0.002421218938472007	125	CSNK2A1	9606.ENSP00000217244	125
0.0021965606573108747	125	YWHAE	9606.ENSP00000264335	125
0.0012139210229739758	124	ETFA	9606.ENSP00000452762	124
7.964505100675582E-4	124	TALDO1	9606.ENSP00000321259	124
0.0018833748878322642	124	P4HB	9606.ENSP00000327801	124
9.237167118267113E-5	124	EIF3L	9606.ENSP00000499067	124
0.0011023882876686538	124	XRCC6	9606.ENSP00000352257	124
0.0019682892528434465	123	PHGDH	9606.ENSP00000493382	123
0.001998195280288008	123	ITGB1	9606.ENSP00000379350	123
5.916995636844743E-4	123	SNW1	9606.ENSP00000451129	123
9.365646363974543E-4	123	PMPCB	9606.ENSP00000249269	123
0.0022391400163656143	123	CUL3	9606.ENSP00000264414	123
0.001955667761261227	122	RPN1	9606.ENSP00000296255	122
0.0028518029109316444	122	ANXA5	9606.ENSP00000296511	122
7.062057503797851E-4	122	HADHB	9606.ENSP00000325136	122

0.0017820318167433723	122	TP53BP1	9606.ENSP00000371475	122
8.306332604436111E-4	122	SRRT	9606.ENSP00000480421	122
4.323699958530834E-4	122	NONO	9606.ENSP00000276079	122
2.0130739420027758E-4	122	RNPS1	9606.ENSP00000457723	122
9.551086209703909E-4	122	USP14	9606.ENSP00000261601	122
6.018106257122285E-4	122	EWSR1	9606.ENSP00000400142	122
2.4065227706361123E-4	122	METAP2	9606.ENSP00000325312	122
5.314115285104259E-4	121	IARS1	9606.ENSP00000364794	121
0.0016928265595963182	121	PFN1	9606.ENSP00000225655	121
4.085506910699904E-4	120	AIMP1	9606.ENSP00000378191	120
0.0030026661861626627	120	EZR	9606.ENSP00000338934	120
8.07260629765651E-4	120	CUL1	9606.ENSP00000326804	120
0.0012816480556660569	120	NUDC	9606.ENSP00000319664	120
0.0011189666955809113	119	PPA2	9606.ENSP00000343885	119
5.028415382711247E-4	118	EIF5	9606.ENSP00000216554	118
0.00182288759619193	118	ATIC	9606.ENSP00000236959	118
7.090058621796063E-4	118	XRCC5	9606.ENSP00000375978	118
0.003395962493874675	118	PPP1CA	9606.ENSP00000326031	118
0.0011545844659165189	118	HADHA	9606.ENSP00000370023	118
3.4711301374358973E-4	117	OGDH	9606.ENSP00000388183	117
0.001765990330230011	117	RAC1	9606.ENSP00000348461	117
9.803809975732837E-5	117	HNRNPA0	9606.ENSP00000316042	117
5.396826651353766E-4	117	STT3A	9606.ENSP00000376472	117
0.0022715408472187802	116	MYH9	9606.ENSP00000216181	116
0.0011935692782234786	116	DNAJC10	9606.ENSP00000264065	116
0.001402288946845491	116	PAK2	9606.ENSP00000314067	116
3.65707452360971E-4	116	DLST	9606.ENSP00000335304	116
1.3812230906415512E-4	116	PSMB7	9606.ENSP00000259457	116
8.767843818856682E-4	116	MEPCE	9606.ENSP00000308546	116
7.248473956573544E-4	116	NUP153	9606.ENSP00000444029	116
3.075131379726724E-4	116	SUCLA2	9606.ENSP00000494360	116
0.0028227793810432188	116	PPP2R1A	9606.ENSP00000324804	116
0.0010756149350301509	115	SEC61G	9606.ENSP00000388337	115
8.500066527333705E-4	115	ACAA2	9606.ENSP00000285093	115
4.527169199186888E-4	115	COX5B	9606.ENSP00000258424	115
6.829415119669961E-4	115	PRDX3	9606.ENSP00000298510	115
7.217082048387107E-4	114	TOP1	9606.ENSP00000354522	114
6.550808070779934E-4	114	NAT10	9606.ENSP00000257829	114
1.502960437407189E-4	114	SNRPC	9606.ENSP00000244520	114
9.853633351066327E-4	114	STUB1	9606.ENSP00000219548	114
5.788543719002434E-4	114	PRPF40A	9606.ENSP00000386458	114
0.0011903266912859577	114	AFG3L2	9606.ENSP00000269143	114
8.806621118675653E-4	114	NUP98	9606.ENSP00000316032	114
0.0025706866549440465	114	COPB1	9606.ENSP00000249923	114
1.8981755154711218E-4	113	TIAL1	9606.ENSP00000358089	113
5.705131529087488E-4	113	NUDT21	9606.ENSP00000300291	113
4.585775114640486E-4	113	PSME3	9606.ENSP00000293362	113
0.0018732994369105617	113	PRMT1	9606.ENSP00000406162	113

8.521867601875864E-4	113	XRN2	9606.ENSP00000366396	113
2.1159109111008637E-4	112	EIF4B	9606.ENSP00000388806	112
0.0022356302565008975	112	CD44	9606.ENSP00000398632	112
0.0010050907072848617	112	HUWE1	9606.ENSP00000340648	112
0.0012769312173376425	112	PEBP1	9606.ENSP00000261313	112
4.834977906495409E-4	112	COX5A	9606.ENSP00000317780	112
0.0014570228003045406	112	VAMP3	9606.ENSP00000054666	112
2.9456582372290504E-4	111	THOC2	9606.ENSP00000245838	111
2.0884373297578085E-4	111	IDH3A	9606.ENSP00000299518	111
3.603344210660956E-4	111	SKIV2L	9606.ENSP00000364543	111
5.462452282389245E-4	110	HSPA2	9606.ENSP00000378199	110
8.932211979005194E-5	110	GSPT1	9606.ENSP00000398131	110
0.0010630144901474555	110	UBA1	9606.ENSP00000338413	110
2.1251726035922253E-4	110	SRSF4	9606.ENSP00000362900	110
6.164697215202983E-4	110	GLUD1	9606.ENSP00000277865	110
0.0017893372222622843	110	ARCN1	9606.ENSP00000264028	110
0.0016563546994986184	110	LMNB1	9606.ENSP00000261366	110
7.746031349389277E-4	110	NAA10	9606.ENSP00000417763	110
5.942892430252191E-4	110	IDH3G	9606.ENSP00000217901	110
7.424259432865195E-4	109	STT3B	9606.ENSP00000295770	109
1.9501568505767122E-4	109	SF3A2	9606.ENSP00000221494	109
8.60813768133755E-4	109	ACADM	9606.ENSP00000359871	109
0.0013500562031619023	109	SUCLG2	9606.ENSP00000419325	109
0.0016879311056303766	109	ELOC	9606.ENSP00000478121	109
9.123265146452566E-4	109	METAP1	9606.ENSP00000296411	109
3.543779775034423E-4	109	PDHX	9606.ENSP00000227868	109
0.0023033220297936758	108	ARF6	9606.ENSP00000298316	108
2.5178565347474917E-4	108	EIF3K	9606.ENSP00000248342	108
0.0020027740570895992	108	RAB1A	9606.ENSP00000387286	108
7.671838574370575E-4	107	SSBP1	9606.ENSP00000419665	107
2.3456421762048236E-4	107	CTNNBL1	9606.ENSP00000355050	107
0.0016416872613116086	107	PRDX2	9606.ENSP00000301522	107
6.590540198398318E-4	107	NDUFA8	9606.ENSP00000362873	107
1.1604623729445178E-4	107	SNRPB2	9606.ENSP00000246071	107
0.0019490076869636067	107	LMAN1	9606.ENSP00000251047	107
2.6458032425471026E-4	107	SRRM2	9606.ENSP00000301740	107
0.0020818008240296388	106	RAB6A	9606.ENSP00000311449	106
7.036470570666108E-4	106	NDUFA5	9606.ENSP00000417142	106
8.848215009970106E-4	106	SHMT1	9606.ENSP00000318868	106
0.0013574591402203725	106	NF1	9606.ENSP00000351015	106
3.9619892887593787E-4	106	DHX38	9606.ENSP00000268482	106
0.0010733942518813908	106	DNAJB1	9606.ENSP00000254322	106
0.002119758195789427	106	DCTN1	9606.ENSP00000354791	106
4.6953592932800575E-4	106	DDX23	9606.ENSP00000310723	106
7.997996255902974E-4	105	KPNA2	9606.ENSP00000438483	105
4.025340378174736E-4	105	STIP1	9606.ENSP00000351646	105
0.0012521005016694496	105	HDAC6	9606.ENSP00000365804	105
8.175981859118387E-4	105	FMR1	9606.ENSP00000359506	105

0.001399155068554077	105	TOMM20	9606.ENSP00000355566	105
0.0015372816769561569	105	PTGES3	9606.ENSP00000482075	105
6.922954546668227E-4	104	HSPB1	9606.ENSP00000248553	104
0.0014269255373114689	104	CTTN	9606.ENSP00000365745	104
0.002464264670604623	104	GOLPH3	9606.ENSP00000265070	104
8.627288313575582E-4	104	FXR1	9606.ENSP00000350170	104
6.847017042553164E-4	104	NUP62	9606.ENSP00000471191	104
7.853655118294043E-4	104	TFAM	9606.ENSP00000420588	104
2.0568142690094438E-4	104	OLA1	9606.ENSP00000284719	104
8.242890473138561E-4	103	UBE3A	9606.ENSP00000497594	103
5.99388562710727E-5	103	RBM25	9606.ENSP00000261973	103
8.241940074547115E-4	103	SSRP1	9606.ENSP00000278412	103
5.295095671904937E-4	103	AHCY	9606.ENSP00000217426	103
3.1650872876861974E-4	103	DRG2	9606.ENSP00000225729	103
0.001816337092117721	103	TMPO	9606.ENSP00000266732	103
6.704018947331076E-4	103	LARS1	9606.ENSP00000377954	103
6.297889647470471E-4	103	UBE2N	9606.ENSP00000316176	103
3.296497368859059E-4	103	PSMD8	9606.ENSP00000215071	103
5.121454739687461E-4	103	PFDN2	9606.ENSP00000356989	103
2.1441428249988125E-4	103	SRP68	9606.ENSP00000312066	103
0.0010300856337701098	103	CFL2	9606.ENSP00000298159	103
4.773439920190162E-4	102	LSM3	9606.ENSP00000302160	102
5.339461865384835E-4	102	NUP214	9606.ENSP00000352400	102
7.749702118221879E-4	102	HDAC2	9606.ENSP00000430432	102
4.0352075008030693E-4	102	NDUFB8	9606.ENSP00000299166	102
4.91151584429392E-4	102	SPCS2	9606.ENSP00000263672	102
5.658654726608196E-4	102	IPO7	9606.ENSP00000369042	102
2.0283034708412802E-4	102	SRSF11	9606.ENSP00000359988	102
3.5406778640217337E-4	102	NDUFS7	9606.ENSP00000233627	102
4.7905219944601325E-4	101	FTSJ3	9606.ENSP00000396673	101
2.997434470630283E-4	101	ATP5PB	9606.ENSP00000358737	101
1.167769494333261E-4	101	NACA	9606.ENSP00000448035	101
0.001032092687916105	101	ALDOA	9606.ENSP00000496166	101
0.0010319911907262715	101	DDB1	9606.ENSP00000301764	101
7.572259186430233E-4	100	EIF2AK2	9606.ENSP00000233057	100
0.0012319735063613356	100	ACTN4	9606.ENSP00000252699	100
0.0017841281543874478	100	SEC63	9606.ENSP00000357998	100
8.722766369034039E-4	100	MYBBP1A	9606.ENSP00000370968	100
0.0026373481258203145	100	TSG101	9606.ENSP00000251968	100
2.1874471655206412E-4	100	FARSB	9606.ENSP00000281828	100
5.187070468491579E-4	100	NDUFA13	9606.ENSP00000423673	100
3.019685233436531E-4	100	ZC3H18	9606.ENSP00000416951	100
2.792117176222246E-4	99	NDUFB9	9606.ENSP00000276689	99
4.5987849844982383E-4	99	PGD	9606.ENSP00000270776	99
0.001105092673025167	99	PRDX6	9606.ENSP00000342026	99
0.0015854270777673353	99	CAPZA1	9606.ENSP00000263168	99
4.915493364144676E-4	99	TRAP1	9606.ENSP00000246957	99
4.8245380283367154E-4	99	NUP107	9606.ENSP00000229179	99

1.1196749954680631E-4	99	HNRNPAB	9606.ENSP00000425031	99
0.0010701894944449984	98	USP7	9606.ENSP00000343535	98
5.245854986003725E-4	98	GRPEL1	9606.ENSP00000264954	98
8.010981262223542E-4	98	SMARCA4	9606.ENSP00000343896	98
1.83695612243366E-4	98	RARS1	9606.ENSP00000231572	98
0.00166261849609563	98	GOLGA2	9606.ENSP00000416097	98
4.794842662908252E-4	98	RAE1	9606.ENSP00000379181	98
0.0016636911136079537	98	TOMM40	9606.ENSP00000410339	98
3.121325215716622E-4	98	PRPF6	9606.ENSP00000266079	98
3.2567809372525416E-4	98	PSMB1	9606.ENSP00000262193	98
5.297263671712562E-4	97	IARS2	9606.ENSP00000355889	97
1.5524607336981616E-4	97	DDX39A	9606.ENSP00000242776	97
6.060476629954896E-4	97	PCCB	9606.ENSP00000419027	97
6.81060366214299E-4	97	RBBP7	9606.ENSP00000369424	97
3.136103937356033E-4	97	IDH3B	9606.ENSP00000482773	97
8.56345204255407E-4	97	MAT2B	9606.ENSP00000325425	97
2.195645276402959E-4	96	HBS1L	9606.ENSP00000356811	96
2.133512823319622E-4	96	SPCS1	9606.ENSP00000233025	96
2.559072829810205E-4	96	NDUFS4	9606.ENSP00000296684	96
0.0010001057802838298	96	BAG3	9606.ENSP00000358081	96
0.0013431493079231454	96	CAPZA2	9606.ENSP00000354947	96
9.775425073381752E-4	96	GLO1	9606.ENSP00000362463	96
0.0012058595028229956	96	YWHAG	9606.ENSP00000306330	96
1.8444134591115246E-4	96	HEATR1	9606.ENSP00000355541	96
2.074632410168134E-4	95	SF3B6	9606.ENSP00000233468	95
0.00116959234654338	95	NAPA	9606.ENSP00000263354	95
5.371802947867196E-4	95	PAICS	9606.ENSP00000382595	95
0.001262726058044243	95	ACTR2	9606.ENSP00000367220	95
8.350908917452985E-4	95	ADSL	9606.ENSP00000485525	95
0.0015339664712290563	94	NSF	9606.ENSP00000381293	94
4.643170635297755E-4	94	ACAD9	9606.ENSP00000312618	94
5.431643848827031E-4	94	AK2	9606.ENSP00000499935	94
0.0014373270757236114	94	ATG7	9606.ENSP00000346437	94
5.383995346051049E-4	94	SMARCA5	9606.ENSP00000283131	94
0.0013992138642050164	94	SRPRA	9606.ENSP00000328023	94
0.001293994956222703	93	LMNA	9606.ENSP00000357283	93
3.6098631855877684E-4	93	DIS3	9606.ENSP00000366997	93
0.001709850651453142	93	TUBA1C	9606.ENSP00000443475	93
8.058395010713007E-4	93	DNAJA1	9606.ENSP00000369127	93
3.3201558047249945E-4	93	COPS6	9606.ENSP00000304102	93
2.1457898664109168E-4	93	PDCD5	9606.ENSP00000466214	93
1.5659224774802706E-4	93	SRP19	9606.ENSP00000424870	93
0.0013685308608361669	93	ACTN1	9606.ENSP00000377941	93
0.0011221110462760844	93	TLN1	9606.ENSP00000316029	93
3.3372520814051784E-4	93	CDC37	9606.ENSP00000222005	93
3.0463576319487133E-4	92	NUP205	9606.ENSP00000285968	92
5.117199754727414E-4	92	ADSS2	9606.ENSP00000355493	92
0.0014529107225850478	92	BCL2L1	9606.ENSP00000365230	92

1.727408735610831E-4	92	SF3B4	9606.ENSP00000271628	92
0.0012080138204982752	92	STAT1	9606.ENSP00000354394	92
1.6424442633462951E-4	92	PSMD6	9606.ENSP00000418695	92
3.1925708907865177E-4	92	NDUFA10	9606.ENSP00000252711	92
8.120084029885579E-4	92	SEH1L	9606.ENSP00000382779	92
0.0012517264036787463	92	PPIB	9606.ENSP00000300026	92
3.7121675509376015E-4	91	UQCRB	9606.ENSP00000430494	91
0.0013192842417332623	91	DNM1L	9606.ENSP00000449089	91
9.041276598966649E-4	91	PRKCA	9606.ENSP00000408695	91
7.413539426247513E-4	91	WASL	9606.ENSP00000223023	91
2.2227711188182753E-4	91	WDR61	9606.ENSP00000453801	91
3.208848721597572E-4	91	NDUFB10	9606.ENSP00000268668	91
1.9966413214803194E-4	91	BCLAF1	9606.ENSP00000435210	91
3.424076769632875E-4	90	RANBP1	9606.ENSP00000401564	90
4.698185680367276E-4	90	HSDL2	9606.ENSP00000381785	90
0.001181107814851269	90	YKT6	9606.ENSP00000223369	90
0.0011809087987357996	90	RAB8A	9606.ENSP00000300935	90
0.0012146737436133056	90	PCNA	9606.ENSP00000368458	90
4.0446957449818277E-4	90	NUP93	9606.ENSP00000310668	90
1.7633928779649935E-4	90	DDX46	9606.ENSP00000416534	90
4.3729817064334746E-4	89	PSMB4	9606.ENSP00000290541	89
0.0013762026671194586	89	DDOST	9606.ENSP00000399457	89
2.2140330234781313E-4	89	FUBP1	9606.ENSP00000359804	89
0.002873135075642854	89	NUDCD1	9606.ENSP00000239690	89
7.035304404648484E-4	89	MAP2K1	9606.ENSP00000302486	89
4.634710562720039E-4	89	NDUFB7	9606.ENSP00000215565	89
3.827560354160601E-4	89	PMPCA	9606.ENSP00000360782	89
3.7605493983728624E-4	89	SLC25A3	9606.ENSP00000228318	89
0.0014775772254951123	89	ALDH3A2	9606.ENSP00000345774	89
4.99133502410642E-4	88	VDAC3	9606.ENSP00000428845	88
4.40873656506177E-4	88	PTMA	9606.ENSP00000344547	88
3.533217258009418E-4	88	PFKM	9606.ENSP00000496597	88
8.20972163427811E-4	88	ACADVL	9606.ENSP00000438689	88
0.001290176274538672	88	TMED10	9606.ENSP00000303145	88
3.376239343791576E-4	88	NDUFA12	9606.ENSP00000330737	88
3.9682150548608317E-4	88	LONP1	9606.ENSP00000353826	88
0.0015288986171021251	88	SPG7	9606.ENSP00000495795	88
4.508345773772035E-4	88	PUF60	9606.ENSP00000434359	88
2.0550921900650603E-4	87	XAB2	9606.ENSP00000351137	87
7.766496321977315E-4	87	TXNRD2	9606.ENSP00000383365	87
4.477084603549496E-4	87	POLR2H	9606.ENSP00000415536	87
8.103554325936764E-4	87	SLC25A5	9606.ENSP00000360671	87
6.187726920840402E-4	87	NUP54	9606.ENSP00000264883	87
2.9148981747974663E-4	87	HNRNPH2	9606.ENSP00000361927	87
3.033992650923292E-4	87	LUC7L3	9606.ENSP00000376919	87
6.989820208201615E-4	87	RANGAP1	9606.ENSP00000401470	87
0.0013047126533063916	87	KIF5B	9606.ENSP00000307078	87
2.814151415078941E-4	87	NUP155	9606.ENSP00000231498	87

7.001109918258456E-4	87	DNAJB11	9606.ENSP00000414398	87
1.8550325627929323E-4	86	LRRC47	9606.ENSP00000367498	86
2.2399614947412165E-4	86	NOP2	9606.ENSP00000371858	86
8.619707991144245E-4	86	ACTR3	9606.ENSP00000263238	86
3.665135720234296E-5	86	HNRNPH3	9606.ENSP00000265866	86
0.0010737065903104654	86	SNAP23	9606.ENSP00000249647	86
0.001567748056317288	86	SCARB2	9606.ENSP00000264896	86
8.817933887077064E-4	86	CUL4A	9606.ENSP00000364589	86
3.1544693744507815E-4	86	CPSF1	9606.ENSP00000484669	86
8.607683399683107E-4	85	SEC22B	9606.ENSP00000463393	85
6.884611720276559E-4	85	HADH	9606.ENSP00000474560	85
3.575139491449519E-4	85	CUL2	9606.ENSP00000414095	85
2.978282156016396E-4	85	FARSA	9606.ENSP00000320309	85
2.5969049955878385E-4	85	SMU1	9606.ENSP00000380336	85
2.687481958367973E-4	85	SART1	9606.ENSP00000310448	85
6.187572496657615E-4	85	ACAT1	9606.ENSP00000265838	85
1.7997046944520602E-4	85	NUP160	9606.ENSP00000367721	85
0.0013064492049986362	85	HYOU1	9606.ENSP00000480150	85
2.0001149652292075E-4	85	PGAM1	9606.ENSP00000359991	85
0.0013651738159821728	85	B2M	9606.ENSP00000497910	85
2.959923929301377E-4	84	BCKDHA	9606.ENSP00000269980	84
1.6838066950966243E-4	84	PSMD3	9606.ENSP00000264639	84
0.002731274724529755	84	FDX1	9606.ENSP00000260270	84
0.0013400326273160779	84	NUP88	9606.ENSP00000458954	84
4.0581276475394466E-4	84	ACTL6A	9606.ENSP00000397552	84
3.6359357273241683E-4	84	NDUFS5	9606.ENSP00000362058	84
6.913216683185644E-4	84	SHC1	9606.ENSP00000401303	84
5.125009076453382E-4	84	SAMM50	9606.ENSP00000345445	84
0.0015049208808695404	84	VPS35	9606.ENSP00000299138	84
9.298594055138007E-4	84	TMED2	9606.ENSP00000262225	84
1.0548014755777814E-4	84	LSM8	9606.ENSP00000249299	84
3.6536838111660147E-4	83	NDUFA6	9606.ENSP00000482543	83
1.8567235340175099E-4	83	EIF4G2	9606.ENSP00000433664	83
0.0012472633427174009	83	TRIP12	9606.ENSP00000373696	83
5.326736381324241E-4	83	ALDH2	9606.ENSP00000261733	83
5.616547720966927E-4	83	PDIA4	9606.ENSP00000499129	83
0.0012401639797348383	83	NSDHL	9606.ENSP00000359297	83
3.220512276272051E-4	82	COX7A2	9606.ENSP00000359098	82
4.4121429993219697E-4	82	SBDS	9606.ENSP00000246868	82
2.696829299080253E-4	82	RBBP4	9606.ENSP00000362592	82
9.513959545136949E-4	82	GOSR1	9606.ENSP00000225724	82
9.394552698247953E-5	82	RRP12	9606.ENSP00000446184	82
8.789067956971758E-4	82	TOMM22	9606.ENSP00000216034	82
5.747417864131335E-4	82	BUB3	9606.ENSP00000357858	82
0.001300869006660227	82	DYNC1H1	9606.ENSP00000348965	82
0.0019610373813402256	82	CASP8	9606.ENSP00000351273	82
1.719723131344224E-4	82	NDUFS6	9606.ENSP00000274137	82
2.961788949204934E-4	82	CMPK1	9606.ENSP00000360939	82

4.069817617547121E-4	82	DAP3	9606.ENSP00000357320	82
0.00137175478087615	81	SLC2A1	9606.ENSP00000416293	81
3.9795046888572516E-4	81	G3BP2	9606.ENSP00000352738	81
8.68954385913476E-4	81	CSTF3	9606.ENSP00000315791	81
2.495350876167129E-4	81	ALDH9A1	9606.ENSP00000346827	81
0.001207724365640818	81	EEA1	9606.ENSP00000317955	81
1.3329770407341764E-4	81	NDUFB5	9606.ENSP00000259037	81
8.464633876074125E-4	81	DNM2	9606.ENSP00000373905	81
4.8669725214074683E-4	81	ACOX1	9606.ENSP00000293217	81
1.8264371643783112E-4	81	HMGCL	9606.ENSP00000363614	81
4.823974147829185E-4	80	NPLOC4	9606.ENSP00000331487	80
3.811937217306925E-4	80	VBP1	9606.ENSP00000286428	80
5.182568050261698E-4	80	PFKL	9606.ENSP00000269848	80
2.8248044696361704E-4	80	CTPS2	9606.ENSP00000401264	80
8.896588678735518E-4	80	RAB1B	9606.ENSP00000310226	80
1.4538421602123766E-4	80	PSMD13	9606.ENSP00000396937	80
4.222135048187894E-4	80	GOT1	9606.ENSP00000359539	80
1.9746256748674623E-4	80	DARS2	9606.ENSP00000497569	80
1.6623097343308396E-4	79	CSTF2	9606.ENSP00000387996	79
1.504897965516244E-4	79	ETFB	9606.ENSP00000346173	79
0.0012232344414099614	79	AP2M1	9606.ENSP00000403362	79
0.0010997476969197703	79	GSN	9606.ENSP00000362924	79
1.3766736660051382E-4	79	PELO	9606.ENSP00000274311	79
0.001053470657962307	79	DYNLL1	9606.ENSP00000376297	79
8.101321737514186E-4	79	COPB2	9606.ENSP00000329419	79
5.880074418751946E-4	79	TOMM70	9606.ENSP00000284320	79
5.595122194538468E-4	79	YWHAQ	9606.ENSP00000371267	79
5.669400735667116E-4	78	PDIA6	9606.ENSP00000385385	78
9.036013112370439E-4	78	ACTR1A	9606.ENSP00000358921	78
2.3226976790004117E-4	78	BCAS2	9606.ENSP00000358554	78
5.361060352935134E-4	78	PFKP	9606.ENSP00000370517	78
5.9449882071001546E-5	78	RALY	9606.ENSP00000246194	78
0.0010611406228445454	78	IGF2R	9606.ENSP00000349437	78
0.0014940194812594588	78	PPP1CB	9606.ENSP00000378769	78
0.0016732146414341516	78	CTSD	9606.ENSP00000236671	78
1.6908423092986538E-4	77	UQCR10	9606.ENSP00000332887	77
0.0026865907507427084	77	VAPA	9606.ENSP00000345656	77
2.5936824528929E-4	77	MRPL46	9606.ENSP00000312311	77
1.0793971309325136E-4	77	SAFB	9606.ENSP00000467423	77
5.287098994558125E-4	77	MAPK14	9606.ENSP00000229795	77
9.163464423877395E-5	77	U2SURP	9606.ENSP00000418563	77
3.49160928286161E-4	77	COX6C	9606.ENSP00000429707	77
1.3237770633531983E-4	77	PRPF31	9606.ENSP00000324122	77
7.228147573375145E-4	77	GRHPR	9606.ENSP00000313432	77
8.993675303799053E-4	77	HGS	9606.ENSP00000331201	77
4.955113077965751E-4	77	UBXN7	9606.ENSP00000296328	77
4.4717906358320386E-5	76	ABCF1	9606.ENSP00000313603	76
6.870013878186876E-5	76	ACIN1	9606.ENSP00000262710	76

3.892593365872951E-4	76	HIBCH	9606.ENSP00000352706	76
2.842638871193677E-4	76	CCDC47	9606.ENSP00000225726	76
5.406057610828948E-4	76	FKBP1A	9606.ENSP00000383003	76
6.311830205593188E-4	76	RTCB	9606.ENSP00000216038	76
2.6332180375318245E-4	76	PRPF4	9606.ENSP00000363313	76
5.343516424302449E-4	76	APEX1	9606.ENSP00000216714	76
6.27141999383417E-4	76	ECI2	9606.ENSP00000369461	76
8.516257706787002E-4	76	OAT	9606.ENSP00000357838	76
6.896923131102818E-4	76	CARS1	9606.ENSP00000369897	76
2.4106808528128016E-4	76	PELP1	9606.ENSP00000301396	76
5.840045142001507E-4	75	AIFM1	9606.ENSP00000287295	75
3.3714681953418295E-4	75	BCAT2	9606.ENSP00000322991	75
3.989785563863329E-4	75	SYMPK	9606.ENSP00000245934	75
2.1614068890552535E-4	75	PSMB5	9606.ENSP00000355325	75
8.721404319970232E-4	75	EPS15	9606.ENSP00000360798	75
4.930981925652478E-4	75	TSTA3	9606.ENSP00000398803	75
2.887824599282311E-4	74	IPO5	9606.ENSP00000261574	74
1.230956454425737E-4	74	WDR75	9606.ENSP00000314193	74
2.1064995692956823E-4	74	CAPRIN1	9606.ENSP00000340329	74
4.936775336645344E-4	74	VAMP8	9606.ENSP00000263864	74
2.1689371996040993E-4	74	AK3	9606.ENSP00000371230	74
2.644316904345006E-4	74	UCHL5	9606.ENSP00000356421	74
0.0010039808848015144	73	ANXA1	9606.ENSP00000366109	73
3.785519710570239E-4	73	APMAP	9606.ENSP00000217456	73
2.7428012693528385E-4	73	SARNP	9606.ENSP00000337632	73
9.793905286293483E-4	73	KLC1	9606.ENSP00000414982	73
5.289657566092028E-4	73	SUPT5H	9606.ENSP00000470252	73
7.522755572251133E-4	73	RHOC	9606.ENSP00000285735	73
5.324853039450377E-4	73	CPSF3	9606.ENSP00000238112	73
1.2182873444238127E-4	73	UTP18	9606.ENSP00000225298	73
7.190223318852287E-4	73	SEC31A	9606.ENSP00000378721	73
3.8114849629134834E-4	73	TTC37	9606.ENSP00000497948	73
5.919756976994721E-4	73	TJP1	9606.ENSP00000348416	73
4.6693206358477295E-4	73	HMGB1	9606.ENSP00000345347	73
2.619766013721213E-4	72	MRPL44	9606.ENSP00000258383	72
4.897509671176062E-4	72	VASP	9606.ENSP00000245932	72
7.167718082114022E-4	72	GOLGB1	9606.ENSP00000377275	72
0.0018055107815246802	72	VAPB	9606.ENSP00000417175	72
1.446557484795625E-4	72	NDUFB6	9606.ENSP00000369176	72
2.8096614848115354E-4	72	SLC25A4	9606.ENSP00000281456	72
2.3320073344905163E-4	72	DNAJC8	9606.ENSP00000263697	72
2.4096952563711004E-4	72	PABPC4	9606.ENSP00000361949	72
3.7418657623644066E-4	72	H2AZ2	9606.ENSP00000308405	72
0.0013649573292359763	71	VPS29	9606.ENSP00000480853	71
5.83682129072792E-4	71	USO1	9606.ENSP00000264904	71
0.0012836150461277014	71	BCAP31	9606.ENSP00000392330	71
7.63354796725192E-4	71	LGALS3	9606.ENSP00000254301	71
7.339621507447703E-4	71	RDX	9606.ENSP00000496414	71

2.720897936315414E-4	71	UCHL3	9606.ENSP00000366819	71
2.596833529067577E-4	71	ETFDH	9606.ENSP00000426638	71
4.7967950687234705E-4	71	CSE1L	9606.ENSP00000262982	71
0.001068309961440453	70	BIN1	9606.ENSP00000316779	70
1.4820041519974703E-4	70	CWC22	9606.ENSP00000387006	70
4.721507635137831E-4	70	UBE2K	9606.ENSP00000261427	70
1.0362265339411082E-4	70	PNN	9606.ENSP00000216832	70
7.806675943774416E-4	70	DBN1	9606.ENSP00000377195	70
6.737724626788862E-4	70	KPNA1	9606.ENSP00000343701	70
8.045201551923799E-4	70	ATP6V1A	9606.ENSP00000273398	70
7.039033649761671E-4	70	HPRT1	9606.ENSP00000298556	70
4.897213626628046E-4	69	STAMBP	9606.ENSP00000377633	69
1.8843833458260297E-4	69	MRPS22	9606.ENSP00000418008	69
1.8685929553274072E-4	69	NOL9	9606.ENSP00000366934	69
8.572950579881703E-4	69	COASY	9606.ENSP00000464814	69
3.388245794690945E-4	69	DHX30	9606.ENSP00000405620	69
7.334495191538477E-4	69	AP2B1	9606.ENSP00000483185	69
1.3538128187048733E-4	69	SRP72	9606.ENSP00000495128	69
4.6317556747184433E-4	69	C1QBP	9606.ENSP00000225698	69
0.0010713099190337106	69	COPG1	9606.ENSP00000325002	69
6.927596210260836E-5	69	TARS1	9606.ENSP00000387710	69
5.935639650752063E-4	69	SCP2	9606.ENSP00000360569	69
1.9122235674632988E-4	69	SNRPN	9606.ENSP00000494831	69
5.556311327022117E-4	69	ILK	9606.ENSP00000379975	69
4.7875421826186856E-4	68	LRPPRC	9606.ENSP00000260665	68
4.481919893945594E-4	68	SMC1A	9606.ENSP00000323421	68
2.757681117340722E-4	68	DNAJA2	9606.ENSP00000314030	68
8.683254649281439E-4	68	GAK	9606.ENSP00000314499	68
2.459710687185686E-4	68	CELF1	9606.ENSP00000436864	68
3.651617504757739E-4	68	PGM2	9606.ENSP00000371393	68
2.949920769624988E-4	68	KPNA4	9606.ENSP00000334373	68
7.111530585063308E-4	68	HSD17B10	9606.ENSP00000168216	68
4.846551702385295E-4	67	FLNB	9606.ENSP00000420213	67
1.0719109390620287E-4	67	LUC7L2	9606.ENSP00000347005	67
6.09203283559953E-4	67	NFKB2	9606.ENSP00000358983	67
0.0021335084306048275	67	CTSB	9606.ENSP00000345672	67
9.925537322080394E-5	67	CHCHD3	9606.ENSP00000389297	67
6.374539812857411E-4	67	FIS1	9606.ENSP00000223136	67
5.994402942688767E-5	67	NGDN	9606.ENSP00000386134	67
9.35188168068555E-5	67	NUP188	9606.ENSP00000361658	67
5.7407091608024E-4	67	COPA	9606.ENSP00000357048	67
2.3818196015446927E-4	67	TIMM23	9606.ENSP00000464522	67
1.8879746981940857E-4	67	PSME1	9606.ENSP00000372155	67
1.3719070230521694E-4	67	MCCC2	9606.ENSP00000343657	67
1.3452474789091344E-4	67	ACADS	9606.ENSP00000242592	67
5.723577554553513E-4	67	SEC24B	9606.ENSP00000428564	67
2.5107707469071194E-4	66	ITCH	9606.ENSP00000499786	66
3.6669260717501665E-4	66	COPS4	9606.ENSP00000424655	66

2.5219494477777735E-4	66 CPSF2	9606.ENSP00000298875	66
3.843161738421547E-4	66 ROCK2	9606.ENSP00000317985	66
0.0013672008040041142	66 SPTAN1	9606.ENSP00000487444	66
4.348818055795855E-4	66 CLTA	9606.ENSP00000242285	66
0.0010731963728549125	66 SEC24C	9606.ENSP00000343405	66
4.745635037143653E-4	66 FKBP4	9606.ENSP00000001008	66
1.6522240006055348E-4	66 PSME2	9606.ENSP00000216802	66
4.271944123749549E-4	66 AIMP2	9606.ENSP00000223029	66
6.241706408618416E-4	66 JUP	9606.ENSP00000377508	66
7.408367499322276E-4	66 GSTO1	9606.ENSP00000358727	66
7.17645538848474E-4	66 DCTN2	9606.ENSP00000408910	66
1.2107768145327821E-4	65 NDUFB11	9606.ENSP00000276062	65
7.603058758927559E-5	65 HNRNPUL1	9606.ENSP00000375863	65
0.0010046015046437234	65 EMD	9606.ENSP00000358857	65
3.0440715435998618E-5	65 PSMD9	9606.ENSP00000440485	65
3.1893714312534267E-4	65 ACADSB	9606.ENSP00000357873	65
3.0424459550660687E-4	65 RCC1	9606.ENSP00000497402	65
6.174762971182828E-4	65 SURF4	9606.ENSP00000361057	65
6.476706430328826E-4	65 COPE	9606.ENSP00000469035	65
4.442665030635798E-4	65 THRAP3	9606.ENSP00000346634	65
6.240138488315408E-4	65 ALDH7A1	9606.ENSP00000387123	65
0.001079311583050271	64 FAF2	9606.ENSP00000261942	64
4.7452185708151513E-4	64 AP2A1	9606.ENSP00000351926	64
7.362607647067193E-4	64 CD9	9606.ENSP00000371958	64
2.882457995951725E-4	64 NSUN2	9606.ENSP00000264670	64
4.633894117152359E-4	64 TNPO3	9606.ENSP00000265388	64
3.8574039223060767E-4	64 AARS1	9606.ENSP00000261772	64
5.082785877067711E-4	64 SND1	9606.ENSP00000346762	64
1.358827068489746E-4	64 ME1	9606.ENSP00000358719	64
6.006462276899674E-4	64 LAMTOR1	9606.ENSP00000278671	64
1.5902702502134828E-4	64 CLPX	9606.ENSP00000300107	64
5.692514157642462E-4	64 ATP6V1F	9606.ENSP00000417378	64
3.090346038223768E-4	63 WARS1	9606.ENSP00000347495	63
3.570069236723432E-4	63 XPOT	9606.ENSP00000327821	63
5.951267530806697E-4	63 GPX4	9606.ENSP00000346103	63
1.4130834724253053E-4	63 FIP1L1	9606.ENSP00000336752	63
1.3979023534399485E-4	63 HINT1	9606.ENSP00000304229	63
3.660980134942208E-4	63 NAPG	9606.ENSP00000324628	63
0.001136150837034315	63 CD81	9606.ENSP00000263645	63
1.242219316269279E-4	63 AHSA1	9606.ENSP00000216479	63
2.1970558623198605E-4	63 FEN1	9606.ENSP00000305480	63
8.273461717480627E-5	63 CPSF7	9606.ENSP00000345412	63
8.357820495246748E-4	63 MYO1C	9606.ENSP00000496954	63
2.4264129283921865E-4	63 PSMD10	9606.ENSP00000217958	63
3.1233802639964734E-4	63 ACSF2	9606.ENSP00000401831	63
2.240049279710888E-4	63 MTHFD1	9606.ENSP00000498336	63
3.473884457062239E-4	63 APRT	9606.ENSP00000367615	63
2.057434068369861E-4	63 ALDH4A1	9606.ENSP00000364490	63

3.5438450733673747E-4	63	SCFD1	9606.ENSP00000390783	63
5.18523896438815E-4	62	ASCC3	9606.ENSP00000358159	62
6.134511057991774E-4	62	GNB1	9606.ENSP00000367872	62
4.401134914075813E-4	62	HK1	9606.ENSP00000494664	62
4.4250851301269985E-4	62	DYNLL2	9606.ENSP00000477310	62
2.807353496061995E-4	62	XPO5	9606.ENSP00000265351	62
3.941054785816267E-4	62	SUPT6H	9606.ENSP00000319104	62
6.161902938295908E-4	62	VTI1B	9606.ENSP00000450731	62
1.0476465980556246E-4	62	NDUFA7	9606.ENSP00000301457	62
4.2364284847823326E-4	62	CRK	9606.ENSP00000300574	62
5.46062455291821E-4	62	PICALM	9606.ENSP00000377015	62
7.283578479051101E-4	62	FKBP8	9606.ENSP00000471700	62
6.657647243445016E-4	62	GLOD4	9606.ENSP00000301329	62
2.2184622943474574E-4	61	SET	9606.ENSP00000361777	61
3.5441058931118426E-4	61	GBF1	9606.ENSP00000359000	61
4.3397488686157935E-4	61	PML	9606.ENSP00000268058	61
5.481126966175653E-4	61	DCTN4	9606.ENSP00000414906	61
4.571431604820778E-4	61	ARHGDIA	9606.ENSP00000463939	61
4.848930401484408E-4	61	RAB5C	9606.ENSP00000447053	61
3.5580660559714744E-5	61	UTP20	9606.ENSP00000261637	61
6.138061108474759E-4	61	RAB10	9606.ENSP00000264710	61
3.707432146724192E-4	61	HK2	9606.ENSP00000290573	61
1.9855761757823123E-4	61	CPT2	9606.ENSP00000360541	61
5.187335876975909E-4	61	ARFGAP1	9606.ENSP00000314615	61
0.0010520388297011348	60	GANAB	9606.ENSP00000340466	60
7.126994089754853E-4	60	ITPR3	9606.ENSP00000363435	60
1.2617647904179373E-4	60	AQR	9606.ENSP00000156471	60
4.297041588498769E-4	60	ARF5	9606.ENSP00000000233	60
3.2355403764020826E-4	60	PPID	9606.ENSP00000303754	60
2.8410692254151953E-4	60	ITGAV	9606.ENSP00000261023	60
4.2328507512000296E-4	60	DYNC1I2	9606.ENSP00000380308	60
1.4866390045250336E-4	60	PCK2	9606.ENSP00000216780	60
4.998108786485196E-4	60	TXNDC5	9606.ENSP00000369081	60
4.094753163935817E-4	60	MAT2A	9606.ENSP00000303147	60
5.66995206198878E-4	60	FECH	9606.ENSP00000498358	60
5.269336449154582E-4	60	PRKAR1A	9606.ENSP00000376475	60
4.030242684084631E-4	59	HSPH1	9606.ENSP00000487365	59
4.92100797814382E-4	59	ISCU	9606.ENSP00000310623	59
1.5587561951949645E-4	59	PDK1	9606.ENSP00000376352	59
2.7126859454835257E-4	59	ARF4	9606.ENSP00000306010	59
3.437753020202436E-4	59	DEK	9606.ENSP00000498653	59
1.8534793691467742E-4	59	HSD17B4	9606.ENSP00000411960	59
1.0782302356678268E-4	59	DDX42	9606.ENSP00000464050	59
3.6795698414622975E-4	59	EXOSC8	9606.ENSP00000374354	59
4.594564827548281E-4	59	SMC3	9606.ENSP00000354720	59
7.583479103876897E-4	58	SDCBP	9606.ENSP00000428184	58
3.7189506621138947E-4	58	SMARCB1	9606.ENSP00000340883	58
4.7339832454652316E-4	58	RHOG	9606.ENSP00000339467	58

8.80231367687869E-4	58 PLEC	9606.ENSP00000323856	58
8.553211548872829E-4	58 MAPRE1	9606.ENSP00000364721	58
5.300392773029534E-4	58 GORASP2	9606.ENSP00000234160	58
9.417560088947923E-4	58 RAB2A	9606.ENSP00000262646	58
3.028257914837883E-4	58 NAMPT	9606.ENSP00000222553	58
3.95187096847971E-4	58 CYFIP1	9606.ENSP00000481038	58
3.683863466944455E-4	58 SP1	9606.ENSP00000329357	58
5.478895803671763E-4	58 HTRA2	9606.ENSP00000258080	58
3.046779197990481E-4	58 SNX9	9606.ENSP00000376024	58
1.4953612314315472E-4	57 BLVRB	9606.ENSP00000263368	57
4.051163557824457E-4	57 SNX1	9606.ENSP00000261889	57
2.61926124795254E-4	57 ARPC2	9606.ENSP00000295685	57
2.3305662341806826E-5	57 NDUFC2	9606.ENSP00000281031	57
4.1237856040335325E-4	57 OTUB1	9606.ENSP00000444357	57
1.0892310911433519E-4	57 HIBADH	9606.ENSP00000265395	57
8.540854571186505E-5	57 CCAR1	9606.ENSP00000265872	57
2.1657851283924835E-4	57 BANF1	9606.ENSP00000433760	57
2.49628025952718E-4	57 YARS2	9606.ENSP00000320658	57
9.055399855993121E-4	57 OS9	9606.ENSP00000318165	57
6.433647332751584E-4	56 USP9X	9606.ENSP00000316357	56
4.5752714556333265E-4	56 NUMA1	9606.ENSP00000377298	56
2.626202924779636E-4	56 GLRX3	9606.ENSP00000357633	56
2.6957805382802496E-4	56 RPA1	9606.ENSP00000254719	56
9.264958461323406E-5	56 OSTC	9606.ENSP00000426167	56
9.321975288124911E-4	56 USP5	9606.ENSP00000229268	56
2.4063101718456E-5	56 ME2	9606.ENSP00000321070	56
8.654775250961719E-4	56 RPN2	9606.ENSP00000237530	56
3.8864093411740354E-4	56 PPP5C	9606.ENSP00000012443	56
1.0314833594914384E-4	56 KPNA6	9606.ENSP00000362728	56
5.29338111143061E-4	56 WDR1	9606.ENSP00000427687	56
0.001387302957733427	55 MECR	9606.ENSP00000263702	55
3.5564315106959406E-4	55 SNX2	9606.ENSP00000368831	55
1.4913387979919636E-4	55 PPAT	9606.ENSP00000264220	55
6.834121154114088E-4	55 DNAJC3	9606.ENSP00000473631	55
2.5356873403218846E-4	55 DYNC1LI1	9606.ENSP00000273130	55
4.06404093495233E-4	55 AP3B1	9606.ENSP00000255194	55
3.541797075945861E-4	55 TMED9	9606.ENSP00000330945	55
3.64774977845441E-4	55 TPM1	9606.ENSP00000351022	55
2.430803905176255E-5	55 PSMD5	9606.ENSP00000210313	55
3.657130415978955E-4	55 FLOT1	9606.ENSP00000365569	55
2.0489810763607932E-4	55 RBM3	9606.ENSP00000365950	55
1.044437789559648E-4	55 SART3	9606.ENSP00000449386	55
1.8600588401148998E-4	55 TIMM44	9606.ENSP00000270538	55
1.2402113596188567E-4	55 WASF2	9606.ENSP00000483313	55
2.0965855750360932E-4	55 TRMT112	9606.ENSP00000438349	55
4.374121222068198E-4	54 ZW10	9606.ENSP00000200135	54
8.140295465049053E-5	54 NDUFV3	9606.ENSP00000346196	54
5.793261352895051E-4	54 DNAJC5	9606.ENSP00000354111	54

5.170690497151523E-4	54	CTBP1	9606.ENSP00000290921	54
0.0014236145698121674	54	GBA	9606.ENSP00000314508	54
7.05324607891665E-4	54	TMED3	9606.ENSP00000299705	54
3.9827098633618774E-4	54	ACSL4	9606.ENSP00000339787	54
2.625461641682678E-4	54	AARS2	9606.ENSP00000244571	54
5.364565952802419E-4	54	COG4	9606.ENSP00000315775	54
3.2934867355922443E-4	54	ADK	9606.ENSP00000443965	54
2.595062444562676E-4	54	EML4	9606.ENSP00000320663	54
9.782496698928684E-5	54	PPIL1	9606.ENSP00000362803	54
2.796332054700779E-4	54	ARHGEF7	9606.ENSP00000495631	54
3.226294638943851E-4	54	UBA3	9606.ENSP00000354340	54
6.766515199277825E-4	54	CTSZ	9606.ENSP00000217131	54
3.1784864497870854E-4	54	MCM4	9606.ENSP00000262105	54
4.088363444293553E-4	53	PYGB	9606.ENSP00000216962	53
2.740896402063519E-4	53	ABI1	9606.ENSP00000365312	53
4.473005144703056E-4	53	GNB2	9606.ENSP00000305260	53
1.7789767608556408E-5	53	SREK1	9606.ENSP00000334538	53
4.1885902138791785E-4	53	MCCC1	9606.ENSP00000265594	53
2.843570874607956E-4	53	ARPC3	9606.ENSP00000228825	53
6.5857375997001E-4	53	PAFAH1B1	9606.ENSP00000380378	53
3.039333189538641E-4	53	AP2A2	9606.ENSP00000327694	53
1.3754492205981256E-4	53	SUMO3	9606.ENSP00000409666	53
7.751666258348784E-4	53	DNAJC11	9606.ENSP00000366800	53
3.8300672805877557E-4	53	FLOT2	9606.ENSP00000378368	53
3.0396699530460855E-5	53	PCCA	9606.ENSP00000365462	53
2.3395744912352697E-4	53	CAD	9606.ENSP00000264705	53
3.2670132684787216E-4	53	H1-0	9606.ENSP00000344504	53
3.638249433309471E-4	53	PPM1G	9606.ENSP00000342778	53
2.35533545370805E-4	53	MAP2K2	9606.ENSP00000262948	53
5.415275788467105E-4	53	SSR1	9606.ENSP00000244763	53
2.4254798707903973E-4	53	ATP6V1B2	9606.ENSP00000276390	53
5.27008406006459E-4	52	HIP1R	9606.ENSP00000253083	52
2.4916408118022575E-4	52	LMNB2	9606.ENSP00000327054	52
2.513387023776288E-4	52	YWHAH	9606.ENSP00000248975	52
1.5441139668176087E-4	52	UPF2	9606.ENSP00000348708	52
4.0066982055699825E-4	52	RRBP1	9606.ENSP00000367038	52
2.0885126247785848E-4	52	SARS1	9606.ENSP00000358939	52
8.672521212988958E-4	52	SEC23A	9606.ENSP00000306881	52
2.57932782631026E-4	52	MYL6	9606.ENSP00000446955	52
4.448427334938928E-5	52	LUC7L	9606.ENSP00000293872	52
4.418782800533399E-4	52	RAB14	9606.ENSP00000362946	52
1.593422462266299E-4	52	PSIP1	9606.ENSP00000370114	52
2.478332692129689E-4	52	AP1G1	9606.ENSP00000377148	52
2.838530838331802E-4	52	SEL1L	9606.ENSP00000337053	52
4.2609837858167I4E-4	52	VPS26A	9606.ENSP00000263559	52
3.8005028674310945E-4	52	GFPT1	9606.ENSP00000349860	52
2.460898186022757E-4	52	DYNC1LI2	9606.ENSP00000258198	52
2.763598165845217E-4	52	GIT1	9606.ENSP00000378338	52

4.1881546200615236E-4	52	NFS1	9606.ENSP00000363205	52
1.3823821061522242E-4	52	TIMM50	9606.ENSP00000445806	52
1.941501545779833E-4	52	TXNL1	9606.ENSP00000217515	52
2.111737814116346E-4	52	MSH2	9606.ENSP00000233146	52
1.5942682449522259E-4	52	ECHDC1	9606.ENSP00000436585	52
3.383574611844125E-4	52	TWF1	9606.ENSP00000449428	52
2.739769265523025E-4	52	STOML2	9606.ENSP00000348886	52
3.599097472370099E-4	51	NAE1	9606.ENSP00000351990	51
1.3127526503681035E-4	51	SMARCC1	9606.ENSP00000254480	51
2.2614037072308675E-4	51	SGTA	9606.ENSP00000221566	51
5.642926691698203E-4	51	MOGS	9606.ENSP00000410992	51
2.1787049630434042E-4	51	CTNNA1	9606.ENSP00000304669	51
0.0012201648627104052	51	PNP	9606.ENSP00000354532	51
1.261408367941921E-4	51	CSDE1	9606.ENSP00000481762	51
6.547312256766204E-4	51	PPP1R12A	9606.ENSP00000389168	51
2.20510903785382E-4	51	UBA2	9606.ENSP00000246548	51
7.049134093681174E-4	51	STRN	9606.ENSP00000263918	51
4.1137127498592726E-4	51	ACSL3	9606.ENSP00000350012	51
5.093945179549058E-4	51	ITPA	9606.ENSP00000369456	51
1.8224958653320376E-4	51	CSK	9606.ENSP00000220003	51
1.8325757615844223E-4	51	FDXR	9606.ENSP00000416515	51
4.0119993706068787E-4	51	BSG	9606.ENSP00000333769	51
2.2864106586345778E-4	50	NAP1L1	9606.ENSP00000477538	50
1.855130109677362E-4	50	DDX19A	9606.ENSP00000306117	50
7.173014601465294E-5	50	NUTF2	9606.ENSP00000219169	50
5.204091417249198E-6	50	EIF2D	9606.ENSP00000271764	50
5.8147556864967886E-5	50	ZNF326	9606.ENSP00000340796	50
2.2739492092153425E-4	50	AP1B1	9606.ENSP00000350199	50
2.517279846647495E-4	50	ECH1	9606.ENSP00000221418	50
7.66230991207704E-5	50	CBX3	9606.ENSP00000336687	50
2.913343336798155E-4	50	ARHGAP1	9606.ENSP00000310491	50
3.7330162709009907E-4	50	ATP6V1E1	9606.ENSP00000253413	50
3.2804450931056344E-4	50	COG7	9606.ENSP00000305442	50
1.494315797108681E-4	50	SLC25A11	9606.ENSP00000225665	50
1.0678250364209852E-4	50	PPIL2	9606.ENSP00000486725	50
3.03923977881645E-4	50	PGM3	9606.ENSP00000425809	50
2.489643289515765E-4	50	COPS3	9606.ENSP00000268717	50
1.0928840431334135E-4	49	LETM1	9606.ENSP00000305653	49
1.321867521634555E-4	49	ARFGAP2	9606.ENSP00000434442	49
4.275655873299901E-5	49	IVD	9606.ENSP00000499074	49
4.320595132641177E-4	49	GDI2	9606.ENSP00000369538	49
1.362775514707704E-4	49	ARFGAP3	9606.ENSP00000263245	49
2.163930529231218E-4	49	COPZ1	9606.ENSP00000449270	49
7.174065237532382E-4	49	ATP2A2	9606.ENSP00000440045	49
9.980431324920871E-5	49	KPNA3	9606.ENSP00000261667	49
1.7928450847710058E-4	49	MSH6	9606.ENSP00000234420	49
3.461137165348326E-4	49	COG2	9606.ENSP00000355629	49
2.726856506922561E-4	49	COG1	9606.ENSP00000299886	49

6.809620933894962E-4	48	ADD3	9606.ENSP00000348381	48
3.5847376110943724E-4	48	ITGB4	9606.ENSP00000200181	48
3.082375146915604E-4	48	AKR1B1	9606.ENSP00000285930	48
1.6505468637435406E-4	48	PREB	9606.ENSP00000260643	48
1.200293680506656E-4	48	CBX5	9606.ENSP00000209875	48
2.561614293612708E-4	48	SNX5	9606.ENSP00000366998	48
2.4061183441532596E-4	48	RPS6KA1	9606.ENSP00000435412	48
1.5213131713761902E-4	48	GPD2	9606.ENSP00000308610	48
1.8113670063993171E-4	48	COPS2	9606.ENSP00000299259	48
6.177218265014887E-6	48	EIF2A	9606.ENSP00000417229	48
3.9085441737710905E-4	48	NEDD4	9606.ENSP00000424827	48
2.3546236397519195E-4	48	TFG	9606.ENSP00000240851	48
1.430379770660233E-4	48	NARS1	9606.ENSP00000256854	48
3.093142354270001E-4	48	UBAP2L	9606.ENSP00000389445	48
2.9529678053931093E-4	48	SURF1	9606.ENSP00000361042	48
2.710052283221643E-4	48	STAM	9606.ENSP00000366746	48
2.855412248299239E-4	47	LMAN2	9606.ENSP00000303366	47
3.524437502300382E-4	47	SEC23B	9606.ENSP00000338844	47
3.8155650884586797E-4	47	CRKL	9606.ENSP00000346300	47
4.057374530016217E-4	47	GUK1	9606.ENSP00000355689	47
6.474089528802912E-4	47	DPYSL2	9606.ENSP00000427985	47
2.6503180079004337E-4	47	GNAQ	9606.ENSP00000286548	47
1.7232123698434693E-4	47	DECR1	9606.ENSP00000220764	47
4.1749040752444845E-5	46	OXCT1	9606.ENSP00000196371	46
3.3282355791899973E-4	46	NELFCD	9606.ENSP00000473290	46
1.6574017113748517E-4	46	PRDX5	9606.ENSP00000265462	46
5.603848889630222E-4	46	GSTP1	9606.ENSP00000381607	46
2.4136024205492594E-4	46	SCRIB	9606.ENSP00000349486	46
2.356398515709574E-4	46	UBA6	9606.ENSP00000313454	46
2.0737243465736158E-4	46	AP1M1	9606.ENSP00000388996	46
1.9530208595123594E-4	46	STX7	9606.ENSP00000356918	46
3.062730361945481E-4	46	CLINT1	9606.ENSP00000429824	46
2.467873351452161E-4	46	NCKAP1	9606.ENSP00000354251	46
2.2037801252296752E-4	46	GCLC	9606.ENSP00000497574	46
1.648292488496431E-4	45	UGGT1	9606.ENSP00000259253	45
1.4872935559732508E-4	45	SDF2L1	9606.ENSP00000248958	45
1.4859073367098503E-4	45	PDS5A	9606.ENSP00000303427	45
2.789736617282681E-4	45	EXOC7	9606.ENSP00000334100	45
1.1829532328107367E-4	45	TIMM13	9606.ENSP00000215570	45
7.733725817910255E-5	45	NUFIP2	9606.ENSP00000225388	45
6.983296834377202E-4	45	CTBP2	9606.ENSP00000311825	45
4.0230444460326575E-4	45	BCKDK	9606.ENSP00000378405	45
1.1109019719069714E-4	45	ALDOC	9606.ENSP00000378731	45
4.0400318394534534E-4	45	STX4	9606.ENSP00000317714	45
2.2635282746226218E-4	45	COPG2	9606.ENSP00000402346	45
1.7592558017694805E-4	45	CPT1A	9606.ENSP00000265641	45
3.061821274622989E-4	44	SPTBN1	9606.ENSP00000349259	44
2.8155918690234963E-4	44	RAB18	9606.ENSP00000478479	44

1.8630878045092992E-4	44	SLIRP	9606.ENSP00000450909	44
1.9442352471956307E-4	44	GOLGA5	9606.ENSP00000163416	44
9.604208231887291E-4	44	PRKAG1	9606.ENSP00000323867	44
2.4109007496973963E-4	44	ARPC1B	9606.ENSP00000389631	44
4.536338012825589E-4	44	HDLBP	9606.ENSP00000375836	44
2.3715693064418338E-4	44	ATP5MD	9606.ENSP00000358840	44
1.555849673382044E-4	44	MCM6	9606.ENSP00000264156	44
9.624852933872264E-5	44	AP1S1	9606.ENSP00000336666	44
1.380110828863805E-4	44	ARHGEF2	9606.ENSP00000354837	44
3.0582389121149153E-4	44	SLC25A1	9606.ENSP00000215882	44
6.613773709036136E-5	44	GTF2F1	9606.ENSP00000377969	44
2.383084741705144E-4	44	TAGLN2	9606.ENSP00000357076	44
2.6110323035627263E-4	44	AFDN	9606.ENSP00000355771	44
4.158315076825159E-4	44	EIF2B3	9606.ENSP00000353575	44
5.473520288706819E-4	44	UBE2L3	9606.ENSP00000400906	44
2.729942034910709E-4	44	GPS1	9606.ENSP00000376167	44
1.889737497439522E-4	44	RALA	9606.ENSP00000005257	44
1.3828101200547824E-4	44	RAP1B	9606.ENSP00000250559	44
1.8993797715499957E-4	44	ACSL5	9606.ENSP00000348429	44
2.9273409853724373E-5	44	ALDH6A1	9606.ENSP00000450436	44
4.7909862085431956E-5	44	NME3	9606.ENSP00000219302	44
2.1885447945900406E-4	44	DERA	9606.ENSP00000416583	44
2.111386489518268E-4	43	DAZAP1	9606.ENSP00000233078	43
2.8447868482530915E-4	43	SNX4	9606.ENSP00000251775	43
3.3711688418237075E-4	43	STMN1	9606.ENSP00000410452	43
1.5183904838023942E-4	43	SUB1	9606.ENSP00000265073	43
3.318793218000152E-4	43	MYO6	9606.ENSP00000358994	43
2.003185604310572E-4	43	UBE4B	9606.ENSP00000343001	43
2.557492425357119E-4	43	LGALS4	9606.ENSP00000302100	43
2.979300408669517E-4	43	EXOC2	9606.ENSP00000230449	43
2.964452271819747E-4	43	SNX27	9606.ENSP00000400333	43
4.113937344750798E-4	43	AKAP9	9606.ENSP00000348573	43
2.9217953955813965E-4	43	TJP2	9606.ENSP00000438262	43
2.6151071544890304E-4	43	CAND1	9606.ENSP00000442318	43
2.108615063796165E-4	43	KLC4	9606.ENSP00000259708	43
1.3571893415753977E-4	43	SCO1	9606.ENSP00000255390	43
6.907143927683907E-5	43	SEC24A	9606.ENSP00000381823	43
1.3095503779537247E-4	43	PUS7	9606.ENSP00000417402	43
1.4691443248083256E-4	43	M6PR	9606.ENSP00000000412	43
2.3310444922621757E-4	42	XRCC1	9606.ENSP00000262887	42
1.5587104780074025E-4	42	API5	9606.ENSP00000431391	42
5.288735787504178E-4	42	PLIN3	9606.ENSP00000221957	42
6.707762776712188E-4	42	IDE	9606.ENSP00000265986	42
2.033443120141894E-4	42	MRPS31	9606.ENSP00000315397	42
3.0506588087864106E-4	42	EDC3	9606.ENSP00000497737	42
1.8996446600758347E-4	42	CD47	9606.ENSP00000355361	42
2.961555957988314E-4	42	EXOC4	9606.ENSP00000253861	42
3.089068553117236E-5	42	BAG2	9606.ENSP00000359727	42

1.7163288064965816E-4	42	SAE1	9606.ENSP00000270225	42
6.179062226928646E-5	42	MACROH2A1	9606.ENSP00000423563	42
9.057807141701403E-5	42	ASL	9606.ENSP00000307188	42
2.9281524404191133E-4	42	TRIP10	9606.ENSP00000320117	42
2.669752590993669E-4	42	EMC2	9606.ENSP00000220853	42
1.952011311798253E-4	42	MRE11	9606.ENSP00000325863	42
1.277118850562963E-4	42	MIF	9606.ENSP00000215754	42
1.2675504458987486E-4	42	USP15	9606.ENSP00000280377	42
3.646794241224905E-4	42	GNAS	9606.ENSP00000360141	42
2.052671113508377E-4	42	SAR1B	9606.ENSP00000385432	42
6.182629847896205E-4	42	ADD1	9606.ENSP00000264758	42
6.863015928201076E-5	42	UBTF	9606.ENSP00000302640	42
1.823371119396965E-4	42	ZYX	9606.ENSP00000324422	42
1.1187402856386103E-4	41	RNF20	9606.ENSP00000373772	41
2.1649555085041965E-4	41	SERPINH1	9606.ENSP00000434412	41
2.1742528857475265E-4	41	PLAA	9606.ENSP00000380460	41
2.3854738453821066E-4	41	ARPC5	9606.ENSP00000294742	41
1.5218924736870194E-4	41	SARS2	9606.ENSP00000472847	41
3.904491011162007E-4	41	GRSF1	9606.ENSP00000254799	41
2.745112298813842E-4	41	EPS8	9606.ENSP00000494689	41
2.729550120621706E-4	41	EDC4	9606.ENSP00000351811	41
2.8785536769034665E-4	41	GDI1	9606.ENSP00000394071	41
0.0010712740117687857	41	CALU	9606.ENSP00000438248	41
1.1263442674266996E-4	41	RBM15	9606.ENSP00000358799	41
9.519898836277666E-5	41	BRD2	9606.ENSP00000378702	41
2.980674807926759E-4	41	LMO7	9606.ENSP00000366757	41
9.921029674323932E-5	41	GLS	9606.ENSP00000317379	41
8.948012346643206E-5	41	GCSH	9606.ENSP00000319531	41
2.754040380942037E-4	40	HSD17B12	9606.ENSP00000278353	40
3.9269932659029516E-4	40	GNG12	9606.ENSP00000360021	40
2.2757119567278275E-4	40	ILVBL	9606.ENSP00000263383	40
2.933473508444653E-4	40	ATP6V0D1	9606.ENSP00000290949	40
2.4455431691295667E-4	40	ATOX1	9606.ENSP00000430598	40
3.282856838617755E-4	40	SEC23IP	9606.ENSP00000358071	40
5.271310430534173E-4	40	GNA13	9606.ENSP00000400717	40
3.724592980701681E-4	40	PLIN2	9606.ENSP00000276914	40
7.769800437599321E-5	40	CAT	9606.ENSP00000241052	40
1.7385476081893772E-4	40	FKBP5	9606.ENSP00000444810	40
2.7331475744920734E-4	40	NSFL1C	9606.ENSP00000418529	40
3.9040861148641626E-4	40	NIPSNAP2	9606.ENSP00000313050	40
2.661403138934014E-4	40	MTCH2	9606.ENSP00000303222	40
2.0762377628201487E-4	40	MTHFD1L	9606.ENSP00000478253	40
1.824587595637053E-4	40	ITGA6	9606.ENSP00000386896	40
7.094206377404946E-5	40	COQ9	9606.ENSP00000262507	40
3.0062282082306757E-4	40	RAB5B	9606.ENSP00000353444	40
1.7997863021989712E-4	40	ERLEC1	9606.ENSP00000185150	40
3.6991645948697443E-4	40	CCAR2	9606.ENSP00000310670	40
4.4878088483121586E-4	40	EMC1	9606.ENSP00000420608	40

2.122550525414495E-4	40	BAX	9606.ENSP00000293288	40
6.755940219556191E-4	40	GALE	9606.ENSP00000483375	40
2.1333265598103018E-4	39	SEC62	9606.ENSP00000337688	39
1.8660002152163515E-4	39	COPS7A	9606.ENSP00000446039	39
1.4642163716525533E-4	39	HCFC1	9606.ENSP00000309555	39
1.188047164479117E-4	39	EPS15L1	9606.ENSP00000393313	39
3.4984187098334365E-4	39	CTSC	9606.ENSP00000227266	39
3.057031967929325E-5	39	HNRNPLL	9606.ENSP00000390625	39
6.716805663963685E-5	39	PURA	9606.ENSP00000332706	39
4.970397096479092E-5	39	EEF1E1	9606.ENSP00000369038	39
8.687910404865621E-5	39	PRPSAP2	9606.ENSP00000268835	39
5.569234024225176E-4	39	DPM1	9606.ENSP00000360638	39
2.5972711385697177E-4	39	SSR4	9606.ENSP00000317331	39
4.338067236075723E-5	38	CISD2	9606.ENSP00000273986	38
7.827274269229032E-5	38	MCM2	9606.ENSP00000265056	38
1.9261284817218734E-4	38	EXOC3	9606.ENSP00000425587	38
6.31826087975062E-5	38	MRPS27	9606.ENSP00000426941	38
4.5786646518540563E-4	38	CD2AP	9606.ENSP00000352264	38
7.00134349771954E-5	38	CTNND1	9606.ENSP00000382004	38
1.8517710922202416E-4	38	PRPSAP1	9606.ENSP00000414624	38
2.7624369979550113E-4	38	OGT	9606.ENSP00000362824	38
1.8321925764339938E-4	38	APOO	9606.ENSP00000368528	38
6.127676458164269E-4	38	MTAP	9606.ENSP00000494373	38
6.192516285024127E-4	38	RTN4	9606.ENSP00000337838	38
2.0163733644945421E-4	38	TSN	9606.ENSP00000374332	38
1.8852738016916917E-4	37	MMUT	9606.ENSP00000274813	37
1.6832808538402928E-4	37	MTX2	9606.ENSP00000249442	37
2.1998985730231477E-4	37	NBAS	9606.ENSP00000281513	37
1.6874757178255237E-4	37	EXOSC6	9606.ENSP00000398597	37
3.2855961415842553E-4	37	PDCD6	9606.ENSP00000264933	37
1.1133636187934475E-4	37	ITGA2	9606.ENSP00000296585	37
2.1333533334164315E-5	37	SLC25A24	9606.ENSP00000457733	37
7.76121484925694E-4	37	CTSA	9606.ENSP00000361562	37
1.8500769919603904E-4	37	CORO1C	9606.ENSP00000394496	37
3.862602060891768E-4	37	ERP29	9606.ENSP00000261735	37
2.193637438927213E-4	37	MAOA	9606.ENSP00000340684	37
2.3030386875653755E-4	37	PPP3CA	9606.ENSP00000378323	37
2.2264446179284138E-4	37	BAIAP2L1	9606.ENSP00000005260	37
8.161138272775382E-5	37	NELFE	9606.ENSP00000364578	37
4.93156279654257E-5	37	L2HGDH	9606.ENSP00000267436	37
1.5501397806500446E-4	37	PPIL3	9606.ENSP00000286175	37
2.4345395793004437E-4	37	GIGYF2	9606.ENSP00000387170	37
2.3786702196312867E-4	36	ARHGEF12	9606.ENSP00000380942	36
8.729180254714095E-5	36	ERO1A	9606.ENSP00000379042	36
4.57930959072509E-4	36	STIM1	9606.ENSP00000478059	36
1.7707411528946113E-4	36	ERP44	9606.ENSP00000262455	36
6.626375235952372E-5	36	RPA3	9606.ENSP00000223129	36
2.125987874602131E-4	36	MICOS13	9606.ENSP00000468723	36

1.6684429912944778E-4	36	ATP6V1D	9606.ENSP00000216442	36
1.2792895189780532E-4	36	BAZ1B	9606.ENSP00000342434	36
1.6759472334188446E-4	36	GCLM	9606.ENSP00000359258	36
2.1217097102602545E-4	36	ADAM10	9606.ENSP00000260408	36
8.750185736816331E-5	36	DOHH	9606.ENSP00000398882	36
2.693154695543292E-4	36	ACP1	9606.ENSP00000272067	36
1.614695839516677E-4	36	CBFB	9606.ENSP00000415151	36
1.382796371892977E-4	36	AGFG1	9606.ENSP00000387282	36
1.5047995950895874E-4	36	DIDO1	9606.ENSP00000266070	36
6.704671534979011E-5	36	SLC25A13	9606.ENSP00000400101	36
2.757435917550765E-4	36	TCIRG1	9606.ENSP00000265686	36
1.7637261099776746E-4	36	ANP32A	9606.ENSP00000417864	36
2.109204540498062E-4	35	SH3GL1	9606.ENSP00000269886	35
0.0014031810912343635	35	RHOT1	9606.ENSP00000351132	35
1.600582762543694E-4	35	STX12	9606.ENSP00000363054	35
2.8643148810014436E-4	35	EIF2B1	9606.ENSP00000416250	35
1.8092079504564824E-4	35	PRKCSH	9606.ENSP00000466134	35
1.3823076010372373E-4	35	UBE4A	9606.ENSP00000387362	35
3.3198195830918994E-4	35	VCPIP1	9606.ENSP00000309031	35
2.4612208067282995E-4	35	RAB35	9606.ENSP00000229340	35
9.883563005894322E-5	35	SLC9A3R1	9606.ENSP00000262613	35
4.4757544574939873E-4	35	AHNAK	9606.ENSP00000367263	35
5.429266000914535E-5	35	SMARCC2	9606.ENSP00000449396	35
5.54170074397599E-5	35	PTCD3	9606.ENSP00000254630	35
3.850273447670647E-5	35	SON	9606.ENSP00000348984	35
1.1600466094203161E-4	35	GOLGA4	9606.ENSP00000349305	35
1.3204199691221335E-4	35	DPY30	9606.ENSP00000345837	35
3.1579541235059834E-4	35	GLYR1	9606.ENSP00000322716	35
1.3611574612202315E-4	35	VPS53	9606.ENSP00000401435	35
1.6217815259095554E-4	35	IPO9	9606.ENSP00000354742	35
1.0403297658073582E-4	35	TRRAP	9606.ENSP00000352925	35
1.560844629100978E-4	34	LAMTOR2	9606.ENSP00000357288	34
1.381064899051422E-4	34	PSPC1	9606.ENSP00000343966	34
2.3882357192836403E-4	34	EXOC1	9606.ENSP00000370695	34
2.3498626620280897E-4	34	VPS28	9606.ENSP00000366565	34
4.545863743216903E-4	34	AGK	9606.ENSP00000497280	34
1.7365014732358727E-4	34	RAB3GAP1	9606.ENSP00000411418	34
5.655045170546396E-5	34	NASP	9606.ENSP00000255120	34
3.083541788466301E-4	34	DBNL	9606.ENSP00000417653	34
9.545289018326713E-5	34	RRAGC	9606.ENSP00000362092	34
3.418650431959104E-4	34	CAPN1	9606.ENSP00000431984	34
3.8113962724977645E-5	34	SDF2	9606.ENSP00000247020	34
1.3269208890173326E-4	34	RER1	9606.ENSP00000475168	34
1.6356939580682034E-4	34	LRP1	9606.ENSP00000243077	34
9.47038266866847E-5	34	ATP6V1H	9606.ENSP00000352522	34
2.031765102280669E-4	34	AUP1	9606.ENSP00000366748	34
2.4172468190436126E-5	34	TNPO2	9606.ENSP00000407182	34
1.163887923825989E-4	34	GET3	9606.ENSP00000466379	34

1.9248886602160915E-4	34	SLC3A2	9606.ENSP00000367123	34
0.0012705041247186779	34	ERLIN2	9606.ENSP00000428112	34
2.947032068598417E-4	34	CTSL	9606.ENSP00000345344	34
2.0054667373029265E-4	33	PEX5	9606.ENSP00000391601	33
2.5058979652536634E-4	33	CHMP4B	9606.ENSP00000217402	33
2.2670051980824677E-4	33	RRAS	9606.ENSP00000246792	33
1.3784141366262383E-4	33	NAA15	9606.ENSP00000296543	33
3.378649615572924E-4	33	HM13	9606.ENSP00000381237	33
2.860572950180969E-4	33	ALG5	9606.ENSP00000239891	33
2.7421568682601817E-4	33	TXNDC12	9606.ENSP00000360688	33
5.762812655211709E-5	33	STRAP	9606.ENSP00000392270	33
2.2627195810087644E-4	33	PPP2R2A	9606.ENSP00000325074	33
2.9237044264718753E-4	33	P4HA1	9606.ENSP00000263556	33
6.722853076513024E-5	33	H1-2	9606.ENSP00000339566	33
2.5752556699086196E-4	33	CCDC22	9606.ENSP00000365401	33
9.342964017144044E-5	33	KIF2A	9606.ENSP00000385000	33
1.9336678100150776E-4	33	DUT	9606.ENSP00000370376	33
7.502058611781078E-5	33	SAR1A	9606.ENSP00000362339	33
3.158934157676077E-4	33	ARFGEF2	9606.ENSP00000360985	33
7.689096804184837E-5	32	OPA1	9606.ENSP00000354681	32
8.161334897995383E-5	32	TRAPPC3	9606.ENSP00000480332	32
1.5197790971304337E-4	32	SRM	9606.ENSP00000366156	32
3.859817484834416E-5	32	AHCTF1	9606.ENSP00000355465	32
5.279062298668991E-5	32	FCHO2	9606.ENSP00000393776	32
1.5120461052011658E-4	32	PRKAA1	9606.ENSP00000346148	32
1.0067334111324936E-4	32	USP19	9606.ENSP00000401197	32
2.3364329798715886E-4	32	ATP6V0A1	9606.ENSP00000264649	32
1.279299623104408E-4	32	DAD1	9606.ENSP00000250498	32
1.414333194375438E-4	32	AP3M1	9606.ENSP00000347408	32
3.4246756647539903E-4	32	CYB5A	9606.ENSP00000341625	32
6.508115654747799E-4	32	ESYT1	9606.ENSP00000267113	32
2.6639099667912766E-4	32	SUN2	9606.ENSP00000385616	32
1.7680668642252359E-4	32	MAN2A1	9606.ENSP00000261483	32
1.186902920305411E-4	32	GNA11	9606.ENSP00000078429	32
4.5819521798748077E-4	32	LAP3	9606.ENSP00000226299	32
1.8507021053447458E-4	32	PLS3	9606.ENSP00000348163	32
3.000972204458806E-4	32	VTA1	9606.ENSP00000356602	32
8.56626195202554E-5	32	EIF2B2	9606.ENSP00000266126	32
8.640410773513134E-4	32	ELP1	9606.ENSP00000363779	32
1.927504926199749E-4	32	GNAI2	9606.ENSP00000312999	32
2.125013757694415E-4	32	GOLIM4	9606.ENSP00000417354	32
1.0301210503499663E-4	32	DSTN	9606.ENSP00000246069	32
1.1771382128912783E-4	32	UGDH	9606.ENSP00000319501	32
5.2722058065469E-5	32	FUBP3	9606.ENSP00000318177	32
8.885745804171276E-5	32	TST	9606.ENSP00000385828	32
1.2476264463440033E-4	32	LAMTOR3	9606.ENSP00000424183	32
1.3036262107699568E-4	31	ARHGEF1	9606.ENSP00000337261	31
2.903669955746738E-4	31	VPS51	9606.ENSP00000279281	31

9.995570367992962E-5	31	PFDN4	9606.ENSP00000360473	31
1.9788062219192902E-4	31	ATAD1	9606.ENSP00000339017	31
1.1490910308687344E-4	31	KIF3B	9606.ENSP00000364864	31
7.69416391560415E-5	31	PBRM1	9606.ENSP00000386593	31
2.3581752735524908E-4	31	STXBP2	9606.ENSP00000413606	31
1.6850793133745134E-4	31	COMT	9606.ENSP00000354511	31
4.47070340790896E-5	31	DARS1	9606.ENSP00000264161	31
4.7731022224767696E-4	31	HSPG2	9606.ENSP00000363827	31
5.0557423099921015E-5	31	NUBPL	9606.ENSP00000281081	31
3.0320517270844436E-4	31	DAG1	9606.ENSP00000442600	31
5.9322382007846504E-5	31	ADH5	9606.ENSP00000296412	31
1.7788239881901495E-4	31	ELAC2	9606.ENSP00000337445	31
1.8500808996067334E-4	31	DBI	9606.ENSP00000486361	31
1.265681355703241E-4	31	LRRC59	9606.ENSP00000225972	31
3.6727656163676583E-4	31	CUX1	9606.ENSP00000353401	31
3.1714467499483655E-4	31	ZMPSTE24	9606.ENSP00000361845	31
2.670768985420537E-5	31	ST13	9606.ENSP00000216218	31
8.085864560509484E-4	31	SPTLC1	9606.ENSP00000262554	31
1.0461704232835337E-4	30	ASH2L	9606.ENSP00000340896	30
3.3580394218046404E-5	30	PDCD4	9606.ENSP00000280154	30
3.8307003181988714E-5	30	MCU	9606.ENSP00000362144	30
2.240669391846456E-4	30	GAPVD1	9606.ENSP00000377665	30
4.506925992191564E-5	30	CKB	9606.ENSP00000299198	30
2.74124658373124E-4	30	CBR1	9606.ENSP00000290349	30
1.794476840365431E-4	30	FKBP2	9606.ENSP00000378046	30
6.18894984654593E-5	30	SLC25A12	9606.ENSP00000388658	30
9.206950831660441E-5	30	TPD52	9606.ENSP00000429309	30
2.0294894576132467E-4	30	SNX17	9606.ENSP00000233575	30
1.8755400849357505E-4	30	DLG1	9606.ENSP00000345731	30
4.343004512937444E-5	30	ATP6V1G1	9606.ENSP00000363162	30
2.141674174459694E-5	30	YES1	9606.ENSP00000462468	30
7.130916330274477E-5	30	CAMK2G	9606.ENSP00000319060	30
9.048162596567461E-4	30	PPT1	9606.ENSP00000493153	30
9.787871819881628E-5	29	UBR4	9606.ENSP00000364403	29
4.854929446816575E-5	29	ZFR	9606.ENSP00000265069	29
1.1754025618140324E-4	29	CISD1	9606.ENSP00000363041	29
7.134603523037556E-5	29	GALM	9606.ENSP00000272252	29
4.0223927810983733E-4	29	NNT	9606.ENSP00000264663	29
8.391705606472065E-5	29	CRYZ	9606.ENSP00000399805	29
1.3007118373753446E-4	29	RAB3GAP2	9606.ENSP00000351832	29
1.3653423836884572E-4	29	AGRN	9606.ENSP00000368678	29
1.5409160722107496E-4	29	PRKAB1	9606.ENSP00000441369	29
1.0939510292925661E-4	29	MANF	9606.ENSP00000499582	29
8.027772332542807E-5	29	ACTR1B	9606.ENSP00000289228	29
1.407533786558422E-4	29	CHMP6	9606.ENSP00000317468	29
4.98310706839081E-5	29	SUGT1	9606.ENSP00000367208	29
1.6008329821979385E-4	29	PITRM1	9606.ENSP00000370377	29
2.427211979322112E-4	29	S100A10	9606.ENSP00000357801	29

8.915898604313641E-5	29	SNX3	9606.ENSP00000230085	29
1.9590602268631496E-4	29	AKR1B10	9606.ENSP00000352584	29
7.515951246831909E-5	29	EXOC8	9606.ENSP00000355605	29
1.5201959581616733E-4	29	VPS4B	9606.ENSP00000238497	29
1.2089410765407811E-4	29	SNX6	9606.ENSP00000498872	29
1.0262794642338468E-4	28	APOOL	9606.ENSP00000362268	28
4.581573773092609E-5	28	PGLS	9606.ENSP00000252603	28
0.0014709290694117428	28	SGPL1	9606.ENSP00000362298	28
9.18674172679016E-5	28	MARK2	9606.ENSP00000385751	28
1.394366361638651E-4	28	NIT2	9606.ENSP00000377696	28
5.5736797867786986E-5	28	RALB	9606.ENSP00000272519	28
7.607627736087419E-5	28	LGALS1	9606.ENSP00000215909	28
1.0946928181444944E-4	28	MYO5C	9606.ENSP00000261839	28
1.3346706794659135E-4	28	TRIP11	9606.ENSP00000267622	28
7.628165528969203E-5	28	AGPS	9606.ENSP00000264167	28
2.4394055282411727E-4	28	DSP	9606.ENSP00000369129	28
1.0465734579755705E-4	28	TNKS1BP1	9606.ENSP00000437271	28
2.9042599621227416E-4	28	DDRGK1	9606.ENSP00000346483	28
1.0012963543189997E-4	28	XPO7	9606.ENSP00000252512	28
6.282711739813749E-5	28	ALDH16A1	9606.ENSP00000293350	28
1.663239451667309E-4	28	CKAP4	9606.ENSP00000367265	28
1.3088447774531293E-4	28	EHD1	9606.ENSP00000479153	28
5.546927623245612E-5	28	SQOR	9606.ENSP00000456019	28
1.3018198060536738E-4	28	UTRN	9606.ENSP00000356515	28
2.553593128469963E-4	28	ARFGEF1	9606.ENSP00000262215	28
1.6101562531575323E-4	28	USP47	9606.ENSP00000382382	28
9.749656803949179E-5	28	PDLIM7	9606.ENSP00000348099	28
7.651014967217621E-5	28	CBR4	9606.ENSP00000303525	28
1.8248815462992295E-4	28	RMDN3	9606.ENSP00000260385	28
1.386454434492991E-4	27	MYO18A	9606.ENSP00000437073	27
1.3246551058294074E-4	27	TCOF1	9606.ENSP00000367028	27
1.7823644341989644E-4	27	MTA2	9606.ENSP00000278823	27
1.6971583942567317E-4	27	NCSTN	9606.ENSP00000294785	27
1.6161083638330256E-4	27	MAP2K3	9606.ENSP00000345083	27
1.5760611421906823E-4	27	ABCD3	9606.ENSP00000359233	27
1.590185440202655E-4	27	DNAJC13	9606.ENSP00000260818	27
4.870625298762988E-5	27	ATP6V1C1	9606.ENSP00000430282	27
1.5714899264855384E-4	27	ATP1A1	9606.ENSP00000445306	27
8.105367579221952E-5	27	CNP	9606.ENSP00000377470	27
1.1131677192429478E-4	27	ARL1	9606.ENSP00000261636	27
1.9634327739151565E-5	27	TBC1D8B	9606.ENSP00000349781	27
1.690000171829098E-4	27	GALNT2	9606.ENSP00000355632	27
7.911919300737374E-5	27	CAMK2D	9606.ENSP00000425824	27
2.0944217508652317E-4	27	ARL3	9606.ENSP00000260746	27
2.0283909854923633E-4	27	MIA3	9606.ENSP00000340900	27
8.546497559105839E-5	26	PRKCI	9606.ENSP00000295797	26
2.130882858238736E-4	26	ESD	9606.ENSP00000367992	26
5.861920427687705E-5	26	NELFB	9606.ENSP00000339495	26

1.712324278381282E-4	26	ANXA11	9606.ENSP00000398610	26
4.323502449957203E-5	26	STXBP3	9606.ENSP00000359025	26
7.95962790792495E-5	26	ERGIC2	9606.ENSP00000353270	26
9.598680293528194E-6	26	CHCHD6	9606.ENSP00000290913	26
5.0227542328686264E-5	26	NEK9	9606.ENSP00000238616	26
6.751303195811518E-5	26	HDGF	9606.ENSP00000357189	26
7.837707674361897E-5	26	ETHE1	9606.ENSP00000292147	26
2.8295915132194713E-5	26	PTBP3	9606.ENSP00000414921	26
8.684065953224733E-5	26	BAG6	9606.ENSP00000365131	26
9.861466094711815E-5	26	AKR1A1	9606.ENSP00000361140	26
2.3100207606380517E-4	26	CLYBL	9606.ENSP00000365533	26
9.933061121349578E-5	26	GALK1	9606.ENSP00000225614	26
3.1047904922338064E-4	26	CORO1B	9606.ENSP00000340211	26
1.613121462239141E-5	26	RAD50	9606.ENSP00000368100	26
3.471326773896637E-5	26	CDC42BPB	9606.ENSP00000355237	26
6.367176767925524E-5	26	CTDP1	9606.ENSP00000484525	26
2.959672807814351E-4	26	ALG2	9606.ENSP00000417764	26
8.952162739764948E-5	26	OSGEP	9606.ENSP00000206542	26
6.303525660672459E-5	26	MAN1A1	9606.ENSP00000357453	26
1.4912530591348728E-4	26	POR	9606.ENSP00000419970	26
1.937963288541903E-4	26	PMM2	9606.ENSP00000268261	26
2.265008994471753E-5	26	UBXN1	9606.ENSP00000294119	26
7.047851652956178E-5	26	FAHD1	9606.ENSP00000372112	26
2.0901269298029726E-4	26	IGBP1	9606.ENSP00000363661	26
1.8038687501668825E-4	26	PYCR1	9606.ENSP00000384949	26
8.87170876675812E-5	26	HAGH	9606.ENSP00000380514	26
1.0805281083208737E-5	26	TOMM6	9606.ENSP00000381859	26
1.1884812804685517E-4	25	MPST	9606.ENSP00000411719	25
6.4463318680310035E-6	25	CRAT	9606.ENSP00000315013	25
1.3734173627905598E-4	25	ACOT13	9606.ENSP00000230048	25
4.87138016181654E-4	25	LNPEP	9606.ENSP00000231368	25
3.602525316734716E-4	25	GLB1	9606.ENSP00000306920	25
7.376889988730366E-5	25	MFF	9606.ENSP00000302037	25
1.513247135711263E-4	25	GIPC1	9606.ENSP00000376753	25
5.1846337211370214E-5	25	H1-4	9606.ENSP00000307705	25
1.7333954131119946E-5	25	MRPS36	9606.ENSP00000256441	25
1.0062654315247705E-4	25	IPO11	9606.ENSP00000386992	25
1.772572050281771E-4	25	RAB11B	9606.ENSP00000333547	25
5.611606778218942E-4	25	CAPNS1	9606.ENSP00000464849	25
2.516986456340001E-4	25	ANXA7	9606.ENSP00000362010	25
8.19433304678046E-5	25	AP3D1	9606.ENSP00000495274	25
2.2874078766268438E-4	25	GPD1L	9606.ENSP00000282541	25
8.216211065114815E-5	25	MMGT1	9606.ENSP00000306220	25
2.018169935627289E-4	25	PLS1	9606.ENSP00000336831	25
2.4780202823381295E-4	25	TPP1	9606.ENSP00000299427	25
6.663636485328333E-5	25	PKN2	9606.ENSP00000359552	25
7.558201137257496E-5	24	LAMC1	9606.ENSP00000258341	24
3.4910662797126306E-4	24	IMPA1	9606.ENSP00000408526	24

8.401118762128756E-5	24	SMS	9606.ENSP00000385746	24
5.53857993343404E-5	24	PHPT1	9606.ENSP00000247665	24
2.49295110054224E-4	24	CYP51A1	9606.ENSP00000003100	24
6.97888406721722E-5	24	PTPN23	9606.ENSP00000265562	24
5.5116879663108427E-5	24	TRADD	9606.ENSP00000341268	24
6.365372849733698E-5	24	AMPD2	9606.ENSP00000499465	24
6.252542970985623E-5	24	HMGA2	9606.ENSP00000437621	24
8.741787419051856E-5	24	RCN2	9606.ENSP00000319739	24
9.018483489841228E-5	24	GNAI3	9606.ENSP00000358867	24
1.615097015938517E-4	24	EPHX1	9606.ENSP00000480004	24
1.272160463035129E-4	24	GET4	9606.ENSP00000265857	24
2.1269440825940356E-5	24	PARVA	9606.ENSP00000334008	24
2.9654598861594426E-5	24	IQGAP2	9606.ENSP00000274364	24
2.9306928899411803E-5	24	AAK1	9606.ENSP00000386456	24
2.5754894707165205E-5	24	EPHB4	9606.ENSP00000350896	24
3.4400956248046834E-5	24	LIMA1	9606.ENSP00000378400	24
1.636164023696369E-4	24	UFM1	9606.ENSP00000368970	24
6.27633814663819E-5	24	AKAP13	9606.ENSP00000354718	24
7.529613065637869E-5	24	PLCB3	9606.ENSP00000443631	24
4.483746929457902E-5	24	PRKRA	9606.ENSP00000318176	24
4.839334786259093E-5	24	CAP1	9606.ENSP00000361883	24
1.0963941690704079E-4	24	MVB12A	9606.ENSP00000324810	24
3.288326321864783E-5	24	COMMD9	9606.ENSP00000263401	24
7.380490005644019E-5	24	MTX1	9606.ENSP00000357360	24
6.682813531487154E-5	24	SYNE2	9606.ENSP00000350719	24
2.4343340601021353E-4	24	CAPN2	9606.ENSP00000295006	24
2.940094568297058E-4	23	NCEH1	9606.ENSP00000442464	23
8.983566643647694E-5	23	TMOD3	9606.ENSP00000308753	23
1.622180022825022E-4	23	RTN3	9606.ENSP00000367050	23
4.439664698889271E-5	23	ALCAM	9606.ENSP00000305988	23
8.614481864668638E-5	23	S100A11	9606.ENSP00000271638	23
1.179127178462442E-4	23	PEX14	9606.ENSP00000349016	23
1.7295779790182643E-4	23	TMSB4X	9606.ENSP00000370010	23
1.3176069422635853E-4	23	ANKFY1	9606.ENSP00000459943	23
4.422356847466746E-5	23	NDRG1	9606.ENSP00000404854	23
3.555291282027994E-5	23	SMARCD2	9606.ENSP00000392617	23
8.289140667987558E-5	23	VPS25	9606.ENSP00000253794	23
3.914867512887519E-5	23	CNOT7	9606.ENSP00000355279	23
3.0375702224871906E-6	23	SLTM	9606.ENSP00000369887	23
1.5303910881544875E-4	23	MRPL28	9606.ENSP00000497004	23
5.164833301956469E-5	23	NT5C	9606.ENSP00000245552	23
7.05357590663833E-5	23	ARL8B	9606.ENSP00000479202	23
8.701942878407594E-5	23	DYNLT1	9606.ENSP00000356056	23
9.370232350238538E-5	23	TBC1D15	9606.ENSP00000448182	23
5.687245590512064E-5	22	VPS37B	9606.ENSP00000267202	22
8.187633841210979E-5	22	TOR1AIP1	9606.ENSP00000435365	22
7.910359764041019E-5	22	PGAM5	9606.ENSP00000438465	22
2.6184953962046925E-6	22	NECAP2	9606.ENSP00000391942	22

1.246758216939334E-4	22	AGL	9606.ENSP00000294724	22
0.001121123533367977	22	RIC8A	9606.ENSP00000325941	22
2.0440496096549215E-4	22	PACSIN2	9606.ENSP00000263246	22
1.2521503595295014E-4	22	TGM2	9606.ENSP00000355330	22
4.046025213372625E-5	22	MICU2	9606.ENSP00000371811	22
9.820059626825155E-5	22	GNS	9606.ENSP00000258145	22
1.8298548932124404E-4	22	FDPS	9606.ENSP00000349078	22
2.308589631910034E-5	22	INTS3	9606.ENSP00000318641	22
1.1350255131036227E-4	22	ABCC1	9606.ENSP00000382342	22
2.240194207392651E-5	22	RAVER1	9606.ENSP00000482486	22
4.3665820012556395E-5	22	MPRIP	9606.ENSP00000379156	22
7.846065204416328E-5	22	GALNT1	9606.ENSP00000269195	22
1.3258055123186848E-4	22	IST1	9606.ENSP00000438399	22
1.4420259020455164E-4	21	STK24	9606.ENSP00000365730	21
7.387801898459249E-5	21	UBXN4	9606.ENSP00000272638	21
6.647772665051041E-5	21	FKBP9	9606.ENSP00000439250	21
1.151648345463255E-4	21	TBRG4	9606.ENSP00000258770	21
4.482069067784229E-5	21	NT5C2	9606.ENSP00000339479	21
2.3360746924267906E-5	21	GATAD2B	9606.ENSP00000357644	21
3.383986687753581E-5	21	CNOT9	9606.ENSP00000486540	21
3.298713064786949E-5	21	VPS33A	9606.ENSP00000267199	21
3.5238940420830697E-5	21	TRIOBP	9606.ENSP00000496394	21
2.9127368150468956E-5	21	HMGB2	9606.ENSP00000296503	21
5.957246545846855E-5	21	PCID2	9606.ENSP00000479494	21
1.2421528060318612E-4	21	GTF2I	9606.ENSP00000460070	21
2.144602248272818E-5	21	ANP32E	9606.ENSP00000463154	21
1.5230547363630846E-4	21	PGRMC2	9606.ENSP00000481886	21
5.055638712889911E-5	21	KIF13B	9606.ENSP00000427900	21
2.1693624874171233E-5	21	PPIL4	9606.ENSP00000253329	21
9.09796612536072E-6	21	CTNNA2	9606.ENSP00000384638	21
6.930305447964202E-6	21	TRAM1	9606.ENSP00000262213	21
7.347513982089028E-5	21	SRI	9606.ENSP00000265729	21
9.117909905886588E-5	21	MYO1B	9606.ENSP00000376132	21
6.889468229253903E-5	21	QDPR	9606.ENSP00000281243	21
1.3605492434953304E-4	21	AKR7A2	9606.ENSP00000235835	21
4.9436976766817174E-5	21	ARPC5L	9606.ENSP00000345361	21
5.585423244604092E-5	20	LASP1	9606.ENSP00000325240	20
7.597248732224595E-5	20	DYNLRB1	9606.ENSP00000349679	20
3.553658627555491E-4	20	CERT1	9606.ENSP00000383996	20
5.183010812446593E-5	20	RAB8B	9606.ENSP00000312734	20
1.3723353242366782E-4	20	LPCAT3	9606.ENSP00000261407	20
2.1416789198115356E-5	20	ACOT2	9606.ENSP00000238651	20
6.666442217958382E-5	20	CCS	9606.ENSP00000436318	20
0.0010661396442099842	20	ADPGK	9606.ENSP00000312250	20
7.424932678264775E-5	20	PDIA5	9606.ENSP00000323313	20
2.433900521499043E-5	20	NAP1L4	9606.ENSP00000369915	20
1.2970298521968615E-4	20	PDXK	9606.ENSP00000291565	20
1.092955297835832E-4	20	GOLGA3	9606.ENSP00000204726	20

2.458409923501173E-5	20	MSI2	9606.ENSP00000284073	20
1.8077279490647522E-4	20	LRPAP1	9606.ENSP00000497444	20
3.3227122673310904E-4	20	RCC2	9606.ENSP00000364585	20
4.438169533949532E-5	20	FAM120A	9606.ENSP00000277165	20
4.2690339094572515E-6	20	MRPL37	9606.ENSP00000473980	20
6.447090928201577E-5	20	FABP5	9606.ENSP00000297258	20
1.8214637079135357E-4	20	MAP1S	9606.ENSP00000325313	20
9.324567411633447E-5	20	TMEM43	9606.ENSP00000303992	20
4.522018301202757E-5	20	RAB21	9606.ENSP00000261263	20
2.877536069075024E-5	19	EMC3	9606.ENSP00000245046	19
8.62475142931179E-6	19	MPC2	9606.ENSP00000271373	19
1.818389132053498E-5	19	MLEC	9606.ENSP00000228506	19
1.3824139558863059E-4	19	ASAH1	9606.ENSP00000371152	19
5.590404691358715E-5	19	UROD	9606.ENSP00000246337	19
5.0634954640612054E-5	19	PRKAR2A	9606.ENSP00000265563	19
9.527503257228E-5	19	TMX1	9606.ENSP00000393316	19
6.536453400886197E-5	19	WASHC4	9606.ENSP00000484713	19
7.555638123432082E-5	19	LGALS3BP	9606.ENSP00000262776	19
4.801263265284295E-5	19	BID	9606.ENSP00000318822	19
2.6451502156222954E-4	19	PREP	9606.ENSP00000499089	19
3.608447418536036E-5	19	H1-5	9606.ENSP00000330074	19
1.4879320638536988E-5	19	CHORDC1	9606.ENSP00000319255	19
6.676338040112325E-5	19	CYB5R3	9606.ENSP00000354468	19
5.724615352492231E-6	19	SAFB2	9606.ENSP00000252542	19
2.733106408826123E-5	19	LAMA5	9606.ENSP00000252999	19
1.7189824698156384E-4	19	CNDP2	9606.ENSP00000325548	19
2.1814300878767466E-5	19	MAN1A2	9606.ENSP00000348959	19
2.0253672355567814E-4	19	DOCK7	9606.ENSP00000251157	19
8.207210923515751E-5	19	NPC2	9606.ENSP00000451206	19
7.196293149302272E-5	19	CCDC6	9606.ENSP00000263102	19
2.6119856957058112E-5	19	PTPA	9606.ENSP00000377036	19
7.597798704042477E-5	19	SLC7A5	9606.ENSP00000261622	19
1.2658489784934013E-4	19	REEP5	9606.ENSP00000368959	19
7.402519201066076E-5	19	LGMN	9606.ENSP00000376911	19
2.027139143249503E-5	19	VPS37C	9606.ENSP00000301765	19
7.968711833013682E-5	19	ATL3	9606.ENSP00000381844	19
2.8621928098159556E-5	18	LAMB1	9606.ENSP00000222399	18
8.517729146510788E-5	18	FKBP15	9606.ENSP00000416158	18
1.0373000841306863E-5	18	ZC3H4	9606.ENSP00000253048	18
2.3028303678962623E-5	18	MARCKS	9606.ENSP00000478061	18
4.039362914360142E-5	18	UACA	9606.ENSP00000314556	18
6.133382045771834E-5	18	MMS19	9606.ENSP00000359818	18
1.904009066999223E-5	18	SPAG9	9606.ENSP00000262013	18
1.4201002410470231E-4	18	NAXE	9606.ENSP00000357218	18
7.463843189214035E-5	18	GGH	9606.ENSP00000260118	18
4.703449409545592E-6	18	TAOK3	9606.ENSP00000376317	18
9.858437724446985E-5	18	GTPBP1	9606.ENSP00000216044	18
9.6260649522321E-5	18	ATP1B3	9606.ENSP00000286371	18

1.4565137765210136E-4	18	ACBD3	9606.ENSP00000355777	18
1.733032933098627E-4	18	APEH	9606.ENSP00000296456	18
5.1492210478602735E-5	18	YLPM1	9606.ENSP00000324463	18
3.8619355521998936E-5	18	PTRH2	9606.ENSP00000387180	18
4.133707040221983E-5	17	BABAM1	9606.ENSP00000352408	17
8.174743621327774E-5	17	CTSH	9606.ENSP00000220166	17
4.614589467942735E-5	17	UBE2H	9606.ENSP00000347836	17
1.443233351721939E-4	17	WNK1	9606.ENSP00000341292	17
2.2579084475660982E-5	17	MYO1E	9606.ENSP00000288235	17
1.0015779023927812E-4	17	NANS	9606.ENSP00000210444	17
1.9349341594824016E-4	17	ABHD11	9606.ENSP00000222800	17
6.726760624402357E-6	17	ACOT9	9606.ENSP00000368605	17
2.2328723922438437E-5	17	MAPRE2	9606.ENSP00000300249	17
6.429927724163934E-5	17	S100A13	9606.ENSP00000392767	17
3.295631253820507E-4	17	VAC14	9606.ENSP00000261776	17
3.7058306066784683E-6	17	DCAF8	9606.ENSP00000357052	17
2.1003548352209948E-5	17	FAM114A2	9606.ENSP00000341597	17
1.5304496343404605E-4	17	SFXN1	9606.ENSP00000316905	17
7.4418058080861335E-6	17	SLK	9606.ENSP00000358770	17
1.7464430433273108E-4	17	ERAP1	9606.ENSP00000296754	17
1.7263979757395095E-5	17	MTDH	9606.ENSP00000338235	17
5.650164386200893E-5	17	PPP1R9B	9606.ENSP00000478767	17
1.0105099049200977E-5	17	RO60	9606.ENSP00000356416	17
5.0387429044448566E-5	17	SCYL1	9606.ENSP00000270176	17
3.245735230557614E-5	17	TRMT1L	9606.ENSP00000356476	17
4.864079476169884E-5	16	ATP2B1	9606.ENSP00000392043	16
6.595165716352662E-5	16	ATP1B1	9606.ENSP00000356790	16
8.845278414663604E-5	16	TECR	9606.ENSP00000215567	16
4.931265406183022E-5	16	LRRFIP1	9606.ENSP00000375857	16
1.0386818233115132E-5	16	CGN	9606.ENSP00000271636	16
3.4896097425397034E-5	16	PDLIM1	9606.ENSP00000360305	16
2.0944713434663407E-6	16	FARP1	9606.ENSP00000486285	16
2.557033722106144E-4	16	RNF213	9606.ENSP00000464087	16
1.9612745899848774E-5	16	FERMT1	9606.ENSP00000217289	16
3.193959444787557E-5	16	AKAP8	9606.ENSP00000269701	16
1.2860035797825886E-4	16	THOP1	9606.ENSP00000304467	16
4.288753390762609E-4	16	TMED4	9606.ENSP00000404042	16
6.0876299500812475E-5	16	PGRMC1	9606.ENSP00000217971	16
7.156749884197859E-5	16	UFL1	9606.ENSP00000358283	16
1.4076438471954127E-5	16	ANP32B	9606.ENSP00000345848	16
1.3225953683246323E-5	16	MYH14	9606.ENSP00000493594	16
1.8802795755683377E-5	16	GSS	9606.ENSP00000495750	16
3.804261986183092E-5	16	MGST1	9606.ENSP00000379512	16
1.3876965211384674E-4	15	HMOX2	9606.ENSP00000477572	15
1.3569178624955959E-4	15	TMEM214	9606.ENSP00000238788	15
4.058593143438394E-5	15	COTL1	9606.ENSP00000262428	15
2.0855175628309096E-4	15	NIPSNAP1	9606.ENSP00000216121	15
1.632118182080406E-5	15	ZMYND8	9606.ENSP00000439800	15

2.9258154763271233E-5	15	GSTZ1	9606.ENSP00000451976	15
3.921472267280843E-5	15	CYB5R1	9606.ENSP00000356218	15
5.839901614541582E-5	15	SH3GLB1	9606.ENSP00000479919	15
6.760435527816355E-6	15	OTUD6B	9606.ENSP00000285420	15
2.1556099947438612E-5	15	HP1BP3	9606.ENSP00000403039	15
4.2190121779799455E-5	15	GAN	9606.ENSP00000497351	15
7.394239571243984E-5	15	PPM1A	9606.ENSP00000327255	15
8.954470128030539E-5	15	SCAMP1	9606.ENSP00000481022	15
1.1431206413454814E-6	15	EPS8L2	9606.ENSP00000435585	15
4.3242444089315855E-5	15	FLII	9606.ENSP00000324573	15
1.4526018678174365E-5	15	SLC16A1	9606.ENSP00000441065	15
3.537715296606389E-5	15	VPS13C	9606.ENSP00000493560	15
5.651562039488698E-5	15	BZW1	9606.ENSP00000394316	15
1.6958386371377705E-5	14	HOOK3	9606.ENSP00000305699	14
3.404624189628661E-5	14	BLVRA	9606.ENSP00000385757	14
1.1855172046683353E-5	14	CARMIL1	9606.ENSP00000331983	14
2.3937105416525692E-5	14	PDLIM5	9606.ENSP00000480359	14
8.995705601206865E-5	14	OSBPL8	9606.ENSP00000261183	14
2.1645194645615554E-5	14	SELENBP1	9606.ENSP00000397261	14
2.6389402933570367E-5	14	MRI1	9606.ENSP00000040663	14
1.4602559718506232E-5	14	RPRD1B	9606.ENSP00000362532	14
1.063435431675109E-4	14	AIP	9606.ENSP00000279146	14
5.607646821729768E-5	14	TBCD	9606.ENSP00000347719	14
1.0404415199206212E-4	14	PLPBP	9606.ENSP00000333551	14
6.103260454328388E-5	14	CSRP1	9606.ENSP00000356275	14
1.167314577256554E-4	14	SPTLC2	9606.ENSP00000216484	14
1.1034881737211693E-5	14	USP24	9606.ENSP00000294383	14
1.465445391165711E-4	14	AGPAT1	9606.ENSP00000378877	14
2.3854911908050122E-5	14	CSTB	9606.ENSP00000291568	14
1.061703774204741E-4	14	ATP2C1	9606.ENSP00000421326	14
5.372374323932049E-5	14	MYOF	9606.ENSP00000352208	14
2.8942038223186733E-5	14	LPP	9606.ENSP00000491657	14
2.5697795522072004E-4	14	DPP3	9606.ENSP00000440502	14
6.280128352285267E-5	14	DPP7	9606.ENSP00000360635	14
6.89281582102661E-4	14	ZFPL1	9606.ENSP00000294258	14
4.58707133109935E-5	14	TOR1B	9606.ENSP00000259339	14
4.7028511419869945E-5	14	CAPG	9606.ENSP00000263867	14
4.905613647619845E-5	14	SPR	9606.ENSP00000234454	14
3.5616538790098324E-5	14	TMEM14C	9606.ENSP00000444561	14
1.9875887569102675E-5	14	MAP4	9606.ENSP00000353375	14
2.7586198540206894E-4	14	PCYT1A	9606.ENSP00000292823	14
5.3800283131922536E-5	14	TMEM165	9606.ENSP00000370736	14
3.586535619645577E-5	14	NUDT5	9606.ENSP00000419628	14
4.3202661805094366E-5	14	PTGES2	9606.ENSP00000345341	14
7.157913025288607E-5	13	GM2A	9606.ENSP00000349687	13
3.2611584772270805E-5	13	NID1	9606.ENSP00000264187	13
1.2402572686857726E-4	13	XPNPEP1	9606.ENSP00000421566	13
4.0519809437024734E-5	13	TUBGCP3	9606.ENSP00000261965	13

1.274801599201138E-4	13	PCYT2	9606.ENSP00000442050	13
1.1338730802436867E-4	13	NPEPPS	9606.ENSP00000320324	13
5.5050189636539493E-5	13	SEPHS1	9606.ENSP00000367893	13
2.8323317787972518E-5	13	TUBGCP2	9606.ENSP00000446093	13
1.2521379553487883E-4	13	CERS2	9606.ENSP00000271688	13
9.326137343823066E-5	13	HEXB	9606.ENSP00000261416	13
5.8497521467537424E-5	13	PLOD1	9606.ENSP00000196061	13
6.769746935177817E-5	13	ATP13A1	9606.ENSP00000349877	13
7.634525160743935E-5	13	NAGA	9606.ENSP00000379680	13
5.176207799586291E-5	13	SLMAP	9606.ENSP00000499241	13
9.584404559408788E-6	13	CLIC1	9606.ENSP00000364935	13
3.536833131847673E-5	13	NUDT2	9606.ENSP00000368452	13
2.4961910917707987E-5	12	PRRC1	9606.ENSP00000421965	12
3.5838810893945766E-5	12	MTPN	9606.ENSP00000376800	12
5.195384401165712E-6	12	FMNL2	9606.ENSP00000288670	12
4.540378179275361E-5	12	TPP2	9606.ENSP00000365220	12
3.9196458537670076E-5	12	SLC12A2	9606.ENSP00000262461	12
5.965619042958551E-5	12	IAH1	9606.ENSP00000417580	12
8.810298956846776E-6	12	ARHGAP17	9606.ENSP00000289968	12
2.6369669867887394E-5	12	BCL2L13	9606.ENSP00000480836	12
3.6113367354756756E-5	12	PPP4R1	9606.ENSP00000383402	12
7.4385632877158205E-6	12	TES	9606.ENSP00000350937	12
2.0949288728290417E-4	12	NUCB1	9606.ENSP00000385923	12
1.8360893129790408E-5	12	FKBP11	9606.ENSP00000449751	12
3.0570490600312816E-6	12	TNS3	9606.ENSP00000312143	12
1.3837560140728675E-5	12	DENND4C	9606.ENSP00000473469	12
4.991834259265879E-5	12	PPL	9606.ENSP00000340510	12
5.880982058053567E-5	12	PITPNA	9606.ENSP00000316809	12
4.289244649760182E-5	11	GAA	9606.ENSP00000305692	11
1.167310466828744E-4	11	PIP4K2C	9606.ENSP00000347032	11
2.130881185921837E-5	11	XPNPEP3	9606.ENSP00000349658	11
1.1292032849378048E-5	11	ADI1	9606.ENSP00000333666	11
1.1489718742944996E-5	11	ASPH	9606.ENSP00000368767	11
1.2213234684122615E-4	11	PIGK	9606.ENSP00000359848	11
1.5043250804654561E-5	11	ATXN10	9606.ENSP00000252934	11
3.052635020330513E-5	11	HACD3	9606.ENSP00000261875	11
4.204675682750133E-5	11	NAGK	9606.ENSP00000477639	11
1.6774854096244524E-5	11	CTSE	9606.ENSP00000350911	11
4.016863647967566E-6	11	H1-3	9606.ENSP00000244534	11
9.970680669573327E-5	11	SCAMP3	9606.ENSP00000307275	11
1.5125391273678224E-4	11	TMX3	9606.ENSP00000299608	11
3.5583671102715054E-5	11	LIN7C	9606.ENSP00000278193	11
9.8372860034308E-6	11	ANXA4	9606.ENSP00000377833	11
4.20853419631245E-5	11	PPP1R7	9606.ENSP00000234038	11
7.437580125251647E-6	11	MVP	9606.ENSP00000378760	11
4.160399974773396E-5	11	MYDGF	9606.ENSP00000262947	11
1.2669110951006424E-5	11	ENDOD1	9606.ENSP00000278505	11
2.4232786822951273E-5	11	TXNDC17	9606.ENSP00000250101	11

1.26229086027105E-5	10	PTGFRN	9606.ENSP00000376899	10
1.0733298820877616E-5	10	PPFIA1	9606.ENSP00000253925	10
7.08779668501048E-5	10	CPNE1	9606.ENSP00000317257	10
3.91896262897907E-5	10	TIPRL	9606.ENSP00000356807	10
1.8108247340558036E-4	10	GLG1	9606.ENSP00000205061	10
4.3048373087102484E-5	10	NAGLU	9606.ENSP00000225927	10
2.6151941345847003E-5	10	ANXA3	9606.ENSP00000264908	10
2.332488025346232E-5	10	SCAMP2	9606.ENSP00000268099	10
5.440704631607376E-5	10	SELENOI	9606.ENSP00000260585	10
1.24425285764701E-5	10	HSD17B11	9606.ENSP00000351035	10
6.329843422997667E-5	10	ESYT2	9606.ENSP00000499020	10
1.2331944558402027E-5	10	PRUNE1	9606.ENSP00000271620	10
3.497005424665545E-5	10	AHNAK2	9606.ENSP00000353114	10
3.479671289342601E-5	10	ARFIP1	9606.ENSP00000296557	10
1.6319640717199403E-5	10	CNIH4	9606.ENSP00000420443	10
3.104760629311783E-5	10	CLCC1	9606.ENSP00000349456	10
3.863733670799859E-5	10	OXSR1	9606.ENSP00000311713	10
2.6342786251406578E-5	10	LNPK	9606.ENSP00000440905	10
4.6862389531679664E-5	10	SCRN2	9606.ENSP00000290216	10
5.5590892132257515E-5	10	B4GALT1	9606.ENSP00000369055	10
7.379314152210811E-6	10	BROX	9606.ENSP00000343742	10
6.967610791991915E-6	10	OCIAD1	9606.ENSP00000370882	10
5.840225641877952E-5	10	PCYOX1	9606.ENSP00000387654	10
1.2284579474865302E-4	10	LYPLA1	9606.ENSP00000320043	10
1.3377750670964843E-4	10	PAFAH1B2	9606.ENSP00000435289	10
1.8848856097533877E-5	10	ERGIC1	9606.ENSP00000377374	10
3.787329125693162E-5	9	CDK5RAP3	9606.ENSP00000438886	9
1.981176282077897E-5	9	RNPEP	9606.ENSP00000295640	9
3.488371160460935E-5	9	CMAS	9606.ENSP00000229329	9
2.556953936857412E-5	9	TBCB	9606.ENSP00000221855	9
1.0636731856047687E-4	9	LTA4H	9606.ENSP00000228740	9
1.056522756345033E-6	9	PDS5B	9606.ENSP00000313851	9
2.2001030627368536E-5	9	NHLRC2	9606.ENSP00000358307	9
1.6928464240353676E-6	9	TKFC	9606.ENSP00000378360	9
2.889835270135036E-5	9	KDSR	9606.ENSP00000494352	9
3.0843288171778984E-5	9	PITPNB	9606.ENSP00000487693	9
2.6094980246865556E-6	9	ARHGEF16	9606.ENSP00000367629	9
3.964188615131786E-5	9	TM9SF2	9606.ENSP00000493515	9
5.6708241226557616E-5	9	TAX1BP3	9606.ENSP00000225525	9
9.68458745250145E-6	9	PRXL2A	9606.ENSP00000482445	9
1.2573309534332197E-5	9	NAXD	9606.ENSP00000311984	9
2.0832063235989107E-5	9	ARL6IP5	9606.ENSP00000273258	9
5.15828890493154E-5	8	SETD3	9606.ENSP00000327436	8
1.7265341345813765E-5	8	OSBPL3	9606.ENSP00000315410	8
6.372943833412178E-7	8	CENPV	9606.ENSP00000299736	8
3.460199862135739E-5	8	CUTA	9606.ENSP00000499422	8
1.655336822241917E-4	8	CARHSP1	9606.ENSP00000379838	8
2.1115154376359207E-5	8	ZC3HC1	9606.ENSP00000351052	8

5.534692640794973E-6	8 SH3BGRL3	9606.ENSP00000270792	8
9.878052788007748E-6	8 GRIPAP1	9606.ENSP00000365606	8
2.279553366393492E-5	8 TBL2	9606.ENSP00000307260	8
1.2364183518459964E-4	8 FAM98B	9606.ENSP00000380734	8
1.0581740767816083E-5	8 VWA8	9606.ENSP00000368612	8
9.600805117830609E-5	8 ABHD14B	9606.ENSP00000420065	8
7.527671227125862E-7	8 RBM47	9606.ENSP00000295971	8
1.0122574946079909E-5	8 ARHGEF40	9606.ENSP00000298694	8
2.4728062007708173E-5	8 STK3	9606.ENSP00000429744	8
2.1056774212940797E-4	8 MYO1D	9606.ENSP00000324527	8
4.3072605133908975E-5	8 TBCE	9606.ENSP00000439170	8
3.95221427812886E-5	8 NLN	9606.ENSP00000370372	8
6.358156814850852E-5	7 RNH1	9606.ENSP00000433999	7
7.411538674983737E-6	7 HINT2	9606.ENSP00000259667	7
5.709466712999425E-6	7 EBP	9606.ENSP00000417052	7
7.51970614587338E-6	7 PTGR1	9606.ENSP00000385763	7
3.260633260558522E-6	7 CLIC4	9606.ENSP00000363500	7
8.814014485358763E-6	7 PCBD1	9606.ENSP00000299299	7
1.3345329209881945E-6	7 PURB	9606.ENSP00000379051	7
4.181392875949897E-5	7 ITM2B	9606.ENSP00000497221	7
3.5248337566652804E-5	7 HDHD3	9606.ENSP00000238379	7
2.8823201032956373E-5	7 LRBA	9606.ENSP00000349629	7
1.5338276900098863E-5	7 PIGU	9606.ENSP00000217446	7
1.3946897579473017E-5	7 GYG1	9606.ENSP00000340736	7
9.665606454937743E-6	7 TMED5	9606.ENSP00000359305	7
6.664401741211661E-6	7 STRIP1	9606.ENSP00000358810	7
1.3220660924283234E-5	7 CNPY2	9606.ENSP00000273308	7
3.1382057838438966E-6	7 DR1	9606.ENSP00000359295	7
6.550292703930509E-5	7 TOR1AIP2	9606.ENSP00000356584	7
1.4271601980606722E-6	7 NPM3	9606.ENSP00000359128	7
9.957343988445452E-6	7 GID8	9606.ENSP00000266069	7
3.67694641565693E-5	7 SUMF2	9606.ENSP00000498402	7
1.298621428718358E-5	7 RDH11	9606.ENSP00000370750	7
9.649767759328437E-6	6 BPNT1	9606.ENSP00000446828	6
1.1387362480062505E-6	6 QTRT2	9606.ENSP00000420682	6
2.172403916731715E-6	6 TOM1L2	9606.ENSP00000368818	6
6.292813023287153E-6	6 SCYL2	9606.ENSP00000489123	6
1.1913206696599013E-5	6 ABHD12	9606.ENSP00000365725	6
5.359661621205618E-6	6 VAT1	9606.ENSP00000347872	6
3.157907455485953E-5	6 RDH13	9606.ENSP00000391121	6
5.967949294141367E-6	6 OSTF1	9606.ENSP00000340836	6
3.397495463520175E-6	6 UFSP2	9606.ENSP00000264689	6
4.346983920937541E-6	6 ZNRD2	9606.ENSP00000312318	6
1.9088664642804173E-5	6 PLOD3	9606.ENSP00000223127	6
3.751555050950662E-6	6 ECM1	9606.ENSP00000358045	6
1.2700302501321467E-5	6 SH2D4A	9606.ENSP00000265807	6
1.0148564349560868E-5	6 APPL2	9606.ENSP00000446917	6
1.4893003198661321E-6	6 ASAP2	9606.ENSP00000281419	6

1.254606194351568E-6	6	EIPR1	9606.ENSP00000381652	6
2.551805288913067E-6	6	PIGS	9606.ENSP00000309430	6
1.4443595978750473E-6	6	PLXNB2	9606.ENSP00000352288	6
1.0299947855269168E-5	6	EPPK1	9606.ENSP00000484472	6
2.529508271243813E-6	6	PEA15	9606.ENSP00000357055	6
2.069419006800546E-6	6	CNN3	9606.ENSP00000359225	6
2.3170325621690447E-6	6	PBDC1	9606.ENSP00000362456	6
2.634859618232127E-5	6	RMDN1	9606.ENSP00000385927	6
4.132012196190645E-6	6	CYP2S1	9606.ENSP00000308032	6
2.0744180975242735E-6	6	RBPMS	9606.ENSP00000340176	6
8.152858485959022E-6	6	KTI12	9606.ENSP00000360676	6
2.017334157867913E-5	6	PAFAH1B3	9606.ENSP00000444935	6
0.0	6	ABR	9606.ENSP00000303909	6
0.0010207142442194533	6	TRABD	9606.ENSP00000379173	6
8.845609881137616E-8	6	GMFB	9606.ENSP00000350757	6
1.2745547749077878E-5	6	BPHL	9606.ENSP00000369739	6
5.134044114573118E-6	5	GOLM1	9606.ENSP00000373364	5
5.263164378870133E-6	5	TTLL12	9606.ENSP00000216129	5
3.1683608962232864E-6	5	FAM49B	9606.ENSP00000429150	5
1.256662923139102E-6	5	BZW2	9606.ENSP00000397249	5
4.985757368223604E-6	5	TRIM56	9606.ENSP00000305161	5
2.0711327211897594E-5	5	OSBPL9	9606.ENSP00000412733	5
1.0741431345163754E-5	5	NRBP1	9606.ENSP00000369192	5
1.3251508157944494E-5	5	COLGALT1	9606.ENSP00000252599	5
1.24249636085574E-6	5	HDDC2	9606.ENSP00000381220	5
0.0	5	THUMPD1	9606.ENSP00000379392	5
1.630584577598839E-5	5	HDHD2	9606.ENSP00000300605	5
2.091389288668575E-6	5	SLC39A7	9606.ENSP00000363809	5
9.817730648092644E-6	5	CDS2	9606.ENSP00000419879	5
1.0881903666022376E-4	5	NBEAL2	9606.ENSP00000415034	5
1.3341326485544427E-5	5	ARMCX3	9606.ENSP00000340672	5
1.0503716460198015E-6	5	PLP2	9606.ENSP00000365505	5
1.0356128776048784E-6	5	ELF1	9606.ENSP00000239882	5
2.808966223060825E-6	5	HDHD5	9606.ENSP00000337358	5
2.3817553666311288E-7	5	EHD4	9606.ENSP00000220325	5
6.301725248145764E-5	5	PCNP	9606.ENSP00000265260	5
8.487293494018526E-6	4	CPNE3	9606.ENSP00000477590	4
9.46990689433721E-6	4	SCCPDH	9606.ENSP00000355467	4
2.199999831929193E-5	4	NDRG3	9606.ENSP00000345292	4
1.8010207216205983E-6	4	PTMS	9606.ENSP00000374113	4
4.845472591855538E-5	4	NAAA	9606.ENSP00000286733	4
2.8525380715031557E-6	4	RUFY1	9606.ENSP00000325594	4
1.4436873509224508E-6	4	SFXN2	9606.ENSP00000358909	4
5.952127892401243E-6	4	LANCL2	9606.ENSP00000254770	4
4.2462794711971275E-6	4	MESD	9606.ENSP00000261758	4
2.8143541552917697E-6	4	SFXN3	9606.ENSP00000224807	4
4.1728900247924795E-6	4	SCPEP1	9606.ENSP00000262288	4
1.700128087494375E-6	4	NUCB2	9606.ENSP00000436455	4

4.601792126440665E-6	4	PDXDC1	9606.ENSP00000379691	4
6.782353901646098E-6	4	CLPTM1	9606.ENSP00000336994	4
3.851465304037135E-6	4	SH3BGRL	9606.ENSP00000362308	4
1.4642586692031701E-5	4	MYADM	9606.ENSP00000375649	4
1.3787219119755752E-5	4	HPCAL1	9606.ENSP00000483786	4
3.2114395339103233E-6	4	MEMO1	9606.ENSP00000295065	4
9.285800837456766E-7	4	PLGRKT	9606.ENSP00000223864	4
4.707622057288166E-6	4	TPD52L2	9606.ENSP00000217121	4
3.1178710621949015E-6	3	DHRS7	9606.ENSP00000216500	3
5.276363537714497E-7	3	HERC4	9606.ENSP00000378624	3
9.459697005570495E-7	3	DTD1	9606.ENSP00000495845	3
1.2363097734216717E-6	3	CAPN5	9606.ENSP00000498132	3
3.33772746674648E-6	3	VIL1	9606.ENSP00000248444	3
0.0	3	MISP	9606.ENSP00000215582	3
2.4681039574189984E-6	3	LAD1	9606.ENSP00000375829	3
6.411051606048867E-7	3	IRF2BP1	9606.ENSP00000307265	3
8.922467379792523E-7	3	SLC25A22	9606.ENSP00000322020	3
7.5381995716647E-7	3	SERPINB9	9606.ENSP00000370074	3
4.154138824758682E-6	3	STARD10	9606.ENSP00000335247	3
7.54260468870517E-6	3	POGLUT1	9606.ENSP00000295588	3
8.632725019749781E-7	3	DDAH1	9606.ENSP00000284031	3
1.819441878102651E-5	3	CNPY3	9606.ENSP00000361926	3
3.0235948594259685E-6	3	PLBD2	9606.ENSP00000280800	3
5.853499623915784E-7	3	HDGFL2	9606.ENSP00000483345	3
9.250213109383765E-6	2	CYP20A1	9606.ENSP00000348380	2
0.0	2	ANO10	9606.ENSP00000292246	2
0.0	2	DDT	9606.ENSP00000381386	2
3.3815053417369207E-6	2	DNPH1	9606.ENSP00000230431	2
1.1547802332994307E-6	2	IKBIP	9606.ENSP00000299157	2
9.394259808999383E-7	2	COBLL1	9606.ENSP00000487041	2
2.833940207547166E-6	2	ACP6	9606.ENSP00000463574	2
1.4311029113261107E-7	2	NIBAN2	9606.ENSP00000362409	2
1.902534292733259E-7	2	NADK2	9606.ENSP00000371362	2
3.6802129296094687E-7	2	TM9SF3	9606.ENSP00000360184	2
1.4309489684541278E-7	2	OXR1	9606.ENSP00000405424	2
6.641697236401098E-6	2	LRRC1	9606.ENSP00000359925	2
3.177237816093857E-7	2	TINAGL1	9606.ENSP00000271064	2
0.0	1	ARMC10	9606.ENSP00000319412	1
0.0	1	VWA5A	9606.ENSP00000407726	1
0.0	1	SERPINB6	9606.ENSP00000484343	1
0.0	1	ANO6	9606.ENSP00000409126	1
0.0	1	LMF2	9606.ENSP00000424381	1
0.0	1	TNFAIP8	9606.ENSP00000427424	1
0.0	1	GALNT7	9606.ENSP00000265000	1
0.0	1	MFSD10	9606.ENSP00000332646	1
0.0	1	FAM162A	9606.ENSP00000419088	1
0.0	1	PPIP5K2	9606.ENSP00000486357	1
0.0	1	MBOAT7	9606.ENSP00000245615	1

0.0	0	VILL	9606.ENSP00000283713	0
0.0	0	LDAH	9606.ENSP00000237822	0
0.0	0	EML2	9606.ENSP00000468312	0
0.0	0	POGLUT3	9606.ENSP00000315386	0
0.0	0	ABRACL	9606.ENSP00000356632	0
0.0	0	PCYOX1L	9606.ENSP00000274569	0
0.0	0	GALNT3	9606.ENSP00000376465	0

name	Term PValue Corrected with Benjamini-Hochberg	UNIQUE_ID
Apoptosis	2.0277466719708354E-19	R-HSA:109581
Intrinsic%20Pathway%20for%20Apoptosis	0.02973053633274273	R-HSA:109606
POLB-Dependent%20Long%20Patch%20Base%20Excision%	0.023818708161433264	R-HSA:110362
Activation%20of%20BAD%20and%20translocation%20to%2	0.008341183710125506	R-HSA:111447
Apoptotic%20cleavage%20of%20cellular%20proteins	1.8000608433922175E-5	R-HSA:111465
Platelet%20degranulation%20	0.014942885275268545	R-HSA:114608
Downstream%20signaling%20events%20of%20B%20Cell%	3.573587058692129E-13	R-HSA:1168372
Activation%20of%20NF-kappaB%20in%20B%20cells	6.195985478926598E-15	R-HSA:1169091
ISG15%20antiviral%20mechanism	1.8723765230605748E-6	R-HSA:1169408
Antiviral%20mechanism%20by%20IFN-stimulated%20gene	6.750291928471725E-7	R-HSA:1169410
ROS%20and%20RNS%20production%20in%20phagocytes	0.009823252427451891	R-HSA:1222556
Cellular%20response%20to%20hypoxia	1.3686116461368238E-11	R-HSA:1234174
Oxygen-dependent%20proline%20hydroxylation%20of%20H	1.9770125818511046E-13	R-HSA:1234176
ER-Phagosome%20pathway	6.17885433582386E-15	R-HSA:1236974
Antigen%20processing-Cross%20presentation	9.527684039124961E-14	R-HSA:1236975
Cross-presentation%20of%20soluble%20exogenous%20an	2.6612976758576216E-17	R-HSA:1236978
Methionine%20salvage%20pathway	0.004357211081235673	R-HSA:1237112
PIP3%20activates%20AKT%20signaling	9.931182929111884E-4	R-HSA:1257604
Developmental%20Biology	1.365713196913256E-9	R-HSA:1266738
Mitochondrial%20protein%20import	3.0882014272599084E-6	R-HSA:1268020
Cytokine%20Signaling%20in%20Immune%20system	8.381705801881707E-5	R-HSA:1280215
Adaptive%20Immune%20System	3.851488416682512E-4	R-HSA:1280218
Apoptosis%20induced%20DNA%20fragmentation	5.435554216055301E-5	R-HSA:140342
Aerobic%20respiration%20and%20respiratory%20electron%	1.1867505695189682E-19	R-HSA:1428517
Metabolism	2.080128042402483E-38	R-HSA:1430728
Translocation%20of%20SLC2A4%20(GLUT4)%20to%20the%	5.990430914118106E-4	R-HSA:1445148
Methylation	0.027595227115477596	R-HSA:156581
Glutathione%20conjugation	0.027912432829980724	R-HSA:156590
L13a-mediated%20translational%20silencing%20of%20Cer	1.8392603915270172E-59	R-HSA:156827
Eukaryotic%20Translation%20Elongation	6.8871955951965436E-46	R-HSA:156842

Peptide%20chain%20elongation	2.6859527003201552E-45	R-HSA:156902
Signaling%20by%20NOTCH	0.004494715506031784	R-HSA:157118
Metabolism%20of%20nucleotides	1.5762579672291363E-4	R-HSA:15869
Mitochondrial%20biogenesis	0.018650676791074714	R-HSA:1592230
Transport%20of%20the%20SLBP%20independent%20Matu	0.0018301671479330301	R-HSA:159227
Transport%20of%20the%20SLBP%20Dependant%20Mature	0.0023661729310671714	R-HSA:159230
Transport%20of%20Mature%20mRNA%20Derived%20from	8.336718466293756E-4	R-HSA:159231
Transport%20of%20Mature%20mRNAs%20Derived%20from	0.0010444918289413947	R-HSA:159234
Transport%20of%20Mature%20mRNA%20derived%20from	8.147805010869634E-7	R-HSA:159236
Sulfide%20oxidation%20to%20sulfate	0.040090734937518226	R-HSA:1614517
Degradation%20of%20cysteine%20and%20homocysteine	0.03671465134813477	R-HSA:1614558
Sulfur%20amino%20acid%20metabolism	0.0018127557160909212	R-HSA:1614635
HIV%20Life%20Cycle	1.9169936015838037E-4	R-HSA:162587
Budding%20and%20maturation%20of%20HIV%20virion	0.02259796391392188	R-HSA:162588
Integration%20of%20provirus	0.023818708161433264	R-HSA:162592
Early%20Phase%20of%20HIV%20Life%20Cycle	0.027595227115477596	R-HSA:162594
Late%20Phase%20of%20HIV%20Life%20Cycle	9.210870192486714E-4	R-HSA:162599
Golgi%20Cisternae%20Pericentriolar%20Stack%20Reorgan	8.958935465244245E-4	R-HSA:162658
HIV%20Infection	3.441394504682432E-20	R-HSA:162906
Host%20Interactions%20of%20HIV%20factors	2.409180874002689E-28	R-HSA:162909
Formation%20of%20ATP%20by%20chemiosmotic%20coup	0.03454512389982467	R-HSA:163210
Cell%20Cycle	1.1206106489942618E-5	R-HSA:1640170
Disease	7.348358181431001E-33	R-HSA:1643685
Nef-mediates%20down%20modulation%20of%20cell%20su	5.609100420625327E-4	R-HSA:164938
The%20role%20of%20Nef%20in%20HIV-1%20replication%	2.9789588066885366E-4	R-HSA:164952
Rev-mediated%20nuclear%20export%20of%20HIV%20RNA	3.649177578673365E-6	R-HSA:165054
mTORC1-mediated%20signalling	0.007100165747890712	R-HSA:166208
Nef%20Mediated%20CD4%20Down-regulation	3.2492799205602557E-4	R-HSA:167590
Innate%20Immune%20System	3.818162869452024E-13	R-HSA:168249
Influenza%20Infection	1.4476615750251674E-48	R-HSA:168255
Immune%20System	6.0719016320399086E-9	R-HSA:168256

Transport%20of%20Ribonucleoproteins%20into%20the%2(	1.7682162814198574E-4	R-HSA:168271
Influenza%20Viral%20RNA%20Transcription%20and%20Re	7.946692196528022E-43	R-HSA:168273
Export%20of%20Viral%20Ribonucleoproteins%20from%20I	7.395710971926386E-5	R-HSA:168274
NS1%20Mediated%20Effects%20on%20Host%20Pathways	3.711987867781279E-5	R-HSA:168276
Viral%20Messenger%20RNA%20Synthesis	0.00430828120208559	R-HSA:168325
NEP/NS2%20Interacts%20with%20the%20Cellular%20Expo	1.7682162814198574E-4	R-HSA:168333
Prolonged%20ERK%20activation%20events	0.0056349509876208285	R-HSA:169893
Regulation%20of%20Apoptosis	4.857987454952864E-18	R-HSA:169911
Regulation%20of%20Glucokinase%20by%20Glucokinase%:	0.0031396596903632114	R-HSA:170822
Signaling%20by%20TGF-beta%20Receptor%20Complex	0.027146740843452876	R-HSA:170834
Frs2-mediated%20activation	0.014246446895949217	R-HSA:170968
Autodegradation%20of%20Cdh1%20by%20Cdh1:APC/C	2.934008208431372E-13	R-HSA:174084
SCF-beta-TrCP%20mediated%20degradation%20of%20Emi	2.40903897795758E-16	R-HSA:174113
APC/C-mediated%20degradation%20of%20cell%20cycle%2	1.1834034115113307E-9	R-HSA:174143
APC/C:Cdc20%20mediated%20degradation%20of%20Secu	2.1943342838182103E-12	R-HSA:174154
APC/C:Cdh1%20mediated%20degradation%20of%20Cdc2(	3.405032345180119E-11	R-HSA:174178
Cdc20:Phospho-APC/C%20mediated%20degradation%20of	2.2052124294655673E-11	R-HSA:174184
Assembly%20Of%20The%20HIV%20Virion	0.047783911609210035	R-HSA:175474
Interactions%20of%20Vpr%20with%20host%20cellular%20	8.61975269643215E-6	R-HSA:176033
Regulation%20of%20APC/C%20activators%20between%20	1.0128143104475571E-10	R-HSA:176408
APC/C:Cdc20%20mediated%20degradation%20of%20mito	7.696990358580419E-11	R-HSA:176409
Activation%20of%20APC/C%20and%20APC/C:Cdc20%20m	1.137403706209467E-10	R-HSA:176814
Interactions%20of%20Rev%20with%20host%20cellular%2(	2.2301797645280275E-7	R-HSA:177243
Retrograde%20neurotrophin%20signalling	8.958935465244245E-4	R-HSA:177504
APC:Cdc20%20mediated%20degradation%20of%20cell%2(	3.405032345180119E-11	R-HSA:179419
SRP-dependent%20cotranslational%20protein%20targeting	8.371089789920577E-51	R-HSA:1799339
Vpu%20mediated%20degradation%20of%20CD4	2.8290879728477095E-16	R-HSA:180534
Vif-mediated%20degradation%20of%20APOBEC3G	1.1569545549610882E-16	R-HSA:180585
APOBEC3G%20mediated%20resistance%20to%20HIV-1%2	0.02296709449582903	R-HSA:180689
Nuclear%20import%20of%20Rev%20protein	1.3764432458071048E-5	R-HSA:180746
Vpr-mediated%20nuclear%20import%20of%20PICs	7.395710971926386E-5	R-HSA:180910

Nef%20Mediated%20CD8%20Down-regulation	7.716774671154945E-4	R-HSA:182218
Signaling%20by%20cytosolic%20FGFR1%20fusion%20muta	0.021041067952343517	R-HSA:1839117
Organelle%20biogenesis%20and%20maintenance	0.022988956714147257	R-HSA:1852241
Signaling%20by%20NTRK1%20(TRKA)	0.04915577830639482	R-HSA:187037
SCF(Skp2)-mediated%20degradation%20of%20p27/p21	6.692855375144122E-15	R-HSA:187577
Signalling%20to%20ERKs	0.019912952557619313	R-HSA:187687
Gap%20junction%20degradation	0.0022772632521632684	R-HSA:190873
snRNP%20Assembly	0.0033793186133392367	R-HSA:191859
Viral%20mRNA%20Translation	6.43952588992417E-44	R-HSA:192823
Signaling%20by%20Rho%20GTPases	1.3830259288008805E-13	R-HSA:194315
Metabolism%20of%20non-coding%20RNA	0.0033793186133392367	R-HSA:194441
Degradation%20of%20beta-catenin%20by%20the%20destr	3.573587058692129E-13	R-HSA:195253
RHO%20GTPase%20Effectors	9.417846032046486E-6	R-HSA:195258
Signaling%20by%20WNT	1.9481202744809332E-4	R-HSA:195721
Formation%20of%20annular%20gap%20junctions	0.009378464334929348	R-HSA:196025
Biotin%20transport%20and%20metabolism	0.049029408830099167	R-HSA:196780
ER%20to%20Golgi%20Anterograde%20Transport	7.991938307941478E-14	R-HSA:199977
Membrane%20Trafficking	1.325828305843867E-23	R-HSA:199991
trans-Golgi%20Network%20Vesicle%20Budding	8.191995822086896E-8	R-HSA:199992
TCF%20dependent%20signaling%20in%20response%20to%	0.0035690408885731867	R-HSA:201681
Metabolism%20of%20nitric%20oxide:%20NOS3%20activat	0.047783911609210035	R-HSA:202131
TCR%20signaling	1.4090252313940131E-8	R-HSA:202403
Downstream%20TCR%20signaling	4.586493654031957E-10	R-HSA:202424
Fcgamma%20receptor%20(FCGR)%20dependent%20phago	1.209565507670488E-4	R-HSA:2029480
Regulation%20of%20actin%20dynamics%20for%20phagoc	1.976625878532007E-7	R-HSA:2029482
COPII-mediated%20vesicle%20transport	9.21596608735982E-6	R-HSA:204005
Regulation%20of%20pyruvate%20dehydrogenase%20(PDH	0.008341183710125506	R-HSA:204174
Basigin%20interactions	0.009220096676786983	R-HSA:210991
Regulation%20of%20activated%20PAK-2p34%20by%20pro	4.934629970930129E-18	R-HSA:211733
MHC%20class%20II%20antigen%20presentation	1.516257501820692E-10	R-HSA:2132295
Cellular%20responses%20to%20stress	2.310087782248495E-48	R-HSA:2262752

Selenoamino%20acid%20metabolism	6.141910291354328E-47	R-HSA:2408522
Selenocysteine%20synthesis	6.023175710158773E-42	R-HSA:2408557
Fc%20epsilon%20receptor%20(FCERI)%20signaling	9.445339599777472E-9	R-HSA:2454202
Separation%20of%20Sister%20Chromatids	4.026740167314147E-9	R-HSA:2467813
Mitotic%20Metaphase%20and%20Anaphase	2.1888791391975973E-12	R-HSA:2555396
Formation%20of%20Senescence-Associated%20Heterochr	0.047783911609210035	R-HSA:2559584
Caspase-mediated%20cleavage%20of%20cytoskeletal%20	0.0022772632521632684	R-HSA:264870
EPH-Ephrin%20signaling	1.4079524413107708E-5	R-HSA:2682334
FCERI%20mediated%20NF-kB%20activation	1.362638849803714E-12	R-HSA:2871837
Nuclear%20Envelope%20Breakdown	9.208570982353685E-4	R-HSA:2980766
SUMOylation	0.010858406261057718	R-HSA:2990846
Initiation%20of%20Nuclear%20Envelope%20(NE)%20Refor	0.0013712615713252323	R-HSA:2995383
Nuclear%20Envelope%20(NE)%20Reassembly	1.2365348771941576E-4	R-HSA:2995410
Laminin%20interactions	0.02730421438037538	R-HSA:3000157
Scavenging%20by%20Class%20F%20Receptors	0.004357211081235673	R-HSA:3000484
SUMO%20is%20conjugated%20to%20E1%20(UBA2:SAE1)	0.02296709449582903	R-HSA:3065676
SUMO%20is%20transferred%20from%20E1%20to%20E2%2	0.008800226997841193	R-HSA:3065678
SUMOylation%20of%20DNA%20damage%20response%20a	0.018621023192867583	R-HSA:3108214
SUMO%20E3%20ligases%20SUMOylate%20target%20prote	0.020213378432259208	R-HSA:3108232
Processing%20and%20activation%20of%20SUMO	0.03493787212115158	R-HSA:3215018
SUMOylation%20of%20ubiquitinylation%20proteins	0.0013913259956069301	R-HSA:3232142
Detoxification%20of%20Reactive%20Oxygen%20Species	1.241031521535156E-5	R-HSA:3299685
Nuclear%20Pore%20Complex%20(NPC)%20Disassembly	0.00804886392591975	R-HSA:3301854
Defects%20in%20biotin%20(Btn)%20metabolism	0.015325773392222491	R-HSA:3323169
Regulation%20of%20HSF1-mediated%20heat%20shock%2	1.364555600055487E-7	R-HSA:3371453
HSP90%20chaperone%20cycle%20for%20steroid%20horm	1.1555137633330041E-7	R-HSA:3371497
HSF1%20activation	2.7562280534308226E-4	R-HSA:3371511
Cellular%20response%20to%20heat%20stress	2.2832226088640748E-9	R-HSA:3371556
Attenuation%20phase	8.958935465244245E-4	R-HSA:3371568
HSF1-dependent%20transactivation	0.007100165747890712	R-HSA:3371571
Defective%20HLCS%20causes%20multiple%20carboxylase	0.008800226997841193	R-HSA:3371599

Autodegradation%20of%20the%20E3%20ubiquitin%20ligas	2.8290879728477095E-16	R-HSA:349425
Regulation%20of%20ornithine%20decarboxylase%20(ODC)	1.3115477192478033E-16	R-HSA:350562
Metabolism%20of%20polyamines	3.663802026336864E-15	R-HSA:351202
Apoptotic%20cleavage%20of%20cell%20adhesion%20%20	0.009378464334929348	R-HSA:351906
Nephrin%20family%20interactions	0.02023614244391323	R-HSA:373753
Semaphorin%20interactions	0.02087961542964951	R-HSA:373755
L1CAM%20interactions	0.0011990454349737065	R-HSA:373760
Signaling%20by%20ROBO%20receptors	2.0353054854702336E-50	R-HSA:376176
Cytosolic%20tRNA%20aminoacylation	2.6641869485217354E-20	R-HSA:379716
tRNA%20Aminoacylation	2.7190208156665424E-13	R-HSA:379724
XBP1(S)%20activates%20chaperone%20genes	4.5638628429130696E-7	R-HSA:381038
IRE1alpha%20activates%20chaperones	1.8733861083060336E-7	R-HSA:381070
Unfolded%20Protein%20Response%20(UPR)	2.6672663253666585E-6	R-HSA:381119
Transport%20of%20small%20molecules	0.007237171876525452	R-HSA:382551
ABC-family%20proteins%20mediated%20transport	5.538263418024355E-12	R-HSA:382556
Beta-catenin%20independent%20WNT%20signaling	9.045041192256801E-11	R-HSA:3858494
Glyoxylate%20metabolism%20and%20glycine%20degradat	0.0013712615713252323	R-HSA:389661
Prefoldin%20mediated%20transfer%20of%20substrate%20	5.6405864639435746E-5	R-HSA:389957
Cooperation%20of%20Prefoldin%20and%20TriC/CCT%20%	2.4962223576413376E-4	R-HSA:389958
Formation%20of%20tubulin%20folding%20intermediates%:	0.009220096676786983	R-HSA:389960
Folding%20of%20actin%20by%20CCT/TriC	9.724693272981416E-8	R-HSA:390450
Chaperonin-mediated%20protein%20folding	6.538824347304906E-4	R-HSA:390466
Association%20of%20TriC/CCT%20with%20target%20prote	8.694004535218912E-5	R-HSA:390471
Protein%20folding	6.52749254119751E-4	R-HSA:391251
Metabolism%20of%20proteins	1.2080300879564681E-55	R-HSA:392499
EPHB-mediated%20forward%20signaling	0.0010444918289413947	R-HSA:3928662
Trafficking%20of%20AMPA%20receptors	0.03271673120448039	R-HSA:399719
Glutamate%20binding,%20activation%20of%20AMPA%20re	0.03271673120448039	R-HSA:399721
Sema3A%20PAK%20dependent%20Axon%20repulsion	0.047783911609210035	R-HSA:399954
SUMOylation%20of%20SUMOylation%20proteins	0.0018301671479330301	R-HSA:4085377
PCP/CE%20pathway	2.495914619262216E-15	R-HSA:4086400

Axon%20guidance	4.8999867235535345E-33	R-HSA:422475
Deadenylation-dependent%20mRNA%20decay	1.6521422478412258E-4	R-HSA:429914
Deadenylation%20of%20mRNA	0.0016833831387830052	R-HSA:429947
mRNA%20decay%20by%203'%20to%205'%20exoribonucle	0.047783911609210035	R-HSA:429958
Lysosome%20Vesicle%20Biogenesis	1.478005503238703E-4	R-HSA:432720
Golgi%20Associated%20Vesicle%20Biogenesis	7.996542958918238E-7	R-HSA:432722
Recycling%20pathway%20of%20L1	0.009139514294766848	R-HSA:437239
VEGFA-VEGFR2%20Pathway	0.042737895027924765	R-HSA:4420097
Signal%20transduction%20by%20L1	0.012228364415750505	R-HSA:445144
Asparagine%20N-linked%20glycosylation	5.322855888127953E-15	R-HSA:446203
Localization%20of%20the%20PINCH-ILK-PARVIN%20compl	0.010844236296116238	R-HSA:446343
Cell-extracellular%20matrix%20interactions	0.004825642317469044	R-HSA:446353
Interleukin-1%20family%20signaling	1.7024786268369355E-7	R-HSA:446652
Interleukin-12%20family%20signaling	7.949374278890972E-14	R-HSA:447115
Signaling%20by%20Interleukins	1.0326449531022741E-5	R-HSA:449147
AUF1%20(hnRNP%20D0)%20binds%20and%20destabilizes	2.8319603181024936E-20	R-HSA:450408
HuR%20(ELAVL1)%20binds%20and%20stabilizes%20mRNA	0.0017290637260829632	R-HSA:450520
Regulation%20of%20mRNA%20stability%20by%20proteins	1.7849706475338433E-22	R-HSA:450531
Mitotic%20G2-G2/M%20phases	1.643382084981039E-7	R-HSA:453274
Regulation%20of%20mitotic%20cell%20cycle	1.1834034115113307E-9	R-HSA:453276
Mitotic%20G1%20phase%20and%20G1/S%20transition	6.750291928471725E-7	R-HSA:453279
SUMOylation%20of%20chromatin%20organization%20prote	0.0415108199225461	R-HSA:4551638
SUMOylation%20of%20RNA%20binding%20proteins	9.937218294980663E-4	R-HSA:4570464
Asymmetric%20localization%20of%20PCP%20proteins	7.018254797589464E-14	R-HSA:4608870
SUMOylation%20of%20DNA%20replication%20proteins	1.6916829909224208E-4	R-HSA:4615885
Degradation%20of%20AXIN	2.4659682848450035E-15	R-HSA:4641257
Degradation%20of%20DVL	9.739450323071855E-16	R-HSA:4641258
WNT5A-dependent%20internalization%20of%20FZD4	0.008341183710125506	R-HSA:5099900
WNT5A-dependent%20internalization%20of%20FZD2,%20F	0.0036750632266488773	R-HSA:5140745
N-glycan%20trimming%20in%20the%20ER%20and%20Cal	1.478005503238703E-4	R-HSA:532668
Uptake%20and%20function%20of%20diphtheria%20toxin	0.040090734937518226	R-HSA:5336415

Programmed%20Cell%20Death	2.1342791703404944E-19	R-HSA:5357801
Hedgehog%20ligand%20biogenesis	2.5513380711563946E-17	R-HSA:5358346
Signaling%20by%20Hedgehog	2.503474720253072E-8	R-HSA:5358351
Hh%20mutants%20are%20degraded%20by%20ERAD	3.596884895445331E-19	R-HSA:5362768
Hh%20mutants%20abrogate%20ligand%20secretion	3.9309801162904394E-18	R-HSA:5387390
Dectin-1%20mediated%20noncanonical%20NF-kB%20sign	7.550940470865407E-16	R-HSA:5607761
CLEC7A%20(Dectin-1)%20signaling	2.712645880164617E-10	R-HSA:5607764
Diseases%20associated%20with%20glycosylation%20prec	0.03671465134813477	R-HSA:5609975
Degradation%20of%20GLI1%20by%20the%20proteasome	6.692855375144122E-15	R-HSA:5610780
Degradation%20of%20GLI2%20by%20the%20proteasome	7.550940470865407E-16	R-HSA:5610783
GLI3%20is%20processed%20to%20GLI3R%20by%20the%2	7.550940470865407E-16	R-HSA:5610785
Hedgehog%20'off'%20state	8.650067065950532E-9	R-HSA:5610787
ABC%20transporter%20disorders	3.761300078801272E-14	R-HSA:5619084
Defective%20TPR%20may%20confer%20susceptibility%20	0.0031396596903632114	R-HSA:5619107
Disorders%20of%20transmembrane%20transporters	2.2615113356578045E-9	R-HSA:5619115
Cargo%20trafficking%20to%20the%20periciliary%20memb	0.017381459648485382	R-HSA:5620920
C-type%20lectin%20receptors%20(CLRs)	1.8634108046556842E-6	R-HSA:5621481
RHO%20GTPases%20activate%20CIT	0.03454512389982467	R-HSA:5625900
RHO%20GTPases%20activate%20KTN1	1.3388232704384943E-4	R-HSA:5625970
RHO%20GTPases%20activate%20IQGAPs	0.039005672978073926	R-HSA:5626467
RHO%20GTPases%20Activate%20ROCKs	0.00924006443740262	R-HSA:5627117
RHO%20GTPases%20activate%20PAKs	0.0016833831387830052	R-HSA:5627123
TP53%20Regulates%20Metabolic%20Genes	0.003982841111008102	R-HSA:5628897
Hedgehog%20'on'%20state	6.018823754929302E-10	R-HSA:5632684
Vesicle-mediated%20transport	5.86375323391938E-23	R-HSA:5653656
Regulation%20of%20RAS%20by%20GAPs	5.456582389538528E-13	R-HSA:5658442
CLEC7A/inflammasome%20pathway	0.040090734937518226	R-HSA:5660668
Diseases%20of%20signal%20transduction%20by%20growt	4.5851432006605494E-10	R-HSA:5663202
Infectious%20disease	1.4538932153570434E-48	R-HSA:5663205
RHO%20GTPases%20Activate%20WASPs%20and%20WAVE	2.2301797645280275E-7	R-HSA:5663213
TNFR2%20non-canonical%20NF-kB%20pathway	9.909148593455424E-8	R-HSA:5668541

RAF/MAP%20kinase%20cascade	4.2948710752132194E-5	R-HSA:5673001
MAP2K%20and%20MAPK%20activation	6.419294203886037E-4	R-HSA:5674135
Negative%20feedback%20regulation%20of%20MAPK%20pa	0.004357211081235673	R-HSA:5674499
NIK-->noncanonical%20NF-kB%20signaling	3.663802026336864E-15	R-HSA:5676590
Defective%20CFTR%20causes%20cystic%20fibrosis	1.593976060991522E-18	R-HSA:5678895
MAPK%20family%20signaling%20cascades	6.232580083653811E-5	R-HSA:5683057
MAPK1/MAPK3%20signaling	7.76794515083954E-5	R-HSA:5684996
MAPK6/MAPK4%20signaling	7.779532221334271E-13	R-HSA:5687128
Deubiquitination	3.058473976360178E-5	R-HSA:5688426
UCH%20proteinases	3.876341392551951E-12	R-HSA:5689603
Ub-specific%20processing%20proteases	7.628750704175157E-6	R-HSA:5689880
Cargo%20concentration%20in%20the%20ER	0.0014019298090037862	R-HSA:5694530
DNA%20Damage%20Recognition%20in%20GG-NER	0.004735920012720006	R-HSA:5696394
Global%20Genome%20Nucleotide%20Excision%20Repair%	0.019485382225287083	R-HSA:5696399
Post-translational%20protein%20modification	2.7185717133547714E-10	R-HSA:597592
Respiratory%20electron%20transport	1.3613368410588422E-10	R-HSA:611105
Glycine%20degradation	7.716774671154945E-4	R-HSA:6783984
tRNA%20processing%20in%20the%20nucleus	0.006759981001181242	R-HSA:6784531
rRNA%20modification%20in%20the%20nucleus%20and%2	0.004866172694588848	R-HSA:6790901
Major%20pathway%20of%20rRNA%20processing%20in%2(	6.944351869835 94E-26	R-HSA:6791226
Neutrophil%20degranulation	4.919308852685942E-23	R-HSA:6798695
Complex%20I%20biogenesis	9.944892641604968E-8	R-HSA:6799198
Signaling%20by%20moderate%20kinase%20activity%20BR.	9.937218294980663E-4	R-HSA:6802946
Signaling%20by%20high-kinase%20activity%20BRAF%20mu	8.333709291268981E-4	R-HSA:6802948
Signaling%20by%20RAS%20mutants	9.937218294980663E-4	R-HSA:6802949
Signaling%20by%20BRAF%20and%20RAF1%20fusions	0.0018678544523010269	R-HSA:6802952
Paradoxical%20activation%20of%20RAF%20signaling%20b	9.937218294980663E-4	R-HSA:6802955
Oncogenic%20MAPK%20signaling	0.003982841111008102	R-HSA:6802957
FGFR2%20alternative%20splicing	0.011803835924824611	R-HSA:6803529
Signaling%20by%20MET	0.046804392539274785	R-HSA:6806834
PTEN%20Regulation	2.5681656223439446E-10	R-HSA:6807070

COPI-mediated%20anterograde%20transport	6.305116407147402E-11	R-HSA:6807878
COPI-dependent%20Golgi-to-ER%20retrograde%20traffic	5.682693874193504E-5	R-HSA:6811434
COPI-independent%20Golgi-to-ER%20retrograde%20traffic	5.825212471421665E-5	R-HSA:6811436
Intra-Golgi%20traffic	0.005206202554548669	R-HSA:6811438
Intra-Golgi%20and%20retrograde%20Golgi-to-ER%20traffic	1.694255932487046E-10	R-HSA:6811442
Cooperation%20of%20PDCL%20(PhLP1)%20and%20TRiC/(	0.0013913259956069301	R-HSA:6814122
Assembly%20of%20the%20pre-replicative%20complex	4.347079015601306E-7	R-HSA:68867
Mitotic%20Prophase	0.006653900395551148	R-HSA:68875
Mitotic%20Anaphase	1.7926805203682023E-12	R-HSA:68882
M%20Phase	4.245403146969399E-9	R-HSA:68886
Orc1%20removal%20from%20chromatin	5.403278899489953E-14	R-HSA:68949
DNA%20Replication%20Pre-Initiation	3.259757281719248E-6	R-HSA:69002
CDK-mediated%20phosphorylation%20and%20removal%2(	2.2052124294655673E-11	R-HSA:69017
Switching%20of%20origins%20to%20a%20post-replicative%	2.1222562854895692E-10	R-HSA:69052
Cyclin%20E%20associated%20events%20during%20G1/S%	6.248754307683719E-11	R-HSA:69202
G1/S%20Transition	5.593065277516576E-8	R-HSA:69206
Synthesis%20of%20DNA	1.4090252313940131E-8	R-HSA:69239
S%20Phase	3.153077078914496E-7	R-HSA:69242
G2/M%20Transition	1.1905760361825963E-7	R-HSA:69275
Cell%20Cycle,%20Mitotic	2.1773554256932377E-6	R-HSA:69278
DNA%20Replication	1.3922649048569047E-5	R-HSA:69306
G2/M%20Checkpoints	2.7531895411190515E-7	R-HSA:69481
Stabilization%20of%20p53	8.91496143414775E-15	R-HSA:69541
p53-Dependent%20G1%20DNA%20Damage%20Response	1.365031286057484E-12	R-HSA:69563
p53-Dependent%20G1/S%20DNA%20damage%20checkpoi	1.365031286057484E-12	R-HSA:69580
Ubiquitin%20Mediated%20Degradation%20of%20Phosphor	2.8290879728477095E-16	R-HSA:69601
p53-Independent%20DNA%20Damage%20Response	2.8290879728477095E-16	R-HSA:69610
p53-Independent%20G1/S%20DNA%20damage%20checkp	2.8290879728477095E-16	R-HSA:69613
G1/S%20DNA%20Damage%20Checkpoints	3.580426488590702E-12	R-HSA:69615
Cell%20Cycle%20Checkpoints	4.2880177863518645E-6	R-HSA:69620
Cyclin%20A:Cdk2-associated%20events%20at%20S%20ph	1.2667153963460705E-10	R-HSA:69656

Glycolysis	4.574216952913044E-7	R-HSA:70171
Gluconeogenesis	0.0031191698432451124	R-HSA:70263
Pyruvate%20metabolism	0.004623886959750267	R-HSA:70268
Glucose%20metabolism	2.341382553430328E-6	R-HSA:70326
Branched-chain%20amino%20acid%20catabolism	1.2996344346786132E-7	R-HSA:70895
Propionyl-CoA%20catabolism	0.02296709449582903	R-HSA:71032
Metabolism%20of%20amino%20acids%20and%20derivativ	4.26757061210635E-55	R-HSA:71291
Pentose%20phosphate%20pathway	0.03671465134813477	R-HSA:71336
Citric%20acid%20cycle%20(TCA%20cycle)	9.546005708568035E-12	R-HSA:71403
mRNA%20Splicing%20-%20Major%20Pathway	2.453629718220224E-24	R-HSA:72163
mRNA%20Splicing%20-%20Minor%20Pathway	2.3068818549506923E-6	R-HSA:72165
mRNA%20Splicing	4.365529723158376E-23	R-HSA:72172
mRNA%203'-end%20processing	4.6674205247368564E-7	R-HSA:72187
Transport%20of%20Mature%20Transcript%20to%20Cytopla	1.6533509070733813E-7	R-HSA:72202
Processing%20of%20Capped%20Intron-Containing%20Pre-	1.5894794217295802E-26	R-HSA:72203
tRNA%20processing	0.04628268432524366	R-HSA:72306
rRNA%20processing	6.819232912448712E-27	R-HSA:72312
Eukaryotic%20Translation%20Initiation	3.158811313296831E-60	R-HSA:72613
Translation%20initiation%20complex%20formation	2.793581619312171E-36	R-HSA:72649
Activation%20of%20the%20mRNA%20upon%20binding%2(	1.3178514442504152E-35	R-HSA:72662
Formation%20of%20a%20pool%20of%20free%2040S%20s	1.8358270464026635E-52	R-HSA:72689
Formation%20of%20the%20ternary%20complex,%20and%:	3.793006996022712E-30	R-HSA:72695
Ribosomal%20scanning%20and%20start%20codon%20rec	2.793581619312171E-36	R-HSA:72702
GTP%20hydrolysis%20and%20joining%20of%20the%2060S	4.0723340370698285E-60	R-HSA:72706
Recycling%20of%20eIF2:GDP	0.015325773392222491	R-HSA:72731
Cap-dependent%20Translation%20Initiation	3.158811313296831E-60	R-HSA:72737
Eukaryotic%20Translation%20Termination	6.8871955951965436E-46	R-HSA:72764
Translation	7.595690253868355E-60	R-HSA:72766
Purine%20ribonucleoside%20monophosphate%20biosynth	9.425985878480443E-6	R-HSA:73817
RNA%20Polymerase%20II%20Transcription%20Termination	4.33238511958709E-7	R-HSA:73856
Purine%20salvage	0.02018968144712899	R-HSA:74217

Chk1/Chk2(Cds1)%20mediated%20inactivation%20of%20(	0.0036750632266488773	R-HSA:75035
Processing%20of%20Capped%20Intronless%20Pre-mRNA	0.006917846516270979	R-HSA:75067
Apoptotic%20execution%20phase	8.737125180605633E-10	R-HSA:75153
Ubiquitin-dependent%20degradation%20of%20Cyclin%20D	2.5465842590671353E-17	R-HSA:75815
Response%20to%20elevated%20platelet%20cytosolic%20(	0.02269948153948981	R-HSA:76005
mitochondrial%20fatty%20acid%20beta-oxidation%20of%2	1.3388232704384943E-4	R-HSA:77286
mitochondrial%20fatty%20acid%20beta-oxidation%20of%2	0.004357211081235673	R-HSA:77288
Mitochondrial%20Fatty%20Acid%20Beta-Oxidation	2.2915053163187226E-6	R-HSA:77289
Beta%20oxidation%20of%20palmitoyl-CoA%20to%20myris	0.0032455363520066246	R-HSA:77305
Beta%20oxidation%20of%20lauroyl-CoA%20to%20decanoy	0.0017126266579375919	R-HSA:77310
Beta%20oxidation%20of%20decanoyl-CoA%20to%20octan	6.610765601365051E-6	R-HSA:77346
Beta%20oxidation%20of%20octanoyl-CoA%20to%20hexan	5.3456383395451676E-5	R-HSA:77348
Beta%20oxidation%20of%20hexanoyl-CoA%20to%20butan	0.0017126266579375919	R-HSA:77350
Insulin%20receptor%20recycling	1.2392797829165718E-4	R-HSA:77387
Processing%20of%20Intronless%20Pre-mRNAs	0.00924006443740262	R-HSA:77595
The%20NLRP3%20inflammasome	0.047783911609210035	R-HSA:844456
Advanced%20glycosylation%20endproduct%20receptor%2(	5.156826583740949E-4	R-HSA:879415
The%20role%20of%20GTSE1%20in%20G2/M%20progressi	9.19272098273731E-13	R-HSA:8852276
FBXL7%20down-regulates%20AURKA%20during%20mitotic	2.40903897795758E-16	R-HSA:8854050
Golgi-to-ER%20retrograde%20transport	1.5453889436302952E-10	R-HSA:8856688
Cargo%20recognition%20for%20clathrin-mediated%20end	4.773433883087076E-4	R-HSA:8856825
Clathrin-mediated%20endocytosis	1.1184282876019735E-9	R-HSA:8856828
Deregulated%20CDK5%20triggers%20multiple%20neurode	1.472931323987047E-4	R-HSA:8862803
Neurodegenerative%20Diseases	1.472931323987047E-4	R-HSA:8863678
VLDLR%20internalisation%20and%20degradation	0.011721283124234276	R-HSA:8866427
rRNA%20processing%20in%20the%20nucleus%20and%20	6.2663365041691185E-28	R-HSA:8868773
TFAP2A%20acts%20as%20a%20transcriptional%20repress	0.02296709449582903	R-HSA:8869496
MET%20activates%20RAP1%20and%20RAC1	0.009378464334929348	R-HSA:8875555
MET%20promotes%20cell%20motility	0.008769593859171337	R-HSA:8875878
Listeria%20monocytogenes%20entry%20into%20host%20c	0.00924006443740262	R-HSA:8876384
Protein%20methylation	0.02740648006131623	R-HSA:8876725

Transcriptional%20regulation%20by%20RUNX3	1.0835717094726128E-9	R-HSA:8878159
Transcriptional%20regulation%20by%20RUNX2	3.828457465175742E-9	R-HSA:8878166
Transcriptional%20regulation%20by%20RUNX1	8.531884213892728E-4	R-HSA:8878171
RUNX1%20regulates%20transcription%20of%20genes%20i	3.24479022971638E-6	R-HSA:8939236
Regulation%20of%20RUNX2%20expression%20and%20act	1.236114847733046E-14	R-HSA:8939902
Regulation%20of%20RUNX3%20expression%20and%20act	2.40903897795758E-16	R-HSA:8941858
Regulation%20of%20PTEN%20stability%20and%20activity	1.8468970588881752E-14	R-HSA:8948751
Mitochondrial%20calcium%20ion%20transport	2.2642369175234907E-4	R-HSA:8949215
Cristae%20formation	3.449158335203386E-4	R-HSA:8949613
Processing%20of%20SMDT1	0.011721283124234276	R-HSA:8949664
Gene%20and%20protein%20expression%20by%20JAK-STA[illegible]	4.2880145466526045E-17	R-HSA:8950505
Neddylation	4.052567465758404E-5	R-HSA:8951664
Metabolism%20of%20RNA	3.560885966630894E-66	R-HSA:8953854
Cellular%20responses%20to%20stimuli	1.6890537206321612E-47	R-HSA:8953897
Nucleotide%20biosynthesis	1.0057329147229143E-5	R-HSA:8956320
LDL%20clearance	0.0013712615713252323	R-HSA:8964038
Plasma%20lipoprotein%20clearance	0.03293280381207297	R-HSA:8964043
Glutamate%20and%20glutamine%20metabolism	0.0056349509876208285	R-HSA:8964539
Fatty%20acid%20metabolism	0.003669060639257484	R-HSA:8978868
Intracellular%20signaling%20by%20second%20messenger[illegible]	0.002642676800985368	R-HSA:9006925
Signaling%20by%20Receptor%20Tyrosine%20Kinases	0.03080312761697694	R-HSA:9006934
Calnexin/calreticulin%20cycle	0.011803835924824611	R-HSA:901042
Regulation%20of%20expression%20of%20SLITs%20and%2	1.8299471382386526E-56	R-HSA:9010553
RHO%20GTPase%20cycle	7.126108280264653E-11	R-HSA:9012999
RHOC%20GTPase%20cycle	0.027678285810630775	R-HSA:9013106
RAC2%20GTPase%20cycle	4.7388932419368184E-4	R-HSA:9013404
RHOD%20GTPase%20cycle	4.44842354449871E-4	R-HSA:9013405
RHOQ%20GTPase%20cycle	0.0012401023286369892	R-HSA:9013406
RHOH%20GTPase%20cycle	0.014514351747612031	R-HSA:9013407
RHOG%20GTPase%20cycle	1.0003379874396029E-4	R-HSA:9013408
RHOJ%20GTPase%20cycle	0.0017003172897406334	R-HSA:9013409

RHOBTB2%20GTPase%20cycle	1.5747498317331587E-11	R-HSA:9013418
RHOU%20GTPase%20cycle	6.419294203886037E-4	R-HSA:9013420
RHOBTB1%20GTPase%20cycle	5.358779131543862E-8	R-HSA:9013422
RAC3%20GTPase%20cycle	0.013646193078642984	R-HSA:9013423
RHOV%20GTPase%20cycle	0.004735920012720006	R-HSA:9013424
Signaling%20by%20NOTCH4	1.362638849803714E-12	R-HSA:9013694
Interleukin-12%20signaling	8.87713981119258E-15	R-HSA:9020591
Interleukin-1%20signaling	6.224779229824222E-11	R-HSA:9020702
RHOF%20GTPase%20cycle	0.0010444918289413947	R-HSA:9035034
Interferon%20Signaling	0.016530627034114825	R-HSA:913531
Endosomal%20Sorting%20Complex%20Required%20For%2	0.039005672978073926	R-HSA:917729
Iron%20uptake%20and%20transport	0.001019389545529158	R-HSA:917937
Transferrin%20endocytosis%20and%20recycling	0.0031396596903632114	R-HSA:917977
Nonsense-Mediated%20Decay%20(NMD)	1.9542417057810646E-43	R-HSA:927802
Transport%20to%20the%20Golgi%20and%20subsequent%	1.7459425824052184E-11	R-HSA:948021
Negative%20regulation%20of%20NOTCH4%20signaling	1.0814719499672745E-17	R-HSA:9604323
Protein%20localization	1.6240320890488439E-7	R-HSA:9609507
Insertion%20of%20tail-anchored%20proteins%20into%20th	8.347822868266679E-4	R-HSA:9609523
HCMV%20Infection	0.01867285252018139	R-HSA:9609646
Autophagy	0.02735342119162148	R-HSA:9612973
Regulation%20of%20localization%20of%20FOXO%20trans	0.049029408830099167	R-HSA:9614399
Postmitotic%20nuclear%20pore%20complex%20(NPC)%20	5.675446499831339E-6	R-HSA:9615933
Protein%20hydroxylation	3.6619469288737543E-4	R-HSA:9629569
Response%20of%20EIF2AK4%20(GCN2)%20to%20amino%	7.828405941647348E-41	R-HSA:9633012
Infection%20with%20Mycobacterium%20tuberculosis	9.888505584413414E-4	R-HSA:9635486
Suppression%20of%20phagosomal%20maturation	0.02018968144712899	R-HSA:9637687
Response%20of%20Mtb%20to%20phagocytosis	2.2642369175234907E-4	R-HSA:9637690
Amino%20acids%20regulate%20mTORC1	0.0048050268444883824	R-HSA:9639288
Signaling%20downstream%20of%20RAS%20mutants	9.937218294980663E-4	R-HSA:9649948
Signaling%20by%20MAP2K%20mutants	4.221384886145331E-4	R-HSA:9652169
Signaling%20by%20RAF1%20mutants	0.00430828120208559	R-HSA:9656223

Leishmania%20infection	0.001118866126249246	R-HSA:9658195
Sensory%20processing%20of%20sound	0.003073536904471067	R-HSA:9659379
Sensory%20processing%20of%20sound%20by%20inner%2	0.002618620951398609	R-HSA:9662360
Sensory%20processing%20of%20sound%20by%20outer%2	0.010837930469264042	R-HSA:9662361
Selective%20autophagy	0.016366623792064244	R-HSA:9663891
Parasite%20infection	9.043581420097893E-6	R-HSA:9664407
Leishmania%20phagocytosis	9.043581420097893E-6	R-HSA:9664417
FCGR3A-mediated%20phagocytosis	9.043581420097893E-6	R-HSA:9664422
Nervous%20system%20development	4.677967080574325E-31	R-HSA:9675108
SARS-CoV-1%20Infection	5.5281038160791395E-17	R-HSA:9678108
Potential%20therapeutics%20for%20SARS	0.002378186752048539	R-HSA:9679191
SARS-CoV%20Infections	6.180834770134268E-17	R-HSA:9679506
Maturation%20of%20spike%20protein	0.02296709449582903	R-HSA:9683686
SARS-CoV-1-host%20interactions	1.8424494524647031E-19	R-HSA:9692914
SARS-CoV-2%20Infection	9.12525494115953E-13	R-HSA:9694516
Maturation%20of%20spike%20protein	0.03293280381207297	R-HSA:9694548
Signaling%20by%20ALK%20in%20cancer	1.1532525546452713E-5	R-HSA:9700206
ALK%20mutants%20bind%20TKIs	0.014246446895949217	R-HSA:9700645
SARS-CoV-2-host%20interactions	1.938706630562041E-12	R-HSA:9705683
RHOBTB%20GTPase%20Cycle	1.3722140212625239E-13	R-HSA:9706574
Cellular%20response%20to%20starvation	2.1679710303192306E-37	R-HSA:9711097
Cellular%20response%20to%20chemical%20stress	3.452640768001065E-17	R-HSA:9711123
Signaling%20by%20Rho%20GTPases,%20Miro%20GTPases	1.8735134173831068E-13	R-HSA:9716542
Signaling%20by%20ALK%20fusions%20and%20activated%	1.1532525546452713E-5	R-HSA:9725370
MITF-M-regulated%20melanocyte%20development	1.0833255311331834E-5	R-HSA:9730414
Defective%20Intrinsic%20Pathway%20for%20Apoptosis	4.979324726659241E-4	R-HSA:9734009
Diseases%20of%20nucleotide%20metabolism	0.010844236296116238	R-HSA:9735804
SARS-CoV-1%20modulates%20host%20translation%20mac	1.0679419699723655E-21	R-HSA:9735869
SARS-CoV-1%20targets%20host%20intracellular%20signall	2.019901876132612E-4	R-HSA:9735871
SARS-CoV-2%20modulates%20host%20translation%20mac	1.1473957030754789E-17	R-HSA:9754678
Ribavirin%20ADME	0.014246446895949217	R-HSA:9755088

KEAP1-NFE2L2%20pathway	5.139787245969016E-16	R-HSA:9755511
SARS-CoV-2%20targets%20host%20intracellular%20signall	0.0022772632521632684	R-HSA:9755779
Gastrulation	1.5346180132801236E-6	R-HSA:9758941
Nuclear%20events%20mediated%20by%20NFE2L2	1.510451270374543E-17	R-HSA:9759194
Nonsense%20Mediated%20Decay%20(NMD)%20independ	3.521306783415425E-46	R-HSA:975956
Nonsense%20Mediated%20Decay%20(NMD)%20enhanced	1.9542417057810646E-43	R-HSA:975957
GSK3B%20and%20BTRC:CUL1-mediated-degradation%20o	2.1200408397489837E-18	R-HSA:9762114
Regulation%20of%20CDH11%20function	0.049029408830099167	R-HSA:9762292
Regulation%20of%20CDH19%20Expression%20and%20Fu	0.008800226997841193	R-HSA:9764302
Formation%20of%20paraxial%20mesoderm	1.3447729374050525E-13	R-HSA:9793380
M-decay:%20degradation%20of%20maternal%20mRNAs%2	0.0016833831387830052	R-HSA:9820841
Z-decay:%20degradation%20of%20maternal%20mRNAs%2	2.4003492645736702E-5	R-HSA:9820865
Respiratory%20syncytial%20virus%20(RSV)%20genome%2	4.319250696975896E-5	R-HSA:9820965
Somitogenesis	2.40903897795758E-16	R-HSA:9824272
Bacterial%20Infection%20Pathways	2.4218178847357182E-5	R-HSA:9824439
Parasitic%20Infection%20Pathways	0.001118866126249246	R-HSA:9824443
Viral%20Infection%20Pathways	7.885437786957497E-44	R-HSA:9824446
Maturation%20of%20hRSV%20A%20proteins	0.0036750632266488773	R-HSA:9828806
Antigen%20processing:%20Ubiquitination%20&%20Proteas	0.018511206710709067	R-HSA:983168
Class%20I%20MHC%20mediated%20antigen%20processir	3.425943361248562E-4	R-HSA:983169
Antigen%20Presentation:%20Folding,%20assembly%20and	0.0018127557160909212	R-HSA:983170
CDH11%20homotypic%20and%20heterotypic%20interactic	0.008800226997841193	R-HSA:9833576
Signaling%20by%20the%20B%20Cell%20Receptor%20(BCI	4.2330862215758194E-10	R-HSA:983705
Mitochondrial%20protein%20degradation	1.0939775071410713E-21	R-HSA:9837999
Cellular%20response%20to%20mitochondrial%20stress	0.023818708161433264	R-HSA:9840373
OGDH%20complex%20synthesizes%20succinyl-CoA%20frc	4.221384886145331E-4	R-HSA:9853506
Maturation%20of%20TCA%20enzymes%20and%20regulatic	0.0019961085382879727	R-HSA:9854311
Transcriptional%20and%20post-translational%20regulation	1.405039596681751E-5	R-HSA:9856649
MITF-M-dependent%20gene%20expression	0.02917048749702113	R-HSA:9856651
Malate-aspartate%20shuttle	3.2492799205602557E-4	R-HSA:9856872
Regulation%20of%20MITF-M-dependent%20genes%20invol	2.4003492645736702E-5	R-HSA:9857377

Protein%20lipoylation	0.03493787212115158	R-HSA:9857492
PDH%20complex%20synthesizes%20acetyl-CoA%20from%	2.6107067003199453E-4	R-HSA:9861559
Regulation%20of%20pyruvate%20metabolism	0.01651527531911075	R-HSA:9861718
Complex%20III%20assembly	0.0023035003848349083	R-HSA:9865881

name	Term PValue Corrected with Benjamini-H	UNIQUE_ID
Apoptosis	0.032285379855300216	R-HSA:109581
Intrinsic%20Pathway%20for%20Apoptosis	0.03640015876644282	R-HSA:109606
Cytochrome%20c-mediated%20apoptotic%20response	0.03077941836978011	R-HSA:111461
Apoptotic%20factor-mediated%20response	0.02817738778075856	R-HSA:111471
Platelet%20degranulation%20	0.017316021878206047	R-HSA:114608
Aerobic%20respiration%20and%20respiratory%20electron%20transport	0.03502850716909681	R-HSA:1428517
Metabolism	2.783842857413548E-4	R-HSA:1430728
Extracellular%20matrix%20organization	0.0322963648151965	R-HSA:1474244
Glutathione%20conjugation	0.046466189073031405	R-HSA:156590
Metabolism%20of%20nucleotides	0.04890606133035483	R-HSA:15869
Transport%20of%20Mature%20mRNA%20derived%20from%20an%20Intron-Containin	0.018627279867871778	R-HSA:159236
HIV%20Life%20Cycle	0.04157605624452897	R-HSA:162587
Late%20Phase%20of%20HIV%20Life%20Cycle	0.030698987338338448	R-HSA:162599
PP2A-mediated%20dephosphorylation%20of%20key%20metabolic%20factors	0.032293221903259124	R-HSA:163767
Cell%20Cycle	0.018467376538773828	R-HSA:1640170
Disease	0.04175717465660903	R-HSA:1643685
MyD88:MAL(TIRAP)%20cascade%20initiated%20on%20plasma%20membrane	0.04947705503115662	R-HSA:166058
Toll%20Like%20Receptor%20TLR6:TLR2%20Cascade	0.04947705503115662	R-HSA:168188
Innate%20Immune%20System	0.047184009545942	R-HSA:168249
SRP-dependent%20cotranslational%20protein%20targeting%20to%20membrane	0.0326069815374156	R-HSA:1799339
DARPP-32%20events	0.02205908844509303	R-HSA:180024
Signaling%20by%20PDGF	0.04416119758411886	R-HSA:186797
snRNP%20Assembly	0.03254398990402931	R-HSA:191859
Metabolism%20of%20non-coding%20RNA	0.03254398990402931	R-HSA:194441
ERK/MAPK%20targets	0.03194047855949558	R-HSA:198753
ER%20to%20Golgi%20Anterograde%20Transport	0.017041728977493436	R-HSA:199977
Membrane%20Trafficking	0.017147580936152578	R-HSA:199991
Assembly%20of%20collagen%20fibrils%20and%20other%20multimeric%20structures	0.03110394719900843	R-HSA:2022090
ERKs%20are%20inactivated	0.03077941836978011	R-HSA:202670
Integrin%20cell%20surface%20interactions	0.04840319342321396	R-HSA:216083

Cellular%20responses%20to%20stress	0.005276693628432567	R-HSA:2262752
Selenoamino%20acid%20metabolism	0.025768346695481757	R-HSA:2408522
MASTL%20Facilitates%20Mitotic%20Progression	0.017717009334540117	R-HSA:2465910
Mitotic%20Metaphase%20and%20Anaphase	0.021731129328632234	R-HSA:2555396
Initiation%20of%20Nuclear%20Envelope%20(NE)%20Reformation	0.024990348646255302	R-HSA:2995383
Nuclear%20Envelope%20(NE)%20Reassembly	0.018627279867871778	R-HSA:2995410
Laminin%20interactions	0.029830001939537696	R-HSA:3000157
ECM%20proteoglycans	0.03283706291377266	R-HSA:3000178
Scavenging%20by%20Class%20A%20Receptors	0.024990348646255302	R-HSA:3000480
Glycogen%20synthesis	0.033001603260751265	R-HSA:3322077
Integrin%20signaling	0.02408799556758114	R-HSA:354192
GRB2:SOS%20provides%20linkage%20to%20MAPK%20signaling%20for%20Integrins%	0.03646864389235669	R-HSA:354194
Transcriptional%20Regulation%20by%20TP53	0.03194405399919193	R-HSA:3700989
p130Cas%20linkage%20to%20MAPK%20signaling%20for%20integrins	0.03646864389235669	R-HSA:372708
NCAM%20signaling%20for%20neurite%20out-growth	0.033035631927149575	R-HSA:375165
CTLA4%20inhibitory%20signaling	0.03043086994374417	R-HSA:389513
Metabolism%20of%20proteins	0.009207695633988296	R-HSA:392499
Axon%20guidance	0.016785594278522928	R-HSA:422475
Deadenylation-dependent%20mRNA%20decay	1.8880161142073435E-4	R-HSA:429914
mRNA%20decay%20by%203'%20to%205'%20exoribonuclease	0.04195902417660218	R-HSA:429958
mRNA%20decay%20by%205'%20to%203'%20exoribonuclease	0.017586923244728072	R-HSA:430039
Platelet%20sensitization%20by%20LDL	0.006716243284585191	R-HSA:432142
Smooth%20Muscle%20Contraction	0.03943813508117212	R-HSA:445355
Asparagine%20N-linked%20glycosylation	0.02818743056405486	R-HSA:446203
Synthesis%20of%20GDP-mannose	0.026335567668901057	R-HSA:446205
MAPK%20targets/%20Nuclear%20events%20mediated%20by%20MAP%20kinases	0.03284753842766893	R-HSA:450282
Butyrate%20Response%20Factor%201%20(BRF1)%20binds%20and%20destabilizes%	0.04730442120502561	R-HSA:450385
Mitotic%20G2-G2/M%20phases	0.04904359332420217	R-HSA:453274
Programmed%20Cell%20Death	0.04872571128296693	R-HSA:5357801
TP53%20Regulates%20Metabolic%20Genes	0.04723898496996881	R-HSA:5628897
Fructose%20metabolism	0.032293221903259124	R-HSA:5652084

Vesicle-mediated%20transport	0.00996277877449441	R-HSA:5653656
Diseases%20of%20signal%20transduction%20by%20growth%20factor%20receptors%	0.03543770700709452	R-HSA:5663202
Infectious%20disease	0.03246340881112912	R-HSA:5663205
RAF%20activation	0.046466189073031405	R-HSA:5673000
Regulation%20of%20TLR%20by%20endogenous%20ligand	0.03194047855949558	R-HSA:5686938
Respiratory%20electron%20transport	0.017519376237775018	R-HSA:611105
rRNA%20modification%20in%20the%20nucleus%20and%20cytosol	0.01716009127428512	R-HSA:6790901
Major%20pathway%20of%20rRNA%20processing%20in%20the%20nucleolus%20and%	0.025460446702795705	R-HSA:6791226
Signaling%20by%20moderate%20kinase%20activity%20BRAF%20mutants	0.045986472185865504	R-HSA:6802946
Signaling%20by%20high-kinase%20activity%20BRAF%20mutants	0.046466189073031405	R-HSA:6802948
Signaling%20by%20RAS%20mutants	0.045986472185865504	R-HSA:6802949
Signaling%20by%20BRAF%20and%20RAF1%20fusions	0.03641437116125538	R-HSA:6802952
Paradoxical%20activation%20of%20RAF%20signaling%20by%20kinase%20inactive%2	0.045986472185865504	R-HSA:6802955
COPI-mediated%20anterograde%20transport	0.032303979478631784	R-HSA:6807878
Mitotic%20Prophase	0.032184383060804116	R-HSA:68875
Mitotic%20Anaphase	0.021905342301370867	R-HSA:68882
M%20Phase	0.021703782367382473	R-HSA:68886
Cyclin%20A/B1/B2%20associated%20events%20during%20G2/M%20transition	0.041876234163061854	R-HSA:69273
G2/M%20Transition	0.04842934856210643	R-HSA:69275
Cell%20Cycle,%20Mitotic	0.02168314406519144	R-HSA:69278
Glycolysis	0.030479583649003734	R-HSA:70171
Glucose%20metabolism	0.04723898496996881	R-HSA:70326
Metabolism%20of%20amino%20acids%20and%20derivatives	0.006803327675130781	R-HSA:71291
Metabolism%20of%20carbohydrates	0.04825103104814824	R-HSA:71387
mRNA%20Splicing%20-%20Major%20Pathway	3.1770569039773987E-7	R-HSA:72163
mRNA%20Splicing	6.383198641071852E-7	R-HSA:72172
mRNA%203'-end%20processing	0.047197336404730485	R-HSA:72187
Transport%20of%20Mature%20Transcript%20to%20Cytoplasm	0.01733189514184999	R-HSA:72202
Processing%20of%20Capped%20Intron-Containing%20Pre-mRNA	1.2863864573279597E-7	R-HSA:72203
rRNA%20processing	0.017418965543868823	R-HSA:72312
Translation	0.036856802790047424	R-HSA:72766

Platelet%20activation,%20signaling%20and%20aggregation	0.036958991533182606	R-HSA:76002
Response%20to%20elevated%20platelet%20cytosolic%20Ca2+	0.01790211946767316	R-HSA:76005
Signaling%20by%20PTK6	0.020093446265703075	R-HSA:8848021
rRNA%20processing%20in%20the%20nucleus%20and%20cytosol	0.010071008918313544	R-HSA:8868773
Collagen%20chain%20trimerization	0.036675012554702416	R-HSA:8948216
Metabolism%20of%20RNA	1.653600516017647E-12	R-HSA:8953854
Cellular%20responses%20to%20stimuli	0.006796688699894478	R-HSA:8953897
Glycogen%20metabolism	0.049312230020330465	R-HSA:8982491
Loss%20of%20function%20of%20MECP2%20in%20Rett%20syndrome	0.033001603260751265	R-HSA:9005891
Pervasive%20developmental%20disorders	0.033001603260751265	R-HSA:9005895
Signaling%20by%20Non-Receptor%20Tyrosine%20Kinases	0.020093446265703075	R-HSA:9006927
Loss%20of%20MECP2%20binding%20ability%20to%20the%20NCoR/SMRT%20compl	0.032293221903259124	R-HSA:9022537
Regulation%20of%20MECP2%20expression%20and%20activity	0.018089301915738484	R-HSA:9022692
Transport%20to%20the%20Golgi%20and%20subsequent%20modification	0.03545172222555464	R-HSA:948021
Regulation%20of%20glycolysis%20by%20fructose%202,6-bisphosphate%20metabolis	0.026713025026585815	R-HSA:9634600
Signaling%20downstream%20of%20RAS%20mutants	0.045986472185865504	R-HSA:9649948
Signaling%20by%20RAF1%20mutants	0.03510154802392978	R-HSA:9656223
Nervous%20system%20development	0.017784086016500188	R-HSA:9675108
Disorders%20of%20Developmental%20Biology	0.033001603260751265	R-HSA:9675151
Disorders%20of%20Nervous%20System%20Development	0.033001603260751265	R-HSA:9697154
Cytoprotection%20by%20HMOX1	0.04816227552410113	R-HSA:9707564
Cellular%20response%20to%20chemical%20stress	0.0236541804627481	R-HSA:9711123
M-decay:%20degradation%20of%20maternal%20mRNAs%20by%20maternally%20sto	0.03675061929732784	R-HSA:9820841
Respiratory%20syncytial%20virus%20(RSV)%20genome%20replication,%20transcripti	0.04195902417660218	R-HSA:9820965
Viral%20Infection%20Pathways	0.02974680918396593	R-HSA:9824446
Maturation%20of%20hRSV%20A%20proteins	0.03077941836978011	R-HSA:9828806
Mitochondrial%20protein%20degradation	0.04848782603948083	R-HSA:9837999
